# Brain structural abnormalities in obesity: relation to age, genetic risk, and common psychiatric disorders: Evidence through univariate and multivariate mega-analysis including 6420 participants from the ENIGMA MDD working group

**DOI:** 10.1038/s41380-020-0774-9

**Published:** 2020-05-28

**Authors:** Nils Opel, Anbupalam Thalamuthu, Yuri Milaneschi, Dominik Grotegerd, Claas Flint, Ramona Leenings, Janik Goltermann, Maike Richter, Tim Hahn, Georg Woditsch, Klaus Berger, Marco Hermesdorf, Andrew McIntosh, Heather C. Whalley, Mathew A. Harris, Frank P. MacMaster, Henrik Walter, Ilya M. Veer, Thomas Frodl, Angela Carballedo, Axel Krug, Igor Nenadic, Tilo Kircher, Andre Aleman, Nynke A. Groenewold, Dan J. Stein, Jair C. Soares, Giovana B. Zunta-Soares, Benson Mwangi, Mon-Ju Wu, Martin Walter, Meng Li, Ben J. Harrison, Christopher G. Davey, Kathryn R. Cullen, Bonnie Klimes-Dougan, Bryon A. Mueller, Philipp G. Sämann, Brenda Penninx, Laura Nawijn, Dick J. Veltman, Lyubomir Aftanas, Ivan V. Brak, Elena A. Filimonova, Evgeniy A. Osipov, Liesbeth Reneman, Anouk Schrantee, Hans J. Grabe, Sandra Van der Auwera, Katharina Wittfeld, Norbert Hosten, Henry Völzke, Kang Sim, Ian H. Gotlib, Matthew D. Sacchet, Jim Lagopoulos, Sean N. Hatton, Ian Hickie, Elena Pozzi, Paul M. Thompson, Neda Jahanshad, Lianne Schmaal, Bernhard T. Baune, Udo Dannlowski

**Affiliations:** 1 Department of Psychiatry, University of Münster, Münster, Germany; 2 Interdisciplinary Centre for Clinical Research (IZKF), University of Münster, Münster, Germany; 3 Centre for Healthy Brain Ageing, School of Psychiatry, University of New South Wales, Sydney, NSW Australia; 4 Neuroscience Research Australia, Randwick, NSW Australia; 5 Department of Psychiatry, Amsterdam UMC/Vrije Universiteit, Amsterdam, Netherlands; 6 Faculty of Mathematics and Computer Science, University of Münster, Münster, Germany; 7 IT Department, University of Muenster, Münster, Germany; 8 Institute of Epidemiology and Social Medicine, University of Münster, Münster, Germany; 9 Division of Psychiatry, University of Edinburgh, Edinburgh, UK; 10 Psychiatry and Paediatrics, University of Calgary, Calgary, AB Canada; 11 Addictions and Mental Health Strategic Clinical Network Calgary, Calgary, AB Canada; 12 Division of Mind and Brain Research, Department of Psychiatry and Psychotherapy CCM, Charité - Universitätsmedizin Berlin, corporate member of Freie Universität Berlin, Humboldt-Universität zu Berlin, and Berlin Institute of Health, Berlin, Germany; 13 Department of Psychiatry, Trinity College Dublin, Dublin, Ireland; 14 Department of Psychiatry and Psychotherapy, Otto von Guericke University Magdeburg, Magdeburg, Germany; 15 Department of Psychiatry and Psychotherapy, University of Marburg, Marburg, Germany; 16 Department of Biomedical Sciences of Cells and Systems, University of Groningen, University Medical Center Groningen, Groningen, The Netherlands; 17 SA MRC Unit on Risk & Resilience, Department of Psychiatry and Neuroscience Institute, University of Cape Town, Cape Town, South Africa; 18 UT Center of Excellence on Mood Disorders, Department of Psychiatry and Behavioral Sciences, University of Texas Health Science Center at Houston, Houston, TX USA; 19 Department of Psychiatry, University of Texas Health Science Center at Houston, Houston, TX USA; 20 Department of Psychiatry and Psychotherapy, Jena University Hospital, Jena, Germany; 21 Melbourne Neuropsychiatry Centre, Department of Psychiatry, The University of Melbourne & Melbourne Health, Parkville, VIC Australia; 22 Orygen, The National Centre of Excellence in Youth Mental Health, Parkville, VIC Australia; 23 Centre for Youth Mental Health, The University of Melbourne, Parkville, VIC Australia; 24 Department of Psychiatry and Behavioral Sciences, School of Medicine, University of Minnesota, Minneapolis, MN USA; 25 Department of Psychology, University of Minnesota, Minneapolis, MN USA; 26 Max Planck Institute of Psychiatry, Munich, Germany; 27 FSSBI “Scientific Research Institute of Physiology & Basic Medicine”, Laboratory of Affective, Cognitive & Translational Neuroscience, Novosibirsk, Russia; 28 Novosibirsk State University, Laboratory of Experimental & Translational Neuroscience, Novosibirsk, Russia; 29 Department of Radiology and Nuclear Medicine, Academic Medical Center, University of Amsterdam, Amsterdam, Netherlands; 30 Department of Psychiatry and Psychotherapy, University Medicine Greifswald, Greifswald, Germany; 31 German Center for Neurodegenerative Diseases (DZNE), Greifswald/Rostock, site Greifswald, Greifswald, Germany; 32 Institute of Diagnostic Radiology and Neuroradiology, University Medicine Greifswald, Greifswald, Germany; 33 Institute for Community Medicine, University Medicine Greifswald, Greifswald, Germany; 34 German Center for Cardiovascular Research (DZHK), Partner Site Greifswald, Greifswald, Germany; 35 West Region, Institute of Mental Health, Singapore, Singapore; 36 Yoo Loo Lin School of Medicine, National University of Singapore, Singapore, Singapore; 37 Department of Psychology, Stanford University, Stanford, CA USA; 38 Center for Depression, Anxiety, and Stress Research, McLean Hospital, Harvard Medical School, Belmont, MA USA; 39 Sunshine Coast Mind and Neuroscience Thompson Institute, University of the Sunshine Coast, Sippy Downs, QLD Australia; 40 Brain and Mind Centre, University of Sydney, Camperdown, NSW Australia; 41 Melbourne Neuropsychiatry Centre, Department of Psychiatry, The University of Melbourne, Parkville, VIC Australia; 42 Mark & Mary Stevens Neuroimaging & Informatics Institute, Keck School of Medicine, University of Southern California, Marina del Rey, CA USA; 43 Department of Psychiatry, Melbourne Medical School, The University of Melbourne, Melbourne, VIC Australia; 44 The Florey Institute of Neuroscience and Mental Health, The University of Melbourne, Parkville, VIC Australia

**Keywords:** Diagnostic markers, Depression

## Abstract

Emerging evidence suggests that obesity impacts brain physiology at multiple levels. Here we aimed to clarify the relationship between obesity and brain structure using structural MRI (*n* = 6420) and genetic data (*n* = 3907) from the ENIGMA Major Depressive Disorder (MDD) working group. Obesity (BMI > 30) was significantly associated with cortical and subcortical abnormalities in both mass-univariate and multivariate pattern recognition analyses independent of MDD diagnosis. The most pronounced effects were found for associations between obesity and lower temporo-frontal cortical thickness (maximum Cohen´s *d* (left fusiform gyrus) = −0.33). The observed regional distribution and effect size of cortical thickness reductions in obesity revealed considerable similarities with corresponding patterns of lower cortical thickness in previously published studies of neuropsychiatric disorders. A higher polygenic risk score for obesity significantly correlated with lower occipital surface area. In addition, a significant age-by-obesity interaction on cortical thickness emerged driven by lower thickness in older participants. Our findings suggest a neurobiological interaction between obesity and brain structure under physiological and pathological brain conditions.

## Introduction

With an estimated worldwide prevalence of 13% among the adult population and up to 38% in western societies [1], obesity is one of the greatest concerns to public health [2]. The role of obesity as a preventable cardiovascular risk factor is well known, but research has only recently started to explore the neurobiological underpinnings of obesity.

On a systemic level, neuroimaging research has identified structural [3–5] and functional [6–8] alterations in obese participants—one of the most consistent findings is decreased gray matter volume in obesity [3, 4, 9, 10]. A recent UK Biobank study including data from *n* = 9652 participants supplemented this notion by showing an inverse association between BMI and global gray matter volume [11]. Further large-scale evidence for associations between body weight and brain structure comes from a recent meta-analysis of voxel-based morphometry studies including data from *n* = 5882 subjects that pointed to consistent associations between BMI and lower gray matter volume in the medial prefrontal cortex, the bilateral cerebellum, and the left temporal pole [12]. However, even though these well-powered studies provide robust evidence for an association between BMI and brain structure in general, the current understanding of the relationship between obesity and brain structure is considerably limited for several reasons.

First, the distribution and effect size of brain structural abnormalities in obesity remains unclear. Several smaller structural neuroimaging studies suggest that obesity might primarily relate to gray matter reductions in brain areas involved in reward processing and impulse regulation such as the orbitofrontal cortex and the striatum [9, 13, 14]. Even so, other reports question the hypothesis of regional specific gray matter decrease in obesity by pointing to widespread associations throughout the brain with diverging effects of obesity on subcortical brain structure [4, 10]. Since prior studies either exhibited limited power to detect subtle effects in small samples or employed hypothesis-driven region of interest approaches, the distribution or regional specificity of obesity-related brain structural abnormalities remains uncertain. Large-scale studies are needed that investigate associations with obesity throughout the entire brain by differentiating effects on subcortical volume and cortical thickness and surface area. Furthermore, while the statistical significance of obesity-related brain structural abnormalities is well documented, the effect sizes and hence the potential relevance of brain structural alterations in obesity remains unknown. We aimed to address this issue by directly comparing profiles of obesity-related brain structural alterations with findings from neuropsychiatric disorders. In addition we aimed to complement group level analyses, by employing individual-level based pattern classification as a further proxy for the robustness of neuroimaging findings [15]. Second, previous neuroimaging findings in obesity are largely based on studies in healthy participants. Yet, obesity has frequently been associated with neuropsychiatric disorders [16, 17] and more specifically previous research has pointed to a bidirectional association between obesity and major depression [18]. Furthermore, preliminary neuroimaging studies have reported overlapping brain structural abnormalities in obesity and major depression [9, 12, 19]. It thus appears relevant to investigate if obesity-related brain structural abnormalities might similarly be present under physiological and pathological brain conditions. Against this backdrop, the present study aimed to provide a well-powered and comprehensive investigation of the relationship between obesity and brain structural abnormalities in healthy participants and depressive patients. A third major issue concerns the relationship between brain structural abnormalities in obesity and ageing. Interestingly, while obesity and gray matter volume are frequently reported to be inversely related in adult samples, the few studies of obesity-related brain structural abnormalities in children and adolescents have diverging results [13, 20, 21]. Thus, it is valuable to investigate whether brain structural impairment in obesity is already detectable in children and adolescents and if brain structural abnormalities in obesity might vary as a function of age. In addition, there may be a genetic contribution to brain structural abnormalities in obesity, given the high heritability of obesity in general [22] and the involvement of multiple BMI-related genetic variants in brain physiology [23]. Thus, the question of a potential genetic contribution to brain structural abnormalities in obesity arises. To address this, we combined individual polygenic risk profiles with imaging data to investigate obesity and BMI-related brain structural abnormalities [24, 25].

## Methods

### Participants

We studied BMI and neuroimaging data in a combined sample of 6420 participants (mean age = 42.91, SD = 15.26; 56.95% female; mean BMI = 25.97, SD = 4.97) including healthy controls (HC: *n* = 3519) and major depressive disorder patients (MDD: *n* = 2901) from 28 sites contributing to the ENIGMA MDD working group [19, 26]. The sample included *n* = 1223 obese participants (BMI > 30) as well as *n* = 2917 normal weight participants (BMI 18.5–25) (Supplementary Results, Supplementary Figs. 1, 2, 3, Supplementary Tables 1, 2). All participating sites obtained approval from local institutional review boards and ethics committees; all study participants provided written informed consent.

### Structural MRI methods

T1-weighted high-resolution anatomical brain images were acquired for all participants and preprocessed locally using FreeSurfer segmentation. Quality control was carried out at each site according to protocols from the ENIGMA consortium. Segmentation quality was assessed by visual inspection and statistically evaluated for outliers with a standardized protocol provided by the ENIGMA consortium (http://enigma.ini.usc.edu/protocols/imaging-protocols). Details of the imaging procedures for each cohort may be found in the Supplementary material (Supplementary Table 3). All structural images were preprocessed using the subcortical and cortical parcellation stream of FreeSurfer with the default parameters [27]. As we aimed to provide a comprehensive overview of obesity-related brain structural alterations that would allow for comparison with previous ENIGMA studies, all available imaging measures were included for the presented analyses: global measures included total intracranial volume, total left and right cortical surface area, and average left and right cortical thickness. Regional measures included subcortical volumetric measures (8 left and 8 right), surface area (34 left and 34 right), and thickness measures (34 left and 34 right) for all cortical regions based on the Desikan–Killiany atlas [28]. The presented morphometric data allowed us to simultaneously investigate both subcortical and cortical abnormalities and furthermore enabled us to examine thickness and surface area separately which have been shown to be driven by distinct genetic mechanisms and to exhibit different developmental trajectories [29, 30].

### Genetic methods

Genetic data were available for 3907 individuals from nine contributing sites. Genotyping of these subjects was performed at each contributing site using published protocols (Supplementary Table 4). Polygenic risk scores (PRS) were generated using sets of SNPs selected based on *p* value thresholds at *p* = [0.1; 0.2; 0.3; 0.4; 0.5; 0.6; 0.7; 0.8; 0.9; 1.0] from the base GWAS data. The R program ‘PRSice' [31]—which uses PLINK-1.9 [32] in the background for linkage disequilibrium pruning—was used for this analysis step. Standardized PRS values based on z-transformation were used for all analyses (Supplementary Methods).

### Statistical analyses

All univariate imaging analyses were carried out using linear models in R, separately for each of the 157 available FreeSurfer derived imaging measures as a dependent variable. Age, sex, MDD diagnosis, and site were included as covariates in all models. For analyses of subcortical volumes and surface area measures, ICV was also included as covariate. For all univariate imaging analyses, FDR correction for 157 tests was conducted using the Benjamini Hochberg procedure with a false discovery rate of *q* < 0.05.

To investigate associations between brain structure and obesity, two main models were applied by including a dichotomous predictor based on a BMI threshold (obese subjects (BMI > 30) versus normal weight subjects (BMI 18.5–25) (Model A)) and furthermore by including BMI as a continuous predictor (Model B).

Effect size estimates (Cohen´s *d*) were calculated based on *t*-values and sample sizes [33] from the regression model including the dichotomous BMI group (obesity versus normal weight) predictor (Model A) thus following a similar methodology compared with previous studies on psychiatric disorders from the ENIGMA consortium [19, 26]. To investigate potential similarities between brain structural alterations in obesity and common neuropsychiatric disorders, we carried out correlational analyses between effect size estimates (Cohen´s *d*) of thickness alterations in all cortical regions in obesity with effect size estimates reported in previous ENIGMA studies on MDD [19] and bipolar disorder [34].

To further test our hypothesis of brain structural alterations in obesity, we complemented the applied mass-univariate testing approach by conducting pattern recognition analyses to investigate multivariate patterns of brain structural differences between obese and normal weight subjects. To this end, a machine learning pipeline consisting of several preprocessing steps including imputation of missing values, dimensionality reduction by principal component analysis and random undersampling and a support vector machine was trained on all available 157 FreeSurfer derived imaging measures to individually classify participants as either obese or normal weight using pooled multisite nested cross-validation employing the PHOTON framework (https://photon-ai.com; Supplementary Methods).

Furthermore, potential interaction effects of body weight and age, sex and MDD diagnosis were carried out as exploratory analyses. In addition, associations between polygenic risk for obesity and brain structure were assessed through univariate models as outlined above.

## Results

### Obesity and brain structure

**Fig. 1:**
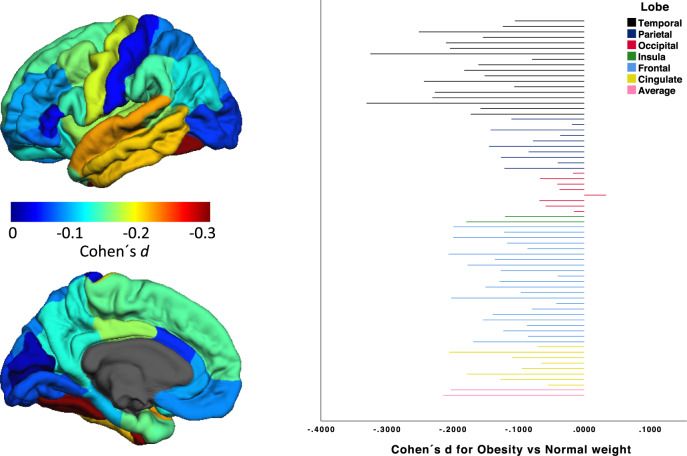
Figure displaying effect sizes for the association between obesity and cortical thickness on left hemispheral thickness. Colorbar displays effect size estimates (Cohen´s *d*) for differences in cortical thickness between obese versus normal weight subjects; Bar diagram depicts effect sizes for all cortical regions sorted by lobe.

Linear regression models including either obesity as dichotomous predictor (Model A) or BMI as continuous predictor (Model B) of brain structure yielded highly consistent results (Supplementary Tables 5, 6, Supplementary Fig. 4). Obesity was associated with lower cortical thickness, with most pronounced and consistent associations between obesity and lower cortical thickness in regions of the temporal and frontal lobe (Table 1 and Fig. 1). Analyses of regionally specific cortical surface area alterations in obesity revealed both significantly lower and higher surface area in obese subjects. Subcortical volumes were found to be significantly increased in obese subjects—with most pronounced volume increases in the amygdala, the thalamus and the nucleus accumbens (Table 1).

**Table 1:** FDR-corrected significant results for group differences between obese and normal weight subjects as assessed using separate linear regression models with a dichotomous group predictor (obesity versus normal weight).

Label	Estimate	Std error	*T*	*p*	FDR adjusted *p*	Cohen´s *d*	*N* Obese	*N* NW
Global measures								
Left hemispheral average thickness	−0.021	0.003	−6.23	5.18E−10	<0.0001	−0.214	1200	2865
Right hemispheral average thickness	−0.020	0.003	−5.89	4.18E−09	<0.0001	−0.203	1200	2865
Total Intracranial Volume	−21634.000	5603.000	−3.86	1.10E−04	0.0005	−0.135	1168	2755
Total right hemispheral surface area	−708.380	258.090	−2.74	6.08E−03	0.0165	−0.095	1189	2872
Total left hemispheral surface area	−654.300	256.890	−2.55	1.09E−02	0.0281	−0.088	1189	2872
Cortical thickness								
Left fusiform gyrus	−0.051	0.005	−9.59	2.00E−16	<0.0001	−0.331	1195	2849
Right fusiform gyrus	−0.050	0.005	−9.42	2.00E−16	<0.0001	−0.325	1193	2849
Right superior temporal gyrus	−0.041	0.006	−7.17	9.09E−13	<0.0001	−0.251	1161	2745
Left superior temporal gyrus	−0.040	0.006	−6.88	7.04E−12	<0.0001	−0.243	1138	2684
Left inferior temporal gyrus	−0.040	0.006	−6.62	4.17E−11	<0.0001	−0.231	1165	2823
Left middle temporal gyrus	−0.039	0.006	−6.46	1.18E−10	<0.0001	−0.227	1149	2748
Right middle temporal gyrus	−0.036	0.006	−6.06	1.49E−09	<0.0001	−0.210	1184	2815
Right pars opercularis	−0.033	0.006	−5.96	2.70E−09	<0.0001	−0.206	1189	2835
Right posterior cingulate cortex	−0.033	0.006	−5.96	2.71E−09	<0.0001	−0.205	1196	2859
Right inferior temporal gyrus	−0.036	0.006	−5.88	4.54E−09	<0.0001	−0.204	1175	2838
Left precentral gyrus	−0.030	0.005	−5.85	5.27E−09	<0.0001	−0.202	1192	2837
Right precentral gyrus	−0.030	0.005	−5.76	9.13E−09	<0.0001	−0.199	1188	2844
Right superior frontal gyrus	−0.030	0.005	−5.76	8.93E−09	<0.0001	−0.199	1189	2859
Left transverse temporal gyrus	−0.042	0.008	−5.29	1.26E−07	<0.0001	−0.182	1195	2853
Left insula	−0.030	0.006	−5.17	2.41E−07	<0.0001	−0.179	1188	2811
Left posterior cingulate cortex	−0.030	0.006	−5.16	2.56E−07	<0.0001	−0.178	1196	2857
Right medial orbitofrontal cortex	−0.031	0.006	−5.12	3.18E−07	<0.0001	−0.177	1183	2831
Left banks of the superior temporal sulcus	−0.031	0.006	−4.88	1.08E−06	<0.0001	−0.172	1139	2708
Left caudal middle frontal gyrus	−0.026	0.005	−4.89	1.04E−06	<0.0001	−0.169	1196	2840
Right banks of the superior temporal sulcus	−0.030	0.006	−4.63	3.81E−06	<0.0001	−0.161	1178	2796
Left entorhinal cortex	−0.061	0.013	−4.5	6.86E−06	<0.0001	−0.158	1164	2725
Left paracentral lobule	−0.024	0.005	−4.46	8.55E−06	<0.0001	−0.154	1195	2857
Right parahippocampal gyrus	−0.044	0.010	−4.46	8.50E−06	<0.0001	−0.154	1192	2850
Left temporal pole	−0.059	0.014	−4.38	1.20E−05	0.0001	−0.151	1187	2851
Left superior frontal gyrus	−0.023	0.005	−4.35	1.36E−05	0.0001	−0.150	1194	2851
Left supramarginal gyrus	−0.021	0.005	−4.15	3.45E−05	0.0002	−0.145	1173	2767
Right precuneus	−0.019	0.005	−4.13	3.75E−05	0.0002	−0.142	1195	2848
Left pars opercularis	−0.021	0.005	−4.03	5.58E−05	0.0003	−0.139	1194	2845
Right paracentral lobule	−0.022	0.005	−3.94	8.38E−05	0.0004	−0.136	1196	2857
Right caudal middle frontal gyrus	−0.020	0.005	−3.73	2.00E−04	0.0008	−0.129	1194	2845
Left isthmus cingulate cortex	−0.026	0.007	−3.7	2.20E−04	0.0009	−0.128	1195	2852
Right lateral orbitofrontal cortex	−0.022	0.006	−3.68	2.40E−04	0.0009	−0.127	1195	2858
Left precuneus	−0.017	0.005	−3.66	2.60E−04	0.0010	−0.126	1189	2851
Right temporal pole	−0.050	0.014	−3.58	3.40E−04	0.0012	−0.124	1191	2850
Left lateral orbitofrontal cortex	−0.021	0.006	−3.56	3.70E−04	0.0013	−0.123	1188	2851
Right rostral middle frontal gyrus	−0.017	0.005	−3.53	4.10E−04	0.0014	−0.122	1192	2849
Left inferior parietal cortex	−0.017	0.005	−3.5	4.80E−04	0.0016	−0.121	1180	2831
Right insula	−0.022	0.006	−3.46	5.40E−04	0.0018	−0.120	1182	2777
Right pars triangularis	−0.020	0.006	−3.39	7.20E−04	0.0023	−0.117	1187	2838
Right isthmus cingulate cortex	−0.022	0.007	−3.18	1.50E−03	0.0045	−0.110	1196	2854
Right supramarginal gyrus	−0.016	0.005	−3.18	1.50E−03	0.0045	−0.111	1178	2780
Left parahippocampal gyrus	−0.035	0.011	−3.08	2.08E−03	0.0060	−0.106	1190	2850
Right transverse temporal gyrus	−0.025	0.008	−3.05	2.30E−03	0.0066	−0.105	1190	2849
Left rostral middle frontal gyrus	−0.014	0.005	−2.8	5.10E−03	0.0140	−0.097	1197	2848
Left rostral anterior cingulate cortex	−0.023	0.009	−2.74	6.20E−03	0.0165	−0.095	1189	2835
Left medial orbitofrontal cortex	−0.015	0.006	−2.51	1.22E−02	0.0309	−0.087	1182	2818
Left frontal pole	−0.028	0.011	−2.49	1.28E−2	0.0313	−0.086	1199	2863
Right pars orbitalis	−0.020	0.008	−2.5	1.26E−02	0.0313	−0.086	1198	2848
Left superior parietal cortex	−0.010	0.004	−2.44	1.50E−02	0.0350	−0.084	1187	2831
Left pars orbitalis	−0.019	0.008	−2.31	2.11E−02	0.0473	−0.080	1194	2854
Cortical surface area								
Left isthmus cingulate cortex	25.900	5.492	4.72	2.50E−06	<0.0001	0.167	1134	2700
Right isthmus cingulate cortex	21.160	5.097	4.15	3.37E−05	0.0002	0.147	1137	2706
Left transverse temporal gyrus	10.183	2.603	3.91	9.32E−05	0.0004	0.138	1141	2708
Right rostral middle frontal gyrus	−71.936	22.908	−3.14	1.70E−03	0.0050	−0.111	1135	2698
Right paracentral lobule	21.589	7.403	2.92	3.57E−03	0.0100	0.104	1117	2688
Left inferior temporal gyrus	−40.910	15.209	−2.69	7.18E−03	0.0188	−0.096	1099	2673
Right inferior temporal gyrus	−35.140	14.136	−2.49	1.30E−02	0.0313	−0.089	1115	2684
Left paracentral lobule	16.023	6.589	2.43	1.51E−02	0.0350	0.087	1099	2658
Left lingual gyrus	−32.434	13.793	−2.35	1.88E−02	0.0428	−0.083	1128	2692
Subcortical volume								
Right amygdala	41.656	6.984	5.96	2.68E−09	<0.0001	0.211	1129	2702
Left thalamus	108.695	26.117	4.16	3.23E−05	0.0002	0.147	1138	2691
Right thalamus	80.814	22.216	3.64	2.80E−04	0.0010	0.129	1134	2680
Left amygdala	22.444	6.474	3.47	5.30E−04	0.0018	0.123	1127	2694
Left nucleus accumbens	11.724	3.541	3.31	9.40E−04	0.0030	0.118	1110	2660
Right hippocampus	33.557	13.810	2.43	1.52E−02	0.0350	0.086	1136	2709

Results are displayed for global measures, cortical thickness, and surface area as well as for subcortical volumes and sorted by *p* value within each domain. All results are adjusted for age, sex, MDD diagnosis, and site. Regional surface and subcortical results are adjusted for total intracranial volume.

*Estimate* regression estimate, *StdError* standard error, *T* t-value, *p* uncorrected *p* value, *FDR adjusted p* FDR adjusted *p* value, *N Obese* number of obese subjects included in analysis, *N NW* number of normal weight subjects included in analysis.

To rule out bias due to antidepressant medication intake in the MDD group, analyses were repeated by including current intake of antidepressant medication as additional nuisance regressor. Regional specificity of cortical thickness findings was assessed by conducting additional analyses accounting for mean cortical thickness. Highly similar results were observed in analyses controlling for the presence of antidepressant medication and in analyses adjusted for mean cortical thickness (Supplementary Tables 7, 8). Consistent results were observed in confirmatory analyses testing quadratic effects of BMI, in analyses accounting for quadratic effects of age and in analyses assessing the effect of weight group by including normal weight, overweight and obesity as categorial predictor (Supplementary Results and Supplementary Tables 9–11, Supplementary Fig. 5). Subsample analyses adjusting for head movement confirmed the overall pattern of results although obesity-related brain structural abnormalities were attenuated in these analyses (Supplementary Results and Supplementary Table 12, Supplementary Fig. 5).

Similar regional effect sizes for the association between obesity and brain structural abnormalities in the left and right hemisphere could be observed in the present study (Supplementary Results), while descriptively larger effects were observed for the association between obesity and lower cortical thickness in the left compared with right cortical hemisphere.

### Comparison of obesity-related brain structural abnormalities with previous findings in neuropsychiatric disorders

**Fig. 2:**
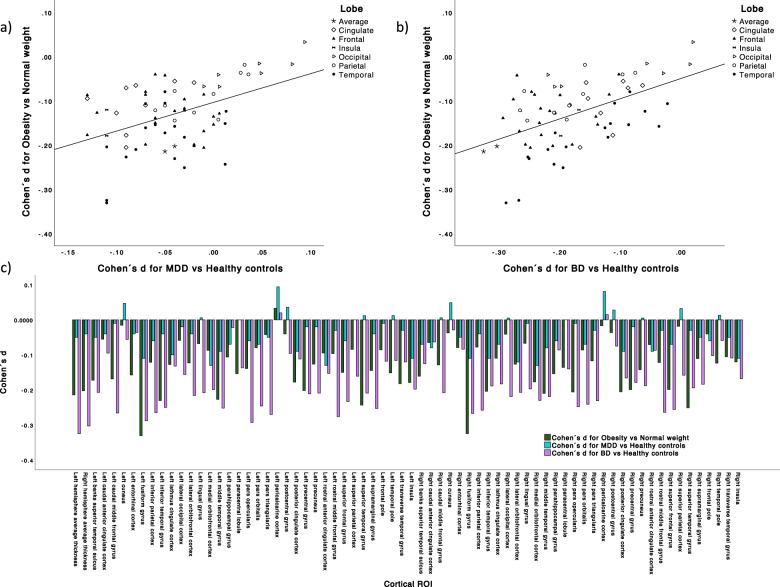
Effect size estimates (Cohen´s *d*) for differences in cortical thickness between obese versus normal weight subjects in direct comparison with previously published effect size estimates for cortical thickness results in major depression (MDD) and bipolar disorder (BD). **a** Plot depicting the positive correlation between effect size estimates for thickness results in all cortical regions mapped to the respective lobe between obesity and MDD (*r* = 0.452) and **b** between obesity and BD (*r* = 0.513). **c** Bar diagram displaying effect size estimates for cortical thickness results separately for all cortical regions.

Correlational analyses of effect size estimates for thickness of each cortical region of interest indicated similarities in the relative distribution or pattern of cortical thickness reductions across cortical regions between obesity and MDD (*r* = 0.452) and obesity and bipolar disorder (*r* = 0.513) (Fig. 2). An additional sensitivity analysis revealed that by contrast to the observed similarities between cortical thickness in obesity and affective disorders, effect sizes for obesity and previously published effect sizes for autism spectrum disorder (ASD) [35] did not show a similar degree of overlap (*r* = 0.149) (Supplementary Results). Further analyses of the absolute extent of effect sizes for cortical thickness indicated overall larger effect sizes in obesity compared with MDD and ASD but lower effect sizes compared with BD (Supplementary Results, Supplementary Fig. 6).

### Multivariate pattern recognition analyses

Multivariate pattern classification analyses further confirmed the relationship between obesity and brain structure by yielding highly significant single-subject differentiation between obese (BMI > 30, *n* = 1223) and normal weight subjects (BMI 18.5–25, *n* = 2917) with a balanced accuracy rate of 68.7% (BAC = 0.687, StD = 0.019, *p* < 0.001; sensitivity = 0.695; specificity = 0.678; F1score = 0.565; ROC-AUC = 0.687).

To rule out bias due to differing age, sex, and MDD diagnosis distributions in obese versus normal weight subjects, pattern recognition analyses were repeated in samples of obese and normal weight subjects that were balanced for age, sex, and MDD diagnosis using the pairmatch function in R (*n*_obese_ = 1223; *n*_normal weight_ = 1223). Similar results were observed when analyses were performed in samples of obese and normal weight subjects that were balanced for age, sex, and MDD diagnosis (*n*_obese_ = 1223; *n*_normal weight_ = 1223; BAC = 0.641, StD = 0.014, *p* < 0.001; sensitivity = 0.666; specificity = 0.617; F1score = 0.650; ROC-AUC = 0.641).

In addition, to demonstrate replicability across differing cohorts and scanning sites, we performed pattern recognition analyses by employing leave-one-site-out cross-validation. For this analysis step, only sites with a minimum of 50 subjects per group were included, to avoid bias due to lenient test sample sizes (*n*_obese_ = 960; *n*_normal weight_ = 1616; *k* = 5 sites). Analyses employing leave-one-site-out-cross-validation including all sites with a minimum *n* > 50 in each group yielded a lower but still highly significant accuracy rate (*n*_obese_ = 960; *n*_normal weight_ = 1616, *k* = 5 sites; BAC = 0.595, StD = 0.018, *p* < 0.001; sensitivity = 0.714; specificity = 0.476; F1score = 0.523; ROC-AUC = 0.595).

Supplementary analyses confirmed the predictive relevance of brain regions associated with obesity in the univariate analyses but also revealed that optimal classifier performance was obtained in analyses including the maximum of available brain structural features (see Supplementary Results).

### Moderating role of MDD diagnosis, age, and sex

To investigate if associations between BMI and brain structure would significantly differ between MDD and HC participants, interaction effects of BMI × MDD diagnosis were assessed based on linear models in analogy to Model B thus comparing slopes of BMI × MRI measure between MDD and HC subjects. No FDR-corrected significant interaction effect of BMI and MDD diagnosis was detected (Supplementary Table 13). Similarly, analyses stratified by diagnostic group confirmed our main result by yielding significant associations between obesity and lower temporo-frontal cortical thickness in both MDD and HC subjects with descriptively larger effect sizes for obesity in MDD compared with HC subjects (Supplementary Tables 14, 15, Supplementary Fig. 7).

Similarly, a moderating role of sex was investigated by assessing BMI × sex interaction effects. We observed FDR-corrected significant interaction effects of sex and BMI on cortical thickness, subcortical volumes, and surface area. The most consistent finding was a significantly enhanced BMI-related cortical thinning in male compared with female subjects (Supplementary Table 16).

To investigate a potential moderating role of age on brain structural alterations observed in obesity, linear models building on Model A were fitted by also including the obesity × age interaction term. FDR-corrected significant interaction effects of obesity and age were observed on cortical thickness of the left rostral middle frontal gyrus, the left lateral orbitofrontal gyrus, the left pars orbitalis, and triangularis of the inferior frontal gyrus driven by significantly enhanced age-related thickness decrease in obese compared with normal weight subjects. Further significant obesity × age interaction effects were observed for right hippocampal and left thalamic volume as well as for surface area of the right precuneus (Supplementary Table 17). Moreover, to investigate if brain structural associations with BMI could be detected in adolescents, regression analyses were repeated in the subgroup of participants with an age <21 (*n* = 520). Due to the limited prevalence of obesity in the adolescent subgroup (*n* = 51), only models including BMI as continuous predictor were conducted in the adolescent subgroup. Additional subgroup analyses of associations between BMI and brain structure in adolescent participants exclusively revealed an FDR-corrected significant positive association between BMI and volume of the right amygdala (*B* = 7.34, StdE = 1.72, *t* = 4.26, *p* = 0.00002, *p*_(FDR)_ = 0.0038, *n* = 503) (Supplementary Table 18), while no further association reached FDR-corrected significance in this subsample.

### Polygenic risk for obesity and brain structure

All calculated PRS scores significantly predicted BMI with proportions of explained variance (R^2^) ranging from 1.2 to 1.8% (*n* = 3907, all *p* < 0.00001; Supplementary Tables 19, 20). To assess the influence of polygenic risk for obesity on brain structure, linear models were fitted (a) by including the PRS based on information from all available SNPs as predictor (*p* value threshold = 1.0) and (b) by employing the polygenic score that explained most variance in BMI as predictor (*p* value threshold = 0.2).

We observed an FDR-corrected significant negative association between PRS_(p1.0)_ and cortical surface area of the left lateral occipital cortex (*B* = −45.92, StdE = 12.56, *t* = −3.66, *p* = 0.00026, *p*_(FDR)_ = 0.041, *n* = 3526) (Supplementary Table 21). Analyses including the PRS_(p0.2)_ as predictor yielded a highly similar pattern of results with the most pronounced association between polygenic risk and surface area of the left lateral occipital surface area, which, however, did not reach FDR-corrected significance (*B* = −40.84, StdE = 11.52, *t* = −3.55, *p* = 0.0004, *p*_(FDR)_ = 0.062, *n* = 3526) (Supplementary Table 22). In addition, mediation analyses were performed to test if the association between polygenic risk and BMI was mediated by left lateral occipital surface area and other brain structures reported previously [24]. While we did not observe a significant mediation effect for left lateral occipital surface area, a significant mediation effect of polygenic risk for obesity on BMI through left lateral orbitofrontal thickness could be detected (see Supplementary Results).

## Discussion

In the present multisite study, we found that obesity significantly associated with cortical and subcortical brain structural abnormalities independent of MDD diagnosis in both univariate and multivariate analyses. We further demonstrate that the regional distribution and effect size of the observed lower cortical thickness in obesity shows considerable similarities with corresponding patterns of cortical thickness alterations that have been described in mental disorders. Similarly, the presence of differential age dependent effects on brain structural measures in obesity—as well as the observed influence of polygenic risk for obesity on brain structure—offers novel insights of relevance for future experimental research on the etiology of obesity-related brain structural impairment.

The applied multisite design combined with a comprehensive neuroimaging approach allowed to differentiate between obesity-related abnormalities in cortical thickness, surface, and subcortical volume with unprecedented statistical power and detail. Our findings clarify that lower fronto-temporal cortical thickness constitutes the most pronounced obesity-related brain structural abnormality across the brain. This finding is supported by prior reports on temporal and frontal cortical gray matter decrease in obesity [4, 9, 10, 20, 24, 36].

Interestingly, while all significant associations between BMI and cortical thickness were negative, differing directions of associations occurred with regard to surface area alterations. This observation appears to match previously reported differential regionally specific positive and negative associations between cortical thickness and surface area [29, 37]. A previously discussed explanation for the inverse relationship between cortical surface and thickness measures refers to a potential stretching of the cortical surface area along the tangential axis due to intracortical myelination [37, 38]. Our finding of larger subcortical volumes in obesity with strongest effects of greater amygdala, thalamic, nucleus accumbens, and hippocampal volume finds support in prior studies of obese subjects that applied a similar volumetric imaging approach reporting larger amygdala, thalamus, and hippocampal volumes [39, 40]. In contrast, previous voxel-based morphometry studies reported negative associations between BMI and gray matter of subcortical structures [10, 41]. The disparity between volumetric and voxel-based findings has been directly investigated in a recent report by Perlaki et al. suggesting that BMI associates with higher amygdala and nucleus accumbens volumes derived from FreeSurfer segmentations but with lower VBM based GM density in identical structures highlighting the relevance to distinguish GM density from volume [13].

Importantly, we found that cortical thickness reductions in obesity are of similar effect size to the previously observed thickness reductions in several neuropsychiatric disorders. More specifically, peak effect sizes for lower cortical thickness in obesity (max. Cohen´s *d* (left fusiform gyrus) = −0.331) exceeded previously reported peak effect sizes for cortical thinning in MDD patients (max. Cohen´s *d* (left medial orbitofrontal cortex) = −0.134) [19], adult OCD patients (max Cohen´s *d* (right inferior parietal cortex) = −0.140) [42], findings in specific substance dependence (max Cohen´s *d* (right fusiform gyrus) = −0.094) [43] and were comparable with peak effect sizes in bipolar disorder (max Cohen´s *d* (left pars opercularis) = −0.293) [34]. Results of our pattern classification analyses further support the notion of a robust association between obesity and brain structure by yielding sMRI-based single-subject classification accuracies of up to 68.7% in pooled multisite cross-validation. Of note, this level of accuracy is comparable with pattern classification results reported for the detection of bipolar patients versus healthy controls using similar methods (65.2% accuracy for support vector classifiers, trained on FreeSurfer segmentations using multisite pooled cross-validation) [44]. Similar to previous reports of accurate individual brain age prediction based on neuroanatomical data [45, 46], our findings highlight the importance to consider multivariate morphometric patterns related to phenotypes such as age and body weight in future pattern classification studies. Importantly, the presence of a multivariate pattern differentiating obese from normal weight subjects could similarly be demonstrated in analyses controlling for age, sex and MDD diagnosis and by transfer of the classifier across cohorts using leave-one-site-out-cross-validation in the present work which underlines the robustness and the replicability of obesity-related brain structural abnormalities across sites. In addition, the relative distribution of obesity-related thickness reductions across all brain regions with most pronounced effects on temporo-frontal cortical regions revealed considerable similarities with patterns of thickness reductions in major depression [19] and bipolar disorder [34] while the absolute extent of effect sizes across all regions in obesity was larger compared with MDD but lower compared with BD. In sum, these findings offer novel insights into shared brain structural abnormalities in obesity and affective disorders. In light of the known bidirectional association between obesity and affective disorders such as MDD [18], future studies should investigate the potential clinical relevance of the shared morphometric signature observed here.

Of note, no significant interaction of BMI and MDD diagnosis on brain structure was observed in the present work and similar obesity-related brain structural abnormalities emerged in separate analyses in the HD and MDD subsamples. We thus conclude that associations between brain structure and BMI are not significantly altered by the presence of depression. This is well in line with previous findings reporting similar associations between BMI and gray matter reductions in MDD patients and healthy subjects alike and no evidence for interaction effects of body weight and depression on brain structure [9, 47].

Furthermore, we observed that cortical thickness effects of obesity were significantly moderated by age. This interaction was driven by enhanced reductions of obesity-related cortical thickness with increasing age. Complementary to this notion, the most pronounced and significant associations between brain structure and BMI in adolescents were not observed in cortical regions but rather in the amygdala. Yet, it is important to acknowledge that BMI was associated with lower cortical thickness in adolescent participants but might have failed to reach significance due to limited sample size in this analysis (see Supplementary Results for power analysis). Regarding a potential explanation for early detectable amygdala volume increase in obesity, it appears important to consider the relevance of the amygdala in increased cue triggered learning [48] and Pavlovian conditioning to hedonic food that represents a key mechanism in future weight gain [49]. Importantly, the apparent discrepancy in obesity between early detectable subcortical volume increase on the one hand, and lower thickness with increasing age on the other, raises questions regarding potentially differing pathways behind the development of brain structural alterations in obesity that should be addressed by future experimental research.

The aforementioned notion of differing pathways underlying brain structural abnormalities in obesity appears to be further supplemented by the imaging genetic findings of the present study. The regionally pronounced effect of polygenic risk for obesity on lateral occipital surface area was unexpected. Prior studies have implicated the lateral occipital cortex in obesity [14, 50, 51], yet BMI was negatively correlated with occipital surface area but failed to reach significance in the present study (*p*(FDR) = 0.089). Similarly, since no significant mediation effect of lateral occipital surface area was observed in the association between polygenic risk and BMI, the functional relevance of this finding remains uncertain. In contrast, it appears important to note that in the present study left lateral orbitofrontal thickness mediated the association between polygenic risk and BMI which appears to replicate similar findings in a previous VBM study [24]. The notion that the influence of genetic risk for obesity on body weight might be mediated through changes in brain physiology is further supported by reports on high expression of obesity-related genes in the central-nervous system [23, 52]. Previous reports on associations between food addiction and OFC thickness [51] appear to further corroborate a model in which prefrontal brain regions might influence eating behavior and subsequent weight gain. However, results from these analyses have to be interpreted with great caution and do not allow for causal interpretations due to the cross-sectional design of the present study. Future studies are needed to directly test this hypothesis in experimental, longitudinal designs before form conclusions can be drawn.

Furthermore, it appears important to note that a large proportion of variance in obesity-related brain structural abnormalities could not be explained by genetic influence in the present study. It thus appears crucial to consider that increased body weight itself could contribute to brain structural abnormalities through mechanisms such as obesity-related low-grade inflammation, kynurenine pathway activation, or neuroendocrine dysregulation [17, 53–55]. Another previously hypothesized link between obesity and brain structural abnormalities implies brain energy consumption during childhood and subsequent development of obesity [56], and hence points to educational interventions during childhood as a preventive measure against obesity.

Finally, the rather unexpected finding of a moderating role of sex on BMI-related cortical thickness decrease should be acknowledged. In the present study, male subjects exhibited significantly lower BMI-related cortical thickness compared with female participants. The potential relevance of this finding is highlighted by a previous PET study reporting significantly lower metabolic brain age in female compared with male subjects [57] and should be targeted by future research.

The presented analysis has strengths and limitations. Major strengths of the present work are the large sample size including healthy participants and depressive patients and the inclusion of imaging and genetic data. In addition, the combination of univariate group-level and multivariate machine learning techniques further highlighted the relevance of the observed associations on single-subject level. The most severe limitation of our study is the cross-sectional design that prevents us from drawing causal conclusions. Our interpretations with regard to the onset and mechanisms behind brain structural abnormalities in obesity need clarification from longitudinal research before firm conclusions can be drawn. It furthermore appears important to note that BMI was not accounted for in previous studies on psychiatric disorders from the ENIGMA consortium. Considering the known association between affective disorders and obesity, the observed similarities between obesity and affective disorders observed here might thus partially be explained by higher BMI in the patient samples of such studies. Moreover, we acknowledge that our study sample is not independent from patient and control samples of previous ENIGMA studies and therefore overlap in participants might contribute to the similarities in brain structural findings between obesity and affective disorders.

To conclude, the present findings demonstrate similar associations between obesity and brain structural abnormalities in healthy participants and depressive patients. Cortical thickness reductions in the temporal and frontal cortex were identified as the most consistent and pronounced structural neuroimaging findings in adult obesity in the present study. Future voxel-wise neuroimaging studies capable of providing higher resolution should aim to further delineate the precise regional distribution of obesity-related gray matter decrease.

Results of the present study suggest that the distribution and extent of obesity-related brain structural abnormalities is comparable with findings in neuropsychiatric disorders. This notion critically underlines the similarities in patterns of impaired brain structural integrity between obesity and common neuropsychiatric disorders and points to the relevance of altered brain physiology in obesity that still appears to be drastically underestimated in current research. While neuropsychiatric disorders such as major depression are widely considered to be disorders of the brain, obesity is primarily considered as a cardiovascular risk factor in research and clinical practice. As the brain structural correlates of obesity exceed those of common neuropsychiatric disorders such as MDD—in terms of affected regions and effect size per region—the findings presented here should urge clinicians and scientists to devote increased attention to neurobiological characteristics of obesity. The association of obesity with altered brain structural integrity in the present study indicates the need for a paradigm shift in obesity prevention and research.

## Supplementary information

Supplementary Information
