# Correction: Brain structural abnormalities in obesity: relation to age, genetic risk, and common psychiatric disorders

**DOI:** 10.1038/s41380-021-01191-1

**Published:** 2021-06-22

**Authors:** Nils Opel, Anbupalam Thalamuthu, Yuri Milaneschi, Dominik Grotegerd, Claas Flint, Ramona Leenings, Janik Goltermann, Maike Richter, Tim Hahn, Georg Woditsch, Klaus Berger, Marco Hermesdorf, Andrew McIntosh, Heather C. Whalley, Mathew A. Harris, Frank P. MacMaster, Henrik Walter, Ilya M. Veer, Thomas Frodl, Angela Carballedo, Axel Krug, Igor Nenadic, Tilo Kircher, Andre Aleman, Nynke A. Groenewold, Dan J. Stein, Jair C. Soares, Giovana B. Zunta-Soares, Benson Mwangi, Mon-Ju Wu, Martin Walter, Meng Li, Ben J. Harrison, Christopher G. Davey, Kathryn R. Cullen, Bonnie Klimes-Dougan, Bryon A. Mueller, Philipp G. Sämann, Brenda Penninx, Laura Nawijn, Dick J. Veltman, Lyubomir Aftanas, Ivan V. Brak, Elena A. Filimonova, Evgeniy A. Osipov, Liesbeth Reneman, Anouk Schrantee, Hans J. Grabe, Sandra Van der Auwera, Katharina Wittfeld, Norbert Hosten, Henry Völzke, Kang Sim, Ian H. Gotlib, Matthew D. Sacchet, Jim Lagopoulos, Sean N. Hatton, Ian Hickie, Elena Pozzi, Paul M. Thompson, Neda Jahanshad, Lianne Schmaal, Bernhard T. Baune, Udo Dannlowski

**Affiliations:** 1 Department of Psychiatry, University of Münster, Münster, Germany; 2 Interdisciplinary Centre for Clinical Research (IZKF), University of Münster, Münster, Germany; 3 Centre for Healthy Brain Ageing, School of Psychiatry, University of New South Wales, Sydney, NSW Australia; 4 Neuroscience Research Australia, Randwick, NSW Australia; 5 Department of Psychiatry, Amsterdam UMC/Vrije Universiteit, Amsterdam, Netherlands; 6 Faculty of Mathematics and Computer Science, University of Münster, Münster, Germany; 7 IT Department, University of Muenster, Münster, Germany; 8 Institute of Epidemiology and Social Medicine, University of Münster, Münster, Germany; 9 Division of Psychiatry, University of Edinburgh, Edinburgh, UK; 10 Psychiatry and Paediatrics, University of Calgary, Calgary, AB Canada; 11 Addictions and Mental Health Strategic Clinical Network Calgary, Calgary, AB Canada; 12 Division of Mind and Brain Research, Department of Psychiatry and Psychotherapy CCM, Charité - Universitätsmedizin Berlin, corporate member of Freie Universität Berlin, Humboldt-Universität zu Berlin, and Berlin Institute of Health, Berlin, Germany; 13 Department of Psychiatry, Trinity College Dublin, Dublin, Ireland; 14 Department of Psychiatry and Psychotherapy, Otto von Guericke University Magdeburg, Magdeburg, Germany; 15 Department of Psychiatry and Psychotherapy, University of Marburg, Marburg, Germany; 16 Department of Biomedical Sciences of Cells and Systems, University of Groningen, University Medical Center Groningen, Groningen, The Netherlands; 17 SA MRC Unit on Risk & Resilience, Department of Psychiatry and Neuroscience Institute, University of Cape Town, Cape Town, South Africa; 18 UT Center of Excellence on Mood Disorders, Department of Psychiatry and Behavioral Sciences, University of Texas Health Science Center at Houston, Houston, TX USA; 19 Department of Psychiatry, University of Texas Health Science Center at Houston, Houston, TX USA; 20 Department of Psychiatry and Psychotherapy, Jena University Hospital, Jena, Germany; 21 Melbourne Neuropsychiatry Centre, Department of Psychiatry, The University of Melbourne & Melbourne Health, Parkville, VIC Australia; 22 Orygen, The National Centre of Excellence in Youth Mental Health, Parkville, VIC Australia; 23 Centre for Youth Mental Health, The University of Melbourne, Parkville, VIC Australia; 24 Department of Psychiatry and Behavioral Sciences, School of Medicine, University of Minnesota, Minneapolis, MN USA; 25 Department of Psychology, University of Minnesota, Minneapolis, MN USA; 26 Max Planck Institute of Psychiatry, Munich, Germany; 27 FSSBI “Scientific Research Institute of Physiology & Basic Medicine”, Laboratory of Affective, Cognitive & Translational Neuroscience, Novosibirsk, Russia; 28 Novosibirsk State University, Laboratory of Experimental & Translational Neuroscience, Novosibirsk, Russia; 29 Department of Radiology and Nuclear Medicine, Academic Medical Center, University of Amsterdam, Amsterdam, Netherlands; 30 Department of Psychiatry and Psychotherapy, University Medicine Greifswald, Greifswald, Germany; 31 German Center for Neurodegenerative Diseases (DZNE), Greifswald/Rostock, site Greifswald, Greifswald, Germany; 32 Institute of Diagnostic Radiology and Neuroradiology, University Medicine Greifswald, Greifswald, Germany; 33 Institute for Community Medicine, University Medicine Greifswald, Greifswald, Germany; 34 German Center for Cardiovascular Research (DZHK), Partner Site Greifswald, Greifswald, Germany; 35 West Region, Institute of Mental Health, Singapore, Singapore; 36 Yoo Loo Lin School of Medicine, National University of Singapore, Singapore, Singapore; 37 Department of Psychology, Stanford University, Stanford, CA USA; 38 Center for Depression, Anxiety, and Stress Research, McLean Hospital, Harvard Medical School, Belmont, MA USA; 39 Sunshine Coast Mind and Neuroscience Thompson Institute, University of the Sunshine Coast, Sippy Downs, QLD Australia; 40 Brain and Mind Centre, University of Sydney, Camperdown, NSW Australia; 41 Melbourne Neuropsychiatry Centre, Department of Psychiatry, The University of Melbourne, Parkville, VIC Australia; 42 Mark & Mary Stevens Neuroimaging & Informatics Institute, Keck School of Medicine, University of Southern California, Marina del Rey, CA USA; 43 Department of Psychiatry, Melbourne Medical School, The University of Melbourne, Melbourne, VIC Australia; 44 The Florey Institute of Neuroscience and Mental Health, The University of Melbourne, Parkville, VIC Australia

**Keywords:** Diagnostic markers, Depression

Correction to: *Mol Psychiatry* 10.1038/s41380-020-0774-9

The article “Brain structural abnormalities in obesity: relation to age, genetic risk, and common psychiatric disorders”, written by Nils Opel, Anbupalam Thalamuthu, Yuri Milaneschi, Dominik Grotegerd, Claas Flint, Ramona Leenings, Janik Goltermann, Maike Richter, Tim Hahn, Georg Woditsch, Klaus Berger, Marco Hermesdorf, Andrew McIntosh, Heather C. Whalley, Mathew A. Harris, Frank P. MacMaster, Henrik Walter, Ilya M. Veer, Thomas Frodl, Angela Carballedo, Axel Krug, Igor Nenadic, Tilo Kircher, Andre Aleman, Nynke A. Groenewold, Dan J. Stein, Jair C. Soares, Giovana B. Zunta-Soares, Benson Mwangi, Mon-Ju Wu, Martin Walter, Meng Li, Ben J. Harrison, Christopher G. Davey, Kathryn R. Cullen, Bonnie Klimes-Dougan, Bryon A. Mueller, Philipp G. Sämann, Brenda Penninx, Laura Nawijn, Dick J. Veltman, Lyubomir Aftanas, Ivan V. Brak, Elena A. Filimonova, Evgeniy A. Osipov, Liesbeth Reneman, Anouk Schrantee, Hans J. Grabe, Sandra Van der Auwera, Katharina Wittfeld, Norbert Hosten, Henry Völzke, Kang Sim, Ian H. Gotlib, Matthew D. Sacchet, Jim Lagopoulos, Sean N. Hatton, Ian Hickie, Elena Pozzi, Paul M. Thompson, Neda Jahanshad, Lianne Schmaal, Bernhard T. Baune & Udo Dannlowski, was originally published electronically on the publisher’s internet portal on 28 May 2020 without open access. With the author(s)’ decision to opt for Open Choice the copyright of the article changed on 4 June 2021 to © The Author(s) 2021 and the article is forthwith distributed under a Creative Commons Attribution 4.0 International License, which permits use, sharing, adaptation, distribution and reproduction in any medium or format, as long as you give appropriate credit to the original author(s) and the source, provide a link to the Creative Commons licence, and indicate if changes were made. The images or other third party material in this article are included in the article’s Creative Commons licence, unless indicated otherwise in a credit line to the material. If material is not included in the article’s Creative Commons licence and your intended use is not permitted by statutory regulation or exceeds the permitted use, you will need to obtain permission directly from the copyright holder. To view a copy of this licence, visit http://creativecommons.org/licenses/by/4.0. Open Access funding enabled and organized by Projekt DEAL.

