# Supplementary material for: Brain structural abnormalities in obesity: relation to age, genetic risk, and common psychiatric disorders: Evidence through univariate and multivariate mega-analysis including 6420 participants from the ENIGMA MDD working group
Source: Mol Psychiatry. 2020 May 28;26(9):4839–52. doi: 10.1038/s41380-020-0774-9 (PMC8589644; doi:10.1038/s41380-020-0774-9)
Supplement: Supplementary file 1 — Supplementary Information [file 41380_2020_774_MOESM1_ESM.docx]

**Supplementary information**

**Supplementary Methods**

**Supplementary Results**

**Supplementary Figure 1.** Distribution of obese participants as a function of participants age

**Supplementary Figure 2.** Histogram of body mass index in the HC sample

**Supplementary Figure 3.** Histogram of body mass index in the MDD sample

**Supplementary Figure 4.** Correlation of effect sizes from Model A and Model B

**Supplementary Figure 5.** Visualisation of effect sizes from main analyses and confirmatory analyses

**Supplementary Figure 6.** Visualisation of effect sizes for obesity and neuropsychiatric disorders

**Supplementary Figure 7.** Visualisation of effect sizes for obesity in the MDD and HC subsample

**Supplementary Table 1.** Sociodemographic details of all cohorts

**Supplementary Table 2.** Exclusion criteria

**Supplementary Table 3.** Structural MRI details

**Supplementary Table 4.** Genotyping details

**Supplementary Table 5.** Full mega-analysis results for BMI

**Supplementary Table 6.** Full mega-analysis results for Obesity vs. Normal weight subjects

**Supplementary Table 7.** Full mega-analysis results for BMI controlling for antidepressant medication

**Supplementary Table 8.** Full mega-analysis results for BMI on cortical thickness adjusted for mean thickness

**Supplementary Table 9.** Full mega-analysis results for quadratic effects of BMI

**Supplementary Table 10.** Full mega-analysis results for BMI corrected for quadratic effects of age

**Supplementary Table 11.** Full mega-analysis results for ANOVA model including weight group (normal-weight. overweight. obesity) as categorical predictor

**Supplementary Table 12.** Full mega-analysis results for BMI corrected for head movement

**Supplementary Table 13.** Full mega-analysis results for the interaction of MDD x BMI

**Supplementary Table 14.** Full mega-analysis results for Obesity vs. Normal weight subjects in the HC subsample

**Supplementary Table 15.** Full mega-analysis results for Obesity vs. Normal weight subjects in the MDD subsample

**Supplementary Table 16.** Full mega-analysis results for the interaction Sex x BMI

**Supplementary Table 17.** Full mega-analysis results for the interaction Age x Obesity

**Supplementary Table 18.** Full mega-analysis results for BMI in adolescent participants

**Supplementary Table 19.** Results of regression analyses of polygenic scores on BMI

**Supplementary Table 20.** Results of regression analyses of polygenic scores on BMI controlling for age. sex. MDD diagnosis

**Supplementary Table 21.** Full mega-analysis results for PRS (p1.0) on brain structure

**Supplementary Table 22.** Full mega-analysis results for PRS (p0.2) on brain structure

**Supplementary Methods**

*Genetic methods*

The quality controlled genotype data was imputed separately in each cohort using the Haplotype Reference Consortium (HRC) reference panel (v3.20101123) (<http://www.haplotype-reference-consortium.org/home)>.^1^ Publicly available data from the GIANT BMI GWAS by Locke at al^2^ were used as the discovery sample. As genetic data from the NESDA, SHIP and SHIP-trend cohorts were previously included in the GIANT BMI-GWAS, we reran the BMI-GWA^2^ by excluding data from NESDA, SHIP and SHIP-trend to avoid any overlap between discovery and replication sample. The newly calculated BMI GWAS results were used as base GWAS data. To harmonize polygenic score calculation across sites, the same set of N~ 133.299 independent SNPs (obtained using the clumping option with r^2^ threshold of 0.25 and physical distance of 250KB) was used for PRS calculation across all cohorts.

*Multivariate pattern recognition analyses*

In order to individually classify participants as either obese or normal-weight all available 157 FreeSurfer derived imaging measures were used as features to train and test a machine learning pipeline with nested cross-validation and hyperparameter optimization using the PHOTON framework (https://photon-ai.com). The pipeline consisted of several pre-processing steps. Missing values were imputed by their respective mean. None of the features entered into the pipeline had more than 11.1 % missing data. Features were scaled to have zero mean and unit variance. To reduce the dimensionality of the feature space and discard noisy information, principal component analysis was applied, optimizing the number of principal components in a range between 5 and 150. We used random undersampling to derive balanced training samples. A support vector machine (SVM) was trained using an rbf-kernel, the kernel coefficient parameter gamma being set to scale, and optimizing the regularization parameter C in a range between 0.5 and 2. To assure a valid estimation of the model‘s generalizability, nested cross-validation (CV) was applied in a framework of five outer folds, respectively repeating the hyperparameter optimization process and five inner folds for evaluating a concrete hyperparameter configuration‘s performance. Hyperparameter optimization was performed based on bayesian optimization as implemented in the scikit-optimize toolbox using the default parameters and 20 iterations. Model performance was then assessed based on balanced accuracy, sensitivity, specificity, F1 score and the area under the receiver operator characteristic curve (ROC-AUC) metric. Best configurations optimizing the f1 score are {n=118 PCA components; C=1.817} for the standard analysis. {n=140 PCA components; C=1.869} for the balanced covariates analysis and {n=134 PCA components; C=1.095} for the leave-one-site out analysis. Finally, permutation tests were conducted to estimate the likelihood of obtaining classification performance by chance (at an α of 0.05 for each balanced accuracy), by using 1000 random permutations of the labels and applying the nested-cross-validation training and test procedure including hyperparameter optimization CV to each permutation. The complete analytical script can be found here: https://github.com/NilsOpel/ENIGMA-MDD-Obesity.git.

**Supplementary Results**

*Distribution of obesity and BMI in the study sample*

Mean BMI was significantly higher in MDD compared to HC participants (MDD: mean BMI=26.28, SD=5.50; HC: mean BMI=25.71, SD=4.47) as assessed through independent sample t-test (t_(1, 6418)_=4.65, p<0.001) and confirmed through linear models controlling for age, sex and site (F_(1, 6389)_=109.28, p<0.001). Furthermore, in MDD patients the number of depressive episodes as well as the extent of self-reported symptoms by means of the Beck Depression Inventory^3^ were positively associated with higher BMI (number of episodes: B=0.074, t_(1,1664)_=3.54, p<0.001; BDI: B=0.049, t_(1,1535)_=4.18, p<0.001).

*Power analysis*

We performed post hoc power analyses to estimate the required effect size for an observed association given the sample size, alpha-threshold and power in this study. All power estimates were obtained using the g-power package (http://www.gpower.hhu.de). In the full sample (n=6.420) we were able to detect cortical differences as small as Cohen’s *d*=0.090 (corresponding to an r=0.015) at a Bonferroni corrected significance threshold for 157 independent tests (p=0.05/157) and 80% power estimated through a two-tailed bivariate correlational design. Group differences between n=1223 obese subjects and n=2917 normal weight subjects could be detected up to a Cohen’s *d*=0.151 at a Bonferroni corrected significance threshold for 157 independent tests (p=0.05/157) and 80% power estimated through a two-tailed t-test.

With 520 adolescent subjects, we were able to detect brain structural differences as small as Cohen’s *d*=0.303 (corresponding to an r=0.015) at a Bonferroni significance threshold for 157 independent tests (p=0.05/157) and 80% power.

*Analyses stratified by diagnostic status*

To assess the distribution of obesity related brain structural abnormalities separately for the HC and MDD subsample of our study, we repeated analysis step 1 (group comparisons between normal-weight and obesity) separately in the HC and MDD subsample (see also **Supplementary Tables 12** and **13).**

Regional effect size estimates of associations between obesity and brain structure from analyses in both subsamples showed nearly perfect correlations with initial effect sizes obtained in the entire sample (HC: r= .922; MDD: r= .964). Similarly, regional effect size estimates of associations between obesity and brain structure in MDD and HC were highly correlated (r= .789). Consistently, no FDR corrected significant BMI x Diagnosis interaction effects could be detected in our main analyses (see also **Supplementary Table 13**).

We furthermore aimed to investigate if obesity related brain structural abnormalities in the HC and MDD subsample would show a similar degree of overlap with findings of brain structural abnormalities in MDD and BD. To this end, analysis step 2 was repeated by including regional effect size estimates for associations between obesity and brain structure derived from the HC and MDD subsample. Effect size estimates for obesity from the HC subsample correlated with both effect size estimates for MDD (r= .390) and for BD (r= .455). Similarly, effect size estimates for obesity from the MDD subsample correlated with effect size estimates for MDD (r= .436) and for BD (r= .491).

In sum, effect size estimates for brain structural abnormalities in obesity showed a similar distribution in both the HC and MDD subsample and furthermore showed a comparable degree of overlap with brain structural abnormalities in major depression and bipolar disorder.

*Bias due to* *head motion*

Since higher BMI has previously been associated with increased head motion^7^, we decided to carry out additional analysis correcting for head movement as potential source of additional bias^8^. Since a) movement parameters were not shared between the participating sites, and b) since motion parameters were not maintained during preprocessing with the usual FreeSurfer segmentation routine, we were obliged to conduct these additional analyses in a subsample of participants for which head motion parameters were available. This was the case for the Munster Cohort and the FOR cohort for which head motion parameters could be derived from preprocessing steps of an fMRI task that was conducted directly after the acquisition of the T1 images in both cohorts. While the inclusion of movement parameters that were not acquired during the acquisition of the T1 image itself might not be optimal, we argue that the assumed general tendency of increased head movement covarying with increased BMI should be present irrespective of the applied MRI sequence. Thus. we first repeated the regression analysis of BMI values on brain structure in the subsample of participants (n=1423) for which head movement parameters were available. As could be expected, this analysis yielded highly consistent results as compared to the original regression analysis of BMI values in the entire sample quantified by a nearly perfect correlation of effect size estimates between both analyses (r= .865). Next. we repeated the regression analysis in the subsample by including individual head movement estimates for the x,y and z axis in mm as additional nuisance covariates in the model (see **Supplementary Table 12** below for details). Again, overall consistent results emerged: Effect size estimates of the regression analysis with and without additional inclusion of movement parameters showed a nearly perfect correlation (r= .981) while the absolute extent of effect sizes was attenuated in analyses controlling for head motion with descriptively lower effect sizes for cortical thickness (t= -1.96) and larger effect sizes for cortical surface (t= 1.19) and subcortical (t= 2.00) regions (t-value assessed through paired sample t-tests, see also **Supplementary Figure 5**).

*Laterality effects*

We aimed to investigate the degree of overlap in effect sizes between left and right hemisphere in all n= 156 ROIs by conducting correlation analyses and paired samples t-tests. We report r-values and t-statistics from these additional analyses and refrain from reporting degrees of freedom and p-values, since it is unclear if independence of observations can be assumed due to the correlation between effect sizes from the ROIs. The additional analysis indicated an overall large degree of overlap between left and right hemispherical effect sizes across all 156 investigated ROIs with an overall correlation of r= .946 between left and right hemispherical effect sizes (t-value for potential differences between left and right hemisphere: t= -.974). Similarly, additional analyses stratified by imaging data entity (subcortical volume, cortical thickness, cortical surface area) indicated high overlap in effect sizes between left and right hemispheres in cortical surface area effect sizes (r= .881, t= -.234) subcortical volume effect sizes (r= .803, t= -.235) and cortical thickness effect sizes (r= .881, t= -1.125). We thus conclude that our results point to an overall large overlap in effect sizes between left and right hemisphere, but acknowledge descriptively larger effects for the association between obesity and lower cortical thickness in the left compared to right cortical hemisphere.

*Additional analyses of effect sizes for obesity and neuropsychiatric disorders*

First, we performed additional sensitivity analyses to rule out that our comparisons of effect sizes between obesity and affective disorders were biased by systematic differences in measuring cortical thickness across the brain (e.g. systematic differences in the Signal-to-noise ratio between brain regions). If such systematic bias was the key driver of the observed similarities in cortical thickness findings between obesity and affective disorders, it should similarly be present in comparison of obesity and any other neuropsychiatric disorder (that would similarly be affected by e.g. systematic differences in the Signal-to-noise ratio between brain regions). Vice versa, such bias is unlikely to underlie the observed correlation between obesity and affective disorders if the correlation between obesity and another neuropsychiatric disorder would be of much weaker size. To test this hypothesis, we carried out an additional analysis by assessing the correlation between effect sizes for obesity related cortical thickness alterations and effect sizes for cortical thickness from the ENIGMA mega-analysis on autism spectrum disorder (ASD)^9^. We observed a much weaker correlation between effect sizes for obesity and ASD (r= .149). as compared to the previously reported pronounced correlations between obesity and MDD (r=0.452) and BD (r=0.513). In light of these additional findings. we believe that SNR differences are unlikely to represent a source of substantial bias in the presented analyses.

Second, we aimed to complement the conducted correlational analyses which assesses the relative similarity between effect sizes for obesity and neuropsychiatric disorders, by further comparison of the absolute extent of effect sizes. To this end, paired t-tests were conducted to investigate differences in effect sizes for cortical thickness across all ROIs between obesity and MDD, BD and ASD. Again, we refrain from reporting degrees of freedom and p-values, since it is unclear if independence of observations can be assumed due to the correlation between effect sizes from the ROIs. Results from the paired t-test yielded overall larger effect sizes for cortical thickness in obesity compared to MDD (t= -11.30) and ASD (t= -9.79) but overall lower effect sizes in obesity compared to BD (t= 5.06). In addition, we plotted effect sizes for cortical thickness for obesity, MDD, BD and ASD to allow easier visualization and comparison of overall as well as local similarities and differences in effect sizes (**Supplementary Figure 6**).

*Predictive features*

Since the non-linear rbf kernel applied in our machine learning pipeline does not allow for the computation of feature weights, we conducted additional machine learning analyses in which we attempted to test the predictive relevance of brain regions associated with obesity in univariate analyses. To this end, we tested whether a subset of features selected based on their univariate effect size would outperform randomly drawn subsets of features. We therefore first reran the precisely similar machine learning pipeline but restricted the classifier input (feature space) to the top ten ROIs showing strongest associations with obesity in the univariate regression analyses. As could be expected, the feature space reduction led to a less optimal classifier performance with a BAC of 57.2%. However, the classifier trained on the top ten ROIs outperformed classifiers trained on randomly drawn samples of ten ROIs (BAC= 50.8%, 10 iterations). We thus conclude that these additional results demonstrate a) that the optimal prediction relies on a multivariate pattern fed by a variety of regional information and b) that the observed univariate associations outweigh randomly selected ROIs in terms of their relevance for the multivariate pattern.

*Mediation analysis*

We conducted additional mediation analyses to further investigate the association between polygenic risk, brain structure and BMI. This was done using the SPSS macro PROCESS which has previously been applied by us and others to assess mediation effects in the context of neuroimaging research.^4.5^ PROCESS estimates direct and indirect effects between a defined set of variables by applying an ordinary least squares path analytic framework. Inference of indirect (mediated) effects is assessed through bootstrap confidence intervals. Significance of an indirect effects is assumed if the 95% confidence interval (95%-CI) does not include zero. The number of bootstrap samples was set to n=5000. Unstandardized regression coefficients (coeff) and standard errors (SE) are presented for each effect.

Our hypothesis for the additional mediation analysis in the present study were based on the following relevant background information: (1) temporal precedence can be assumed for the PRS variable, since of course, genetic predisposition is supposed to precede phenotypes such as BMI or brain structure; (2) in a previous study by our group we observed a significant mediation effect of polygenic risk for obesity on BMI through orbitofrontal gray matter volume^4^ (3) the data from this previous report^4^ came from two cohorts (Munster Cohort and BiDirect) that are also part of the present mega-analysis. Based on this information, we aimed to test the hypothesis that brain structure mediates the influence of PRS on BMI.

For the additional mediation analyses in the present study, we decided to test for mediation effects a) of the brain structural variable showing the strongest association with PRS and b) of the brain structural variable that had previously been shown to mediate the effect of PRS on BMI in an attempt to test for replication of this previous finding.

For a) as reported in the main result section. the left lateral occipital surface area showed the strongest association with PRS and represented the only association to reach FDR corrected significance (B=-45.92, StdE=12.56, t=-3.66, p=0.00026, p(FDR)=0.041, n=3526). However. it should be taken into account that no significant association between left lateral occipital surface area and BMI was observed in the present study. Thus, the conventional requirement for mediation analysis is not met in the present case.

For b) since the present study did not employ voxel-based morphometry and hence no local gray matter volume data was available in the present mega-analysis, we selected OFC thickness as the best proxy for replication of our previous findings considering the known high correlation between OFC gray matter volume and thickness.^6^ In the present study, the left lateral orbitofrontal cortex showed the most pronounced association with PRS out of all OFC ROIs and was therefore selected as candidate for the mediation analysis (B=-0.0098, StdE=0.0036, t= -2.76. p=0.0058, p(FDR)= 0.23, n=3812).

To account for the potential influence of further variables that are likely to bias the relationship between PRS, brain structure and BMI, we accounted for the standard nuisance regressors (age, sex, diagnosis, site) in all mediation models.

1. We did not observe a mediation effect of left lateral occipital surface area in the association between polygenic risk score (p= 1.0) and BMI (indirect effect= .0218, SE= .0182, 95% CI = -.0145 to .0546). A similar result emerged for the model including the PRS (p=0.2) (indirect effect= .0209, SE= .0168, 95% CI = -.0142 to .0533).
2. A significant mediation of PRS (p=1.0) on BMI through left lateral orbitofrontal thickness emerged (indirect effect= -.052, SE= .019, 95% CI = -.091 to -.014). A similar result emerged when applying the PRS (p= 0.2) (indirect effect= -.041, SE= .017, 95% CI = -.074 to -.007)

**Supplementary References**

1. Consortium the HR. McCarthy S. Das S. et al. A reference panel of 64.976 haplotypes for genotype imputation. *Nat Genet*. 2016;48(10):1279-1283. doi:10.1038/ng.3643

2. Locke AE. Kahali B. Berndt SI. et al. Genetic studies of body mass index yield new insights for obesity biology. *Nature*. 2015;518(7538):197-206. doi:10.1038/nature14177

3. Beck AT. Steer RA. *Beck Depression Inventory: Manual*. San Antonio: The Psychological Corporation. Harcourt Brace Jovanovich.; 1987.

4. Opel N. Redlich R. Kaehler C. et al. Prefrontal gray matter volume mediates genetic risks for obesity. *Mol Psychiatry*. 2017;22(5):703-710. doi:10.1038/mp.2017.51

5. Mackey S. Chaarani B. Kan K-J. et al. Brain Regions Related to Impulsivity Mediate the Effects of Early Adversity on Antisocial Behavior. *Biol Psychiatry*. January 2016. doi:10.1016/j.biopsych.2015.12.027

6. Storsve AB. Fjell AM. Tamnes CK. et al. Differential Longitudinal Changes in Cortical Thickness. Surface Area and Volume across the Adult Life Span: Regions of Accelerating and Decelerating Change. *J Neurosci*. 2014;34(25):8488-8498. doi:10.1523/JNEUROSCI.0391-14.2014

7. Hodgson K. Poldrack RA. Curran JE. et al. Shared Genetic Factors Influence Head Motion During MRI and Body Mass Index. *Cereb Cortex*. 2016;27(12):5539-5546. doi:10.1093/cercor/bhw321

8. Reuter M. Tisdall MD. Qureshi A. Buckner RL. van der Kouwe AJW. Fischl B. Head motion during MRI acquisition reduces gray matter volume and thickness estimates. *Neuroimage*. 2015;107:107-115. doi:10.1016/j.neuroimage.2014.12.006

9. van Rooij D. Anagnostou E. Arango C. et al. Cortical and Subcortical Brain Morphometry Differences Between Patients With Autism Spectrum Disorder and Healthy Individuals Across the Lifespan: Results From the ENIGMA ASD Working Group. *Am J Psychiatry*. 2018;175(4):359-369. doi:10.1176/appi.ajp.2017.17010100

**Supplementary Figure 1**

Histogram displaying the distribution of obese participants as a function of participants age.


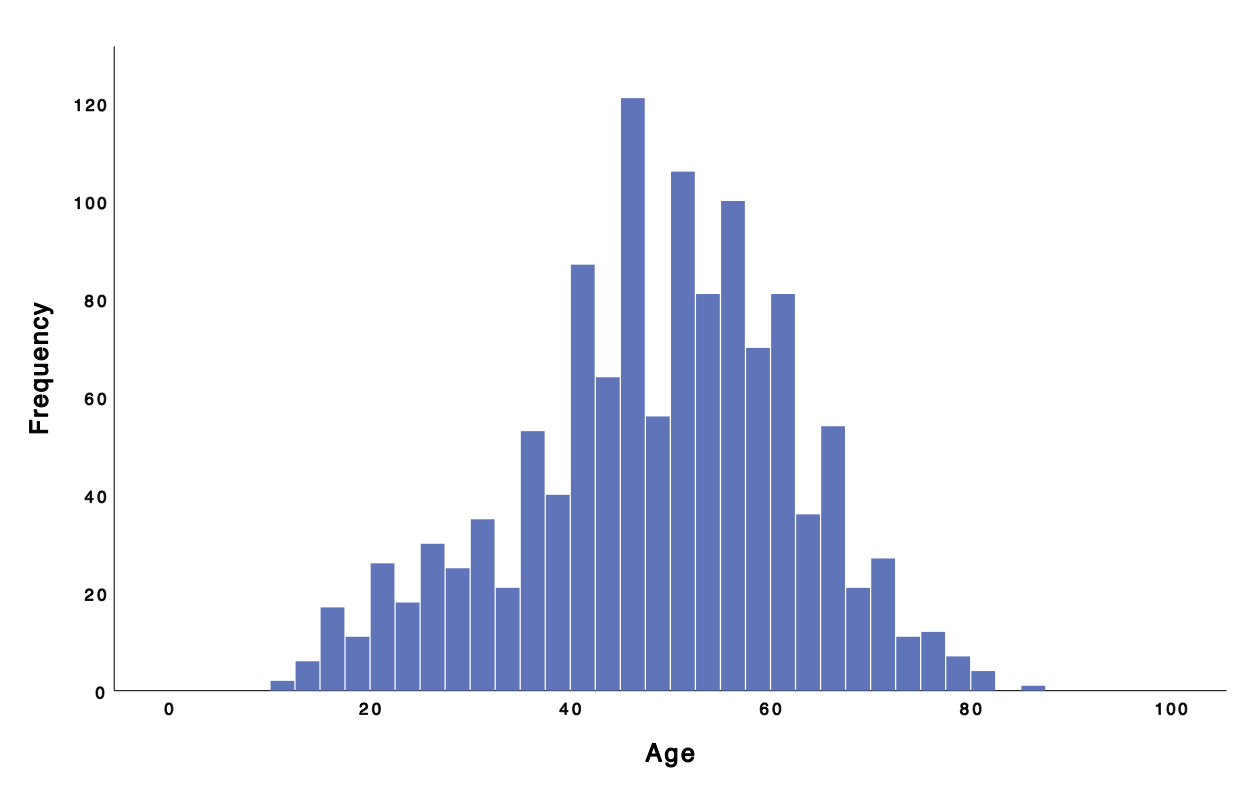


**Supplementary Figure 2**

Histogram displaying the distribution of body weight as operationalized by body mass index in the HC group

**Supplementary Figure 3**

Histogram displaying the distribution of body weight as operationalized by body mass index in the MDD group

**Supplementary Figure 4**

Plot displaying the correlation between the effect sizes (Cohen´s d values) resulting from Model A (group comparison between obesity vs. normal weight) and Model B (regression analysis using BMI as continuous predictor) (r= .978)

**Supplementary Figure 5**

Visualisation of effect sizes (Cohen´s d) from main analyses of associations between BMI and cortical thickness (d_BMI) and confirmatory analyses of quadratic effects of BMI (d_quadrBMI), analyses controlling for quadratic effects of Age (d_BMI_quadrAgeCorr) and analyses controlling for head movement (d_BMI_MovCorr).

**Supplementary Figure 6**

Visualisation of effect sizes (Cohen´s d) for associations between cortical thickness and obesity and effect sizes for associations between cortical thickness and major depression (MDD), bipolar disorder (BD) and autism spectrum disorder (ASD) from previous ENIGMA studies.

**Supplementary Figure 7**

Visualisation of effect sizes (Cohen´s d) from main analyses of associations between BMI and cortical thickness (d_BMI), obesity and cortical thickness (d_BMIGr) and confirmatory analyses of associations between obesity and cortical thickness stratified for diagnostic group in the MDD subsample (d_BMIGr_MDD) and the HC subsample (d_BMIGr_HC).

| **Study** | **Sample** | **Total N** | **Age (Mean)** | **Age (SD)** | **% Female** | **% MDD cases** | **BMI (Mean)** | **BMI (SD)** | **N obese cases** | **N normal weight cases** |
| --- | --- | --- | --- | --- | --- | --- | --- | --- | --- | --- |
|  |  |  |  |  |  |  |  |  |  |  |
| 1 | NESDA. Scanner/Site 1 | 72 | 38.00 | 9.57 | 63.89 | 63.89 | 24.24 | 3.87 | 4 | 45 |
| 2 | NESDA. Scanner/Site 2 | 88 | 38.52 | 9.91 | 69.32 | 69.32 | 25.74 | 4.69 | 19 | 40 |
| 3 | NESDA. Scanner/Site 3 | 59 | 37.63 | 11.94 | 64.41 | 79.66 | 25.45 | 4.48 | 9 | 32 |
| 4 | Groningen (DIP) | 43 | 42.33 | 13.93 | 72.09 | 46.51 | 24.86 | 4.28 | 5 | 25 |
| 5 | Dublin. Scanner/Site 1 | 104 | 39.16 | 12.14 | 59.62 | 50.00 | 24.44 | 3.78 | 8 | 64 |
| 6 | Dublin. Scanner/Site 2 | 32 | 35.69 | 9.55 | 43.75 | 46.88 | 23.81 | 4.57 | 3 | 18 |
| 7 | CODE | 92 | 39.52 | 11.94 | 68.48 | 66.30 | 24.85 | 4.47 | 9 | 50 |
| 8 | Singapore | 40 | 38.98 | 6.94 | 47.50 | 57.50 | 24.19 | 4.65 | 4 | 18 |
| 9 | SHIP | 586 | 55.06 | 12.57 | 50.51 | 23.55 | 27.63 | 4.48 | 167 | 179 |
| 10 | SHIP-trend | 1250 | 50.30 | 13.80 | 48.96 | 24.96 | 27.47 | 4.34 | 331 | 380 |
| 11 | Sydney | 135 | 21.32 | 5.83 | 64.44 | 76.30 | 23.05 | 3.97 | 9 | 85 |
| 12 | Calgary | 84 | 16.95 | 4.69 | 53.57 | 46.43 | 22.55 | 5.26 | 10 | 47 |
| 13 | Magdeburg | 33 | 37.18 | 13.75 | 54.55 | 51.52 | 24.71 | 5.13 | 5 | 21 |
| 14 | Bipolar Family Study | 68 | 23.45 | 2.48 | 67.65 | 20.59 | 22.90 | 4.06 | 5 | 47 |
| 15 | BiDirect | 996 | 50.32 | 7.81 | 55.82 | 57.03 | 27.44 | 5.10 | 259 | 330 |
| 16 | MPIP. Scanner/Site 1 | 347 | 47.43 | 14.01 | 54.18 | 73.78 | 24.25 | 4.09 | 31 | 207 |
| 17 | MPIP2. Scanner/Site 2 | 178 | 50.58 | 12.30 | 60.67 | 37.08 | 24.68 | 3.68 | 14 | 101 |
| 18 | Pharmo (AMC) | 41 | 29.93 | 5.03 | 100.00 | 100.00 | 24.52 | 5.00 | 6 | 21 |
| 19 | Houston. Scanner/Site 1 | 74 | 39.59 | 13.63 | 70.27 | 100.00 | 27.86 | 6.72 | 22 | 23 |
| 20 | Houston. Scanner/Site 2 | 22 | 12.91 | 2.76 | 22.73 | 100.00 | 23.05 | 5.86 | 4 | 10 |
| 21 | Minnesota | 110 | 15.47 | 1.90 | 71.82 | 63.64 | 24.29 | 6.07 | 17 | 70 |
| 22 | Melbourne | 58 | 19.14 | 2.77 | 56.90 | 100.00 | 23.75 | 4.77 | 7 | 32 |
| 23 | FOR2107 - Münster | 772 | 36.21 | 13.36 | 63.47 | 45.47 | 25.45 | 5.35 | 129 | 415 |
| 24 | FOR2107 - Marburg | 407 | 31.85 | 12.33 | 64.13 | 43.49 | 24.22 | 4.98 | 46 | 269 |
| 25 | Muenster cohort | 571 | 38.66 | 11.44 | 52.54 | 32.40 | 25.16 | 4.56 | 74 | 312 |
| 26 | Novosibirsk. Scanner/Site 1 | 35 | 45.43 | 11.35 | 77.14 | 51.43 | 24.98 | 3.67 | 3 | 15 |
| 27 | Novosibirsk. Scanner/Site 2 | 92 | 43.35 | 11.73 | 61.96 | 61.96 | 25.75 | 5.60 | 17 | 46 |
| 28 | Stanford | 31 | 39.38 | 11.17 | 67.74 | 32.26 | 25.29 | 5.32 | 6 | 15 |
|  | Combined | 6420 | 42.91 | 15.26 | 56.95 | 45.19 | 25.97 | 4.97 | 1223 | 2917 |

| **Study** | **Sample characteristics** | **Diagnosis measurement** | **Exclusion criteria** |
| --- | --- | --- | --- |
| BiDirect | MDD patients who had been hospitalized due to depression at least once during the 12 months period prior to inclusion into the study, and population based healthy controls | M.I.N.I. International Neuropsychiatric Interview (German version 5.0.0) | MDD subjects: Exclusion criteria were (i) compulsory admission, (ii) comorbid dementia, and (iii) comorbid drug abuse (including alcohol). |
| Bipolar Family Study (Edinburgh) | The MDD group were originally people with a FHx of bipolar disorder | SCID interview | MDD subjects: presence of other axis I diagnoses. Control subjects: no medical history, including neurological and psychiatric history, as well as no previous or actual use of psychotropic medication All subjects: any major neurological disorder, learning disability, or any history of head injury that included loss of consciousness and any contraindications to MRI. |
| Calgary | First episode MDD and healthy controls (Dalhousie sample). Recurrent MDD and healthy controls (Calgary Sample) | KSADS | Dalhousie Sample: A history of neurological illness, medical illness, claustrophobia >21 year of age, or the presence of a ferrous implant or pacemaker. University of Calgary: Left handed; history of seizures. epilepsy or other neurological or psychiatric diagnoses (specifically bipolar disorder. psychosis. pervasive developmental disorder, eating disorders, PTSD); pregnancy |
| CODE (Berlin) | Chronic depression, at least for 1 year, subjects medication free (CBASP1); Chronic depression, at least 2 years, early onset (befor, 21 years), medicaton free (CBASP2) | SCID interview | MDD: Presence of any other Axis-1 diagnosis; Acute risk for suicide (in contrast to suicidal ideation); History of psychotic symptoms, bipolar disorder, or dementia; Schizotypal, antisocial or borderline personality disorder; Use of psychotropic medication within two weeks prior to the start of the study; No current psychotherapeutic treatment, Control subjects: No history of or current Axis-1 or 2 disorders, All subjects: History of or current neurological disorder or brain injury; Serious medical condition; Severe cognitive impairment; Substance-related abuse or dependence disorder; Use of psychotropic medication; Use of central-acting medication; Pregnancy; General MRI contraindications. |
| Dublin | 250 HC (3T) from other studies available too | SCID-1 interview | MDD subjects: comorbid psychiatric disorders (Axis I or Axis II, other than MDD). Treatment with antipsychotics or mood stabilizers, age 65, Control subjects: no Axis-I diagnosis, no medication use. All subjects: history of neurological or other severe medical illness, head injury or severe substance abuse in their lifetime history and general MRI contraindications. |
| FOR2107 | Participants recruited by means of public advertisement and from the inpatient services | SCID-1 | Inclusion criteria: age 18-65 years; patients were diagnosed with major depressive disorder by SCID-Interview, currently depressed or remitted, Exclusion criteria all: any MRI contraindications; any neurological abnormalities. Exclusion criteria controls: any current or former psychiatric disorder; Exclusion criteria patients: substance dependence or current benzodiazepine treatment (wash out of at least three half-lives before study participation)" |
| Groningen sample (DIP) | Outpatients with MDD diagnosis | MINI-SCAN | Inclusion MDD: Outpatients treated in mental health care for depression, BDI-II>13 at screening, adults. Exclusion MDD: Comorbid axis-I disorders other than anxiety disorders or past substance abuse, other psychotropic medication than stable use of SSRI/SNRI/TCA, established cardiovascular disease, active and concrete suicidal plans, inadequate language proficiency, cognitive impairments or neurological disease that interferes with task performance. Exclusion CTL: Same as MDD, lifetime history of MDD, BDI>8. |
| Houston | Outpatients | SCID interview | MDD subjects: age below 18; lifetime or current diagnosis of psychotic disorder, or bipolar I or II disorder; substance abuse/dependence in 6 months prior to study inclusion; current major medical problems. Control subjects: age below 18; current major medical problems; current psychiatric or neurologic disorder; history of psychiatric disorders in a first-degree relative; current major medical problems. Both groups: MRI contra-indications |
| Houston adolescents |  | major depressive disorder (MDD) diagnosis according to DSM-IV | MDD subjects: head trauma with residual effects, neurological disorders, uncontrolled major medical conditions based on patient self reports and current drug abuse. In addition, Healthy controls (HC) were excluded if they had a history of any Axis I disorder or had a first-degree relative with any Axis I disorder. |
| Magdeburg (SFB - Sexpect) |  | ICD-10 interview | MDD subjects: history of seizures, medication with glutamate modulating drugs (ketamine, riluzole, etc.) or benzodiazepines, prior electroconvulsive therapy (ECT) treatments and pregnancy, atypical forms of depression, any additional psychiatric disorder, and a history of substance abuse or dependence. Control subjects: psychiatric illness, Both groups: contraindications against MRI, major medical and neurological illness. |
| Melbourne | Youth depression sample: 15-25 years of age. Recruited as part of 2 large RCTs (incl. YoDA-C - Davey et al.. 2014; Trials) and scanned prior to treatment randomisation. 60 patients unmedicated (YoDA-C). Scanning is ongoing at a rate of 2-3 patients per week. | SCID interview | MDD subjects: lifetime or current SCID-I diagnosis of psychotic disorder. or bipolar I or II disorder, Control subjects: any SCID-I diagnosis or medication use. Both groups: Acute or unstable medical disorder; general MRI contraindications |
| Minnesota | Adolescents with MDD and HCs aged 12 to 19 years were recruited to participate through community postings and referrals from local mental health services. Adolescents with MDD were eligible if they had a primary diagnosis of MDD and had not received any psychotropic medication treatment for the past 2 months. Healthy adolescents were eligible if they had no current or past psychiatric diagnoses and were frequency matched to the MDD group on age and sex | Schedule for Affective Disorders and Schizophrenia for School-Age Children–Present and Lifetime Version and the Children’s Depression Rating Scale–Revised (CDRS-R). | Exclusion criteria for both groups included the presence of a neurologic or other chronic medical condition, mental retardation, pervasive developmental disorder, substance use disorder, bipolar disorder, or schizophrenia |
| MPIP | M. A. R. S. sample: both first and recurrent episodes; RUD sample: only recurrent episodes with some patients scanned in remission | M-CIDI/SCAN interview | 1. Munich Antidepressant Response Signature (MARS) study MDD subjects (clinical consensus diagnosis or M-CIDI (since 2008)): depressive syndromes secondary to any medical or neurological condition (e. g.. intoxication, drug abuse, stroke), the presence of manic, hypomanic or mixed affective symptoms, lifetime diagnosis of alcohol dependence, illicit drug abuse or the presence of severe medical conditions (e.g.. ischemic heart disease). Patients with bipolar depression were excluded for the current MR study. Control subjects: age > 65, MMSE<27, presence of severe somatic diseases or lifetime history of the following axis I disorders as assessed by the M-CIDI interview: alcohol dependence, drug abuse or dependence, possible psychotic disorder, mood disorder, anxiety disorder including OCD and PTSD, somatoform disorder, dissociative disorder NOS, and eating disorder 2, Recurrent unipolar depression (RUD) study: MDD subjects (SCAN interview): presence of manic episodes, mood incongruent psychotic symptoms, the presence of a lifetime diagnosis of intravenous drug abuse and depressive symptoms only secondary to alcohol or substance abuse or to medical illness or medication.Control subjects: presence of severe somatic diseases or life-time history of anxiety and affective disorders according to the Composite International Diagnostic-Screener (CIDI-S), All subjects: gross incidental MR findings such as territorial infarction, tumor, hydrocephalus. malformations and anatomical deviations (e.g. enlarged ventricles) that prevent appropriate image processing were additional exclusion criteria, 3, MR images of 9 additional controls acquired at the LMU, Munich, meeting equivalent criteria as the RUD control sample were included. |
| Münster Neuroimaging Cohort | Currently depressed (HAMD>18) inpatients | SCID interview | MDD subjects: presence of bipolar disorder, schizoaffective disorders and schizophrenia; substancerelated disorders or current benzodiazepine treatment (wash out of at least three half-lives before study participation), and former electroconvulsive therapy. Control subjects: any current or former psychiatric disorder. Both groups: any neurological abnormalities. MRI contra-indications |
| NESDA | 93 (60%) MDD patients have a comorbid ANX diagnosis | CIDI interview | MDD subjects: presence of axis-I disorders other than MDD, panic disorder, social anxiety disorder, or generalized anxiety disorder and any use of psychotropic medication other than stable use of SSRIs or infrequent benzodiazepine use (i.e.. equivalent to 2 doses of 10 mg of oxazepam 3 times per week or use within 48 hours prior to scanning). Control subjects: no Axis-I diagnosis, no medication use, All subjects: presence or history of major internal or neurological disorder, dependence on or recent abuse (past year) of alcohol and/or drugs, hypertension, and general MRI contraindications. |
| Novosibirsk | MDD Patients: 100% hospital-based, outpatients: 0%, general population: 0% | MINI. SCID. ICD-10 interviews | MDD subjects: Presence of axis-I disorders other than MDD, panic disorder, social anxiety disorder, or generalized anxiety disorder and any use of psychotropic medication other than stable use of SSRIs or infrequent benzodiazepine use; age 18 or below; alcohol or substance abuse/dependence within 6 months of study participation; current major medical problems. Control subjects: age over 65; any current or former psychiatric disorder. Both groups: MRI contra-indications. |
| Pharmo (AMC) | 48 subjects with lifetime diagnosis of either MDD and/or AD and 14 helthy controls. Patients were startified depending on exposure to SSRIs: early (before age 23) or late (after age 23) exposure to SSRI's, or no exposure at all (UN), 15 subjects were diagnosed with only MDD, 3 with only AD and 22 with both MDD and AD (8 subjects did not receive a diagnosis due to incomplete M.I.N.I. Plus assessment). According to the M.I.N.I. Plus, none of the HC subjects were ever diagnosed with MDD or AD | MINI Plus | Less than three week medication-free interval before scanning, current psychotropic medication use, a history of chronic or neurological disorder, family history of sudden heart failure or epileptic attacks, pregnancy (tested via urine sampling prior to the assessment), breast feeding, alcohol dependence and contra-indications for an MRI scan (e.g.. ferromagnetic fragments). Participants agreed to abstain from smoking, caffeine and alcohol use for 24 hours prior to the assessments. |
| SHIP | Community based sample | M-CIDI interview | MDD subjects: presence of axis-I disorders other than MDD, anxiety disorders, conversion, somatization and eating disorder, Control subjects: no lifetime diagnosis of depression, no antidepressiva, and severity index=0 All subjects: We removed subjects with medical conditions (e.g. a history of cerebral tumor, stroke, Parkinson’s diseases, multiple sclerosis, epilepsy, hydrocephalus, enlarged ventricles, pathological lesions) or due to technical reasons (e.g. severe movement artifacts or inhomogeneity of the magnetic field). |
| SHIP-trend | Community based sample | M-CIDI interview | MDD subjects: no special exclusion criteria Control subjects: no lifetime diagnosis of depression, no antidepressiva, and severity index=0 All subjects: We removed subjects with due to medical conditions (e.g. a history of cerebral tumor, stroke, Parkinson’s diseases, multiple sclerosis, epilepsy, hydrocephalus, enlarged ventricles, pathological lesions) or due to technical reasons (e.g. severe movement artifacts or inhomogeneity of the magnetic field). |
| Singapore |  | SCID interview | Inclusion: 1) DSM IV dx of MDD (Patients) 2) Age: 21-65 3) English speaking 4) Provision of informed written consent Exclusion criteria 1) History of significant head injury 2)Neurological diseases such as epilepsy, cerebrovascular accident 3) Impaired thyroid function 4) Steroid use 5) DSM IV alcohol or substance use or dependence 6) Contraindications to MRI (e.g. pacemaker, orbital foreign body, recent surgery/procedure with metallic devices/implants deployed) using standard MRI Request Form from NNI 7)Pregnant women 8) Claustrophobia |
| Stanford University | Community-based DSM-diagnosed sample | SCID interview | MDD subjects: presence of axis-I disorders other than MDD, anxiety and eating disorders , Control subjects: control individuals did not meet diagnostic criteria for any current psychiatric. Both groups: alcohol / substance abuse or dependence within six months prior to MRI scanning, history of head trauma with loss of consciousness > 5 min, aneurysm, or any neurological or metabolic disorders that require ongoing medication or that may affect the central nervous system (including thyroid disease, diabetes, epilepsy or other seizures, or multiple sclerosis), MRI contraindications, or bad MRI data (e.g.. extreme movement). |
| Sydney | We potentially have an additional 50-70 MDD patients | SCID interview | MDD subjects: presence of axis-I disorders other than MDD, panic disorder, social anxiety disorder, or generalized anxiety disorder, Control subjects: no Axis-I diagnosis. no medication use. Exclusion criteria for all subjects included medical instability (as determined by a psychiatrist). history of neurological disease (e.g. tumour, head trauma, epilepsy), medical illness known to impact cognitive and brain function (e.g. cancer), intellectual and/or developmental disability and insufficient English for neuropsychological assessment. All subjects were asked to abstain from drug or alcohol use for 48 hours prior to testing and informed about a drug screen protocol. |

| **Study** | **Scanner type** | **Sequence T1** | **FreeSurfer version** |
| --- | --- | --- | --- |
| BiDirect | 3 T Philips Intera scanner | 3D T1-weighted turbo field echo images were collected with a the following parameters: TR = 7.26. TE = 3.56. 9° flip angle. 160 sagittal slices. matrix dimension 256 x 256. FOV = 256 x 256mm. 2mm slice thickness (reconstructed to 1mm) and a resulting voxel size of 1x1x1mm | 5.3 |
| Bipolar Family Study (Edinburgh) | 1.5T GE Signa | T1-weighted sequence. TR=500 msec; TE=4 msec; flip angle 8°; matrix 192 x 192; 180 slices; voxel size 1.25 mm x 1.25 mm x 1.20 mm; FOV=24. phase FOV 1 | 5.3 |
| Calgary | 1.5T Siemens Magnetom Vision. 3T GE Discovery MR750 | 1.5T: A sagittal scout series was acquired to test image quality. 3D fast low angle shot (FLASH) sequence was used to acquire data from 124 1.5 mm-thick contiguous coronal slices through the entire brain (echo time = 5ms. repetition time = 25ms. acquisition matrix = 256 x 256 pixels. field of view = 24 cm and flip angle = 40°). 3T: Anatomical imaging acquisition parameters: axial acquisition. repetition time (TR). 2200 milliseconds (ms); echo time (TE). 3.04 ms; TI. 766. 780; flip angle. 13 degrees; 208 partitions; 256 × 256 matrix; and field of view. 256. | 5.3 |
| CODE (Berlin) | 3T Siemens Trio (4 Sites). 3 T Philips Achieva (1 site) | Siemens: T1 mprage. voxel size 1 mm x 1 mm x 1 mm; TR=1900 msec; TE=2.52 msec; Sample 1: 192 slices. Sample 2: 176 slices (except 1 site: 192) Philips: T1 3D-TFE. voxel size 1 mm x 1 mm x 1 mm; TR=8.3 msec; TE=3.8 msec; 170 slices. | 5.3 |
| Dublin | 3T Phillips Achieva; 1.5T Siemens Vision | 3T: A sagittal T1 3D TFE was used to scan all participants. TR=8.5 msec; TE=3.9 msec; FOV = 256 mm. AP: 256 mm. RL: 160 mm; matrix: 256×256. 1.5T: 3D-MPRAGE T1-weighted sequence. TR=11.6 msec; TE=4.9 msec; FOV=230 mm; matrix 512 x 512. slice thickness: 1.5 mm. | 5.3 |
| FOR2107 | Marburg: 3T Siemens Magnetom TiroTim syngo; Münster: 3T Siemens PRISMA | Marburg: - Sequence: 3D T1-weighted magnetization prepared rapid acquisition gradient echo (MPRAGE) - Sagittal Acquisition Direction. # of Slices 176. 0.5mm Slice Gap. 1.0x1.0x1.0 Voxel Size (mm3). TI 900 ms. TE 2.26 ms. TR 1900 ms. Flip Angle 9. Münster: - Sequence: 3D T1-weighted magnetization prepared rapid acquisition gradient echo (MPRAGE). - Sagittal Acquisition Direction. # of Slices 192. 0mm Slice Gap. 1.0x1.0x1.0 Voxel Size (mm3). TI 900 ms. TE 2.28 ms. TR 1900 ms. Flip Angle 8 | 5.3 |
| Groningen sample (DIP) | 3T Philips | 3D T1-weighted scan (170 slices; TR = 9ms; TE = 3.6ms; 256x231 matrix of 1×1×1 mm voxels) | 5.3 |
| Houston | 1.5 T Philips Medical Systems Gyroscan Intera | T -1 weighted fast field echo sequence (3D T1 -FFE) with repetition time (TR) = 25 ms. echo time (TE) = 5 ms. field of view (FOV) = 240 mm × 220 mm. gap = 0. and matrix size = 256 × 256. | 5.3 |
| Houston adolescents | Philips 1.5 Tesla MRI scanner | A three-dimensional axial fast field echo sequence with the following parameters: repetition time (TR) = 24 ms. echo time (TE) = 5 ms. flip angle = 40°. field of view (FOV) = 256 mm. slice thickness = 1 mm. matrix size = 256 × 256 and 150 slices. | 5.3 |
| Magdeburg (SFB - Sexpect) | 3 Tesla Siemens MAGNETOM Trio scanner (Siemens. Erlangen. Germany) | High resolution T1 -weighted structural MRI scans of the brain were acquired for structural reference using a 3D -MPRAGE sequence (TE = 4.77 ms. TR = 2500 ms. T1 = 1100 ms. flip angle = 7°. bandwidth = 140 Hz/pixel. acquisition matrix = 256 × 256 × 192. isometric voxel size = 1.0 mm3). | 5.3 |
| Melbourne | 3T GE Signa Excite | 3D BRAVO sequence 140; TR=7900 ms; TE=3000 ms; flip angle=13º; FOV=256 mm; matrix=256 x 256 | 5.3 |
| Minnesota | 3.0 Tesla Tim Trio scanner; Siemens Corp | A 5-minute structural scan was acquired using a T1-weighted. high-resolution. magnetization-prepared gradient-echo sequence: repetition time. 2530 milliseconds; echo time. 3.65 milliseconds; inversion time. 1100 milliseconds; flip angle. 7°; field of view. 256 × 176 mm; voxel size. 1-mm isotropic; 224 slices; and generalized. autocalibrating. partially parallel acquisition acceleration factor. 2. | 5.3 |
| MPIP | 1.5T GE and Siemens (the latter: only few cases) | #1: T1-weighted SPGR sagittal 3D volume. TR=1030 msec; TE=3.4 msec; 124 slices; matrix=256x256; FOV=23.0x23.0 cm2; voxel size=0.8975 mm x0.8975 mm x 1.2- 1.4 mm; flip angle=90°; birdcage resonator. #2: same scanner as #1. platform update Signa Excite. sagittal T1-weighted (spin echo sequence. TR=9.7 msec. TE=2.1 msec; FOV=25.0x25.0 cm2. voxel size=0.875 mm x0.875 mm x1.2 mm. 124- 132 slices. flip angle=90°. #3: Siemens 1.5 Tesla. Vario. 3D MPRAGE. TR=11.6 msec; TE=4.9 msec; FOV 23x23 cm2; matrix 512x512; 126 axial slices; voxel site 0.45 mm x 0.45 mm x 1.5 mm. (only N=2 subjects) | 5.3 |
| Münster Neuroimaging Cohort | 3T Philips Gyroscan Intera | 3D fast gradient echo sequence (turbo field echo). repetition time = 7.4 milliseconds. echo time = 3.4 milliseconds. flip angle = 9°. two signal averages. inversion prepulse every 814.5 milliseconds. acquired over a field of view of 256 (feet -head [FH]) × 204 (anterior -posterior [AP]) × 160 (right -left [RL]) mm. phase encoding in AP and RL direction. reconstructed to cubic voxels of .5 mm × .5 mm × .5 mm | 5.3 |
| NESDA | 3T Phillips Achieva/Intera | 3D gradient-echo T1-weighted sequence. TR=9 msec; TE=3.5 msec; flip angle 8º. FOV = 256 mm; matrix: 25x62x56; in plane voxel size = 1 mm × 1 mm x 1 mm; 170 slices. | 5.3 |
| Novosibirsk | 3T GE Discovery™ MR750w | Whole-brain T1-weighted images - 3D fast spin gradient echo sequence (FSPGR BRAVO). repetition time = 9.5 ms. echo time = 3.7 ms. flip angle = 3°. acquired over a field of view of 256 (feet-head [FH]) × 256 (anterior-posterior [AP]) × 188 (rightleft [RL]) mm. reconstructed to cubic voxels of 1 mm × 1 mm × 1 mm | 5.3 |
| Pharmo (AMC) | 3T Philips | T1 sequence details: 3D-TFE sequence TR= 9.7 ms. TE=4.6ms. matrix 192x192. voxel size = 0.875 x 0.875 x 1.2 mm; 120 slices. Axial plane. Philips 3T Ingenia 16 channel coil | 5.3 |
| SHIP | 1.5T Siemens Avanto | 3D T1-weighted (MP-RAGE/ axial plane); TR=1900 msec; TE=3.4 msec; Flip angle=15°; voxel size 1 mm x 1 mm x 1 mm | 5.3 |
| SHIP-trend | 1.5T Siemens Avanto | 3D T1-weighted (MP-RAGE/ axial plane); TR=1900 msec; TE=3.4 msec; Flip 5.3 Axial Centos6_x86_64 angle=15°; voxel size 1 mm x 1 mm x 1 mm | 5.3 |
| Singapore | Achieva 3T. Philips Medical Systems. Netherlands | Whole brain high resolution 3D MP-RAGE (magnetisation-prepared rapid acquisition with a gradient echo) volumetric scans (TR/TE/TI/flip angle 8.4/3.8/3000/8; matrix 256x204; FOV 240mm2) with axial orientation (reformatted to coronal) | 5.3 |
| Stanford University | 1.5T GE Signa Excite | Whole-brain T1-weighted images were collected using a spoiled gradient echo (SPGR) pulse sequence (116 sagittal slices; through-plane resolution = 1.5 mm; in-plane resolution = 0.86 x 0.86 mm; flip angle = 15 degrees; repetition time [TR] = 8.3-10.1 ms; echo time [TE] = 1.7-3.0; inversion time [TI] = 300 ms; matrix = 256 x 192). | 5.3 |
| Sydney | 3T GE MR750 | 3D T1-weighted sequence. TR=7.2 msec; TE=2.78 msec; matrix =256; FOV=240; No. slices=196; thick=0.9mm; inplane resolution=0.9375 | 5.1 |

| **Study** | **Genotyping Platform** | **Details on QC. Inclusion and exclusion criteria** |
| --- | --- | --- |
| BiDirect | Illumina Human Core Exome chip va. 1.1 (Psych-chip) | Exclusion criteria: Sex inconsistencies (X-chromosome heterozygosity), genetically related individuals were not included in the present study. Genotyping rate <95%, strand ambiguity (A/T and C/G SNPs), MAF<1%, Hardy-Weinberg Equilibrium (p<10-6) |
| FOR2107 | Infinium PsychArray-24 | Exclusion criteria: Sex inconsistencies (X-chromosome heterozygosity), genetically related individuals were not included in the present study, Genotyping rate <95%, strand ambiguity (A/T and C/G SNPs), MAF<1%, Hardy-Weinberg Equilibrium (p<10-6) |
| Münster Neuroimaging Cohort | Infinium PsychArray-24 | Exclusion criteria: Sex inconsistencies (X-chromosome heterozygosity), genetically related individuals were not included in the present study, Genotyping rate <95%, strand ambiguity (A/T and C/G SNPs), MAF<1%, Hardy-Weinberg Equilibrium (p<10-6) |
| NESDA | Affymetrix Perlegen 5.0 and Affymetrix 6.0 Human SNP array | SNP QC was done on each platform separately removing SNP under the following criteria: SNP were only selected if they could be aligned to the reference panel forward strand, SNPs where the allele frequencies differed more than 10% with the reference allele frequency were excluded, SNPs with MAF < 0.005 were excluded, SNPs with a significant deviation from Hardy-Weinberg Equilibrium (HWE) p < 10-12 were excluded, as well as SNPs with a genotype call rate less than 95%. Missing SNP genotypes between each platform were then imputed using the GONL (Genomes of the Netherlands) reference dataset.  Then the SNPs were filtered with the following more stringent criteria: SNP significantly associated with a single genotyping platform as compared to the other platforms (p < 10-5); SNPs had an allele frequency difference larger than 10% with the GONL reference set; HWE p < 10-5, Mendelian error rate > mean + 5 sd (N>40) over all SNPs, and if the imputation quality R2 was less than 0.90. |
| SHIP | Affymetrix Human SNP Array 6.0 | Inclusion criteria: Hardy-Weinberg Equilibrium (p>10-4), callrate 80% |
| SHIP-trend | Illumina Human Omni 2.5 | Inclusion criteria: Hardy-Weinberg Equilibrium (p>10-4), callrate 90% |

| **Label** | **Estimate** | **StdError** | **T** | **p** | **FDR adjusted p** | **Cohen´s d** | **N** |
| --- | --- | --- | --- | --- | --- | --- | --- |
|  |  |  |  |  |  |  |  |
| **Global measures** |  |  |  |  |  |  |  |
| Total Intracranial Volume | -1539.00000 | 415.000000 | -3.71 | 2.10E-04 | 0.0006 | -0.0952 | 6112 |
| Total left hemispheral surface area | -32.450000 | 18.890000 | -1.72 | 8.59E-02 | 0.1237 | -0.0435 | 6287 |
| Total right hemispheral surface area | -34.860000 | 18.930000 | -1.84 | 6.56E-02 | 0.0963 | -0.0465 | 6287 |
| Left hemispheral average thickness | -0.001732 | 0.000245 | -7.07 | 1.70E-12 | <0.0001 | -0.1785 | 6306 |
| Right hemispheral average thickness | -0.001610 | 0.000245 | -6.58 | 5.21E-11 | <0.0001 | -0.1662 | 6305 |
|  |  |  |  |  |  |  |  |
| **Cortical thickness** |  |  |  |  |  |  |  |
| Left banks of the superior temporal sulcus | -0.002974 | 0.000465 | -6.39 | 1.78E-10 | <0.0001 | -0.1658 | 5977 |
| Left caudal anterior cingulate cortex | -0.001755 | 0.000645 | -2.72 | 6.50E-03 | 0.0131 | -0.0688 | 6285 |
| Left caudal middle frontal gyrus | -0.001990 | 0.000389 | -5.12 | 3.15E-07 | <0.0001 | -0.1297 | 6263 |
| Left cuneus | -0.000453 | 0.000358 | -1.26 | 2.10E-01 | 0.2659 | -0.0321 | 6195 |
| Left entorhinal cortex | -0.005557 | 0.000972 | -5.72 | 1.14E-08 | <0.0001 | -0.1473 | 6066 |
| Left fusiform gyrus | -0.004144 | 0.000385 | -10.75 | <2.00E-16 | <0.0001 | -0.2721 | 6276 |
| Left inferior parietal cortex | -0.001379 | 0.000348 | -3.96 | 7.52E-05 | 0.0002 | -0.1007 | 6220 |
| Left inferior temporal gyrus | -0.002977 | 0.000439 | -6.78 | 1.30E-11 | <0.0001 | -0.1728 | 6193 |
| Left isthmus cingulate cortex | -0.002455 | 0.000522 | -4.70 | 2.64E-06 | <0.0001 | -0.1189 | 6284 |
| Left lateral occipital cortex | -0.000662 | 0.000319 | -2.08 | 3.80E-02 | 0.0621 | -0.0527 | 6267 |
| Left lateral orbitofrontal cortex | -0.001511 | 0.000431 | -3.51 | 4.50E-04 | 0.0012 | -0.0889 | 6267 |
| Left lingual gyrus | -0.000816 | 0.000325 | -2.51 | 1.20E-02 | 0.0219 | -0.0636 | 6254 |
| Left medial orbitofrontal cortex | -0.001754 | 0.000430 | -4.08 | 4.50E-05 | 0.0001 | -0.1038 | 6214 |
| Left middle temporal gyrus | -0.003220 | 0.000446 | -7.22 | 5.70E-13 | <0.0001 | -0.1859 | 6063 |
| Left parahippcampal gyrus | -0.003557 | 0.000834 | -4.26 | 2.05E-05 | 0.0001 | -0.1078 | 6275 |
| Left paracentral lobule | -0.001884 | 0.000399 | -4.72 | 2.38E-06 | <0.0001 | -0.1194 | 6282 |
| Left pars opercularis | -0.002004 | 0.000388 | -5.16 | 2.50E-07 | <0.0001 | -0.1307 | 6264 |
| Left pars orbitalis | -0.001378 | 0.000588 | -2.34 | 1.91E-02 | 0.0322 | -0.0592 | 6279 |
| Left pars triangularis | -0.000665 | 0.000422 | -1.58 | 1.15E-01 | 0.1581 | -0.0400 | 6268 |
| Left pericalcarine | 0.000490 | 0.000350 | 1.40 | 1.62E-01 | 0.2158 | 0.0357 | 6190 |
| Left precentral gyrus | -0.000095 | 0.000316 | -0.30 | 7.63E-01 | 0.8040 | -0.0076 | 6225 |
| Left posterior cingulate cortex | -0.002651 | 0.000421 | -6.30 | 3.24E-10 | <0.0001 | -0.1593 | 6287 |
| Left precentral gyrus | -0.002080 | 0.000368 | -5.66 | 1.63E-08 | <0.0001 | -0.1436 | 6246 |
| Left precuneus | -0.001573 | 0.000339 | -4.64 | 3.60E-06 | <0.0001 | -0.1175 | 6272 |
| Left rostral anterior cingulate cortex | -0.003289 | 0.000619 | -5.32 | 1.09E-07 | <0.0001 | -0.1349 | 6255 |
| Left rostral middle frontal gyrus | -0.001299 | 0.000361 | -3.60 | 3.20E-04 | 0.0009 | -0.0911 | 6276 |
| Left superior frontal gyrus | -0.002031 | 0.000377 | -5.39 | 7.41E-08 | <0.0001 | -0.1365 | 6272 |
| Left superior parietal cortex | -0.000919 | 0.000310 | -2.96 | 3.10E-03 | 0.0071 | -0.0751 | 6239 |
| Left superior temporal gyrus | -0.003054 | 0.000430 | -7.11 | 1.34E-12 | <0.0001 | -0.1849 | 5949 |
| Left supramarginal gyrus | -0.001684 | 0.000361 | -4.66 | 3.20E-06 | <0.0001 | -0.1194 | 6128 |
| Left frontal pole | -0.002190 | 0.000803 | -2.73 | 6.41E-03 | 0.0131 | -0.0690 | 6300 |
| Left temporal pole | -0.004088 | 0.000985 | -4.15 | 3.40E-05 | 0.0001 | -0.1052 | 6262 |
| Left transverse temporal gyrus | -0.003635 | 0.000580 | -6.27 | 3.96E-10 | <0.0001 | -0.1587 | 6276 |
| Left insula | -0.002465 | 0.000429 | -5.74 | 9.83E-09 | <0.0001 | -0.1460 | 6217 |
| Right banks of the superior temporal sulcus | -0.002332 | 0.000463 | -5.04 | 4.89E-07 | <0.0001 | -0.1287 | 6165 |
| Right caudal anterior cingulate cortex | -0.001890 | 0.000599 | -3.16 | 1.60E-03 | 0.0039 | -0.0799 | 6288 |
| Right caudal middle frontal gyrus | -0.001369 | 0.000387 | -3.54 | 4.10E-04 | 0.0011 | -0.0896 | 6271 |
| Right cuneus | -0.000607 | 0.000361 | -1.68 | 9.30E-02 | 0.1315 | -0.0428 | 6199 |
| Right entorhinal cortex | -0.003661 | 0.001050 | -3.49 | 4.90E-04 | 0.0013 | -0.0903 | 6010 |
| Right fusiform gyrus | -0.004101 | 0.000391 | -10.48 | <2.00E-16 | <0.0001 | -0.2651 | 6281 |
| Right inferior parietal cortex | -0.000885 | 0.000340 | -2.60 | 9.20E-03 | 0.0176 | -0.0661 | 6224 |
| Right inferior temporal gyrus | -0.003201 | 0.000447 | -7.16 | 9.20E-13 | <0.0001 | -0.1819 | 6227 |
| Right isthmus cingulate cortex | -0.002095 | 0.000509 | -4.12 | 3.84E-05 | 0.0001 | -0.1042 | 6284 |
| Right lateral occipital cortex | -0.000389 | 0.000330 | -1.18 | 2.40E-01 | 0.2967 | -0.0299 | 6271 |
| Right lateral orbitofrontal cortex | -0.001885 | 0.000429 | -4.39 | 1.15E-05 | <0.0001 | -0.1111 | 6283 |
| Right lingual gyrus | -0.000545 | 0.000325 | -1.68 | 9.40E-02 | 0.1318 | -0.0426 | 6239 |
| Right medial orbitofrontal cortex | -0.002665 | 0.000445 | -5.99 | 2.17E-09 | <0.0001 | -0.1521 | 6237 |
| Right middle temporal gyrus | -0.002983 | 0.000430 | -6.93 | 4.52E-12 | <0.0001 | -0.1763 | 6212 |
| Right parahippcampal gyrus | -0.003953 | 0.000714 | -5.53 | 3.27E-08 | <0.0001 | -0.1399 | 6280 |
| Right paracentral lobule | -0.001571 | 0.000399 | -3.94 | 8.36E-05 | 0.0003 | -0.0996 | 6286 |
| Right pars opercularis | -0.002100 | 0.000407 | -5.16 | 2.52E-07 | <0.0001 | -0.1310 | 6242 |
| Right pars orbitalis | -0.001733 | 0.000575 | -3.01 | 2.60E-03 | 0.0061 | -0.0762 | 6277 |
| Right pars triangularis | -0.001396 | 0.000414 | -3.37 | 7.50E-04 | 0.0019 | -0.0855 | 6241 |
| Right pericalcarine | 0.000094 | 0.000354 | 0.26 | 7.90E-01 | 0.8269 | 0.0066 | 6172 |
| Right precentral gyrus | -0.000120 | 0.000319 | -0.38 | 7.07E-01 | 0.7603 | -0.0096 | 6245 |
| Right posterior cingulate cortex | -0.003072 | 0.000409 | -7.52 | 6.47E-14 | <0.0001 | -0.1901 | 6290 |
| Right precentral gyrus | -0.002034 | 0.000372 | -5.47 | 4.58E-08 | <0.0001 | -0.1387 | 6254 |
| Right precuneus | -0.001761 | 0.000335 | -5.25 | 1.60E-07 | <0.0001 | -0.1329 | 6274 |
| Right rostral anterior cingulate cortex | -0.002551 | 0.000607 | -4.20 | 2.72E-05 | 0.0001 | -0.1065 | 6258 |
| Right rostral middle frontal gyrus | -0.001584 | 0.000360 | -4.40 | 1.09E-05 | <0.0001 | -0.1114 | 6269 |
| Right superior frontal gyrus | -0.002464 | 0.000377 | -6.54 | 6.80E-11 | <0.0001 | -0.1655 | 6277 |
| Right superior parietal cortex | -0.000276 | 0.000310 | -0.89 | 3.73E-01 | 0.4403 | -0.0226 | 6256 |
| Right superior temporal gyrus | -0.002844 | 0.000421 | -6.76 | 1.56E-11 | <0.0001 | -0.1738 | 6081 |
| Right supramarginal gyrus | -0.001304 | 0.000362 | -3.60 | 3.20E-04 | 0.0009 | -0.0921 | 6145 |
| Right frontal pole | -0.001964 | 0.000793 | -2.48 | 1.33E-02 | 0.0234 | -0.0627 | 6296 |
| Right temporal pole | -0.003555 | 0.001019 | -3.49 | 4.90E-04 | 0.0013 | -0.0884 | 6267 |
| Right transverse temporal gyrus | -0.002507 | 0.000610 | -4.11 | 4.02E-05 | 0.0001 | -0.1041 | 6267 |
| Right insula | -0.001959 | 0.000456 | -4.30 | 1.74E-05 | 0.0001 | -0.1098 | 6165 |
|  |  |  |  |  |  |  |  |
| **Subcortical volumes** |  |  |  |  |  |  |  |
| Left lateral ventricle | 17.098101 | 12.555127 | 1.36 | 1.73E-01 | 0.2267 | 0.0352 | 5991 |
| Right lateral ventricle | 23.386510 | 11.654216 | 2.01 | 4.48E-02 | 0.0704 | 0.0521 | 5976 |
| Left thalamus | 13.487006 | 1.907018 | 7.07 | 1.70E-12 | <0.0001 | 0.1835 | 5971 |
| Right thalamus | 8.331665 | 1.646517 | 5.06 | 4.31E-07 | <0.0001 | 0.1315 | 5957 |
| Left caudate | 0.399762 | 1.119659 | 0.36 | 7.21E-01 | 0.7701 | 0.0093 | 5968 |
| Right caudate | 0.462746 | 1.131673 | 0.41 | 6.83E-01 | 0.7442 | 0.0107 | 5914 |
| Left putamen | 3.926520 | 1.530496 | 2.57 | 1.03E-02 | 0.0195 | 0.0678 | 5773 |
| Right putamen | 3.470803 | 1.421466 | 2.44 | 1.47E-02 | 0.0253 | 0.0640 | 5842 |
| Left pallidum | -1.429881 | 0.579377 | -2.47 | 1.36E-02 | 0.0238 | -0.0656 | 5702 |
| Right pallidum | -0.330883 | 0.516112 | -0.64 | 5.21E-01 | 0.5933 | -0.0167 | 5925 |
| Left hippocampus | 3.284155 | 1.026195 | 3.20 | 1.40E-03 | 0.0035 | 0.0832 | 5943 |
| Right hippocamups | 4.006099 | 1.019494 | 3.93 | 8.61E-05 | 0.0003 | 0.1018 | 5994 |
| Left amygdala | 2.764486 | 0.482574 | 5.73 | 1.06E-08 | <0.0001 | 0.1489 | 5956 |
| Right amygdala | 4.481277 | 0.513217 | 8.73 | <2.00E-16 | <0.0001 | 0.2266 | 5967 |
| Left nucleus accumbens | 0.787358 | 0.257643 | 3.06 | 2.25E-03 | 0.0054 | 0.0802 | 5856 |
| Right nucleus accumbens | 0.050514 | 0.235721 | 0.21 | 8.30E-01 | 0.8633 | 0.0055 | 5879 |
|  |  |  |  |  |  |  |  |
| **Cortical surface area** |  |  |  |  |  |  |  |
| Left banks of the superior temporal sulcus | -0.834611 | 0.426178 | -1.96 | 5.02E-02 | 0.0761 | -0.0527 | 5566 |
| Left caudal anterior cingulate cortex | 0.032111 | 0.373745 | 0.09 | 9.32E-01 | 0.9496 | 0.0023 | 5904 |
| Left caudal middle frontal gyrus | 0.789925 | 0.938247 | 0.84 | 4.00E-01 | 0.4685 | 0.0219 | 5904 |
| Left cuneus | -0.620187 | 0.560578 | -1.11 | 2.69E-01 | 0.3289 | -0.0291 | 5859 |
| Left entorhinal cortex | -0.008303 | 0.216400 | -0.04 | 9.69E-01 | 0.9756 | -0.0011 | 5555 |
| Left fusiform gyrus | -2.624635 | 0.946967 | -2.77 | 5.60E-03 | 0.0119 | -0.0730 | 5799 |
| Left inferior parietal cortex | -2.690404 | 1.515370 | -1.78 | 7.59E-02 | 0.1103 | -0.0467 | 5853 |
| Left inferior temporal gyrus | -2.863703 | 1.128756 | -2.54 | 1.12E-02 | 0.0210 | -0.0665 | 5876 |
| Left isthmus cingulate cortex | 2.442238 | 0.409288 | 5.97 | 2.56E-09 | <0.0001 | 0.1551 | 5962 |
| Left lateral occipital cortex | -2.699240 | 1.435587 | -1.88 | 6.00E-02 | 0.0889 | -0.0489 | 5946 |
| Left lateral orbitofrontal cortex | 0.897924 | 0.665901 | 1.35 | 1.78E-01 | 0.2286 | 0.0350 | 5979 |
| Left lingual gyrus | -3.002335 | 1.023718 | -2.93 | 3.40E-03 | 0.0076 | -0.0762 | 5944 |
| Left medial orbitofrontal cortex | 0.356810 | 0.576289 | 0.62 | 5.36E-01 | 0.6052 | 0.0163 | 5841 |
| Left middle temporal gyrus | -1.211872 | 0.963836 | -1.26 | 2.09E-01 | 0.2659 | -0.0335 | 5678 |
| Left parahippcampal gyrus | 0.473173 | 0.240133 | 1.97 | 4.88E-02 | 0.0759 | 0.0514 | 5897 |
| Left paracentral lobule | 1.644390 | 0.487839 | 3.37 | 7.50E-04 | 0.0019 | 0.0885 | 5838 |
| Left pars opercularis | 0.308584 | 0.664813 | 0.46 | 6.43E-01 | 0.7055 | 0.0120 | 5921 |
| Left pars orbitalis | 0.000812 | 0.207777 | 0.00 | 1.00E+00 | 1.0000 | <0.0001 | 5968 |
| Left pars triangularis | -0.074609 | 0.511034 | -0.15 | 8.84E-01 | 0.9130 | -0.0039 | 5943 |
| Left pericalcarine | -1.737076 | 0.620443 | -2.80 | 5.13E-03 | 0.0110 | -0.0732 | 5888 |
| Left precentral gyrus | 1.459335 | 1.060249 | 1.38 | 1.69E-01 | 0.2226 | 0.0362 | 5838 |
| Left posterior cingulate cortex | 1.163961 | 0.446326 | 2.61 | 9.10E-03 | 0.0176 | 0.0678 | 5965 |
| Left precentral gyrus | 2.479247 | 1.211486 | 2.05 | 4.08E-02 | 0.0654 | 0.0536 | 5881 |
| Left precuneus | 2.652236 | 1.052745 | 2.52 | 1.20E-02 | 0.0219 | 0.0655 | 5949 |
| Left rostral anterior cingulate cortex | 1.041822 | 0.382018 | 2.73 | 6.41E-03 | 0.0131 | 0.0718 | 5816 |
| Left rostral middle frontal gyrus | -2.059856 | 1.662525 | -1.24 | 2.15E-01 | 0.2705 | -0.0322 | 5952 |
| Left superior frontal gyrus | 5.020245 | 1.775526 | 2.83 | 4.71E-03 | 0.0103 | 0.0741 | 5859 |
| Left superior parietal cortex | 3.011377 | 1.538615 | 1.96 | 5.04E-02 | 0.0761 | 0.0513 | 5875 |
| Left superior temporal gyrus | 1.741044 | 1.024431 | 1.70 | 8.93E-02 | 0.1274 | 0.0457 | 5577 |
| Left supramarginal gyrus | 2.670921 | 1.328261 | 2.01 | 4.44E-02 | 0.0704 | 0.0534 | 5701 |
| Left frontal pole | 0.147775 | 0.092266 | 1.60 | 1.09E-01 | 0.1518 | 0.0415 | 5990 |
| Left temporal pole | -0.007228 | 0.159374 | -0.05 | 9.64E-01 | 0.9756 | -0.0013 | 5954 |
| Left transverse temporal gyrus | 0.862081 | 0.192384 | 4.48 | 7.57E-06 | <0.0001 | 0.1161 | 5986 |
| Left insula | 1.732343 | 0.579096 | 2.99 | 2.79E-03 | 0.0064 | 0.0781 | 5899 |
| Right banks of the superior temporal sulcus | 0.133055 | 0.348821 | 0.38 | 7.03E-01 | 0.7603 | 0.0100 | 5804 |
| Right caudal anterior cingulate cortex | 0.385845 | 0.404337 | 0.95 | 3.40E-01 | 0.4044 | 0.0247 | 5930 |
| Right caudal middle frontal gyrus | -0.635532 | 0.933075 | -0.68 | 4.96E-01 | 0.5724 | -0.0177 | 5922 |
| Right cuneus | -0.355660 | 0.527835 | -0.67 | 5.01E-01 | 0.5736 | -0.0175 | 5874 |
| Right entorhinal cortex | -0.113681 | 0.219296 | -0.52 | 6.04E-01 | 0.6728 | -0.0141 | 5468 |
| Right fusiform gyrus | -0.310302 | 0.931543 | -0.33 | 7.39E-01 | 0.7840 | -0.0087 | 5817 |
| Right inferior parietal cortex | -2.409353 | 1.646776 | -1.46 | 1.44E-01 | 0.1941 | -0.0383 | 5850 |
| Right inferior temporal gyrus | -3.219989 | 1.056002 | -3.05 | 2.30E-03 | 0.0055 | -0.0796 | 5911 |
| Right isthmus cingulate cortex | 2.000339 | 0.375249 | 5.33 | 1.01E-07 | <0.0001 | 0.1383 | 5976 |
| Right lateral occipital cortex | -1.400842 | 1.435896 | -0.98 | 3.29E-01 | 0.3943 | -0.0255 | 5962 |
| Right lateral orbitofrontal cortex | 1.529912 | 0.715825 | 2.14 | 3.26E-02 | 0.0539 | 0.0555 | 5984 |
| Right lingual gyrus | -2.512432 | 1.006531 | -2.50 | 1.26E-02 | 0.0224 | -0.0650 | 5942 |
| Right medial orbitofrontal cortex | 0.761820 | 0.496478 | 1.53 | 1.25E-01 | 0.1706 | 0.0399 | 5909 |
| Right middle temporal gyrus | -1.029303 | 0.937606 | -1.10 | 2.72E-01 | 0.3289 | -0.0289 | 5839 |
| Right parahippcampal gyrus | 0.293984 | 0.238888 | 1.23 | 2.19E-01 | 0.2723 | 0.0321 | 5909 |
| Right paracentral lobule | 2.105975 | 0.542514 | 3.88 | 1.00E-04 | 0.0003 | 0.1013 | 5904 |
| Right pars opercularis | 0.278339 | 0.598496 | 0.47 | 6.42E-01 | 0.7055 | 0.0123 | 5897 |
| Right pars orbitalis | -0.173042 | 0.243075 | -0.71 | 4.77E-01 | 0.5542 | -0.0184 | 5964 |
| Right pars triangularis | -0.316251 | 0.600166 | -0.53 | 5.98E-01 | 0.6709 | -0.0138 | 5909 |
| Right pericalcarine | -1.547545 | 0.643596 | -2.40 | 1.62E-02 | 0.0277 | -0.0628 | 5882 |
| Right precentral gyrus | 2.184387 | 1.056629 | 2.07 | 3.88E-02 | 0.0627 | 0.0541 | 5883 |
| Right posterior cingulate cortex | 1.482259 | 0.436404 | 3.40 | 6.90E-04 | 0.0018 | 0.0882 | 5972 |
| Right precentral gyrus | 1.711024 | 1.264516 | 1.35 | 1.76E-01 | 0.2285 | 0.0353 | 5894 |
| Right precuneus | 2.883930 | 1.103280 | 2.61 | 8.97E-03 | 0.0176 | 0.0678 | 5959 |
| Right rostral anterior cingulate cortex | 0.872489 | 0.348092 | 2.51 | 1.22E-02 | 0.0221 | 0.0656 | 5894 |
| Right rostral middle frontal gyrus | -4.687853 | 1.705131 | -2.75 | 5.99E-03 | 0.0125 | -0.0715 | 5947 |
| Right superior frontal gyrus | 4.796697 | 1.797443 | 2.67 | 7.64E-03 | 0.0152 | 0.0697 | 5897 |
| Right superior parietal cortex | 1.615187 | 1.464990 | 1.10 | 2.70E-01 | 0.3289 | 0.0287 | 5903 |
| Right superior temporal gyrus | 1.774857 | 0.903370 | 1.96 | 4.95E-02 | 0.0761 | 0.0520 | 5707 |
| Right supramarginal gyrus | 1.806517 | 1.238305 | 1.46 | 1.45E-01 | 0.1941 | 0.0386 | 5760 |
| Right frontal pole | 0.012376 | 0.115771 | 0.11 | 9.15E-01 | 0.9388 | 0.0029 | 5989 |
| Right temporal pole | 0.330919 | 0.174408 | 1.90 | 5.78E-02 | 0.0865 | 0.0496 | 5891 |
| Right transverse temporal gyrus | 0.320753 | 0.147399 | 2.18 | 2.96E-02 | 0.0494 | 0.0565 | 5991 |
| Right insula | 1.878089 | 0.653863 | 2.87 | 4.10E-03 | 0.0091 | 0.0753 | 5849 |

| **Label** | **Estimate** | **StdError** | **T** | **p** | **FDR adjusted p** | **Cohen´s d** | **N Obese** | **N NW** |
| --- | --- | --- | --- | --- | --- | --- | --- | --- |
|  |  |  |  |  |  |  |  |  |
| **Global measures** |  |  |  |  |  |  |  |  |
| Total Intracranial Volume | -21634.0000 | 5603.000000 | -3.86 | 1.10E-04 | 0.0005 | -0.1348 | 1168 | 2755 |
| Total left hemispheral surface area | -654.300000 | 256.890000 | -2.55 | 1.09E-02 | 0.0281 | -0.0880 | 1189 | 2872 |
| Total right hemispheral surface area | -708.380000 | 258.090000 | -2.74 | 6.08E-03 | 0.0165 | -0.0945 | 1189 | 2872 |
| Left hemispheral average thickness | -0.021001 | 0.003372 | -6.23 | 5.18E-10 | <0.0001 | -0.2143 | 1200 | 2865 |
| Right hemispheral average thickness | -0.019900 | 0.003379 | -5.89 | 4.18E-09 | <0.0001 | -0.2026 | 1200 | 2865 |
|  |  |  |  |  |  |  |  |  |
| **Cortical thickness** |  |  |  |  |  |  |  |  |
| Left banks of the superior temporal sulcus | -0.031088 | 0.006365 | -4.88 | 1.08E-06 | <0.0001 | -0.1724 | 1139 | 2708 |
| Left caudal anterior cingulate cortex | -0.014292 | 0.008888 | -1.61 | 1.08E-01 | 0.1803 | -0.0554 | 1198 | 2852 |
| Left caudal middle frontal gyrus | -0.026321 | 0.005381 | -4.89 | 1.04E-06 | <0.0001 | -0.1686 | 1196 | 2840 |
| Left cuneus | -0.002227 | 0.004912 | -0.45 | 6.50E-01 | 0.6990 | -0.0156 | 1183 | 2797 |
| Left entorhinal cortex | -0.060509 | 0.013434 | -4.50 | 6.86E-06 | <0.0001 | -0.1576 | 1164 | 2725 |
| Left fusiform gyrus | -0.050715 | 0.005289 | -9.59 | <2.00E-16 | <0.0001 | -0.3306 | 1195 | 2849 |
| Left inferior parietal cortex | -0.016516 | 0.004724 | -3.50 | 4.80E-04 | 0.0016 | -0.1213 | 1180 | 2831 |
| Left inferior temporal gyrus | -0.039540 | 0.005976 | -6.62 | 4.17E-11 | <0.0001 | -0.2306 | 1165 | 2823 |
| Left isthmus cingulate cortex | -0.026463 | 0.007161 | -3.70 | 2.20E-04 | 0.0009 | -0.1275 | 1195 | 2852 |
| Left lateral occipital cortex | -0.007458 | 0.004379 | -1.70 | 8.90E-02 | 0.1553 | -0.0587 | 1186 | 2853 |
| Left lateral orbitofrontal cortex | -0.021017 | 0.005899 | -3.56 | 3.70E-04 | 0.0013 | -0.1230 | 1188 | 2851 |
| Left lingual gyrus | -0.008806 | 0.004479 | -1.97 | 4.90E-02 | 0.0962 | -0.0680 | 1191 | 2837 |
| Left medial orbitofrontal cortex | -0.014788 | 0.005897 | -2.51 | 1.22E-02 | 0.0309 | -0.0870 | 1182 | 2818 |
| Left middle temporal gyrus | -0.039033 | 0.006043 | -6.46 | 1.18E-10 | <0.0001 | -0.2270 | 1149 | 2748 |
| Left parahippcampal gyrus | -0.035409 | 0.011493 | -3.08 | 2.08E-03 | 0.0060 | -0.1063 | 1190 | 2850 |
| Left paracentral lobule | -0.024221 | 0.005435 | -4.46 | 8.55E-06 | <0.0001 | -0.1537 | 1195 | 2857 |
| Left pars opercularis | -0.021441 | 0.005315 | -4.03 | 5.58E-05 | 0.0003 | -0.1390 | 1194 | 2845 |
| Left pars orbitalis | -0.018623 | 0.008071 | -2.31 | 2.11E-02 | 0.0473 | -0.0796 | 1194 | 2854 |
| Left pars triangularis | -0.007147 | 0.005792 | -1.23 | 2.17E-01 | 0.3121 | -0.0424 | 1193 | 2848 |
| Left pericalcarine | 0.004615 | 0.004876 | 0.95 | 3.44E-01 | 0.4400 | 0.0330 | 1180 | 2803 |
| Left precentral gyrus | -0.005010 | 0.004320 | -1.16 | 2.46E-01 | 0.3305 | -0.0402 | 1182 | 2829 |
| Left posterior cingulate cortex | -0.029690 | 0.005751 | -5.16 | 2.56E-07 | <0.0001 | -0.1778 | 1196 | 2857 |
| Left precentral gyrus | -0.029515 | 0.005044 | -5.85 | 5.27E-09 | <0.0001 | -0.2020 | 1192 | 2837 |
| Left precuneus | -0.017022 | 0.004653 | -3.66 | 2.60E-04 | 0.0010 | -0.1264 | 1189 | 2851 |
| Left rostral anterior cingulate cortex | -0.023447 | 0.008559 | -2.74 | 6.20E-03 | 0.0165 | -0.0947 | 1189 | 2835 |
| Left rostral middle frontal gyrus | -0.013895 | 0.004960 | -2.80 | 5.10E-03 | 0.0140 | -0.0965 | 1197 | 2848 |
| Left superior frontal gyrus | -0.022530 | 0.005173 | -4.35 | 1.36E-05 | 0.0001 | -0.1500 | 1194 | 2851 |
| Left superior parietal cortex | -0.010381 | 0.004259 | -2.44 | 1.50E-02 | 0.0350 | -0.0844 | 1187 | 2831 |
| Left superior temporal gyrus | -0.040383 | 0.005871 | -6.88 | 7.04E-12 | <0.0001 | -0.2434 | 1138 | 2684 |
| Left supramarginal gyrus | -0.020501 | 0.004944 | -4.15 | 3.45E-05 | 0.0002 | -0.1446 | 1173 | 2767 |
| Left frontal pole | -0.027743 | 0.011142 | -2.49 | 1.28E-02 | 0.0313 | -0.0857 | 1199 | 2863 |
| Left temporal pole | -0.059265 | 0.013522 | -4.38 | 1.20E-05 | 0.0001 | -0.1513 | 1187 | 2851 |
| Left transverse temporal gyrus | -0.042329 | 0.007996 | -5.29 | 1.26E-07 | <0.0001 | -0.1823 | 1195 | 2853 |
| Left insula | -0.030499 | 0.005895 | -5.17 | 2.41E-07 | <0.0001 | -0.1790 | 1188 | 2811 |
| Right banks of the superior temporal sulcus | -0.029565 | 0.006388 | -4.63 | 3.81E-06 | <0.0001 | -0.1609 | 1178 | 2796 |
| Right caudal anterior cingulate cortex | -0.015619 | 0.008283 | -1.89 | 5.94E-02 | 0.1110 | -0.0651 | 1198 | 2852 |
| Right caudal middle frontal gyrus | -0.019566 | 0.005248 | -3.73 | 2.00E-04 | 0.0008 | -0.1287 | 1194 | 2845 |
| Right cuneus | -0.005411 | 0.004994 | -1.08 | 2.80E-01 | 0.3694 | -0.0375 | 1179 | 2809 |
| Right entorhinal cortex | -0.032346 | 0.014339 | -2.26 | 2.41E-02 | 0.0525 | -0.0794 | 1162 | 2686 |
| Right fusiform gyrus | -0.050063 | 0.005312 | -9.42 | <2.00E-16 | <0.0001 | -0.3249 | 1193 | 2849 |
| Right inferior parietal cortex | -0.010510 | 0.004667 | -2.25 | 2.44E-02 | 0.0525 | -0.0778 | 1187 | 2826 |
| Right inferior temporal gyrus | -0.036217 | 0.006163 | -5.88 | 4.54E-09 | <0.0001 | -0.2040 | 1175 | 2838 |
| Right isthmus cingulate cortex | -0.022097 | 0.006953 | -3.18 | 1.50E-03 | 0.0045 | -0.1096 | 1196 | 2854 |
| Right lateral occipital cortex | -0.005438 | 0.004557 | -1.19 | 2.30E-01 | 0.3224 | -0.0410 | 1193 | 2848 |
| Right lateral orbitofrontal cortex | -0.021590 | 0.005867 | -3.68 | 2.40E-04 | 0.0009 | -0.1268 | 1195 | 2858 |
| Right lingual gyrus | -0.008744 | 0.004504 | -1.94 | 5.20E-02 | 0.0996 | -0.0672 | 1182 | 2834 |
| Right medial orbitofrontal cortex | -0.031300 | 0.006112 | -5.12 | 3.18E-07 | <0.0001 | -0.1773 | 1183 | 2831 |
| Right middle temporal gyrus | -0.036010 | 0.005940 | -6.06 | 1.49E-09 | <0.0001 | -0.2100 | 1184 | 2815 |
| Right parahippcampal gyrus | -0.043909 | 0.009850 | -4.46 | 8.50E-06 | <0.0001 | -0.1539 | 1192 | 2850 |
| Right paracentral lobule | -0.021644 | 0.005497 | -3.94 | 8.38E-05 | 0.0004 | -0.1357 | 1196 | 2857 |
| Right pars opercularis | -0.033003 | 0.005535 | -5.96 | 2.70E-09 | <0.0001 | -0.2060 | 1189 | 2835 |
| Right pars orbitalis | -0.019736 | 0.007904 | -2.50 | 1.26E-02 | 0.0313 | -0.0861 | 1198 | 2848 |
| Right pars triangularis | -0.019566 | 0.005780 | -3.39 | 7.20E-04 | 0.0023 | -0.1172 | 1187 | 2838 |
| Right pericalcarine | -0.002402 | 0.004946 | -0.49 | 6.30E-01 | 0.6917 | -0.0170 | 1177 | 2803 |
| Right precentral gyrus | -0.004693 | 0.004412 | -1.06 | 2.88E-01 | 0.3768 | -0.0366 | 1187 | 2842 |
| Right posterior cingulate cortex | -0.032964 | 0.005529 | -5.96 | 2.71E-09 | <0.0001 | -0.2053 | 1196 | 2859 |
| Right precentral gyrus | -0.029649 | 0.005149 | -5.76 | 9.13E-09 | <0.0001 | -0.1990 | 1188 | 2844 |
| Right precuneus | -0.018946 | 0.004591 | -4.13 | 3.75E-05 | 0.0002 | -0.1424 | 1195 | 2848 |
| Right rostral anterior cingulate cortex | -0.017120 | 0.008360 | -2.05 | 4.08E-02 | 0.0843 | -0.0707 | 1194 | 2836 |
| Right rostral middle frontal gyrus | -0.017394 | 0.004921 | -3.53 | 4.10E-04 | 0.0014 | -0.1218 | 1192 | 2849 |
| Right superior frontal gyrus | -0.029640 | 0.005140 | -5.76 | 8.93E-09 | <0.0001 | -0.1988 | 1189 | 2859 |
| Right superior parietal cortex | -0.002285 | 0.004249 | -0.54 | 5.91E-01 | 0.6673 | -0.0186 | 1195 | 2842 |
| Right superior temporal gyrus | -0.041290 | 0.005761 | -7.17 | 9.09E-13 | <0.0001 | -0.2511 | 1161 | 2745 |
| Right supramarginal gyrus | -0.015713 | 0.004946 | -3.18 | 1.50E-03 | 0.0045 | -0.1106 | 1178 | 2780 |
| Right frontal pole | -0.012807 | 0.010907 | -1.17 | 2.40E-01 | 0.3292 | -0.0402 | 1200 | 2861 |
| Right temporal pole | -0.050004 | 0.013957 | -3.58 | 3.40E-04 | 0.0012 | -0.1236 | 1191 | 2850 |
| Right transverse temporal gyrus | -0.025266 | 0.008282 | -3.05 | 2.30E-03 | 0.0066 | -0.1053 | 1190 | 2849 |
| Right insula | -0.021532 | 0.006215 | -3.46 | 5.40E-04 | 0.0018 | -0.1202 | 1182 | 2777 |
|  |  |  |  |  |  |  |  |  |
| **Subcortical volumes** |  |  |  |  |  |  |  |  |
| Left lateral ventricle | 365.900484 | 162.243069 | 2.26 | 2.42E-02 | 0.0525 | 0.0799 | 1138 | 2710 |
| Right lateral ventricle | 329.876349 | 151.890783 | 2.17 | 2.99E-02 | 0.0627 | 0.0769 | 1130 | 2706 |
| Left thalamus | 108.694501 | 26.117337 | 4.16 | 3.23E-05 | 0.0002 | 0.1471 | 1138 | 2691 |
| Right thalamus | 80.814421 | 22.216216 | 3.64 | 2.80E-04 | 0.0010 | 0.1290 | 1134 | 2680 |
| Left caudate | 10.320382 | 15.274489 | 0.68 | 4.99E-01 | 0.5894 | 0.0240 | 1151 | 2684 |
| Right caudate | 7.023465 | 15.377368 | 0.46 | 6.48E-01 | 0.6990 | 0.0163 | 1128 | 2674 |
| Left putamen | 46.894181 | 21.069401 | 2.23 | 2.61E-02 | 0.0554 | 0.0805 | 1089 | 2613 |
| Right putamen | 25.948110 | 19.404370 | 1.34 | 1.81E-01 | 0.2710 | 0.0480 | 1107 | 2649 |
| Left pallidum | -12.112149 | 7.880550 | -1.54 | 1.24E-01 | 0.2034 | -0.0559 | 1080 | 2563 |
| Right pallidum | -2.834755 | 6.862186 | -0.41 | 6.80E-01 | 0.7209 | -0.0146 | 1129 | 2678 |
| Left hippocampus | 16.348108 | 13.803886 | 1.18 | 2.36E-01 | 0.3284 | 0.0419 | 1127 | 2683 |
| Right hippocamups | 33.556619 | 13.809830 | 2.43 | 1.52E-02 | 0.0350 | 0.0859 | 1136 | 2709 |
| Left amygdala | 22.444069 | 6.474463 | 3.47 | 5.30E-04 | 0.0018 | 0.1231 | 1127 | 2694 |
| Right amygdala | 41.656392 | 6.983922 | 5.96 | 2.68E-09 | <0.0001 | 0.2113 | 1129 | 2702 |
| Left nucleus accumbens | 11.723700 | 3.541291 | 3.31 | 9.40E-04 | 0.0030 | 0.1183 | 1110 | 2660 |
| Right nucleus accumbens | -0.554827 | 3.227906 | -0.17 | 8.64E-01 | 0.8811 | -0.0061 | 1116 | 2657 |
|  |  |  |  |  |  |  |  |  |
| **Cortical surface area** |  |  |  |  |  |  |  |  |
| Left banks of the superior temporal sulcus | -6.965510 | 5.661702 | -1.23 | 2.19E-01 | 0.3121 | -0.0451 | 1052 | 2527 |
| Left caudal anterior cingulate cortex | 1.386969 | 5.112727 | 0.27 | 7.86E-01 | 0.8122 | 0.0096 | 1120 | 2687 |
| Left caudal middle frontal gyrus | -0.743680 | 12.741721 | -0.06 | 9.54E-01 | 0.9535 | -0.0021 | 1123 | 2678 |
| Left cuneus | -4.559617 | 7.548955 | -0.60 | 5.46E-01 | 0.6338 | -0.0214 | 1115 | 2644 |
| Left entorhinal cortex | -0.788449 | 2.908055 | -0.27 | 7.86E-01 | 0.8122 | -0.0099 | 1046 | 2513 |
| Left fusiform gyrus | -25.479234 | 12.841094 | -1.98 | 4.73E-02 | 0.0940 | -0.0711 | 1097 | 2653 |
| Left inferior parietal cortex | -31.427650 | 20.327002 | -1.55 | 1.22E-01 | 0.2019 | -0.0553 | 1115 | 2663 |
| Left inferior temporal gyrus | -40.910059 | 15.209251 | -2.69 | 7.18E-03 | 0.0188 | -0.0964 | 1099 | 2673 |
| Left isthmus cingulate cortex | 25.899777 | 5.492321 | 4.72 | 2.50E-06 | <0.0001 | 0.1671 | 1134 | 2700 |
| Left lateral occipital cortex | -16.923857 | 19.461258 | -0.87 | 3.85E-01 | 0.4792 | -0.0309 | 1127 | 2701 |
| Left lateral orbitofrontal cortex | 11.430561 | 9.096922 | 1.26 | 2.10E-01 | 0.3053 | 0.0445 | 1139 | 2708 |
| Left lingual gyrus | -32.433630 | 13.793410 | -2.35 | 1.88E-02 | 0.0428 | -0.0834 | 1128 | 2692 |
| Left medial orbitofrontal cortex | 0.484625 | 7.856466 | 0.06 | 9.51E-01 | 0.9535 | 0.0021 | 1107 | 2650 |
| Left middle temporal gyrus | -23.120706 | 12.786532 | -1.81 | 7.07E-02 | 0.1290 | -0.0660 | 1064 | 2578 |
| Left parahippcampal gyrus | 2.698774 | 3.330071 | 0.81 | 4.18E-01 | 0.5084 | 0.0289 | 1114 | 2682 |
| Left paracentral lobule | 16.022848 | 6.588988 | 2.43 | 1.51E-02 | 0.0350 | 0.0872 | 1099 | 2658 |
| Left pars opercularis | -3.091651 | 8.888063 | -0.35 | 7.28E-01 | 0.7671 | -0.0125 | 1120 | 2687 |
| Left pars orbitalis | -1.556344 | 2.772170 | -0.56 | 5.75E-01 | 0.6537 | -0.0198 | 1139 | 2703 |
| Left pars triangularis | -1.180120 | 6.901739 | -0.17 | 8.64E-01 | 0.8811 | -0.0060 | 1134 | 2692 |
| Left pericalcarine | -12.264448 | 8.331347 | -1.47 | 1.41E-01 | 0.2216 | -0.0523 | 1124 | 2654 |
| Left precentral gyrus | 6.602008 | 14.398379 | 0.46 | 6.47E-01 | 0.6990 | 0.0165 | 1094 | 2658 |
| Left posterior cingulate cortex | 8.471467 | 6.095981 | 1.39 | 1.65E-01 | 0.2511 | 0.0492 | 1135 | 2702 |
| Left precentral gyrus | 11.796923 | 16.419622 | 0.72 | 4.73E-01 | 0.5656 | 0.0257 | 1117 | 2674 |
| Left precuneus | 21.619871 | 14.381359 | 1.50 | 1.33E-01 | 0.2153 | 0.0532 | 1130 | 2697 |
| Left rostral anterior cingulate cortex | 7.226503 | 5.146764 | 1.40 | 1.60E-01 | 0.2469 | 0.0503 | 1097 | 2655 |
| Left rostral middle frontal gyrus | -39.644719 | 22.507417 | -1.76 | 7.83E-02 | 0.1409 | -0.0623 | 1135 | 2694 |
| Left superior frontal gyrus | 42.510659 | 24.194596 | 1.76 | 7.90E-02 | 0.1409 | 0.0629 | 1106 | 2672 |
| Left superior parietal cortex | 19.543282 | 20.679942 | 0.95 | 3.45E-01 | 0.4400 | 0.0339 | 1111 | 2668 |
| Left superior temporal gyrus | 16.422933 | 14.034262 | 1.17 | 2.42E-01 | 0.3292 | 0.0428 | 1064 | 2514 |
| Left supramarginal gyrus | 29.680971 | 18.016300 | 1.65 | 9.96E-02 | 0.1700 | 0.0598 | 1082 | 2572 |
| Left frontal pole | 1.827656 | 1.251138 | 1.46 | 1.44E-01 | 0.2241 | 0.0515 | 1141 | 2711 |
| Left temporal pole | -1.106090 | 2.147302 | -0.52 | 6.07E-01 | 0.6753 | -0.0184 | 1134 | 2698 |
| Left transverse temporal gyrus | 10.183081 | 2.603108 | 3.91 | 9.32E-05 | 0.0004 | 0.1380 | 1141 | 2708 |
| Left insula | 14.361908 | 7.804346 | 1.84 | 6.58E-02 | 0.1216 | 0.0654 | 1127 | 2660 |
| Right banks of the superior temporal sulcus | -2.394744 | 4.771208 | -0.50 | 6.16E-01 | 0.6808 | -0.0179 | 1106 | 2632 |
| Right caudal anterior cingulate cortex | 7.177138 | 5.471283 | 1.31 | 1.90E-01 | 0.2809 | 0.0466 | 1119 | 2693 |
| Right caudal middle frontal gyrus | -9.090738 | 12.738152 | -0.71 | 4.76E-01 | 0.5656 | -0.0252 | 1123 | 2685 |
| Right cuneus | -8.019912 | 7.088857 | -1.13 | 2.58E-01 | 0.3432 | -0.0403 | 1115 | 2654 |
| Right entorhinal cortex | -2.213486 | 2.960118 | -0.75 | 4.55E-01 | 0.5491 | -0.0278 | 1036 | 2477 |
| Right fusiform gyrus | -7.360900 | 12.477411 | -0.59 | 5.55E-01 | 0.6363 | -0.0212 | 1089 | 2665 |
| Right inferior parietal cortex | -36.013348 | 22.224580 | -1.62 | 1.05E-01 | 0.1776 | -0.0578 | 1118 | 2652 |
| Right inferior temporal gyrus | -35.139819 | 14.135573 | -2.49 | 1.30E-02 | 0.0313 | -0.0887 | 1115 | 2684 |
| Right isthmus cingulate cortex | 21.159800 | 5.096805 | 4.15 | 3.37E-05 | 0.0002 | 0.1467 | 1137 | 2706 |
| Right lateral occipital cortex | -11.787840 | 19.355496 | -0.61 | 5.43E-01 | 0.6338 | -0.0216 | 1131 | 2701 |
| Right lateral orbitofrontal cortex | 8.227716 | 9.829713 | 0.84 | 4.03E-01 | 0.4977 | 0.0297 | 1140 | 2709 |
| Right lingual gyrus | -27.523652 | 13.690582 | -2.01 | 4.45E-02 | 0.0895 | -0.0713 | 1127 | 2688 |
| Right medial orbitofrontal cortex | 3.013548 | 6.836985 | 0.44 | 6.59E-01 | 0.7038 | 0.0157 | 1116 | 2679 |
| Right middle temporal gyrus | -11.718412 | 12.844896 | -0.91 | 3.62E-01 | 0.4543 | -0.0325 | 1114 | 2644 |
| Right parahippcampal gyrus | 0.978461 | 3.312839 | 0.30 | 7.68E-01 | 0.8036 | 0.0107 | 1116 | 2685 |
| Right paracentral lobule | 21.588547 | 7.403385 | 2.92 | 3.57E-03 | 0.0100 | 0.1040 | 1117 | 2688 |
| Right pars opercularis | 6.688585 | 8.179631 | 0.82 | 4.14E-01 | 0.5073 | 0.0292 | 1117 | 2671 |
| Right pars orbitalis | -3.423715 | 3.297050 | -1.04 | 2.99E-01 | 0.3881 | -0.0368 | 1139 | 2696 |
| Right pars triangularis | -9.440515 | 8.089021 | -1.17 | 2.43E-01 | 0.3292 | -0.0416 | 1124 | 2677 |
| Right pericalcarine | -11.581469 | 8.588355 | -1.35 | 1.78E-01 | 0.2681 | -0.0480 | 1128 | 2653 |
| Right precentral gyrus | 8.593024 | 14.338372 | 0.60 | 5.49E-01 | 0.6338 | 0.0214 | 1115 | 2679 |
| Right posterior cingulate cortex | 11.570834 | 5.904125 | 1.96 | 5.01E-02 | 0.0971 | 0.0693 | 1134 | 2707 |
| Right precentral gyrus | -9.015771 | 17.038990 | -0.53 | 5.97E-01 | 0.6692 | -0.0189 | 1111 | 2678 |
| Right precuneus | 19.591776 | 15.037080 | 1.30 | 1.93E-01 | 0.2827 | 0.0460 | 1135 | 2698 |
| Right rostral anterior cingulate cortex | 8.051825 | 4.732591 | 1.70 | 8.90E-02 | 0.1553 | 0.0606 | 1115 | 2679 |
| Right rostral middle frontal gyrus | -71.936197 | 22.907571 | -3.14 | 1.70E-03 | 0.0050 | -0.1111 | 1135 | 2698 |
| Right superior frontal gyrus | 49.516244 | 24.325938 | 2.04 | 4.19E-02 | 0.0854 | 0.0728 | 1110 | 2686 |
| Right superior parietal cortex | 2.635044 | 20.025557 | 0.13 | 8.95E-01 | 0.9065 | 0.0046 | 1125 | 2679 |
| Right superior temporal gyrus | 18.207262 | 12.370360 | 1.47 | 1.41E-01 | 0.2216 | 0.0531 | 1091 | 2572 |
| Right supramarginal gyrus | 20.424606 | 16.680233 | 1.22 | 2.21E-01 | 0.3124 | 0.0438 | 1104 | 2604 |
| Right frontal pole | -1.425171 | 1.559168 | -0.91 | 3.61E-01 | 0.4543 | -0.0321 | 1140 | 2710 |
| Right temporal pole | 3.435361 | 2.297118 | 1.50 | 1.35E-01 | 0.2161 | 0.0536 | 1106 | 2686 |
| Right transverse temporal gyrus | 3.788124 | 2.003686 | 1.89 | 5.90E-02 | 0.1110 | 0.0667 | 1141 | 2712 |
| Right insula | 14.622575 | 8.849120 | 1.65 | 9.85E-02 | 0.1700 | 0.0589 | 1121 | 2626 |

| **Label** | **Estimate** | **StdError** | **T** | **p** | **FDR adjusted p** | **Cohen´s d** | **N** |
| --- | --- | --- | --- | --- | --- | --- | --- |
|  |  |  |  |  |  |  |  |
| **Global measures** |  |  |  |  |  |  |  |
| Total Intracranial Volume | -1562.00000 | 412.000000 | -3.79 | 1.50E-04 | 0.0004 | -0.0977 | 6052 |
| Total left hemispheral surface area | -34.650000 | 19.070000 | -1.82 | 6.93E-02 | 0.1062 | -0.0463 | 6227 |
| Total right hemispheral surface area | -37.500000 | 19.100000 | -1.96 | 4.97E-02 | 0.0789 | -0.0498 | 6227 |
| Left hemispheral average thickness | -0.001730 | 0.000248 | -6.98 | 3.20E-12 | <0.0001 | -0.1771 | 6246 |
| Right hemispheral average thickness | -0.001623 | 0.000247 | -6.56 | 5.65E-11 | <0.0001 | -0.1665 | 6245 |
|  |  |  |  |  |  |  |  |
| **Cortical thickness** |  |  |  |  |  |  |  |
| Left banks of the superior temporal sulcus | -0.002952 | 0.000471 | -6.27 | 3.94E-10 | <0.0001 | -0.1635 | 5918 |
| Left caudal anterior cingulate cortex | -0.001814 | 0.000652 | -2.78 | 5.43E-03 | 0.0114 | -0.0707 | 6225 |
| Left caudal middle frontal gyrus | -0.002036 | 0.000393 | -5.18 | 2.30E-07 | <0.0001 | -0.1319 | 6203 |
| Left cuneus | -0.000424 | 0.000362 | -1.17 | 2.41E-01 | 0.3027 | -0.0300 | 6135 |
| Left entorhinal cortex | -0.005518 | 0.000982 | -5.62 | 2.03E-08 | <0.0001 | -0.1454 | 6009 |
| Left fusiform gyrus | -0.004060 | 0.000389 | -10.43 | <2.00E-16 | <0.0001 | -0.2653 | 6217 |
| Left inferior parietal cortex | -0.001471 | 0.000352 | -4.18 | 2.94E-05 | 0.0001 | -0.1068 | 6160 |
| Left inferior temporal gyrus | -0.002986 | 0.000442 | -6.75 | 1.58E-11 | <0.0001 | -0.1728 | 6137 |
| Left isthmus cingulate cortex | -0.002436 | 0.000527 | -4.62 | 3.87E-06 | <0.0001 | -0.1174 | 6224 |
| Left lateral occipital cortex | -0.000606 | 0.000321 | -1.89 | 5.90E-02 | 0.0917 | -0.0481 | 6208 |
| Left lateral orbitofrontal cortex | -0.001508 | 0.000435 | -3.47 | 5.30E-04 | 0.0014 | -0.0883 | 6207 |
| Left lingual gyrus | -0.000712 | 0.000326 | -2.18 | 2.90E-02 | 0.0490 | -0.0555 | 6195 |
| Left medial orbitofrontal cortex | -0.001685 | 0.000435 | -3.87 | 1.10E-04 | 0.0003 | -0.0989 | 6154 |
| Left middle temporal gyrus | -0.003268 | 0.000448 | -7.29 | 3.60E-13 | <0.0001 | -0.1887 | 6007 |
| Left parahippcampal gyrus | -0.003340 | 0.000843 | -3.96 | 7.47E-05 | 0.0002 | -0.1007 | 6216 |
| Left paracentral lobule | -0.001856 | 0.000402 | -4.61 | 4.09E-06 | <0.0001 | -0.1172 | 6222 |
| Left pars opercularis | -0.001975 | 0.000392 | -5.03 | 4.95E-07 | <0.0001 | -0.1281 | 6204 |
| Left pars orbitalis | -0.001370 | 0.000594 | -2.31 | 2.10E-02 | 0.0375 | -0.0587 | 6219 |
| Left pars triangularis | -0.000570 | 0.000426 | -1.34 | 1.81E-01 | 0.2444 | -0.0341 | 6208 |
| Left pericalcarine | 0.000460 | 0.000353 | 1.31 | 1.92E-01 | 0.2575 | 0.0336 | 6130 |
| Left precentral gyrus | -0.000093 | 0.000319 | -0.29 | 7.71E-01 | 0.8020 | -0.0074 | 6165 |
| Left posterior cingulate cortex | -0.002665 | 0.000425 | -6.26 | 4.00E-10 | <0.0001 | -0.1591 | 6227 |
| Left precentral gyrus | -0.002120 | 0.000372 | -5.70 | 1.28E-08 | <0.0001 | -0.1453 | 6186 |
| Left precuneus | -0.001537 | 0.000342 | -4.49 | 7.30E-06 | <0.0001 | -0.1142 | 6212 |
| Left rostral anterior cingulate cortex | -0.003292 | 0.000625 | -5.27 | 1.44E-07 | <0.0001 | -0.1343 | 6195 |
| Left rostral middle frontal gyrus | -0.001291 | 0.000365 | -3.53 | 4.10E-04 | 0.0012 | -0.0898 | 6216 |
| Left superior frontal gyrus | -0.002026 | 0.000382 | -5.31 | 1.14E-07 | <0.0001 | -0.1351 | 6212 |
| Left superior parietal cortex | -0.000995 | 0.000314 | -3.17 | 1.50E-03 | 0.0036 | -0.0809 | 6179 |
| Left superior temporal gyrus | -0.003036 | 0.000434 | -6.99 | 3.00E-12 | <0.0001 | -0.1827 | 5890 |
| Left supramarginal gyrus | -0.001677 | 0.000365 | -4.59 | 4.51E-06 | <0.0001 | -0.1182 | 6069 |
| Left frontal pole | -0.002374 | 0.000812 | -2.92 | 3.50E-03 | 0.0080 | -0.0741 | 6240 |
| Left temporal pole | -0.004065 | 0.000993 | -4.09 | 4.33E-05 | 0.0002 | -0.1041 | 6204 |
| Left transverse temporal gyrus | -0.003676 | 0.000586 | -6.27 | 3.85E-10 | <0.0001 | -0.1595 | 6216 |
| Left insula | -0.002386 | 0.000433 | -5.51 | 3.76E-08 | <0.0001 | -0.1408 | 6157 |
| Right banks of the superior temporal sulcus | -0.002264 | 0.000467 | -4.85 | 1.30E-06 | <0.0001 | -0.1244 | 6110 |
| Right caudal anterior cingulate cortex | -0.001895 | 0.000605 | -3.13 | 1.74E-03 | 0.0041 | -0.0795 | 6228 |
| Right caudal middle frontal gyrus | -0.001360 | 0.000392 | -3.47 | 5.20E-04 | 0.0014 | -0.0883 | 6211 |
| Right cuneus | -0.000600 | 0.000363 | -1.65 | 9.84E-02 | 0.1392 | -0.0422 | 6139 |
| Right entorhinal cortex | -0.003474 | 0.001059 | -3.28 | 1.05E-03 | 0.0026 | -0.0853 | 5953 |
| Right fusiform gyrus | -0.004097 | 0.000394 | -10.40 | <2.00E-16 | <0.0001 | -0.2644 | 6222 |
| Right inferior parietal cortex | -0.000976 | 0.000343 | -2.85 | 4.44E-03 | 0.0097 | -0.0728 | 6165 |
| Right inferior temporal gyrus | -0.003206 | 0.000451 | -7.10 | 1.30E-12 | <0.0001 | -0.1813 | 6169 |
| Right isthmus cingulate cortex | -0.002011 | 0.000514 | -3.91 | 9.34E-05 | 0.0003 | -0.0994 | 6224 |
| Right lateral occipital cortex | -0.000416 | 0.000333 | -1.25 | 2.10E-01 | 0.2702 | -0.0318 | 6211 |
| Right lateral orbitofrontal cortex | -0.001846 | 0.000433 | -4.27 | 2.02E-05 | 0.0001 | -0.1086 | 6223 |
| Right lingual gyrus | -0.000419 | 0.000328 | -1.28 | 2.01E-01 | 0.2652 | -0.0327 | 6179 |
| Right medial orbitofrontal cortex | -0.002680 | 0.000450 | -5.96 | 2.68E-09 | <0.0001 | -0.1520 | 6180 |
| Right middle temporal gyrus | -0.002952 | 0.000435 | -6.78 | 1.28E-11 | <0.0001 | -0.1733 | 6153 |
| Right parahippcampal gyrus | -0.003735 | 0.000722 | -5.17 | 2.37E-07 | <0.0001 | -0.1315 | 6221 |
| Right paracentral lobule | -0.001623 | 0.000403 | -4.03 | 5.59E-05 | 0.0002 | -0.1024 | 6226 |
| Right pars opercularis | -0.002106 | 0.000411 | -5.13 | 3.06E-07 | <0.0001 | -0.1309 | 6182 |
| Right pars orbitalis | -0.001815 | 0.000582 | -3.12 | 1.80E-03 | 0.0042 | -0.0794 | 6217 |
| Right pars triangularis | -0.001376 | 0.000418 | -3.29 | 9.90E-04 | 0.0025 | -0.0839 | 6181 |
| Right pericalcarine | 0.000093 | 0.000354 | 0.26 | 7.92E-01 | 0.8074 | 0.0067 | 6112 |
| Right precentral gyrus | -0.000188 | 0.000321 | -0.58 | 5.59E-01 | 0.6269 | -0.0148 | 6185 |
| Right posterior cingulate cortex | -0.002991 | 0.000413 | -7.24 | 5.07E-13 | <0.0001 | -0.1840 | 6230 |
| Right precentral gyrus | -0.002073 | 0.000376 | -5.52 | 3.60E-08 | <0.0001 | -0.1407 | 6194 |
| Right precuneus | -0.001757 | 0.000339 | -5.18 | 2.20E-07 | <0.0001 | -0.1318 | 6214 |
| Right rostral anterior cingulate cortex | -0.002435 | 0.000614 | -3.97 | 7.35E-05 | 0.0002 | -0.1011 | 6198 |
| Right rostral middle frontal gyrus | -0.001583 | 0.000364 | -4.35 | 1.37E-05 | 0.0001 | -0.1107 | 6209 |
| Right superior frontal gyrus | -0.002503 | 0.000381 | -6.58 | 5.30E-11 | <0.0001 | -0.1674 | 6217 |
| Right superior parietal cortex | -0.000362 | 0.000313 | -1.16 | 2.48E-01 | 0.3090 | -0.0296 | 6196 |
| Right superior temporal gyrus | -0.002863 | 0.000425 | -6.73 | 1.82E-11 | <0.0001 | -0.1739 | 6025 |
| Right supramarginal gyrus | -0.001319 | 0.000366 | -3.60 | 3.20E-04 | 0.0009 | -0.0925 | 6087 |
| Right frontal pole | -0.002009 | 0.000802 | -2.51 | 1.22E-02 | 0.0226 | -0.0637 | 6236 |
| Right temporal pole | -0.003614 | 0.001030 | -3.51 | 4.50E-04 | 0.0013 | -0.0893 | 6209 |
| Right transverse temporal gyrus | -0.002614 | 0.000616 | -4.24 | 2.20E-05 | 0.0001 | -0.1079 | 6207 |
| Right insula | -0.001853 | 0.000460 | -4.03 | 5.67E-05 | 0.0002 | -0.1034 | 6105 |
|  |  |  |  |  |  |  |  |
| **Subcortical volumes** |  |  |  |  |  |  |  |
| Left lateral ventricle | 13.248154 | 12.661627 | 1.05 | 2.95E-01 | 0.3596 | 0.0273 | 5954 |
| Right lateral ventricle | 20.876397 | 11.747847 | 1.78 | 7.56E-02 | 0.1109 | 0.0463 | 5939 |
| Left thalamus | 13.503112 | 1.927691 | 7.00 | 2.75E-12 | <0.0001 | 0.1823 | 5934 |
| Right thalamus | 8.224585 | 1.664508 | 4.94 | 7.98E-07 | <0.0001 | 0.1288 | 5920 |
| Left caudate | 0.602293 | 1.129585 | 0.53 | 5.94E-01 | 0.6540 | 0.0138 | 5931 |
| Right caudate | 0.605713 | 1.141397 | 0.53 | 5.96E-01 | 0.6540 | 0.0139 | 5877 |
| Left putamen | 4.187568 | 1.546374 | 2.71 | 6.79E-03 | 0.0137 | 0.0718 | 5736 |
| Right putamen | 3.718655 | 1.436088 | 2.59 | 9.64E-03 | 0.0189 | 0.0682 | 5805 |
| Left pallidum | -1.440011 | 0.584442 | -2.46 | 1.38E-02 | 0.0251 | -0.0656 | 5667 |
| Right pallidum | -0.399432 | 0.520869 | -0.77 | 4.43E-01 | 0.5154 | -0.0201 | 5888 |
| Left hippocampus | 3.470055 | 1.034346 | 3.35 | 8.00E-04 | 0.0021 | 0.0874 | 5906 |
| Right hippocamups | 4.240650 | 1.028673 | 4.12 | 3.80E-05 | 0.0001 | 0.1071 | 5957 |
| Left amygdala | 2.734364 | 0.486064 | 5.63 | 1.93E-08 | <0.0001 | 0.1468 | 5920 |
| Right amygdala | 4.505177 | 0.516411 | 8.72 | <2.00E-16 | <0.0001 | 0.2271 | 5930 |
| Left nucleus accumbens | 0.846976 | 0.260238 | 3.25 | 1.14E-03 | 0.0028 | 0.0855 | 5819 |
| Right nucleus accumbens | 0.070277 | 0.238225 | 0.30 | 7.68E-01 | 0.8020 | 0.0079 | 5841 |
|  |  |  |  |  |  |  |  |
| **Cortical surface area** |  |  |  |  |  |  |  |
| Left banks of the superior temporal sulcus | -0.853281 | 0.429326 | -1.99 | 4.69E-02 | 0.0759 | -0.0524 | 5795 |
| Left caudal anterior cingulate cortex | 0.106468 | 0.376959 | 0.28 | 7.78E-01 | 0.8032 | 0.0072 | 6133 |
| Left caudal middle frontal gyrus | 0.565599 | 0.945724 | 0.60 | 5.50E-01 | 0.6210 | 0.0154 | 6131 |
| Left cuneus | -0.544892 | 0.563013 | -0.97 | 3.33E-01 | 0.4024 | -0.0249 | 6083 |
| Left entorhinal cortex | 0.069053 | 0.216201 | 0.32 | 7.49E-01 | 0.7950 | 0.0084 | 5779 |
| Left fusiform gyrus | -2.628387 | 0.947575 | -2.77 | 5.56E-03 | 0.0115 | -0.0716 | 6029 |
| Left inferior parietal cortex | -2.540188 | 1.526184 | -1.66 | 9.61E-02 | 0.1371 | -0.0427 | 6081 |
| Left inferior temporal gyrus | -2.869084 | 1.128549 | -2.54 | 1.10E-02 | 0.0206 | -0.0652 | 6110 |
| Left isthmus cingulate cortex | 2.475908 | 0.413239 | 5.99 | 2.20E-09 | <0.0001 | 0.1527 | 6188 |
| Left lateral occipital cortex | -2.593336 | 1.437085 | -1.80 | 7.12E-02 | 0.1065 | -0.0459 | 6173 |
| Left lateral orbitofrontal cortex | 0.930144 | 0.668991 | 1.39 | 1.65E-01 | 0.2246 | 0.0354 | 6208 |
| Left lingual gyrus | -3.045086 | 1.031987 | -2.95 | 3.20E-03 | 0.0074 | -0.0753 | 6175 |
| Left medial orbitofrontal cortex | 0.401355 | 0.581394 | 0.69 | 4.90E-01 | 0.5615 | 0.0178 | 6065 |
| Left middle temporal gyrus | -1.244640 | 0.962820 | -1.29 | 1.96E-01 | 0.2610 | -0.0337 | 5912 |
| Left parahippcampal gyrus | 0.422322 | 0.240256 | 1.76 | 7.88E-02 | 0.1135 | 0.0451 | 6128 |
| Left paracentral lobule | 1.655861 | 0.491077 | 3.37 | 7.50E-04 | 0.0020 | 0.0867 | 6071 |
| Left pars opercularis | 0.268778 | 0.671842 | 0.40 | 6.89E-01 | 0.7360 | 0.0102 | 6148 |
| Left pars orbitalis | -0.002547 | 0.209287 | -0.01 | 9.90E-01 | 0.9900 | -0.0003 | 6194 |
| Left pars triangularis | -0.017578 | 0.514729 | -0.03 | 9.73E-01 | 0.9790 | -0.0008 | 6171 |
| Left pericalcarine | -1.619042 | 0.626145 | -2.59 | 9.74E-03 | 0.0189 | -0.0664 | 6118 |
| Left precentral gyrus | 1.251463 | 1.066772 | 1.17 | 2.41E-01 | 0.3027 | 0.0301 | 6065 |
| Left posterior cingulate cortex | 1.295514 | 0.449950 | 2.88 | 4.00E-03 | 0.0088 | 0.0734 | 6195 |
| Left precentral gyrus | 2.209942 | 1.220482 | 1.81 | 7.02E-02 | 0.1062 | 0.0465 | 6102 |
| Left precuneus | 2.255820 | 1.055390 | 2.14 | 3.30E-02 | 0.0545 | 0.0546 | 6181 |
| Left rostral anterior cingulate cortex | 1.028199 | 0.384683 | 2.67 | 7.54E-03 | 0.0150 | 0.0689 | 6036 |
| Left rostral middle frontal gyrus | -2.123464 | 1.670215 | -1.27 | 2.04E-01 | 0.2664 | -0.0324 | 6182 |
| Left superior frontal gyrus | 4.528321 | 1.778598 | 2.55 | 1.09E-02 | 0.0206 | 0.0656 | 6086 |
| Left superior parietal cortex | 2.949564 | 1.545157 | 1.91 | 5.60E-02 | 0.0879 | 0.0490 | 6105 |
| Left superior temporal gyrus | 1.858055 | 1.026451 | 1.81 | 7.03E-02 | 0.1062 | 0.0476 | 5807 |
| Left supramarginal gyrus | 2.732236 | 1.333538 | 2.05 | 4.05E-02 | 0.0662 | 0.0534 | 5931 |
| Left frontal pole | 0.140051 | 0.092922 | 1.51 | 1.32E-01 | 0.1831 | 0.0384 | 6222 |
| Left temporal pole | -0.043356 | 0.160602 | -0.27 | 7.87E-01 | 0.8074 | -0.0069 | 6187 |
| Left transverse temporal gyrus | 0.883021 | 0.194217 | 4.55 | 5.56E-06 | <0.0001 | 0.1157 | 6216 |
| Left insula | 1.625397 | 0.584601 | 2.78 | 5.45E-03 | 0.0114 | 0.0712 | 6125 |
| Right banks of the superior temporal sulcus | 0.162013 | 0.351490 | 0.46 | 6.45E-01 | 0.6982 | 0.0119 | 6040 |
| Right caudal anterior cingulate cortex | 0.319825 | 0.407845 | 0.78 | 4.33E-01 | 0.5111 | 0.0199 | 6158 |
| Right caudal middle frontal gyrus | -0.825501 | 0.939277 | -0.88 | 3.80E-01 | 0.4548 | -0.0225 | 6151 |
| Right cuneus | -0.446716 | 0.528640 | -0.85 | 3.98E-01 | 0.4735 | -0.0218 | 6101 |
| Right entorhinal cortex | -0.172223 | 0.221387 | -0.78 | 4.37E-01 | 0.5115 | -0.0207 | 5687 |
| Right fusiform gyrus | -0.460594 | 0.937140 | -0.49 | 6.23E-01 | 0.6794 | -0.0126 | 6047 |
| Right inferior parietal cortex | -2.407238 | 1.655171 | -1.45 | 1.46E-01 | 0.2009 | -0.0373 | 6078 |
| Right inferior temporal gyrus | -2.912625 | 1.059786 | -2.75 | 6.00E-03 | 0.0122 | -0.0704 | 6141 |
| Right isthmus cingulate cortex | 2.043375 | 0.377668 | 5.41 | 6.53E-08 | <0.0001 | 0.1378 | 6200 |
| Right lateral occipital cortex | -0.903814 | 1.447419 | -0.62 | 5.32E-01 | 0.6057 | -0.0158 | 6189 |
| Right lateral orbitofrontal cortex | 1.537736 | 0.717529 | 2.14 | 3.21E-02 | 0.0536 | 0.0544 | 6215 |
| Right lingual gyrus | -2.332428 | 1.013912 | -2.30 | 2.15E-02 | 0.0379 | -0.0587 | 6172 |
| Right medial orbitofrontal cortex | 0.596144 | 0.498951 | 1.19 | 2.32E-01 | 0.2964 | 0.0305 | 6128 |
| Right middle temporal gyrus | -0.668609 | 0.934447 | -0.72 | 4.74E-01 | 0.5476 | -0.0185 | 6068 |
| Right parahippcampal gyrus | 0.273771 | 0.239303 | 1.14 | 2.53E-01 | 0.3124 | 0.0292 | 6142 |
| Right paracentral lobule | 2.191919 | 0.546339 | 4.01 | 6.10E-05 | 0.0002 | 0.1027 | 6134 |
| Right pars opercularis | 0.186044 | 0.602413 | 0.31 | 7.57E-01 | 0.7981 | 0.0079 | 6125 |
| Right pars orbitalis | -0.132497 | 0.244833 | -0.54 | 5.88E-01 | 0.6540 | -0.0138 | 6193 |
| Right pars triangularis | -0.257354 | 0.607015 | -0.42 | 6.72E-01 | 0.7222 | -0.0108 | 6137 |
| Right pericalcarine | -1.523626 | 0.648409 | -2.35 | 1.88E-02 | 0.0340 | -0.0603 | 6106 |
| Right precentral gyrus | 1.870319 | 1.061206 | 1.76 | 7.80E-02 | 0.1134 | 0.0452 | 6105 |
| Right posterior cingulate cortex | 1.493147 | 0.439683 | 3.40 | 6.90E-04 | 0.0018 | 0.0866 | 6202 |
| Right precentral gyrus | 1.603173 | 1.270129 | 1.26 | 2.07E-01 | 0.2685 | 0.0323 | 6114 |
| Right precuneus | 2.508480 | 1.107059 | 2.27 | 2.35E-02 | 0.0410 | 0.0579 | 6188 |
| Right rostral anterior cingulate cortex | 0.792656 | 0.350830 | 2.26 | 2.39E-02 | 0.0412 | 0.0580 | 6103 |
| Right rostral middle frontal gyrus | -4.786272 | 1.708689 | -2.80 | 5.11E-03 | 0.0110 | -0.0715 | 6175 |
| Right superior frontal gyrus | 4.607031 | 1.801160 | 2.56 | 1.06E-02 | 0.0202 | 0.0656 | 6123 |
| Right superior parietal cortex | 1.605026 | 1.466039 | 1.09 | 2.74E-01 | 0.3356 | 0.0279 | 6132 |
| Right superior temporal gyrus | 1.770374 | 0.901872 | 1.96 | 4.97E-02 | 0.0789 | 0.0510 | 5943 |
| Right supramarginal gyrus | 2.044860 | 1.247932 | 1.64 | 1.01E-01 | 0.1421 | 0.0425 | 5992 |
| Right frontal pole | 0.028348 | 0.116632 | 0.24 | 8.08E-01 | 0.8184 | 0.0061 | 6216 |
| Right temporal pole | 0.304800 | 0.171469 | 1.78 | 7.55E-02 | 0.1109 | 0.0456 | 6122 |
| Right transverse temporal gyrus | 0.324418 | 0.148480 | 2.18 | 2.89E-02 | 0.0490 | 0.0554 | 6223 |
| Right insula | 1.881507 | 0.653855 | 2.88 | 4.00E-03 | 0.0088 | 0.0741 | 6076 |

| **Label** | **Estimate** | **StdError** | **T** | **p** | **FDR adjusted p** | **Cohen´s d** | **N** |
| --- | --- | --- | --- | --- | --- | --- | --- |
|  |  |  |  |  |  |  |  |
| **Cortical thickness** |  |  |  |  |  |  |  |
| Left banks of the superior temporal sulcus | -0.001311 | 0.000392 | -3.35 | 8.20E-04 | 0.0029 | -0.0869 | 5977 |
| Left caudal anterior cingulate cortex | -0.000373 | 0.000617 | -0.60 | 5.46E-01 | 0.6293 | -0.0152 | 6285 |
| Left caudal middle frontal gyrus | -0.000076 | 0.000281 | -0.27 | 7.86E-01 | 0.8218 | -0.0068 | 6263 |
| Left cuneus | 0.000736 | 0.000319 | 2.30 | 2.13E-02 | 0.0467 | 0.0586 | 6195 |
| Left entorhinal cortex | -0.003861 | 0.000946 | -4.08 | 4.57E-05 | 0.0003 | -0.1051 | 6066 |
| Left fusiform gyrus | -0.000017 | 0.000745 | -0.02 | 9.82E-01 | 0.9824 | -0.0005 | 6276 |
| Left inferior parietal cortex | -0.002413 | 0.000303 | -7.97 | 1.90E-15 | <0.0001 | -0.2027 | 6220 |
| Left inferior temporal gyrus | 0.000422 | 0.000237 | 1.78 | 7.48E-02 | 0.1271 | 0.0454 | 6193 |
| Left isthmus cingulate cortex | -0.001205 | 0.000355 | -3.39 | 6.90E-04 | 0.0028 | -0.0858 | 6284 |
| Left lateral occipital cortex | -0.000898 | 0.000370 | -2.43 | 1.53E-02 | 0.0372 | -0.0616 | 6267 |
| Left lateral orbitofrontal cortex | -0.001179 | 0.000492 | -2.40 | 1.65E-02 | 0.0374 | -0.0608 | 6267 |
| Left lingual gyrus | 0.000788 | 0.000248 | 3.18 | 1.50E-03 | 0.0046 | 0.0806 | 6254 |
| Left medial orbitofrontal cortex | 0.000255 | 0.000352 | 0.73 | 4.68E-01 | 0.5609 | 0.0186 | 6214 |
| Left middle temporal gyrus | 0.000458 | 0.000274 | 1.67 | 9.50E-02 | 0.1538 | 0.0430 | 6063 |
| Left parahippcampal gyrus | -0.000369 | 0.000387 | -0.95 | 3.41E-01 | 0.4380 | -0.0240 | 6275 |
| Left paracentral lobule | -0.001315 | 0.000341 | -3.86 | 1.10E-04 | 0.0007 | -0.0977 | 6282 |
| Left pars opercularis | -0.000164 | 0.000321 | -0.51 | 6.09E-01 | 0.6787 | -0.0129 | 6264 |
| Left pars orbitalis | -0.001945 | 0.000807 | -2.41 | 1.60E-02 | 0.0374 | -0.0610 | 6279 |
| Left pars triangularis | -0.000204 | 0.000296 | -0.69 | 4.90E-01 | 0.5745 | -0.0175 | 6268 |
| Left pericalcarine | 0.000785 | 0.000504 | 1.56 | 1.19E-01 | 0.1885 | 0.0398 | 6190 |
| Left precentral gyrus | 0.001133 | 0.000338 | 3.35 | 8.00E-04 | 0.0029 | 0.0851 | 6225 |
| Left posterior cingulate cortex | 0.001478 | 0.000324 | 4.57 | 5.06E-06 | <0.0001 | 0.1156 | 6287 |
| Left precentral gyrus | 0.001393 | 0.000239 | 5.83 | 5.81E-09 | <0.0001 | 0.1479 | 6246 |
| Left precuneus | -0.001167 | 0.000368 | -3.17 | 1.50E-03 | 0.0046 | -0.0803 | 6272 |
| Left rostral anterior cingulate cortex | -0.000229 | 0.000263 | -0.87 | 3.83E-01 | 0.4731 | -0.0221 | 6255 |
| Left rostral middle frontal gyrus | 0.000213 | 0.000228 | 0.93 | 3.52E-01 | 0.4433 | 0.0235 | 6276 |
| Left superior frontal gyrus | -0.001584 | 0.000574 | -2.76 | 5.83E-03 | 0.0152 | -0.0699 | 6272 |
| Left superior parietal cortex | 0.000612 | 0.000242 | 2.53 | 1.14E-02 | 0.0286 | 0.0642 | 6239 |
| Left superior temporal gyrus | 0.000079 | 0.000230 | 0.34 | 7.33E-01 | 0.7790 | 0.0088 | 5949 |
| Left supramarginal gyrus | 0.000748 | 0.000209 | 3.58 | 3.50E-04 | 0.0016 | 0.0917 | 6128 |
| Left frontal pole | -0.001177 | 0.000315 | -3.74 | 1.90E-04 | 0.0011 | -0.0945 | 6300 |
| Left temporal pole | 0.000271 | 0.000236 | 1.15 | 2.52E-01 | 0.3491 | 0.0291 | 6262 |
| Left transverse temporal gyrus | -0.001748 | 0.000930 | -1.88 | 6.03E-02 | 0.1108 | -0.0476 | 6276 |
| Left insula | -0.001787 | 0.000520 | -3.44 | 5.90E-04 | 0.0025 | -0.0875 | 6217 |
| Right banks of the superior temporal sulcus | -0.000783 | 0.000411 | -1.90 | 5.72E-02 | 0.1080 | -0.0485 | 6165 |
| Right caudal anterior cingulate cortex | -0.000689 | 0.000577 | -1.19 | 2.32E-01 | 0.3308 | -0.0301 | 6288 |
| Right caudal middle frontal gyrus | 0.000360 | 0.000302 | 1.19 | 2.33E-01 | 0.3308 | 0.0301 | 6271 |
| Right cuneus | 0.000595 | 0.000320 | 1.86 | 6.36E-02 | 0.1138 | 0.0474 | 6199 |
| Right entorhinal cortex | -0.002082 | 0.001031 | -2.02 | 4.36E-02 | 0.0872 | -0.0523 | 6010 |
| Right fusiform gyrus | 0.000175 | 0.000735 | 0.24 | 8.11E-01 | 0.8236 | 0.0061 | 6281 |
| Right inferior parietal cortex | -0.002594 | 0.000328 | -7.92 | 2.85E-15 | <0.0001 | -0.2013 | 6224 |
| Right inferior temporal gyrus | 0.000811 | 0.000243 | 3.34 | 8.60E-04 | 0.0029 | 0.0849 | 6227 |
| Right isthmus cingulate cortex | -0.001578 | 0.000384 | -4.11 | 3.98E-05 | 0.0003 | -0.1040 | 6284 |
| Right lateral occipital cortex | -0.000411 | 0.000404 | -1.02 | 3.09E-01 | 0.4040 | -0.0258 | 6271 |
| Right lateral orbitofrontal cortex | -0.000969 | 0.000484 | -2.00 | 4.55E-02 | 0.0884 | -0.0506 | 6283 |
| Right lingual gyrus | 0.001094 | 0.000262 | 4.18 | 2.99E-05 | 0.0003 | 0.1061 | 6239 |
| Right medial orbitofrontal cortex | -0.000264 | 0.000365 | -0.72 | 4.70E-01 | 0.5609 | -0.0183 | 6237 |
| Right middle temporal gyrus | 0.000638 | 0.000280 | 2.28 | 2.28E-02 | 0.0485 | 0.0580 | 6212 |
| Right parahippcampal gyrus | -0.001267 | 0.000400 | -3.17 | 1.54E-03 | 0.0046 | -0.0802 | 6280 |
| Right paracentral lobule | -0.001307 | 0.000355 | -3.69 | 2.30E-04 | 0.0011 | -0.0933 | 6286 |
| Right pars opercularis | 0.000083 | 0.000322 | 0.26 | 7.98E-01 | 0.8218 | 0.0066 | 6242 |
| Right pars orbitalis | -0.002534 | 0.000688 | -3.68 | 2.30E-04 | 0.0011 | -0.0931 | 6277 |
| Right pars triangularis | -0.000446 | 0.000337 | -1.32 | 1.87E-01 | 0.2761 | -0.0335 | 6241 |
| Right pericalcarine | 0.000232 | 0.000504 | 0.46 | 6.46E-01 | 0.7081 | 0.0117 | 6172 |
| Right precentral gyrus | 0.000380 | 0.000338 | 1.13 | 2.60E-01 | 0.3536 | 0.0287 | 6245 |
| Right posterior cingulate cortex | 0.001026 | 0.000331 | 3.10 | 1.90E-03 | 0.0054 | 0.0784 | 6290 |
| Right precentral gyrus | 0.001207 | 0.000262 | 4.61 | 4.03E-06 | <0.0001 | 0.1169 | 6254 |
| Right precuneus | -0.001725 | 0.000364 | -4.74 | 2.23E-06 | <0.0001 | -0.1200 | 6274 |
| Right rostral anterior cingulate cortex | -0.000312 | 0.000282 | -1.10 | 2.69E-01 | 0.3593 | -0.0279 | 6258 |
| Right rostral middle frontal gyrus | -0.000100 | 0.000239 | -0.42 | 6.75E-01 | 0.7286 | -0.0106 | 6269 |
| Right superior frontal gyrus | -0.001238 | 0.000581 | -2.13 | 3.31E-02 | 0.0681 | -0.0539 | 6277 |
| Right superior parietal cortex | 0.000154 | 0.000267 | 0.58 | 5.63E-01 | 0.6386 | 0.0147 | 6256 |
| Right superior temporal gyrus | -0.000445 | 0.000247 | -1.80 | 7.23E-02 | 0.1260 | -0.0463 | 6081 |
| Right supramarginal gyrus | 0.001266 | 0.000223 | 5.68 | 1.38E-08 | <0.0001 | 0.1453 | 6145 |
| Right frontal pole | -0.000915 | 0.000320 | -2.86 | 4.26E-03 | 0.0116 | -0.0723 | 6296 |
| Right temporal pole | 0.000456 | 0.000267 | 1.71 | 8.78E-02 | 0.1456 | 0.0433 | 6267 |
| Right transverse temporal gyrus | -0.001516 | 0.000980 | -1.55 | 1.22E-01 | 0.1887 | -0.0393 | 6267 |
| Right insula | -0.000776 | 0.000561 | -1.38 | 1.66E-01 | 0.2513 | -0.0352 | 6165 |

| **Label** | **Estimate** | **StdError** | **T** | **p** | **FDR adjusted p** | **Cohen´s d** | **N** |
| --- | --- | --- | --- | --- | --- | --- | --- |
|  |  |  |  |  |  |  |  |
| **Global measures** |  |  |  |  |  |  |  |
| Total Intracranial Volume | -28.100000 | 7.200000 | -3.90 | 9.89E-05 | 0.0003 | -0.0998 | 6112 |
| Total left hemispheral surface area | -0.690000 | 0.326000 | -2.12 | 3.44E-02 | 0.0563 | -0.0535 | 6287 |
| Total right hemispheral surface area | -0.741000 | 0.327000 | -2.27 | 2.34E-02 | 0.0395 | -0.0573 | 6287 |
| Left hemispheral average thickness | -0.000030 | 0.000004 | -7.17 | 8.20E-13 | <0.0001 | -0.1806 | 6306 |
| Right hemispheral average thickness | -0.000028 | 0.000004 | -6.73 | 1.81E-11 | <0.0001 | -0.1695 | 6305 |
|  |  |  |  |  |  |  |  |
| **Cortical thickness** |  |  |  |  |  |  |  |
| Left banks of the superior temporal sulcus | -0.000055 | 0.000008 | -6.81 | 1.10E-11 | <0.0001 | -0.1762 | 5977 |
| Left caudal anterior cingulate cortex | -0.000030 | 0.000011 | -2.67 | 7.68E-03 | 0.0153 | -0.0674 | 6285 |
| Left caudal middle frontal cortex | -0.000034 | 0.000007 | -5.04 | 4.77E-07 | <0.0001 | -0.1274 | 6263 |
| Left cuneus | -0.000005 | 0.000006 | -0.85 | 3.97E-01 | 0.4651 | -0.0216 | 6195 |
| Left entorhinal cortex | -0.000105 | 0.000017 | -6.24 | 4.72E-10 | <0.0001 | -0.1603 | 6066 |
| Left fusiform cortex | -0.000073 | 0.000007 | -11.02 | <2.00E-16 | <0.0001 | -0.2783 | 6276 |
| Left inferior parietal cortex | -0.000025 | 0.000006 | -4.24 | 2.24E-05 | 0.0001 | -0.1075 | 6220 |
| Left inferior temporal cortex | -0.000053 | 0.000008 | -7.03 | 2.34E-12 | <0.0001 | -0.1787 | 6193 |
| Left isthmus cingulate cortex | -0.000040 | 0.000009 | -4.45 | 8.81E-06 | <0.0001 | -0.1123 | 6284 |
| Left lateral occipital cortex | -0.000013 | 0.000006 | -2.36 | 1.80E-02 | 0.0314 | -0.0596 | 6267 |
| Left lateral orbitofrontal cortex | -0.000024 | 0.000007 | -3.24 | 1.20E-03 | 0.0030 | -0.0819 | 6267 |
| Left lingual cortex | -0.000014 | 0.000006 | -2.54 | 1.10E-02 | 0.0199 | -0.0642 | 6254 |
| Left medial orbitofrontal cortex | -0.000029 | 0.000007 | -3.86 | 1.10E-04 | 0.0003 | -0.0979 | 6214 |
| Left middle temporal cortex | -0.000059 | 0.000008 | -7.59 | 3.80E-14 | <0.0001 | -0.1950 | 6063 |
| Left parahippocampal cortex | -0.000063 | 0.000014 | -4.38 | 1.18E-05 | <0.0001 | -0.1106 | 6275 |
| Left paracentral cortex | -0.000033 | 0.000007 | -4.81 | 1.54E-06 | <0.0001 | -0.1214 | 6282 |
| Left pars opercularis | -0.000036 | 0.000007 | -5.31 | 1.15E-07 | <0.0001 | -0.1342 | 6264 |
| Left pars orbitalis | -0.000024 | 0.000010 | -2.37 | 1.79E-02 | 0.0314 | -0.0598 | 6279 |
| Left pars triangularis | -0.000010 | 0.000007 | -1.37 | 1.71E-01 | 0.2251 | -0.0346 | 6268 |
| Left pericalcarine | 0.000009 | 0.000006 | 1.51 | 1.30E-01 | 0.1758 | 0.0384 | 6190 |
| Left postcentral cortex | -0.000003 | 0.000005 | -0.50 | 6.15E-01 | 0.6709 | -0.0127 | 6225 |
| Left posterior cingulate cortex | -0.000046 | 0.000007 | -6.25 | 4.40E-10 | <0.0001 | -0.1577 | 6287 |
| Left precentral cortex | -0.000036 | 0.000006 | -5.73 | 1.03E-08 | <0.0001 | -0.1450 | 6246 |
| Left precuneus | -0.000027 | 0.000006 | -4.68 | 2.90E-06 | <0.0001 | -0.1182 | 6272 |
| Left rostral anterior cingulate cortex | -0.000054 | 0.000011 | -5.09 | 3.70E-07 | <0.0001 | -0.1287 | 6255 |
| Left rostral middle frontal cortex | -0.000022 | 0.000006 | -3.50 | 4.60E-04 | 0.0013 | -0.0884 | 6276 |
| Left superior frontal cortex | -0.000034 | 0.000007 | -5.21 | 1.99E-07 | <0.0001 | -0.1316 | 6272 |
| Left superior parietal cortex | -0.000017 | 0.000005 | -3.10 | 1.90E-03 | 0.0047 | -0.0785 | 6239 |
| Left superior temporal cortex | -0.000055 | 0.000007 | -7.46 | 1.00E-13 | <0.0001 | -0.1935 | 5949 |
| Left supramarginal cortex | -0.000029 | 0.000006 | -4.72 | 2.44E-06 | <0.0001 | -0.1206 | 6128 |
| Left frontal pole | -0.000036 | 0.000014 | -2.58 | 1.00E-02 | 0.0187 | -0.0650 | 6300 |
| Left temporal pole | -0.000077 | 0.000017 | -4.52 | 6.38E-06 | <0.0001 | -0.1143 | 6262 |
| Left transverse temporal cortex | -0.000062 | 0.000010 | -6.20 | 5.93E-10 | <0.0001 | -0.1565 | 6276 |
| Left insula | -0.000042 | 0.000007 | -5.63 | 1.88E-08 | <0.0001 | -0.1428 | 6217 |
| Right banks of the superior temporal sulcus | -0.000042 | 0.000008 | -5.29 | 1.25E-07 | <0.0001 | -0.1348 | 6165 |
| Right caudal anterior cingulate cortex | -0.000031 | 0.000010 | -2.99 | 2.77E-03 | 0.0064 | -0.0754 | 6288 |
| Right caudal middle frontal cortex | -0.000024 | 0.000007 | -3.62 | 3.00E-04 | 0.0009 | -0.0914 | 6271 |
| Right cuneus | -0.000010 | 0.000006 | -1.53 | 1.27E-01 | 0.1734 | -0.0389 | 6199 |
| Right entorhinal cortex | -0.000070 | 0.000018 | -3.86 | 1.10E-04 | 0.0003 | -0.0996 | 6010 |
| Right fusiform cortex | -0.000072 | 0.000007 | -10.68 | <2.00E-16 | <0.0001 | -0.2696 | 6281 |
| Right inferior parietal cortex | -0.000016 | 0.000006 | -2.70 | 6.90E-03 | 0.0144 | -0.0685 | 6224 |
| Right inferior temporal cortex | -0.000058 | 0.000008 | -7.51 | 6.70E-14 | <0.0001 | -0.1904 | 6227 |
| Right isthmus cingulate cortex | -0.000035 | 0.000009 | -3.98 | 7.10E-05 | 0.0002 | -0.1004 | 6284 |
| Right lateral occipital cortex | -0.000007 | 0.000006 | -1.30 | 1.90E-01 | 0.2406 | -0.0328 | 6271 |
| Right lateral orbitofrontal cortex | -0.000032 | 0.000007 | -4.27 | 1.94E-05 | 0.0001 | -0.1078 | 6283 |
| Right lingual cortex | -0.000009 | 0.000006 | -1.56 | 1.20E-01 | 0.1653 | -0.0395 | 6239 |
| Right medial orbitofrontal cortex | -0.000044 | 0.000008 | -5.72 | 1.11E-08 | <0.0001 | -0.1449 | 6237 |
| Right middle temporal cortex | -0.000056 | 0.000007 | -7.55 | 5.14E-14 | <0.0001 | -0.1916 | 6212 |
| Right parahippocampal cortex | -0.000071 | 0.000012 | -5.72 | 1.09E-08 | <0.0001 | -0.1444 | 6280 |
| Right paracentral cortex | -0.000027 | 0.000007 | -3.88 | 1.10E-04 | 0.0003 | -0.0979 | 6286 |
| Right pars opercularis | -0.000038 | 0.000007 | -5.35 | 9.32E-08 | <0.0001 | -0.1355 | 6242 |
| Right pars orbitalis | -0.000030 | 0.000010 | -3.02 | 2.60E-03 | 0.0061 | -0.0762 | 6277 |
| Right pars triangularis | -0.000023 | 0.000007 | -3.23 | 1.20E-03 | 0.0030 | -0.0818 | 6241 |
| Right pericalcarine | 0.000001 | 0.000006 | 0.17 | 8.60E-01 | 0.9001 | 0.0043 | 6172 |
| Right postcentral cortex | -0.000003 | 0.000006 | -0.51 | 6.13E-01 | 0.6709 | -0.0129 | 6245 |
| Right posterior cingulate cortex | -0.000052 | 0.000007 | -7.29 | 3.52E-13 | <0.0001 | -0.1839 | 6290 |
| Right precentral cortex | -0.000035 | 0.000006 | -5.53 | 3.31E-08 | <0.0001 | -0.1399 | 6254 |
| Right precuneus | -0.000031 | 0.000006 | -5.29 | 1.30E-07 | <0.0001 | -0.1336 | 6274 |
| Right rostral anterior cingulate cortex | -0.000041 | 0.000011 | -3.92 | 9.10E-05 | 0.0003 | -0.0991 | 6258 |
| Right rostral middle frontal cortex | -0.000028 | 0.000006 | -4.45 | 8.80E-06 | <0.0001 | -0.1124 | 6269 |
| Right superior frontal cortex | -0.000042 | 0.000007 | -6.46 | 1.10E-10 | <0.0001 | -0.1631 | 6277 |
| Right superior parietal cortex | -0.000006 | 0.000005 | -1.07 | 2.87E-01 | 0.3493 | -0.0271 | 6256 |
| Right superior temporal cortex | -0.000053 | 0.000007 | -7.32 | 2.90E-13 | <0.0001 | -0.1878 | 6081 |
| Right supramarginal cortex | -0.000024 | 0.000006 | -3.84 | 1.20E-04 | 0.0004 | -0.0980 | 6145 |
| Right frontal pole | -0.000036 | 0.000014 | -2.59 | 9.50E-03 | 0.0182 | -0.0653 | 6296 |
| Right temporal pole | -0.000067 | 0.000018 | -3.80 | 1.50E-04 | 0.0004 | -0.0960 | 6267 |
| Right transverse temporal cortex | -0.000042 | 0.000011 | -3.97 | 7.37E-05 | 0.0002 | -0.1003 | 6267 |
| Right insula | -0.000034 | 0.000008 | -4.26 | 2.04E-05 | 0.0001 | -0.1085 | 6165 |
|  |  |  |  |  |  |  |  |
| **Subcortical volumes** |  |  |  |  |  |  |  |
| Left lateral ventricle | 0.351867 | 0.218314 | 1.61 | 1.07E-01 | 0.1501 | 0.0416 | 5991 |
| Right lateral ventricle | 0.451511 | 0.202486 | 2.23 | 2.58E-02 | 0.0426 | 0.0577 | 5976 |
| Left thalamus | 0.213195 | 0.033117 | 6.44 | 1.31E-10 | 0.0000 | 0.1667 | 5971 |
| Right thalamus | 0.132450 | 0.028605 | 4.63 | 3.73E-06 | 0.0000 | 0.1200 | 5957 |
| Left caudate | 0.002053 | 0.019424 | 0.11 | 9.16E-01 | 0.9460 | 0.0028 | 5968 |
| Right caudate | 0.001130 | 0.019639 | 0.06 | 9.54E-01 | 0.9727 | 0.0016 | 5914 |
| Left putamen | 0.059313 | 0.026579 | 2.23 | 2.57E-02 | 0.0426 | 0.0587 | 5773 |
| Right putamen | 0.049095 | 0.024686 | 1.99 | 4.68E-02 | 0.0742 | 0.0521 | 5842 |
| Left pallidum | -0.025678 | 0.010085 | -2.55 | 1.09E-02 | 0.0199 | -0.0676 | 5702 |
| Right pallidum | -0.009094 | 0.008960 | -1.01 | 3.10E-01 | 0.3717 | -0.0262 | 5925 |
| Left hippocampus | 0.049150 | 0.017852 | 2.75 | 5.90E-03 | 0.0127 | 0.0714 | 5943 |
| Right hippocamups | 0.058751 | 0.017710 | 3.32 | 9.10E-04 | 0.0025 | 0.0858 | 5994 |
| Left amygdala | 0.043000 | 0.008395 | 5.12 | 3.12E-07 | <0.0001 | 0.1327 | 5956 |
| Right amygdala | 0.071576 | 0.008924 | 8.02 | 1.30E-15 | <0.0001 | 0.2077 | 5967 |
| Left nucleus accumbens | 0.013772 | 0.004483 | 3.07 | 2.14E-03 | 0.0051 | 0.0802 | 5856 |
| Right nucleus accumbens | -0.000183 | 0.004091 | -0.04 | 9.64E-01 | 0.9749 | -0.0010 | 5879 |
|  |  |  |  |  |  |  |  |
| **Cortical surface area** |  |  |  |  |  |  |  |
| Left banks of the superior temporal sulcus | -0.014662 | 0.007389 | -1.98 | 4.73E-02 | 0.0742 | -0.0531 | 5566 |
| Left caudal anterior cingulate cortex | -0.000511 | 0.006491 | -0.08 | 9.37E-01 | 0.9618 | -0.0021 | 5904 |
| Left caudal middle frontal cortex | 0.012888 | 0.016287 | 0.79 | 4.29E-01 | 0.4950 | 0.0206 | 5904 |
| Left cuneus | -0.013387 | 0.009739 | -1.37 | 1.69E-01 | 0.2251 | -0.0358 | 5859 |
| Left entorhinal cortex | 0.000118 | 0.003760 | 0.03 | 9.75E-01 | 0.9749 | 0.0008 | 5555 |
| Left fusiform cortex | -0.054039 | 0.016461 | -3.28 | 1.03E-03 | 0.0027 | -0.0862 | 5799 |
| Left inferior parietal cortex | -0.049390 | 0.026320 | -1.88 | 6.06E-02 | 0.0915 | -0.0492 | 5853 |
| Left inferior temporal cortex | -0.050846 | 0.019672 | -2.58 | 9.77E-03 | 0.0185 | -0.0673 | 5876 |
| Left isthmus cingulate cortex | 0.040520 | 0.007115 | 5.69 | 1.29E-08 | 0.0000 | 0.1474 | 5962 |
| Left lateral occipital cortex | -0.048906 | 0.024961 | -1.96 | 5.00E-02 | 0.0777 | -0.0508 | 5946 |
| Left lateral orbitofrontal cortex | 0.013042 | 0.011563 | 1.13 | 2.59E-01 | 0.3207 | 0.0292 | 5979 |
| Left lingual cortex | -0.056589 | 0.017805 | -3.18 | 1.50E-03 | 0.0037 | -0.0825 | 5944 |
| Left medial orbitofrontal cortex | 0.005399 | 0.010016 | 0.54 | 5.90E-01 | 0.6522 | 0.0141 | 5841 |
| Left middle temporal cortex | -0.022633 | 0.016796 | -1.35 | 1.78E-01 | 0.2327 | -0.0358 | 5678 |
| Left parahippocampal cortex | 0.007504 | 0.004171 | 1.80 | 7.21E-02 | 0.1067 | 0.0469 | 5897 |
| Left paracentral cortex | 0.026267 | 0.008471 | 3.10 | 1.94E-03 | 0.0047 | 0.0812 | 5838 |
| Left pars opercularis | 0.004525 | 0.011545 | 0.39 | 6.95E-01 | 0.7475 | 0.0101 | 5921 |
| Left pars orbitalis | -0.000522 | 0.003608 | -0.14 | 8.80E-01 | 0.9150 | -0.0036 | 5968 |
| Left pars triangularis | -0.002416 | 0.008872 | -0.27 | 7.85E-01 | 0.8332 | -0.0070 | 5943 |
| Left pericalcarine | -0.032103 | 0.010775 | -2.98 | 2.90E-03 | 0.0066 | -0.0777 | 5888 |
| Left postcentral cortex | 0.024311 | 0.018410 | 1.32 | 1.87E-01 | 0.2383 | 0.0346 | 5838 |
| Left posterior cingulate cortex | 0.019757 | 0.007751 | 2.55 | 1.08E-02 | 0.0199 | 0.0660 | 5965 |
| Left precentral cortex | 0.043257 | 0.021037 | 2.06 | 3.98E-02 | 0.0644 | 0.0537 | 5881 |
| Left precuneus | 0.043118 | 0.018305 | 2.36 | 1.90E-02 | 0.0328 | 0.0612 | 5949 |
| Left rostral anterior cingulate cortex | 0.017559 | 0.006641 | 2.64 | 8.21E-03 | 0.0159 | 0.0692 | 5816 |
| Left rostral middle frontal cortex | -0.041646 | 0.028863 | -1.44 | 1.49E-01 | 0.2001 | -0.0373 | 5952 |
| Left superior frontal cortex | 0.082394 | 0.030857 | 2.67 | 7.60E-03 | 0.0153 | 0.0698 | 5859 |
| Left superior parietal cortex | 0.046426 | 0.026746 | 1.74 | 8.27E-02 | 0.1202 | 0.0454 | 5875 |
| Left superior temporal cortex | 0.030161 | 0.017768 | 1.70 | 8.97E-02 | 0.1280 | 0.0455 | 5577 |
| Left supramarginal cortex | 0.044284 | 0.023046 | 1.92 | 5.47E-02 | 0.0842 | 0.0509 | 5701 |
| Left frontal pole | 0.002812 | 0.001602 | 1.76 | 7.93E-02 | 0.1164 | 0.0455 | 5990 |
| Left temporal pole | -0.000100 | 0.002767 | -0.04 | 9.71E-01 | 0.9749 | -0.0010 | 5954 |
| Left transverse temporal cortex | 0.014248 | 0.003341 | 4.26 | 2.04E-05 | 0.0001 | 0.1101 | 5986 |
| Left insula | 0.028791 | 0.010061 | 2.86 | 4.23E-03 | 0.0092 | 0.0745 | 5899 |
| Right banks of the superior temporal sulcus | 0.001314 | 0.006050 | 0.22 | 8.28E-01 | 0.8725 | 0.0058 | 5804 |
| Right caudal anterior cingulate cortex | 0.005597 | 0.007024 | 0.80 | 4.26E-01 | 0.4950 | 0.0208 | 5930 |
| Right caudal middle frontal cortex | -0.011153 | 0.016201 | -0.69 | 4.91E-01 | 0.5588 | -0.0179 | 5922 |
| Right cuneus | -0.007984 | 0.009163 | -0.87 | 3.84E-01 | 0.4528 | -0.0227 | 5874 |
| Right entorhinal cortex | -0.001811 | 0.003816 | -0.47 | 6.35E-01 | 0.6877 | -0.0127 | 5468 |
| Right fusiform cortex | -0.010430 | 0.016209 | -0.64 | 5.20E-01 | 0.5831 | -0.0168 | 5817 |
| Right inferior parietal cortex | -0.047670 | 0.028611 | -1.67 | 9.57E-02 | 0.1354 | -0.0437 | 5850 |
| Right inferior temporal cortex | -0.059659 | 0.018394 | -3.24 | 1.19E-03 | 0.0030 | -0.0843 | 5911 |
| Right isthmus cingulate cortex | 0.032800 | 0.006519 | 5.03 | 5.00E-07 | 0.0000 | 0.1302 | 5976 |
| Right lateral occipital cortex | -0.026422 | 0.024962 | -1.06 | 2.90E-01 | 0.3502 | -0.0275 | 5962 |
| Right lateral orbitofrontal cortex | 0.024848 | 0.012431 | 2.00 | 4.57E-02 | 0.0731 | 0.0517 | 5984 |
| Right lingual cortex | -0.048071 | 0.017490 | -2.75 | 6.01E-03 | 0.0128 | -0.0714 | 5942 |
| Right medial orbitofrontal cortex | 0.011559 | 0.008632 | 1.34 | 1.81E-01 | 0.2343 | 0.0349 | 5909 |
| Right middle temporal cortex | -0.018744 | 0.016268 | -1.15 | 2.49E-01 | 0.3106 | -0.0301 | 5839 |
| Right parahippocampal cortex | 0.004105 | 0.004149 | 0.99 | 3.23E-01 | 0.3837 | 0.0258 | 5909 |
| Right paracentral cortex | 0.033763 | 0.009421 | 3.58 | 3.40E-04 | 0.0010 | 0.0932 | 5904 |
| Right pars opercularis | 0.005956 | 0.010406 | 0.57 | 5.67E-01 | 0.6315 | 0.0148 | 5897 |
| Right pars orbitalis | -0.003282 | 0.004219 | -0.78 | 4.37E-01 | 0.5005 | -0.0202 | 5964 |
| Right pars triangularis | -0.006817 | 0.010434 | -0.65 | 5.14E-01 | 0.5801 | -0.0169 | 5909 |
| Right pericalcarine | -0.029730 | 0.011166 | -2.66 | 7.78E-03 | 0.0153 | -0.0694 | 5882 |
| Right postcentral cortex | 0.034544 | 0.018353 | 1.88 | 5.99E-02 | 0.0912 | 0.0490 | 5883 |
| Right posterior cingulate cortex | 0.022301 | 0.007580 | 2.94 | 3.27E-03 | 0.0073 | 0.0761 | 5972 |
| Right precentral cortex | 0.024252 | 0.021980 | 1.10 | 2.70E-01 | 0.3311 | 0.0287 | 5894 |
| Right precuneus | 0.051247 | 0.019157 | 2.68 | 7.49E-03 | 0.0153 | 0.0694 | 5959 |
| Right rostral anterior cingulate cortex | 0.013718 | 0.006046 | 2.27 | 2.33E-02 | 0.0395 | 0.0591 | 5894 |
| Right rostral middle frontal cortex | -0.086141 | 0.029614 | -2.91 | 3.64E-03 | 0.0080 | -0.0755 | 5947 |
| Right superior frontal cortex | 0.074814 | 0.031243 | 2.39 | 1.67E-02 | 0.0297 | 0.0623 | 5897 |
| Right superior parietal cortex | 0.029823 | 0.025449 | 1.17 | 2.41E-01 | 0.3031 | 0.0305 | 5903 |
| Right superior temporal cortex | 0.025180 | 0.015662 | 1.61 | 1.08E-01 | 0.1501 | 0.0426 | 5707 |
| Right supramarginal cortex | 0.028644 | 0.021481 | 1.33 | 1.82E-01 | 0.2348 | 0.0351 | 5760 |
| Right frontal pole | 0.000554 | 0.002010 | 0.28 | 7.83E-01 | 0.8332 | 0.0072 | 5989 |
| Right temporal pole | 0.005217 | 0.003030 | 1.72 | 8.52E-02 | 0.1227 | 0.0448 | 5891 |
| Right transverse temporal cortex | 0.004757 | 0.002560 | 1.86 | 6.32E-02 | 0.0945 | 0.0481 | 5991 |
| Right insula | 0.030334 | 0.011353 | 2.67 | 7.60E-03 | 0.0153 | 0.0698 | 5849 |

| **Label** | **Estimate** | **StdError** | **T** | **p** | **FDR adjusted p** | **Cohen´s d** | **N** |
| --- | --- | --- | --- | --- | --- | --- | --- |
|  |  |  |  |  |  |  |  |
| **Global measures** |  |  |  |  |  |  |  |
| Total Intracranial Volume | -1460.33000 | 417.080000 | -3.50 | 4.70E-04 | 0.0013 | -0.0896 | 6112 |
| Total left hemispheral surface area | -29.152000 | 18.980000 | -1.54 | 1.25E-01 | 0.1731 | -0.0389 | 6287 |
| Total right hemispheral surface area | -32.328000 | 19.029000 | -1.70 | 8.94E-02 | 0.1324 | -0.0429 | 6287 |
| Left hemispheral average thickness | -0.001741 | 0.000246 | -7.08 | 1.60E-12 | <0.0001 | -0.1783 | 6306 |
| Right hemispheral average thickness | -0.001631 | 0.000246 | -6.63 | 3.53E-11 | <0.0001 | -0.1670 | 6305 |
|  |  |  |  |  |  |  |  |
| **Cortical thickness** |  |  |  |  |  |  |  |
| Left banks of the superior temporal sulcus | -0.002921 | 0.000467 | -6.25 | 4.37E-10 | <0.0001 | -0.1617 | 5977 |
| Left caudal anterior cingulate cortex | -0.001278 | 0.000645 | -1.98 | 4.75E-02 | 0.0746 | -0.0500 | 6285 |
| Left caudal middle frontal cortex | -0.002009 | 0.000391 | -5.14 | 2.77E-07 | <0.0001 | -0.1299 | 6263 |
| Left cuneus | -0.000323 | 0.000360 | -0.90 | 3.68E-01 | 0.4317 | -0.0229 | 6195 |
| Left entorhinal cortex | -0.005799 | 0.000976 | -5.94 | 2.99E-09 | <0.0001 | -0.1526 | 6066 |
| Left fusiform cortex | -0.004101 | 0.000387 | -10.59 | <2.00E-16 | <0.0001 | -0.2674 | 6276 |
| Left inferior parietal cortex | -0.001425 | 0.000350 | -4.08 | 4.61E-05 | 0.0002 | -0.1035 | 6220 |
| Left inferior temporal cortex | -0.003066 | 0.000441 | -6.95 | 3.94E-12 | <0.0001 | -0.1767 | 6193 |
| Left isthmus cingulate cortex | -0.002309 | 0.000524 | -4.40 | 1.09E-05 | <0.0001 | -0.1110 | 6284 |
| Left lateral occipital cortex | -0.000735 | 0.000320 | -2.30 | 2.20E-02 | 0.0367 | -0.0581 | 6267 |
| Left lateral orbitofrontal cortex | -0.001443 | 0.000432 | -3.34 | 8.50E-04 | 0.0022 | -0.0844 | 6267 |
| Left lingual cortex | -0.000797 | 0.000327 | -2.44 | 1.50E-02 | 0.0262 | -0.0617 | 6254 |
| Left medial orbitofrontal cortex | -0.001601 | 0.000431 | -3.71 | 2.10E-04 | 0.0006 | -0.0941 | 6214 |
| Left middle temporal cortex | -0.003290 | 0.000448 | -7.35 | 2.30E-13 | <0.0001 | -0.1888 | 6063 |
| Left parahippocampal cortex | -0.003362 | 0.000838 | -4.01 | 6.07E-05 | 0.0002 | -0.1013 | 6275 |
| Left paracentral cortex | -0.001858 | 0.000401 | -4.64 | 3.63E-06 | <0.0001 | -0.1171 | 6282 |
| Left pars opercularis | -0.001831 | 0.000389 | -4.70 | 2.61E-06 | <0.0001 | -0.1188 | 6264 |
| Left pars orbitalis | -0.001445 | 0.000591 | -2.45 | 1.40E-02 | 0.0247 | -0.0618 | 6279 |
| Left pars triangularis | -0.000627 | 0.000424 | -1.48 | 1.39E-01 | 0.1912 | -0.0374 | 6268 |
| Left pericalcarine | 0.000491 | 0.000352 | 1.40 | 1.63E-01 | 0.2133 | 0.0356 | 6190 |
| Left postcentral cortex | -0.000219 | 0.000317 | -0.69 | 4.89E-01 | 0.5417 | -0.0175 | 6225 |
| Left posterior cingulate cortex | -0.002326 | 0.000421 | -5.53 | 3.42E-08 | <0.0001 | -0.1395 | 6287 |
| Left precentral cortex | -0.002259 | 0.000369 | -6.12 | 9.69E-10 | <0.0001 | -0.1549 | 6246 |
| Left precuneus | -0.001536 | 0.000341 | -4.51 | 6.64E-06 | <0.0001 | -0.1139 | 6272 |
| Left rostral anterior cingulate cortex | -0.002607 | 0.000615 | -4.24 | 2.26E-05 | 0.0001 | -0.1072 | 6255 |
| Left rostral middle frontal cortex | -0.001495 | 0.000362 | -4.13 | 3.60E-05 | 0.0001 | -0.1043 | 6276 |
| Left superior frontal cortex | -0.002004 | 0.000379 | -5.29 | 1.27E-07 | <0.0001 | -0.1336 | 6272 |
| Left superior parietal cortex | -0.000954 | 0.000312 | -3.06 | 2.20E-03 | 0.0051 | -0.0775 | 6239 |
| Left superior temporal cortex | -0.003110 | 0.000432 | -7.21 | 6.50E-13 | <0.0001 | -0.1870 | 5949 |
| Left supramarginal cortex | -0.001703 | 0.000363 | -4.69 | 2.74E-06 | <0.0001 | -0.1198 | 6128 |
| Left frontal pole | -0.002356 | 0.000807 | -2.92 | 3.51E-03 | 0.0077 | -0.0736 | 6300 |
| Left temporal pole | -0.004677 | 0.000988 | -4.74 | 2.23E-06 | <0.0001 | -0.1198 | 6262 |
| Left transverse temporal cortex | -0.003454 | 0.000582 | -5.93 | 3.18E-09 | <0.0001 | -0.1497 | 6276 |
| Left insula | -0.002188 | 0.000430 | -5.09 | 3.64E-07 | <0.0001 | -0.1291 | 6217 |
| Right banks of the superior temporal sulcus | -0.002173 | 0.000465 | -4.68 | 2.98E-06 | <0.0001 | -0.1192 | 6165 |
| Right caudal anterior cingulate cortex | -0.001620 | 0.000600 | -2.70 | 7.00E-03 | 0.0141 | -0.0681 | 6288 |
| Right caudal middle frontal cortex | -0.001380 | 0.000389 | -3.55 | 3.90E-04 | 0.0011 | -0.0897 | 6271 |
| Right cuneus | -0.000453 | 0.000362 | -1.25 | 2.11E-01 | 0.2650 | -0.0318 | 6199 |
| Right entorhinal cortex | -0.004083 | 0.001053 | -3.88 | 1.10E-04 | 0.0004 | -0.1001 | 6010 |
| Right fusiform cortex | -0.004056 | 0.000393 | -10.32 | <2.00E-16 | <0.0001 | -0.2605 | 6281 |
| Right inferior parietal cortex | -0.000903 | 0.000341 | -2.64 | 8.20E-03 | 0.0155 | -0.0669 | 6224 |
| Right inferior temporal cortex | -0.003220 | 0.000450 | -7.16 | 8.80E-13 | <0.0001 | -0.1815 | 6227 |
| Right isthmus cingulate cortex | -0.001886 | 0.000510 | -3.70 | 2.20E-04 | 0.0007 | -0.0934 | 6284 |
| Right lateral occipital cortex | -0.000459 | 0.000331 | -1.39 | 1.66E-01 | 0.2154 | -0.0351 | 6271 |
| Right lateral orbitofrontal cortex | -0.001785 | 0.000431 | -4.14 | 3.54E-05 | 0.0001 | -0.1045 | 6283 |
| Right lingual cortex | -0.000542 | 0.000327 | -1.66 | 9.70E-02 | 0.1423 | -0.0420 | 6239 |
| Right medial orbitofrontal cortex | -0.002636 | 0.000447 | -5.90 | 3.81E-09 | <0.0001 | -0.1494 | 6237 |
| Right middle temporal cortex | -0.003013 | 0.000432 | -6.97 | 3.48E-12 | <0.0001 | -0.1769 | 6212 |
| Right parahippocampal cortex | -0.003856 | 0.000718 | -5.37 | 8.08E-08 | <0.0001 | -0.1355 | 6280 |
| Right paracentral cortex | -0.001550 | 0.000401 | -3.87 | 1.10E-04 | 0.0004 | -0.0976 | 6286 |
| Right pars opercularis | -0.001957 | 0.000408 | -4.79 | 1.69E-06 | <0.0001 | -0.1213 | 6242 |
| Right pars orbitalis | -0.001807 | 0.000578 | -3.13 | 1.77E-03 | 0.0043 | -0.0790 | 6277 |
| Right pars triangularis | -0.001289 | 0.000416 | -3.10 | 1.90E-03 | 0.0045 | -0.0785 | 6241 |
| Right pericalcarine | 0.000081 | 0.000355 | 0.23 | 8.20E-01 | 0.8412 | 0.0059 | 6172 |
| Right postcentral cortex | -0.000163 | 0.000320 | -0.51 | 6.10E-01 | 0.6515 | -0.0129 | 6245 |
| Right posterior cingulate cortex | -0.002773 | 0.000409 | -6.78 | 1.29E-11 | <0.0001 | -0.1710 | 6290 |
| Right precentral cortex | -0.002203 | 0.000373 | -5.91 | 3.59E-09 | <0.0001 | -0.1495 | 6254 |
| Right precuneus | -0.001695 | 0.000337 | -5.03 | 5.00E-07 | <0.0001 | -0.1270 | 6274 |
| Right rostral anterior cingulate cortex | -0.002114 | 0.000608 | -3.48 | 5.10E-04 | 0.0014 | -0.0880 | 6258 |
| Right rostral middle frontal cortex | -0.001829 | 0.000360 | -5.08 | 3.90E-07 | <0.0001 | -0.1283 | 6269 |
| Right superior frontal cortex | -0.002537 | 0.000379 | -6.70 | 2.30E-11 | <0.0001 | -0.1692 | 6277 |
| Right superior parietal cortex | -0.000294 | 0.000312 | -0.94 | 3.45E-01 | 0.4073 | -0.0238 | 6256 |
| Right superior temporal cortex | -0.002938 | 0.000423 | -6.95 | 4.01E-12 | <0.0001 | -0.1783 | 6081 |
| Right supramarginal cortex | -0.001250 | 0.000364 | -3.44 | 5.90E-04 | 0.0016 | -0.0878 | 6145 |
| Right frontal pole | -0.002343 | 0.000795 | -2.95 | 3.22E-03 | 0.0071 | -0.0744 | 6296 |
| Right temporal pole | -0.004124 | 0.001022 | -4.04 | 5.52E-05 | 0.0002 | -0.1021 | 6267 |
| Right transverse temporal cortex | -0.002318 | 0.000613 | -3.78 | 1.60E-04 | 0.0005 | -0.0955 | 6267 |
| Right insula | -0.001644 | 0.000456 | -3.61 | 3.10E-04 | 0.0009 | -0.0920 | 6165 |
|  |  |  |  |  |  |  |  |
| **Subcortical volumes** |  |  |  |  |  |  |  |
| Left lateral ventricle | 34.758805 | 12.441215 | 2.79 | 5.23E-03 | 0.0109 | 0.0721 | 5991 |
| Right lateral ventricle | 39.587283 | 11.544783 | 3.43 | 6.10E-04 | 0.0016 | 0.0888 | 5976 |
| Left thalamus | 10.974065 | 1.891668 | 5.80 | 6.92E-09 | <0.0001 | 0.1501 | 5971 |
| Right thalamus | 6.599230 | 1.641860 | 4.02 | 5.91E-05 | 0.0002 | 0.1042 | 5957 |
| Left caudate | 0.940133 | 1.123042 | 0.84 | 4.03E-01 | 0.4647 | 0.0218 | 5968 |
| Right caudate | 1.325165 | 1.132528 | 1.17 | 2.42E-01 | 0.2992 | 0.0304 | 5914 |
| Left putamen | 3.799182 | 1.539230 | 2.47 | 1.36E-02 | 0.0243 | 0.0650 | 5773 |
| Right putamen | 3.819068 | 1.428430 | 2.67 | 7.52E-03 | 0.0148 | 0.0699 | 5842 |
| Left pallidum | -1.335460 | 0.582272 | -2.29 | 2.19E-02 | 0.0367 | -0.0607 | 5702 |
| Right pallidum | -0.412124 | 0.518758 | -0.79 | 4.27E-01 | 0.4837 | -0.0205 | 5925 |
| Left hippocampus | 2.115021 | 1.022405 | 2.07 | 3.86E-02 | 0.0625 | 0.0537 | 5943 |
| Right hippocamups | 2.794907 | 1.013328 | 2.76 | 5.83E-03 | 0.0120 | 0.0713 | 5994 |
| Left amygdala | 2.485160 | 0.484105 | 5.13 | 2.93E-07 | <0.0001 | 0.1330 | 5956 |
| Right amygdala | 4.376161 | 0.516155 | 8.48 | <2.00E-16 | <0.0001 | 0.2196 | 5967 |
| Left nucleus accumbens | 0.919352 | 0.258753 | 3.55 | 3.80E-04 | 0.0011 | 0.0928 | 5856 |
| Right nucleus accumbens | 0.163459 | 0.236763 | 0.69 | 4.90E-01 | 0.5417 | 0.0180 | 5879 |
|  |  |  |  |  |  |  |  |
| **Cortical surface area** |  |  |  |  |  |  |  |
| Left banks of the superior temporal sulcus | -0.781504 | 0.428540 | -1.82 | 6.83E-02 | 0.1021 | -0.0488 | 5566 |
| Left caudal anterior cingulate cortex | 0.092357 | 0.375794 | 0.25 | 8.06E-01 | 0.8324 | 0.0065 | 5904 |
| Left caudal middle frontal cortex | 0.997221 | 0.943667 | 1.06 | 2.91E-01 | 0.3499 | 0.0276 | 5904 |
| Left cuneus | -0.682386 | 0.563696 | -1.21 | 2.26E-01 | 0.2817 | -0.0316 | 5859 |
| Left entorhinal cortex | -0.117126 | 0.217306 | -0.54 | 5.90E-01 | 0.6344 | -0.0145 | 5555 |
| Left fusiform cortex | -2.851910 | 0.952226 | -2.99 | 2.80E-03 | 0.0064 | -0.0785 | 5799 |
| Left inferior parietal cortex | -2.378957 | 1.523892 | -1.56 | 1.19E-01 | 0.1663 | -0.0408 | 5853 |
| Left inferior temporal cortex | -3.107555 | 1.134809 | -2.74 | 6.20E-03 | 0.0126 | -0.0715 | 5876 |
| Left isthmus cingulate cortex | 2.543895 | 0.411400 | 6.18 | 6.69E-10 | <0.0001 | 0.1601 | 5962 |
| Left lateral occipital cortex | -2.785693 | 1.443547 | -1.93 | 5.40E-02 | 0.0823 | -0.0501 | 5946 |
| Left lateral orbitofrontal cortex | 0.919700 | 0.669753 | 1.37 | 1.70E-01 | 0.2170 | 0.0354 | 5979 |
| Left lingual cortex | -3.190469 | 1.029361 | -3.10 | 1.90E-03 | 0.0045 | -0.0804 | 5944 |
| Left medial orbitofrontal cortex | 0.126009 | 0.578806 | 0.22 | 8.28E-01 | 0.8438 | 0.0058 | 5841 |
| Left middle temporal cortex | -1.409974 | 0.969228 | -1.45 | 1.46E-01 | 0.1956 | -0.0385 | 5678 |
| Left parahippocampal cortex | 0.355695 | 0.241136 | 1.48 | 1.40E-01 | 0.1915 | 0.0386 | 5897 |
| Left paracentral cortex | 1.708228 | 0.490547 | 3.48 | 5.00E-04 | 0.0014 | 0.0911 | 5838 |
| Left pars opercularis | 0.376587 | 0.668712 | 0.56 | 5.73E-01 | 0.6208 | 0.0146 | 5921 |
| Left pars orbitalis | -0.004380 | 0.208961 | -0.02 | 9.80E-01 | 0.9863 | -0.0005 | 5968 |
| Left pars triangularis | -0.008709 | 0.513868 | -0.02 | 9.86E-01 | 0.9865 | -0.0005 | 5943 |
| Left pericalcarine | -1.844798 | 0.623813 | -2.96 | 3.10E-03 | 0.0070 | -0.0772 | 5888 |
| Left postcentral cortex | 1.531086 | 1.066699 | 1.44 | 1.51E-01 | 0.2012 | 0.0377 | 5838 |
| Left posterior cingulate cortex | 1.133063 | 0.448913 | 2.52 | 1.16E-02 | 0.0215 | 0.0653 | 5965 |
| Left precentral cortex | 2.498491 | 1.218946 | 2.05 | 4.04E-02 | 0.0647 | 0.0535 | 5881 |
| Left precuneus | 2.670410 | 1.058671 | 2.52 | 1.20E-02 | 0.0219 | 0.0654 | 5949 |
| Left rostral anterior cingulate cortex | 1.029638 | 0.384108 | 2.68 | 7.37E-03 | 0.0146 | 0.0703 | 5816 |
| Left rostral middle frontal cortex | -1.385280 | 1.670215 | -0.83 | 4.07E-01 | 0.4663 | -0.0215 | 5952 |
| Left superior frontal cortex | 5.168580 | 1.785377 | 2.89 | 3.81E-03 | 0.0082 | 0.0755 | 5859 |
| Left superior parietal cortex | 3.092192 | 1.547531 | 2.00 | 4.57E-02 | 0.0725 | 0.0522 | 5875 |
| Left superior temporal cortex | 1.667766 | 1.029857 | 1.62 | 1.05E-01 | 0.1514 | 0.0434 | 5577 |
| Left supramarginal cortex | 2.621471 | 1.336164 | 1.96 | 4.98E-02 | 0.0767 | 0.0519 | 5701 |
| Left frontal pole | 0.145064 | 0.092799 | 1.56 | 1.18E-01 | 0.1663 | 0.0403 | 5990 |
| Left temporal pole | -0.046624 | 0.160265 | -0.29 | 7.71E-01 | 0.8071 | -0.0075 | 5954 |
| Left transverse temporal cortex | 0.839195 | 0.193466 | 4.34 | 1.46E-05 | 0.0001 | 0.1122 | 5986 |
| Left insula | 1.796578 | 0.582323 | 3.09 | 2.04E-03 | 0.0048 | 0.0805 | 5899 |
| Right banks of the superior temporal sulcus | 0.090618 | 0.350642 | 0.26 | 7.96E-01 | 0.8277 | 0.0068 | 5804 |
| Right caudal anterior cingulate cortex | 0.428633 | 0.406726 | 1.05 | 2.92E-01 | 0.3499 | 0.0273 | 5930 |
| Right caudal middle frontal cortex | -0.389988 | 0.937912 | -0.42 | 6.78E-01 | 0.7188 | -0.0109 | 5922 |
| Right cuneus | -0.516598 | 0.530357 | -0.97 | 3.30E-01 | 0.3926 | -0.0253 | 5874 |
| Right entorhinal cortex | -0.193848 | 0.220494 | -0.88 | 3.79E-01 | 0.4412 | -0.0238 | 5468 |
| Right fusiform cortex | -0.530713 | 0.936655 | -0.57 | 5.71E-01 | 0.6208 | -0.0149 | 5817 |
| Right inferior parietal cortex | -2.427331 | 1.656241 | -1.47 | 1.43E-01 | 0.1933 | -0.0384 | 5850 |
| Right inferior temporal cortex | -3.402771 | 1.062034 | -3.20 | 1.36E-03 | 0.0034 | -0.0833 | 5911 |
| Right isthmus cingulate cortex | 2.025406 | 0.377433 | 5.37 | 8.34E-08 | <0.0001 | 0.1390 | 5976 |
| Right lateral occipital cortex | -1.673439 | 1.443705 | -1.16 | 2.46E-01 | 0.3023 | -0.0301 | 5962 |
| Right lateral orbitofrontal cortex | 1.563025 | 0.719943 | 2.17 | 3.00E-02 | 0.0495 | 0.0561 | 5984 |
| Right lingual cortex | -2.677391 | 1.012025 | -2.65 | 8.18E-03 | 0.0155 | -0.0688 | 5942 |
| Right medial orbitofrontal cortex | 0.701661 | 0.499300 | 1.41 | 1.60E-01 | 0.2111 | 0.0367 | 5909 |
| Right middle temporal cortex | -1.182137 | 0.942576 | -1.25 | 2.10E-01 | 0.2650 | -0.0327 | 5839 |
| Right parahippocampal cortex | 0.190075 | 0.239932 | 0.79 | 4.28E-01 | 0.4837 | 0.0206 | 5909 |
| Right paracentral cortex | 2.231762 | 0.545410 | 4.09 | 4.34E-05 | 0.0002 | 0.1065 | 5904 |
| Right pars opercularis | 0.391265 | 0.601657 | 0.65 | 5.16E-01 | 0.5660 | 0.0169 | 5897 |
| Right pars orbitalis | -0.191880 | 0.244432 | -0.79 | 4.32E-01 | 0.4845 | -0.0205 | 5964 |
| Right pars triangularis | -0.109654 | 0.603074 | -0.18 | 8.56E-01 | 0.8668 | -0.0047 | 5909 |
| Right pericalcarine | -1.684708 | 0.647078 | -2.60 | 9.25E-03 | 0.0173 | -0.0678 | 5882 |
| Right postcentral cortex | 1.965085 | 1.062275 | 1.85 | 6.44E-02 | 0.0972 | 0.0482 | 5883 |
| Right posterior cingulate cortex | 1.473387 | 0.438905 | 3.36 | 7.90E-04 | 0.0020 | 0.0870 | 5972 |
| Right precentral cortex | 1.749667 | 1.271761 | 1.38 | 1.69E-01 | 0.2170 | 0.0360 | 5894 |
| Right precuneus | 2.948007 | 1.109554 | 2.66 | 7.91E-03 | 0.0153 | 0.0689 | 5959 |
| Right rostral anterior cingulate cortex | 0.847900 | 0.350180 | 2.42 | 1.55E-02 | 0.0267 | 0.0631 | 5894 |
| Right rostral middle frontal cortex | -4.253872 | 1.714127 | -2.48 | 1.31E-02 | 0.0236 | -0.0643 | 5947 |
| Right superior frontal cortex | 5.110005 | 1.807815 | 2.83 | 4.72E-03 | 0.0100 | 0.0737 | 5897 |
| Right superior parietal cortex | 1.617292 | 1.473178 | 1.10 | 2.72E-01 | 0.3314 | 0.0286 | 5903 |
| Right superior temporal cortex | 1.798341 | 0.908284 | 1.98 | 4.80E-02 | 0.0746 | 0.0524 | 5707 |
| Right supramarginal cortex | 2.039680 | 1.244967 | 1.64 | 1.01E-01 | 0.1474 | 0.0432 | 5760 |
| Right frontal pole | 0.038581 | 0.116389 | 0.33 | 7.40E-01 | 0.7800 | 0.0085 | 5989 |
| Right temporal pole | 0.283488 | 0.175380 | 1.62 | 1.06E-01 | 0.1514 | 0.0422 | 5891 |
| Right transverse temporal cortex | 0.306790 | 0.148235 | 2.07 | 3.85E-02 | 0.0625 | 0.0535 | 5991 |
| Right insula | 1.587920 | 0.656578 | 2.42 | 1.60E-02 | 0.0273 | 0.0633 | 5849 |

| **Label** | **Sum Sq** | **Mean Sq** | **F** | **p** | **FDR adjusted p** | **N** |
| --- | --- | --- | --- | --- | --- | --- |
|  |  |  |  |  |  |  |
| **Global measures** |  |  |  |  |  |  |
| Total Intracranial Volume | 907318227560.00 | 453659113780.00 | 20.90 | 8.60E-10 | 1.48E-09 | 5961 |
| Total left hemispheral surface area | 1360267599.00 | 680133799.00 | 14.40 | 5.70E-07 | 8.52E-07 | 6136 |
| Total right hemispheral surface area | 1444063998.00 | 722031999.00 | 15.20 | 2.50E-07 | 3.77E-07 | 6136 |
| Left hemispheral average thickness | 7.10 | 3.60 | 448.00 | <2.00E-16 | <4.19E-16 | 6155 |
| Right hemispheral average thickness | 6.50 | 3.23 | 407.00 | <2.00E-16 | <4.19E-16 | 6154 |
|  |  |  |  |  |  |  |
| **Cortical thickness** |  |  |  |  |  |  |
| Left banks of the superior temporal sulcus | 2.80 | 1.41 | 51.61 | <2.00E-16 | <4.19E-16 | 5826 |
| Left caudal anterior cingulate cortex | 4.00 | 1.84 | 33.50 | 3.20E-15 | 6.13E-15 | 6134 |
| Left caudal middle frontal cortex | 9.50 | 4.80 | 238.00 | <2.00E-16 | <4.19E-16 | 6112 |
| Left cuneus | 5.40 | 2.70 | 164.14 | <2.00E-16 | <4.19E-16 | 6044 |
| Left entorhinal cortex | 2.00 | 0.75 | 6.31 | 1.80E-03 | 2.05E-03 | 5915 |
| Left fusiform cortex | 6.30 | 3.14 | 161.52 | <2.00E-16 | <4.19E-16 | 6125 |
| Left inferior parietal cortex | 6.80 | 3.40 | 216.80 | <2.00E-16 | <4.19E-16 | 6069 |
| Left inferior temporal cortex | 2.40 | 1.20 | 49.00 | <2.00E-16 | <4.19E-16 | 6042 |
| Left isthmus cingulate cortex | 16.80 | 8.40 | 233.71 | <2.00E-16 | <4.19E-16 | 6133 |
| Left lateral occipital cortex | 4.50 | 2.27 | 171.00 | <2.00E-16 | <4.19E-16 | 6116 |
| Left lateral orbitofrontal cortex | 3.30 | 1.65 | 67.95 | <2.00E-16 | <4.19E-16 | 6116 |
| Left lingual cortex | 4.00 | 2.01 | 147.40 | <2.00E-16 | <4.19E-16 | 6103 |
| Left medial orbitofrontal cortex | 1.60 | 0.82 | 34.10 | 1.90E-15 | 3.73E-15 | 6063 |
| Left middle temporal cortex | 5.60 | 2.82 | 113.08 | <2.00E-16 | <4.19E-16 | 5912 |
| Left parahippocampal cortex | 3.00 | 1.60 | 17.39 | 2.90E-08 | 4.46E-08 | 6124 |
| Left paracentral cortex | 21.90 | 10.90 | 520.00 | <2.00E-16 | <4.19E-16 | 6131 |
| Left pars opercularis | 7.20 | 3.60 | 179.84 | <2.00E-16 | <4.19E-16 | 6113 |
| Left pars orbitalis | 6.70 | 3.30 | 72.90 | <2.00E-16 | <4.19E-16 | 6128 |
| Left pars triangularis | 4.40 | 2.20 | 94.90 | <2.00E-16 | <4.19E-16 | 6117 |
| Left pericalcarine | 4.00 | 2.02 | 126.59 | <2.00E-16 | <4.19E-16 | 6039 |
| Left postcentral cortex | 6.30 | 3.20 | 242.00 | <2.00E-16 | <4.19E-16 | 6074 |
| Left posterior cingulate cortex | 13.40 | 6.70 | 288.90 | <2.00E-16 | <4.19E-16 | 6136 |
| Left precentral cortex | 18.80 | 9.40 | 526.00 | <2.00E-16 | <4.19E-16 | 6095 |
| Left precuneus | 9.40 | 4.70 | 312.50 | <2.00E-16 | <4.19E-16 | 6121 |
| Left rostral anterior cingulate cortex | 9.90 | 4.90 | 98.89 | <2.00E-16 | <4.19E-16 | 6104 |
| Left rostral middle frontal cortex | 4.70 | 2.34 | 136.10 | <2.00E-16 | <4.19E-16 | 6125 |
| Left superior frontal cortex | 11.40 | 5.70 | 305.70 | <2.00E-16 | <4.19E-16 | 6121 |
| Left superior parietal cortex | 5.90 | 2.95 | 236.00 | <2.00E-16 | <4.19E-16 | 6088 |
| Left superior temporal cortex | 7.80 | 3.90 | 167.21 | <2.00E-16 | <4.19E-16 | 5798 |
| Left supramarginal cortex | 8.10 | 4.00 | 240.00 | <2.00E-16 | <4.19E-16 | 5977 |
| Left frontal pole | 11.00 | 5.60 | 65.70 | <2.00E-16 | <4.19E-16 | 6149 |
| Left temporal pole | 1.00 | 0.28 | 2.24 | 1.10E-01 | 1.13E-01 | 6111 |
| Left transverse temporal cortex | 13.30 | 6.70 | 149.60 | <2.00E-16 | <4.19E-16 | 6125 |
| Left insula | 5.80 | 2.90 | 120.60 | <2.00E-16 | <4.19E-16 | 6066 |
| Right banks of the superior temporal sulcus | 2.90 | 1.46 | 52.58 | <2.00E-16 | <4.19E-16 | 6014 |
| Right caudal anterior cingulate cortex | 6.20 | 3.12 | 66.48 | <2.00E-16 | <4.19E-16 | 6137 |
| Right caudal middle frontal cortex | 7.90 | 3.90 | 199.80 | <2.00E-16 | <4.19E-16 | 6120 |
| Right cuneus | 6.10 | 3.07 | 181.58 | <2.00E-16 | <4.19E-16 | 6048 |
| Right entorhinal cortex | 1.00 | 0.50 | 3.64 | 2.60E-02 | 2.78E-02 | 5859 |
| Right fusiform cortex | 6.30 | 3.17 | 157.90 | <2.00E-16 | <4.19E-16 | 6130 |
| Right inferior parietal cortex | 7.60 | 3.80 | 253.00 | <2.00E-16 | <4.19E-16 | 6073 |
| Right inferior temporal cortex | 1.10 | 0.54 | 21.04 | 7.80E-10 | 1.36E-09 | 6076 |
| Right isthmus cingulate cortex | 17.10 | 8.50 | 250.60 | <2.00E-16 | <4.19E-16 | 6133 |
| Right lateral occipital cortex | 6.80 | 3.39 | 237.90 | <2.00E-16 | <4.19E-16 | 6120 |
| Right lateral orbitofrontal cortex | 2.70 | 1.33 | 54.70 | <2.00E-16 | <4.19E-16 | 6132 |
| Right lingual cortex | 3.70 | 1.84 | 133.97 | <2.00E-16 | <4.19E-16 | 6088 |
| Right medial orbitofrontal cortex | 4.00 | 2.01 | 78.63 | <2.00E-16 | <4.19E-16 | 6086 |
| Right middle temporal cortex | 1.70 | 0.85 | 34.92 | 8.40E-16 | 1.69E-15 | 6061 |
| Right parahippocampal cortex | 5.00 | 2.64 | 38.83 | <2.00E-16 | <4.19E-16 | 6129 |
| Right paracentral cortex | 18.90 | 9.50 | 448.00 | <2.00E-16 | <4.19E-16 | 6135 |
| Right pars opercularis | 7.80 | 3.90 | 179.73 | <2.00E-16 | <4.19E-16 | 6091 |
| Right pars orbitalis | 4.60 | 2.32 | 53.30 | <2.00E-16 | <4.19E-16 | 6126 |
| Right pars triangularis | 4.90 | 2.43 | 108.20 | <2.00E-16 | <4.19E-16 | 6090 |
| Right pericalcarine | 3.00 | 1.49 | 91.40 | <2.00E-16 | <4.19E-16 | 6021 |
| Right postcentral cortex | 6.40 | 3.20 | 243.00 | <2.00E-16 | <4.19E-16 | 6094 |
| Right posterior cingulate cortex | 14.10 | 7.00 | 325.00 | <2.00E-16 | <4.19E-16 | 6139 |
| Right precentral cortex | 16.50 | 8.30 | 453.00 | <2.00E-16 | <4.19E-16 | 6103 |
| Right precuneus | 10.00 | 5.00 | 339.60 | <2.00E-16 | <4.19E-16 | 6123 |
| Right rostral anterior cingulate cortex | 2.70 | 1.37 | 28.39 | 5.30E-13 | 9.91E-13 | 6107 |
| Right rostral middle frontal cortex | 2.80 | 1.40 | 82.40 | <2.00E-16 | <4.19E-16 | 6118 |
| Right superior frontal cortex | 9.60 | 4.80 | 256.90 | <2.00E-16 | <4.19E-16 | 6126 |
| Right superior parietal cortex | 6.50 | 3.30 | 260.00 | <2.00E-16 | <4.19E-16 | 6105 |
| Right superior temporal cortex | 4.50 | 2.27 | 100.34 | <2.00E-16 | <4.19E-16 | 5930 |
| Right supramarginal cortex | 7.50 | 3.80 | 223.00 | <2.00E-16 | <4.19E-16 | 5994 |
| Right frontal pole | 9.00 | 4.70 | 56.60 | <2.00E-16 | <4.19E-16 | 6145 |
| Right temporal pole | 0.00 | 0.13 | 0.98 | 3.77E-01 | 3.80E-01 | 6116 |
| Right transverse temporal cortex | 12.70 | 6.30 | 128.48 | <2.00E-16 | <4.19E-16 | 6116 |
| Right insula | 7.20 | 3.60 | 133.22 | <2.00E-16 | <4.19E-16 | 6014 |
|  |  |  |  |  |  |  |
| **Subcortical volumes** |  |  |  |  |  |  |
| Left lateral ventricle | 3712699510.00 | 1856349755.00 | 95.30 | <2.00E-16 | <4.19E-16 | 5840 |
| Right lateral ventricle | 2649436617.00 | 1324718308.00 | 78.87 | <2.00E-16 | <4.19E-16 | 5825 |
| Left thalamus | 80097392.00 | 40048696.00 | 89.40 | <2.00E-16 | <4.19E-16 | 5820 |
| Right thalamus | 5128585.00 | 2564293.00 | 7.77 | 4.30E-04 | 5.23E-04 | 5806 |
| Left caudate | 22481676.00 | 11240838.00 | 73.09 | <2.00E-16 | <4.19E-16 | 5817 |
| Right caudate | 17296660.00 | 8648330.00 | 55.58 | <2.00E-16 | <4.19E-16 | 5763 |
| Left putamen | 109883479.00 | 54941739.00 | 201.29 | <2.00E-16 | <4.19E-16 | 5622 |
| Right putamen | 87687967.00 | 43843983.00 | 182.06 | <2.00E-16 | <4.19E-16 | 5691 |
| Left pallidum | 1414895.00 | 707447.00 | 18.38 | 1.10E-08 | 1.76E-08 | 5551 |
| Right pallidum | 366687.00 | 183344.00 | 5.69 | 3.40E-03 | 3.79E-03 | 5774 |
| Left hippocampus | 19596420.00 | 9798210.00 | 77.00 | <2.00E-16 | <4.19E-16 | 5792 |
| Right hippocamups | 9021270.00 | 4510635.00 | 35.45 | 5.00E-16 | 1.02E-15 | 5843 |
| Left amygdala | 1070887.00 | 535444.00 | 18.86 | 6.80E-09 | 1.12E-08 | 5805 |
| Right amygdala | 533078.00 | 266539.00 | 8.26 | 2.60E-04 | 3.19E-04 | 5816 |
| Left nucleus accumbens | 1104995.00 | 552498.00 | 70.70 | <2.00E-16 | <4.19E-16 | 5705 |
| Right nucleus accumbens | 4246199.00 | 2123099.00 | 315.10 | <2.00E-16 | <4.19E-16 | 5728 |
|  |  |  |  |  |  |  |
| **Cortical surface area** |  |  |  |  |  |  |
| Left banks of the superior temporal sulcus | 293588.00 | 146794.00 | 7.07 | 8.60E-04 | 1.02E-03 | 5415 |
| Left caudal anterior cingulate cortex | 260225.00 | 130112.00 | 7.65 | 4.80E-04 | 5.80E-04 | 5753 |
| Left caudal middle frontal cortex | 564782.00 | 282391.00 | 2.63 | 7.20E-02 | 7.54E-02 | 5753 |
| Left cuneus | 751642.00 | 375821.00 | 9.92 | 5.00E-05 | 6.60E-05 | 5708 |
| Left entorhinal cortex | 102651.00 | 51325.00 | 9.72 | 6.13E-05 | 8.02E-05 | 5404 |
| Left fusiform cortex | 8245791.00 | 4122896.00 | 38.80 | <2.00E-16 | <4.19E-16 | 5648 |
| Left inferior parietal cortex | 14731378.00 | 7365689.00 | 26.70 | 2.90E-12 | 5.23E-12 | 5702 |
| Left inferior temporal cortex | 5249768.00 | 2624884.00 | 17.50 | 2.72E-08 | 4.23E-08 | 5725 |
| Left isthmus cingulate cortex | 1461113.00 | 730557.00 | 35.60 | 4.10E-16 | 8.47E-16 | 5811 |
| Left lateral occipital cortex | 4793649.00 | 2396825.00 | 9.56 | 7.20E-05 | 9.19E-05 | 5795 |
| Left lateral orbitofrontal cortex | 692123.00 | 346062.00 | 6.35 | 1.80E-03 | 2.05E-03 | 5828 |
| Left lingual cortex | 3470138.00 | 1735069.00 | 13.70 | 1.20E-06 | 1.71E-06 | 5793 |
| Left medial orbitofrontal cortex | 1103309.00 | 551655.00 | 13.80 | 1.09E-06 | 1.56E-06 | 5690 |
| Left middle temporal cortex | 5903263.00 | 2951631.00 | 27.80 | 9.30E-13 | 1.72E-12 | 5527 |
| Left parahippocampal cortex | 270846.00 | 135423.00 | 19.50 | 3.70E-09 | 6.18E-09 | 5746 |
| Left paracentral cortex | 693452.00 | 346726.00 | 12.15 | 5.40E-06 | 7.50E-06 | 5687 |
| Left pars opercularis | 458387.00 | 229194.00 | 4.25 | 1.40E-02 | 1.52E-02 | 5770 |
| Left pars orbitalis | 188464.00 | 94232.00 | 17.82 | 1.93E-08 | 3.03E-08 | 5817 |
| Left pars triangularis | 989845.00 | 494923.00 | 15.47 | 2.00E-07 | 3.05E-07 | 5792 |
| Left pericalcarine | 592949.00 | 296475.00 | 6.36 | 1.70E-03 | 1.96E-03 | 5737 |
| Left postcentral cortex | 3647169.00 | 1823585.00 | 13.50 | 1.40E-06 | 1.98E-06 | 5687 |
| Left posterior cingulate cortex | 84839.00 | 42420.00 | 1.73 | 1.80E-01 | 1.84E-01 | 5814 |
| Left precentral cortex | 12106427.00 | 6053214.00 | 33.90 | 2.20E-15 | 4.26E-15 | 5730 |
| Left precuneus | 3134238.00 | 1567119.00 | 11.60 | 9.19E-06 | 1.25E-05 | 5798 |
| Left rostral anterior cingulate cortex | 208683.00 | 104342.00 | 5.97 | 2.60E-03 | 2.92E-03 | 5665 |
| Left rostral middle frontal cortex | 12313546.00 | 6156773.00 | 18.10 | 1.50E-08 | 2.38E-08 | 5801 |
| Left superior frontal cortex | 3664559.00 | 1832280.00 | 4.81 | 8.20E-03 | 8.94E-03 | 5708 |
| Left superior parietal cortex | 1824913.00 | 912457.00 | 3.20 | 4.10E-02 | 4.32E-02 | 5724 |
| Left superior temporal cortex | 2568279.00 | 1284139.00 | 10.70 | 2.26E-05 | 3.01E-05 | 5426 |
| Left supramarginal cortex | 4039178.00 | 2019589.00 | 9.70 | 6.30E-05 | 8.17E-05 | 5550 |
| Left frontal pole | 17981.00 | 8990.00 | 8.57 | 1.90E-04 | 2.37E-04 | 5839 |
| Left temporal pole | 9040.00 | 4520.00 | 1.46 | 2.30E-01 | 2.33E-01 | 5803 |
| Left transverse temporal cortex | 127505.00 | 63753.00 | 14.10 | 8.10E-07 | 1.19E-06 | 5835 |
| Left insula | 1995596.00 | 997798.00 | 24.70 | 2.10E-11 | 3.75E-11 | 5748 |
| Right banks of the superior temporal sulcus | 784286.00 | 392143.00 | 27.10 | 2.00E-12 | 3.65E-12 | 5653 |
| Right caudal anterior cingulate cortex | 1113.00 | 557.00 | 0.03 | 9.73E-01 | 9.73E-01 | 5779 |
| Right caudal middle frontal cortex | 1471804.00 | 735902.00 | 6.92 | 1.00E-03 | 1.17E-03 | 5771 |
| Right cuneus | 1442073.00 | 721036.00 | 21.70 | 4.24E-10 | 7.48E-10 | 5723 |
| Right entorhinal cortex | 26359.00 | 13179.00 | 2.45 | 8.70E-02 | 8.99E-02 | 5317 |
| Right fusiform cortex | 10562634.00 | 5281317.00 | 51.40 | <2.00E-16 | <4.19E-16 | 5666 |
| Right inferior parietal cortex | 6291315.00 | 3145657.00 | 9.63 | 6.70E-05 | 8.62E-05 | 5699 |
| Right inferior temporal cortex | 7742151.00 | 3871076.00 | 28.90 | 3.40E-13 | 6.43E-13 | 5760 |
| Right isthmus cingulate cortex | 695756.00 | 347878.00 | 20.00 | 2.15E-09 | 3.63E-09 | 5825 |
| Right lateral occipital cortex | 7180183.00 | 3590091.00 | 14.20 | 7.10E-07 | 1.05E-06 | 5811 |
| Right lateral orbitofrontal cortex | 499777.00 | 249888.00 | 3.96 | 1.90E-02 | 2.04E-02 | 5833 |
| Right lingual cortex | 1524892.00 | 762446.00 | 6.18 | 2.10E-03 | 2.37E-03 | 5791 |
| Right medial orbitofrontal cortex | 832573.00 | 416287.00 | 13.94 | 9.20E-07 | 1.34E-06 | 5758 |
| Right middle temporal cortex | 1966539.00 | 983269.00 | 9.34 | 8.90E-05 | 1.13E-04 | 5688 |
| Right parahippocampal cortex | 483361.00 | 241680.00 | 34.80 | 9.40E-16 | 1.87E-15 | 5758 |
| Right paracentral cortex | 3254822.00 | 1627411.00 | 45.20 | <2.00E-16 | <4.19E-16 | 5753 |
| Right pars opercularis | 1024258.00 | 512129.00 | 11.93 | 6.80E-06 | 9.36E-06 | 5746 |
| Right pars orbitalis | 97880.00 | 48940.00 | 6.72 | 1.20E-03 | 1.40E-03 | 5813 |
| Right pars triangularis | 746848.00 | 373424.00 | 8.55 | 2.00E-04 | 2.47E-04 | 5758 |
| Right pericalcarine | 915239.00 | 457619.00 | 9.09 | 1.10E-04 | 1.38E-04 | 5731 |
| Right postcentral cortex | 3060541.00 | 1530270.00 | 11.40 | 1.10E-05 | 1.49E-05 | 5732 |
| Right posterior cingulate cortex | 326225.00 | 163113.00 | 6.96 | 9.60E-04 | 1.13E-03 | 5821 |
| Right precentral cortex | 16972177.00 | 8486089.00 | 43.70 | <2.00E-16 | <4.19E-16 | 5743 |
| Right precuneus | 1490434.00 | 745217.00 | 5.02 | 6.70E-03 | 7.36E-03 | 5808 |
| Right rostral anterior cingulate cortex | 544536.00 | 272268.00 | 18.40 | 1.00E-08 | 1.62E-08 | 5743 |
| Right rostral middle frontal cortex | 13342836.00 | 6671418.00 | 18.80 | 7.60E-09 | 1.24E-08 | 5796 |
| Right superior frontal cortex | 5799316.00 | 2899658.00 | 7.40 | 6.10E-04 | 7.31E-04 | 5746 |
| Right superior parietal cortex | 1860777.00 | 930388.00 | 3.56 | 2.80E-02 | 2.97E-02 | 5752 |
| Right superior temporal cortex | 2447612.00 | 1223806.00 | 12.70 | 3.01E-06 | 4.22E-06 | 5556 |
| Right supramarginal cortex | 4017110.00 | 2008555.00 | 11.00 | 1.80E-05 | 2.42E-05 | 5609 |
| Right frontal pole | 8529.00 | 4265.00 | 2.57 | 7.60E-02 | 7.90E-02 | 5838 |
| Right temporal pole | 40013.00 | 20007.00 | 5.42 | 4.40E-03 | 4.86E-03 | 5740 |
| Right transverse temporal cortex | 108280.00 | 54140.00 | 20.33 | 1.60E-09 | 2.73E-09 | 5840 |
| Right insula | 6063520.00 | 3031760.00 | 58.97 | <2.00E-16 | <4.19E-16 | 5698 |

| **Label** | **Estimate** | **StdError** | **T** | **p** | **FDR adjusted p** | **Cohen´s d** | **N** |
| --- | --- | --- | --- | --- | --- | --- | --- |
|  |  |  |  |  |  |  |  |
| **Global measures** |  |  |  |  |  |  |  |
| Total Intracranial Volume | -994.000000 | 823.000000 | -1.21 | 2.27E-01 | 0.4261 | -0.0642 | 1421 |
| Total left hemispheral surface area | -31.100000 | 40.200000 | -0.77 | 4.39E-01 | 0.6308 | -0.0413 | 1393 |
| Total right hemispheral surface area | -30.600000 | 40.600000 | -0.75 | 4.51E-01 | 0.6322 | -0.0402 | 1393 |
| Left hemispheral average thickness | -0.001612 | 0.000508 | -3.17 | 1.55E-03 | 0.0128 | -0.1700 | 1393 |
| Right hemispheral average thickness | -0.001586 | 0.000509 | -3.12 | 1.90E-03 | 0.0142 | -0.1673 | 1393 |
|  |  |  |  |  |  |  |  |
| **Cortical thickness** |  |  |  |  |  |  |  |
| Left banks of the superior temporal sulcus | -0.002623 | 0.001020 | -2.57 | 1.00E-02 | 0.0604 | -0.1445 | 1267 |
| Left caudal anterior cingulate cortex | -0.001688 | 0.001382 | -1.22 | 2.22E-01 | 0.4261 | -0.0656 | 1385 |
| Left caudal middle frontal cortex | -0.002583 | 0.000784 | -3.29 | 1.00E-03 | 0.0098 | -0.1766 | 1390 |
| Left cuneus | -0.001004 | 0.000833 | -1.21 | 2.28E-01 | 0.4261 | -0.0654 | 1371 |
| Left entorhinal cortex | -0.003681 | 0.002358 | -1.56 | 1.20E-01 | 0.2944 | -0.0874 | 1275 |
| Left fusiform cortex | -0.003417 | 0.000848 | -4.03 | 5.80E-05 | 0.0019 | -0.2163 | 1390 |
| Left inferior parietal cortex | -0.002758 | 0.000712 | -3.88 | 1.10E-04 | 0.0019 | -0.2092 | 1378 |
| Left inferior temporal cortex | -0.003870 | 0.000969 | -4 | 6.81E-05 | 0.0019 | -0.2162 | 1371 |
| Left isthmus cingulate cortex | -0.002397 | 0.001204 | -1.99 | 4.70E-02 | 0.1573 | -0.1069 | 1389 |
| Left lateral occipital cortex | -0.001674 | 0.000726 | -2.31 | 2.10E-02 | 0.0999 | -0.1240 | 1390 |
| Left lateral orbitofrontal cortex | -0.000810 | 0.000880 | -0.92 | 3.58E-01 | 0.5565 | -0.0493 | 1393 |
| Left lingual cortex | -0.000549 | 0.000721 | -0.76 | 4.46E-01 | 0.6308 | -0.0410 | 1379 |
| Left medial orbitofrontal cortex | -0.000585 | 0.000916 | -0.64 | 5.23E-01 | 0.6777 | -0.0344 | 1388 |
| Left middle temporal cortex | -0.003440 | 0.000997 | -3.45 | 5.80E-04 | 0.0083 | -0.1905 | 1314 |
| Left parahippocampal cortex | -0.004245 | 0.001775 | -2.39 | 1.69E-02 | 0.0858 | -0.1284 | 1388 |
| Left paracentral cortex | -0.001315 | 0.000833 | -1.58 | 1.15E-01 | 0.2866 | -0.0848 | 1389 |
| Left pars opercularis | -0.001664 | 0.000782 | -2.13 | 3.40E-02 | 0.1369 | -0.1144 | 1389 |
| Left pars orbitalis | -0.001183 | 0.001209 | -0.98 | 3.28E-01 | 0.5361 | -0.0526 | 1393 |
| Left pars triangularis | 0.000621 | 0.000873 | 0.71 | 4.77E-01 | 0.6441 | 0.0381 | 1392 |
| Left pericalcarine | 0.001743 | 0.000797 | 2.19 | 2.90E-02 | 0.1265 | 0.1187 | 1364 |
| Left postcentral cortex | 0.000853 | 0.000692 | 1.23 | 2.18E-01 | 0.4261 | 0.0665 | 1372 |
| Left posterior cingulate cortex | -0.001021 | 0.000887 | -1.15 | 2.50E-01 | 0.4408 | -0.0617 | 1390 |
| Left precentral cortex | -0.002315 | 0.000686 | -3.38 | 7.50E-04 | 0.0098 | -0.1819 | 1383 |
| Left precuneus | -0.001116 | 0.000707 | -1.58 | 1.15E-01 | 0.2866 | -0.0848 | 1390 |
| Left rostral anterior cingulate cortex | -0.002686 | 0.001336 | -2.01 | 4.50E-02 | 0.1573 | -0.1081 | 1386 |
| Left rostral middle frontal cortex | -0.000975 | 0.000700 | -1.39 | 1.64E-01 | 0.3656 | -0.0746 | 1390 |
| Left superior frontal cortex | -0.001428 | 0.000746 | -1.91 | 5.60E-02 | 0.1758 | -0.1026 | 1388 |
| Left superior parietal cortex | -0.000785 | 0.000651 | -1.21 | 2.28E-01 | 0.4261 | -0.0650 | 1389 |
| Left superior temporal cortex | -0.002567 | 0.000957 | -2.68 | 7.40E-03 | 0.0465 | -0.1511 | 1260 |
| Left supramarginal cortex | -0.001198 | 0.000742 | -1.61 | 1.07E-01 | 0.2775 | -0.0884 | 1329 |
| Left frontal pole | -0.001845 | 0.001772 | -1.04 | 2.98E-01 | 0.4925 | -0.0558 | 1392 |
| Left temporal pole | -0.003942 | 0.002547 | -1.55 | 1.22E-01 | 0.2947 | -0.0831 | 1393 |
| Left transverse temporal cortex | -0.005094 | 0.001247 | -4.08 | 4.70E-05 | 0.0019 | -0.2189 | 1391 |
| Left insula | -0.001691 | 0.000927 | -1.82 | 6.85E-02 | 0.2030 | -0.0990 | 1353 |
| Right banks of the superior temporal sulcus | -0.003029 | 0.001026 | -2.95 | 3.20E-03 | 0.0228 | -0.1616 | 1335 |
| Right caudal anterior cingulate cortex | -0.001059 | 0.001369 | -0.77 | 4.40E-01 | 0.6308 | -0.0414 | 1388 |
| Right caudal middle frontal cortex | -0.002046 | 0.000805 | -2.54 | 1.10E-02 | 0.0640 | -0.1364 | 1390 |
| Right cuneus | 0.000491 | 0.000890 | 0.55 | 5.81E-01 | 0.7297 | 0.0299 | 1358 |
| Right entorhinal cortex | -0.001070 | 0.002550 | -0.42 | 6.70E-01 | 0.8092 | -0.0237 | 1253 |
| Right fusiform cortex | -0.004164 | 0.000859 | -4.85 | 1.40E-06 | 0.0002 | -0.2605 | 1389 |
| Right inferior parietal cortex | -0.000805 | 0.000715 | -1.13 | 2.61E-01 | 0.4445 | -0.0610 | 1373 |
| Right inferior temporal cortex | -0.003603 | 0.000997 | -3.61 | 3.10E-04 | 0.0049 | -0.1941 | 1385 |
| Right isthmus cingulate cortex | -0.002387 | 0.001201 | -1.99 | 4.71E-02 | 0.1573 | -0.1068 | 1391 |
| Right lateral occipital cortex | -0.000365 | 0.000744 | -0.49 | 6.24E-01 | 0.7712 | -0.0263 | 1391 |
| Right lateral orbitofrontal cortex | -0.001739 | 0.000904 | -1.92 | 5.50E-02 | 0.1758 | -0.1030 | 1393 |
| Right lingual cortex | -0.000679 | 0.000755 | -0.9 | 3.69E-01 | 0.5680 | -0.0486 | 1374 |
| Right medial orbitofrontal cortex | -0.003031 | 0.000942 | -3.22 | 1.30E-03 | 0.0120 | -0.1729 | 1390 |
| Right middle temporal cortex | -0.002020 | 0.001010 | -2 | 4.52E-02 | 0.1573 | -0.1078 | 1378 |
| Right parahippocampal cortex | -0.003453 | 0.001606 | -2.15 | 3.17E-02 | 0.1322 | -0.1153 | 1392 |
| Right paracentral cortex | -0.002130 | 0.000843 | -2.53 | 1.17E-02 | 0.0656 | -0.1359 | 1388 |
| Right pars opercularis | -0.001807 | 0.000803 | -2.25 | 2.50E-02 | 0.1121 | -0.1207 | 1392 |
| Right pars orbitalis | -0.002483 | 0.001225 | -2.03 | 4.28E-02 | 0.1573 | -0.1089 | 1392 |
| Right pars triangularis | -0.000530 | 0.000841 | -0.63 | 5.29E-01 | 0.6777 | -0.0338 | 1392 |
| Right pericalcarine | 0.001197 | 0.000873 | 1.37 | 1.70E-01 | 0.3656 | 0.0746 | 1352 |
| Right postcentral cortex | -0.000013 | 0.000708 | -0.02 | 9.90E-01 | 0.9900 | -0.0011 | 1375 |
| Right posterior cingulate cortex | -0.001829 | 0.000903 | -2.02 | 4.31E-02 | 0.1573 | -0.1083 | 1393 |
| Right precentral cortex | -0.002418 | 0.000726 | -3.33 | 8.90E-04 | 0.0098 | -0.1793 | 1381 |
| Right precuneus | -0.002062 | 0.000727 | -2.83 | 4.70E-03 | 0.0321 | -0.1519 | 1390 |
| Right rostral anterior cingulate cortex | -0.003202 | 0.001330 | -2.41 | 1.62E-02 | 0.0845 | -0.1296 | 1386 |
| Right rostral middle frontal cortex | -0.002689 | 0.000687 | -3.91 | 9.50E-05 | 0.0019 | -0.2099 | 1390 |
| Right superior frontal cortex | -0.002478 | 0.000750 | -3.3 | 9.80E-04 | 0.0098 | -0.1771 | 1391 |
| Right superior parietal cortex | -0.000056 | 0.000677 | -0.08 | 9.30E-01 | 0.9645 | -0.0043 | 1386 |
| Right superior temporal cortex | -0.001653 | 0.000934 | -1.77 | 7.70E-02 | 0.2187 | -0.0980 | 1306 |
| Right supramarginal cortex | -0.000931 | 0.000742 | -1.26 | 2.09E-01 | 0.4261 | -0.0690 | 1335 |
| Right frontal pole | -0.003257 | 0.001638 | -1.99 | 4.69E-02 | 0.1573 | -0.1068 | 1392 |
| Right temporal pole | 0.000264 | 0.002692 | 0.1 | 9.22E-01 | 0.9645 | 0.0054 | 1389 |
| Right transverse temporal cortex | -0.004905 | 0.001248 | -3.93 | 8.90E-05 | 0.0019 | -0.2108 | 1392 |
| Right insula | -0.000736 | 0.001022 | -0.72 | 4.71E-01 | 0.6441 | -0.0397 | 1318 |
|  |  |  |  |  |  |  |  |
| **Subcortical volumes** |  |  |  |  |  |  |  |
| Left lateral ventricle | -28.125555 | 23.914490 | -1.18 | 2.40E-01 | 0.4360 | -0.0626 | 1421 |
| Right lateral ventricle | -27.634246 | 24.252891 | -1.14 | 2.55E-01 | 0.4408 | -0.0605 | 1421 |
| Left thalamus | 18.081920 | 4.630190 | 3.91 | 9.86E-05 | 0.0019 | 0.2077 | 1420 |
| Right thalamus | 11.079574 | 3.349880 | 3.31 | 9.70E-04 | 0.0098 | 0.1757 | 1421 |
| Left caudate | -3.096568 | 2.413775 | -1.28 | 2.00E-01 | 0.4132 | -0.0683 | 1407 |
| Right caudate | -3.357384 | 2.443735 | -1.37 | 1.70E-01 | 0.3656 | -0.0729 | 1415 |
| Left putamen | 0.514457 | 3.376406 | 0.15 | 8.80E-01 | 0.9272 | 0.0081 | 1359 |
| Right putamen | -0.826692 | 2.906402 | -0.28 | 7.80E-01 | 0.8747 | -0.0150 | 1387 |
| Left pallidum | -1.387837 | 1.219848 | -1.14 | 2.56E-01 | 0.4408 | -0.0645 | 1252 |
| Right pallidum | -0.610559 | 0.959734 | -0.64 | 5.25E-01 | 0.6777 | -0.0344 | 1386 |
| Left hippocampus | 3.697942 | 2.298007 | 1.61 | 1.08E-01 | 0.2775 | 0.0858 | 1409 |
| Right hippocamups | 7.205584 | 2.264027 | 3.18 | 1.50E-03 | 0.0128 | 0.1693 | 1413 |
| Left amygdala | 2.569655 | 1.126227 | 2.28 | 2.27E-02 | 0.1048 | 0.1221 | 1396 |
| Right amygdala | 5.026768 | 1.160731 | 4.33 | 1.59E-05 | 0.0013 | 0.2317 | 1399 |
| Left nucleus accumbens | 1.275868 | 0.606778 | 2.1 | 3.60E-02 | 0.1413 | 0.1127 | 1392 |
| Right nucleus accumbens | -0.605353 | 0.518066 | -1.17 | 2.43E-01 | 0.4360 | -0.0624 | 1410 |
|  |  |  |  |  |  |  |  |
| **Cortical surface area** |  |  |  |  |  |  |  |
| Left banks of the superior temporal sulcus | -1.208966 | 0.911148 | -1.33 | 1.85E-01 | 0.3868 | -0.0748 | 1267 |
| Left caudal anterior cingulate cortex | -0.552093 | 0.778509 | -0.71 | 4.80E-01 | 0.6441 | -0.0382 | 1385 |
| Left caudal middle frontal cortex | 1.922332 | 1.993409 | 0.96 | 3.35E-01 | 0.5422 | 0.0515 | 1390 |
| Left cuneus | -0.850767 | 1.258527 | -0.68 | 4.99E-01 | 0.6636 | -0.0368 | 1371 |
| Left entorhinal cortex | -0.361675 | 0.433019 | -0.84 | 4.04E-01 | 0.6041 | -0.0471 | 1275 |
| Left fusiform cortex | -3.533138 | 1.920878 | -1.84 | 6.60E-02 | 0.1993 | -0.0988 | 1390 |
| Left inferior parietal cortex | 0.113594 | 3.063455 | 0.04 | 9.70E-01 | 0.9784 | 0.0022 | 1378 |
| Left inferior temporal cortex | -3.812783 | 2.276812 | -1.67 | 9.42E-02 | 0.2550 | -0.0903 | 1371 |
| Left isthmus cingulate cortex | 2.882405 | 0.914783 | 3.15 | 1.66E-03 | 0.0130 | 0.1692 | 1389 |
| Left lateral occipital cortex | 1.853419 | 2.958576 | 0.63 | 5.31E-01 | 0.6777 | 0.0338 | 1390 |
| Left lateral orbitofrontal cortex | 1.122054 | 1.287377 | 0.87 | 3.84E-01 | 0.5853 | 0.0467 | 1393 |
| Left lingual cortex | -0.900964 | 2.311712 | -0.39 | 6.97E-01 | 0.8241 | -0.0210 | 1379 |
| Left medial orbitofrontal cortex | -0.426374 | 1.240626 | -0.34 | 7.30E-01 | 0.8450 | -0.0183 | 1388 |
| Left middle temporal cortex | -2.269602 | 1.926604 | -1.18 | 2.39E-01 | 0.4360 | -0.0652 | 1314 |
| Left parahippocampal cortex | -0.110804 | 0.519390 | -0.21 | 8.31E-01 | 0.9054 | -0.0113 | 1388 |
| Left paracentral cortex | 2.979731 | 1.059048 | 2.81 | 5.00E-03 | 0.0327 | 0.1509 | 1389 |
| Left pars opercularis | 2.607901 | 1.403670 | 1.86 | 6.34E-02 | 0.1951 | 0.0999 | 1389 |
| Left pars orbitalis | -0.176221 | 0.402191 | -0.44 | 6.61E-01 | 0.8049 | -0.0236 | 1393 |
| Left pars triangularis | -0.303021 | 1.046436 | -0.29 | 7.72E-01 | 0.8722 | -0.0156 | 1392 |
| Left pericalcarine | -1.385585 | 1.326410 | -1.04 | 2.96E-01 | 0.4925 | -0.0564 | 1364 |
| Left postcentral cortex | 0.481956 | 2.108151 | 0.23 | 8.19E-01 | 0.8992 | 0.0124 | 1372 |
| Left posterior cingulate cortex | 1.300106 | 0.970793 | 1.34 | 1.81E-01 | 0.3834 | 0.0719 | 1390 |
| Left precentral cortex | 3.804425 | 2.539141 | 1.5 | 1.34E-01 | 0.3094 | 0.0807 | 1383 |
| Left precuneus | 2.941163 | 2.085839 | 1.41 | 1.60E-01 | 0.3641 | 0.0757 | 1390 |
| Left rostral anterior cingulate cortex | -0.025770 | 0.740185 | -0.03 | 9.72E-01 | 0.9784 | -0.0016 | 1386 |
| Left rostral middle frontal cortex | -6.907822 | 3.209077 | -2.15 | 3.20E-02 | 0.1322 | -0.1154 | 1390 |
| Left superior frontal cortex | -0.697240 | 3.353830 | -0.21 | 8.40E-01 | 0.9054 | -0.0113 | 1388 |
| Left superior parietal cortex | 4.181456 | 3.041960 | 1.37 | 1.69E-01 | 0.3656 | 0.0736 | 1389 |
| Left superior temporal cortex | -0.169611 | 2.039716 | -0.08 | 9.34E-01 | 0.9645 | -0.0045 | 1260 |
| Left supramarginal cortex | 1.827798 | 2.554055 | 0.72 | 4.74E-01 | 0.6441 | 0.0395 | 1329 |
| Left frontal pole | 0.007286 | 0.181751 | 0.04 | 9.68E-01 | 0.9784 | 0.0021 | 1392 |
| Left temporal pole | -0.379195 | 0.347907 | -1.09 | 2.76E-01 | 0.4658 | -0.0585 | 1393 |
| Left transverse temporal cortex | 0.751465 | 0.390099 | 1.93 | 5.43E-02 | 0.1758 | 0.1036 | 1391 |
| Left insula | 0.247309 | 1.240255 | 0.2 | 8.42E-01 | 0.9054 | 0.0109 | 1353 |
| Right banks of the superior temporal sulcus | 0.723377 | 0.773291 | 0.94 | 3.50E-01 | 0.5495 | 0.0515 | 1335 |
| Right caudal anterior cingulate cortex | 0.337475 | 0.885994 | 0.38 | 7.03E-01 | 0.8241 | 0.0204 | 1388 |
| Right caudal middle frontal cortex | -0.921923 | 1.956029 | -0.47 | 6.40E-01 | 0.7850 | -0.0252 | 1390 |
| Right cuneus | -0.274341 | 1.152187 | -0.24 | 8.12E-01 | 0.8976 | -0.0130 | 1358 |
| Right entorhinal cortex | -0.080282 | 0.478771 | -0.17 | 8.67E-01 | 0.9259 | -0.0096 | 1253 |
| Right fusiform cortex | -1.784347 | 1.891202 | -0.94 | 3.46E-01 | 0.5481 | -0.0505 | 1389 |
| Right inferior parietal cortex | -2.082240 | 3.357600 | -0.62 | 5.35E-01 | 0.6777 | -0.0335 | 1373 |
| Right inferior temporal cortex | -3.743328 | 2.278399 | -1.64 | 1.01E-01 | 0.2688 | -0.0882 | 1385 |
| Right isthmus cingulate cortex | 1.433508 | 0.817047 | 1.75 | 7.96E-02 | 0.2192 | 0.0939 | 1391 |
| Right lateral occipital cortex | 1.243378 | 2.970906 | 0.42 | 6.76E-01 | 0.8102 | 0.0225 | 1391 |
| Right lateral orbitofrontal cortex | 1.051038 | 1.479037 | 0.71 | 4.77E-01 | 0.6441 | 0.0381 | 1393 |
| Right lingual cortex | -2.782864 | 2.255558 | -1.23 | 2.18E-01 | 0.4261 | -0.0664 | 1374 |
| Right medial orbitofrontal cortex | 0.789335 | 0.928268 | 0.85 | 3.95E-01 | 0.5967 | 0.0456 | 1390 |
| Right middle temporal cortex | 0.642372 | 2.020875 | 0.32 | 7.51E-01 | 0.8569 | 0.0173 | 1378 |
| Right parahippocampal cortex | -0.197338 | 0.517732 | -0.38 | 7.03E-01 | 0.8241 | -0.0204 | 1392 |
| Right paracentral cortex | 1.102048 | 1.152293 | 0.96 | 3.40E-01 | 0.5447 | 0.0516 | 1388 |
| Right pars opercularis | 0.200402 | 1.255376 | 0.16 | 8.73E-01 | 0.9263 | 0.0086 | 1392 |
| Right pars orbitalis | -0.597971 | 0.513458 | -1.16 | 2.44E-01 | 0.4360 | -0.0622 | 1392 |
| Right pars triangularis | 1.084108 | 1.313161 | 0.83 | 4.09E-01 | 0.6061 | 0.0445 | 1392 |
| Right pericalcarine | -0.941862 | 1.405024 | -0.67 | 5.03E-01 | 0.6636 | -0.0365 | 1352 |
| Right postcentral cortex | 0.529494 | 2.166865 | 0.24 | 8.07E-01 | 0.8976 | 0.0130 | 1375 |
| Right posterior cingulate cortex | 1.644169 | 0.920352 | 1.79 | 7.40E-02 | 0.2151 | 0.0960 | 1393 |
| Right precentral cortex | -0.180139 | 2.569206 | -0.07 | 9.44E-01 | 0.9687 | -0.0038 | 1381 |
| Right precuneus | 5.854084 | 2.355810 | 2.48 | 1.30E-02 | 0.0704 | 0.1331 | 1390 |
| Right rostral anterior cingulate cortex | 1.820744 | 0.774547 | 2.35 | 1.89E-02 | 0.0926 | 0.1263 | 1386 |
| Right rostral middle frontal cortex | -5.181958 | 3.391426 | -1.53 | 1.30E-01 | 0.3065 | -0.0821 | 1390 |
| Right superior frontal cortex | 1.712460 | 3.359530 | 0.51 | 6.10E-01 | 0.7601 | 0.0274 | 1391 |
| Right superior parietal cortex | 2.186608 | 2.865727 | 0.76 | 4.46E-01 | 0.6308 | 0.0409 | 1386 |
| Right superior temporal cortex | 1.548571 | 1.981638 | 0.78 | 4.35E-01 | 0.6308 | 0.0432 | 1306 |
| Right supramarginal cortex | 4.010445 | 2.652570 | 1.51 | 1.31E-01 | 0.3065 | 0.0827 | 1335 |
| Right frontal pole | -0.082455 | 0.240846 | -0.34 | 7.32E-01 | 0.8450 | -0.0182 | 1392 |
| Right temporal pole | 0.432776 | 0.353731 | 1.22 | 2.21E-01 | 0.4261 | 0.0655 | 1389 |
| Right transverse temporal cortex | 0.605062 | 0.342607 | 1.77 | 7.80E-02 | 0.2187 | 0.0950 | 1392 |
| Right insula | -0.418930 | 1.331967 | -0.31 | 7.53E-01 | 0.8569 | -0.0171 | 1318 |

| **Label** | **Estimate** | **StdError** | **T** | **p** | **FDR adjusted p** | **Cohen´s d** | **N** |
| --- | --- | --- | --- | --- | --- | --- | --- |
|  |  |  |  |  |  |  |  |
| **Global measures** |  |  |  |  |  |  |  |
| Total Intracranial Volume | 1079.000000 | 793.000000 | 1.36 | 1.74E-01 | 0.5547 | 0.0349 | 6112 |
| Total left hemispheral surface area | 90.580000 | 36.230000 | 2.50 | 1.24E-02 | 0.2781 | 0.0632 | 6287 |
| Total right hemispheral surface area | 92.260000 | 36.310000 | 2.54 | 1.11E-02 | 0.2781 | 0.0642 | 6287 |
| Left hemispheral average thickness | -0.000132 | 0.000470 | -0.28 | 7.78E-01 | 0.8592 | -0.0071 | 6306 |
| Right hemispheral average thickness | -0.000210 | 0.000469 | -0.45 | 6.54E-01 | 0.7891 | -0.0114 | 6305 |
|  |  |  |  |  |  |  |  |
| **Cortical thickness** |  |  |  |  |  |  |  |
| Left banks of the superior temporal sulcus | -0.000770 | 0.000890 | -0.87 | 3.87E-01 | 0.6390 | -0.0226 | 5977 |
| Left caudal anterior cingulate cortex | -0.000489 | 0.001235 | -0.40 | 6.92E-01 | 0.8112 | -0.0101 | 6285 |
| Left caudal middle frontal gyrus | -0.000363 | 0.000745 | -0.49 | 6.27E-01 | 0.7686 | -0.0124 | 6263 |
| Left cuneus | 0.000544 | 0.000686 | 0.79 | 4.30E-01 | 0.6819 | 0.0201 | 6195 |
| Left entorhinal cortex | -0.003561 | 0.001859 | -1.92 | 5.54E-02 | 0.5415 | -0.0494 | 6066 |
| Left fusiform gyrus | -0.000121 | 0.000739 | -0.16 | 8.70E-01 | 0.9170 | -0.0040 | 6276 |
| Left inferior parietal cortex | -0.000112 | 0.000666 | -0.17 | 8.67E-01 | 0.9170 | -0.0043 | 6220 |
| Left inferior temporal gyrus | 0.001084 | 0.000840 | 1.29 | 1.97E-01 | 0.5556 | 0.0329 | 6193 |
| Left isthmus cingulate cortex | -0.001317 | 0.001001 | -1.32 | 1.88E-01 | 0.5547 | -0.0334 | 6284 |
| Left lateral occipital cortex | 0.000093 | 0.000610 | 0.15 | 8.80E-01 | 0.9211 | 0.0038 | 6267 |
| Left lateral orbitofrontal cortex | 0.000510 | 0.000826 | 0.62 | 5.37E-01 | 0.7313 | 0.0157 | 6267 |
| Left lingual gyrus | -0.000230 | 0.000623 | -0.37 | 7.11E-01 | 0.8208 | -0.0094 | 6254 |
| Left medial orbitofrontal cortex | -0.001869 | 0.000823 | -2.27 | 2.31E-02 | 0.3300 | -0.0577 | 6214 |
| Left middle temporal gyrus | -0.000598 | 0.000851 | -0.70 | 4.83E-01 | 0.7058 | -0.0180 | 6063 |
| Left parahippcampal gyrus | -0.002113 | 0.001598 | -1.32 | 1.86E-01 | 0.5547 | -0.0334 | 6275 |
| Left paracentral lobule | 0.000800 | 0.000765 | 1.05 | 2.95E-01 | 0.5845 | 0.0266 | 6282 |
| Left pars opercularis | -0.000805 | 0.000744 | -1.08 | 2.79E-01 | 0.5845 | -0.0274 | 6264 |
| Left pars orbitalis | -0.001543 | 0.001127 | -1.37 | 1.71E-01 | 0.5547 | -0.0347 | 6279 |
| Left pars triangularis | -0.000611 | 0.000808 | -0.76 | 4.50E-01 | 0.6924 | -0.0192 | 6268 |
| Left pericalcarine | 0.000712 | 0.000671 | 1.06 | 2.89E-01 | 0.5845 | 0.0270 | 6190 |
| Left precentral gyrus | 0.000369 | 0.000607 | 0.61 | 5.43E-01 | 0.7313 | 0.0155 | 6225 |
| Left posterior cingulate cortex | 0.000556 | 0.000807 | 0.69 | 4.90E-01 | 0.7058 | 0.0175 | 6287 |
| Left precentral gyrus | 0.000498 | 0.000705 | 0.71 | 4.80E-01 | 0.7058 | 0.0180 | 6246 |
| Left precuneus | 0.000137 | 0.000650 | 0.21 | 8.33E-01 | 0.8893 | 0.0053 | 6272 |
| Left rostral anterior cingulate cortex | -0.002862 | 0.001184 | -2.42 | 1.57E-02 | 0.3053 | -0.0614 | 6255 |
| Left rostral middle frontal gyrus | -0.000652 | 0.000692 | -0.94 | 3.46E-01 | 0.6034 | -0.0238 | 6276 |
| Left superior frontal gyrus | -0.000249 | 0.000722 | -0.34 | 7.31E-01 | 0.8279 | -0.0086 | 6272 |
| Left superior parietal cortex | 0.000794 | 0.000595 | 1.34 | 1.82E-01 | 0.5547 | 0.0340 | 6239 |
| Left superior temporal gyrus | -0.001266 | 0.000821 | -1.54 | 1.23E-01 | 0.5547 | -0.0400 | 5949 |
| Left supramarginal gyrus | -0.001052 | 0.000692 | -1.52 | 1.29E-01 | 0.5547 | -0.0389 | 6128 |
| Left frontal pole | -0.001355 | 0.001540 | -0.88 | 3.79E-01 | 0.6330 | -0.0222 | 6300 |
| Left temporal pole | -0.002680 | 0.001890 | -1.42 | 1.56E-01 | 0.5547 | -0.0360 | 6262 |
| Left transverse temporal gyrus | -0.001739 | 0.001112 | -1.56 | 1.18E-01 | 0.5547 | -0.0395 | 6276 |
| Left insula | -0.001261 | 0.000823 | -1.53 | 1.25E-01 | 0.5547 | -0.0389 | 6217 |
| Right banks of the superior temporal sulcus | -0.001188 | 0.000886 | -1.34 | 1.80E-01 | 0.5547 | -0.0342 | 6165 |
| Right caudal anterior cingulate cortex | -0.000646 | 0.001147 | -0.56 | 5.73E-01 | 0.7328 | -0.0142 | 6288 |
| Right caudal middle frontal gyrus | -0.000308 | 0.000742 | -0.42 | 6.78E-01 | 0.8065 | -0.0106 | 6271 |
| Right cuneus | -0.000468 | 0.000692 | -0.68 | 4.99E-01 | 0.7086 | -0.0173 | 6199 |
| Right entorhinal cortex | -0.002201 | 0.002004 | -1.10 | 2.72E-01 | 0.5845 | -0.0285 | 6010 |
| Right fusiform gyrus | -0.000746 | 0.000749 | -1.00 | 3.19E-01 | 0.5845 | -0.0253 | 6281 |
| Right inferior parietal cortex | 0.000163 | 0.000651 | 0.25 | 8.02E-01 | 0.8684 | 0.0064 | 6224 |
| Right inferior temporal gyrus | -0.000663 | 0.000857 | -0.77 | 4.39E-01 | 0.6876 | -0.0196 | 6227 |
| Right isthmus cingulate cortex | -0.001548 | 0.000975 | -1.59 | 1.12E-01 | 0.5547 | -0.0402 | 6284 |
| Right lateral occipital cortex | 0.000840 | 0.000633 | 1.33 | 1.84E-01 | 0.5547 | 0.0337 | 6271 |
| Right lateral orbitofrontal cortex | -0.000836 | 0.000823 | -1.02 | 3.10E-01 | 0.5845 | -0.0258 | 6283 |
| Right lingual gyrus | 0.000306 | 0.000623 | 0.49 | 6.24E-01 | 0.7686 | 0.0124 | 6239 |
| Right medial orbitofrontal cortex | 0.000060 | 0.000851 | 0.07 | 9.44E-01 | 0.9496 | 0.0018 | 6237 |
| Right middle temporal gyrus | -0.001086 | 0.000824 | -1.32 | 1.88E-01 | 0.5547 | -0.0336 | 6212 |
| Right parahippcampal gyrus | -0.001480 | 0.001370 | -1.08 | 2.81E-01 | 0.5845 | -0.0273 | 6280 |
| Right paracentral lobule | 0.000427 | 0.000765 | 0.56 | 5.77E-01 | 0.7328 | 0.0142 | 6286 |
| Right pars opercularis | -0.000554 | 0.000779 | -0.71 | 4.77E-01 | 0.7058 | -0.0180 | 6242 |
| Right pars orbitalis | -0.000700 | 0.001102 | -0.63 | 5.26E-01 | 0.7303 | -0.0159 | 6277 |
| Right pars triangularis | -0.000553 | 0.000793 | -0.70 | 4.85E-01 | 0.7058 | -0.0178 | 6241 |
| Right pericalcarine | -0.000227 | 0.000678 | -0.33 | 7.40E-01 | 0.8299 | -0.0084 | 6172 |
| Right precentral gyrus | 0.000439 | 0.000610 | 0.72 | 4.72E-01 | 0.7058 | 0.0183 | 6245 |
| Right posterior cingulate cortex | 0.001240 | 0.000784 | 1.58 | 1.14E-01 | 0.5547 | 0.0399 | 6290 |
| Right precentral gyrus | 0.000629 | 0.000712 | 0.88 | 3.77E-01 | 0.6330 | 0.0223 | 6254 |
| Right precuneus | 0.000001 | 0.000643 | 0.00 | 9.98E-01 | 0.9982 | <0.0001 | 6274 |
| Right rostral anterior cingulate cortex | -0.003109 | 0.001163 | -2.67 | 7.55E-03 | 0.2781 | -0.0677 | 6258 |
| Right rostral middle frontal gyrus | -0.001338 | 0.000689 | -1.94 | 5.22E-02 | 0.5415 | -0.0491 | 6269 |
| Right superior frontal gyrus | -0.000247 | 0.000723 | -0.34 | 7.33E-01 | 0.8279 | -0.0086 | 6277 |
| Right superior parietal cortex | 0.000790 | 0.000595 | 1.33 | 1.84E-01 | 0.5547 | 0.0337 | 6256 |
| Right superior temporal gyrus | -0.002083 | 0.000806 | -2.58 | 9.81E-03 | 0.2781 | -0.0664 | 6081 |
| Right supramarginal gyrus | -0.000449 | 0.000694 | -0.65 | 5.18E-01 | 0.7260 | -0.0166 | 6145 |
| Right frontal pole | 0.000137 | 0.001520 | 0.09 | 9.28E-01 | 0.9403 | 0.0023 | 6296 |
| Right temporal pole | -0.002323 | 0.001954 | -1.19 | 2.35E-01 | 0.5693 | -0.0301 | 6267 |
| Right transverse temporal gyrus | -0.002179 | 0.001170 | -1.86 | 6.30E-02 | 0.5415 | -0.0471 | 6267 |
| Right insula | -0.002010 | 0.000873 | -2.30 | 2.13E-02 | 0.3300 | -0.0587 | 6165 |
|  |  |  |  |  |  |  |  |
| **Subcortical volumes** |  |  |  |  |  |  |  |
| Left lateral ventricle | -38.800629 | 24.029984 | -1.61 | 1.06E-01 | 0.5547 | -0.0417 | 5991 |
| Right lateral ventricle | -10.007504 | 22.299033 | -0.45 | 6.54E-01 | 0.7891 | -0.0117 | 5976 |
| Left thalamus | 6.592650 | 3.645129 | 1.81 | 7.06E-02 | 0.5415 | 0.0470 | 5971 |
| Right thalamus | 2.647502 | 3.150355 | 0.84 | 4.01E-01 | 0.6486 | 0.0218 | 5957 |
| Left caudate | 1.729833 | 2.140085 | 0.81 | 4.19E-01 | 0.6712 | 0.0210 | 5968 |
| Right caudate | 2.173924 | 2.163796 | 1.00 | 3.15E-01 | 0.5845 | 0.0261 | 5914 |
| Left putamen | -1.124154 | 2.928679 | -0.38 | 7.01E-01 | 0.8154 | -0.0100 | 5773 |
| Right putamen | -3.173115 | 2.714113 | -1.17 | 2.42E-01 | 0.5693 | -0.0307 | 5842 |
| Left pallidum | -1.012099 | 1.105513 | -0.92 | 3.60E-01 | 0.6143 | -0.0244 | 5702 |
| Right pallidum | 0.294766 | 0.986988 | 0.30 | 7.65E-01 | 0.8521 | 0.0078 | 5925 |
| Left hippocampus | 2.940653 | 1.961980 | 1.50 | 1.34E-01 | 0.5547 | 0.0390 | 5943 |
| Right hippocamups | 1.970417 | 1.950896 | 1.01 | 3.13E-01 | 0.5845 | 0.0262 | 5994 |
| Left amygdala | -0.558810 | 0.921810 | -0.61 | 5.44E-01 | 0.7313 | -0.0159 | 5956 |
| Right amygdala | -1.032826 | 0.981386 | -1.05 | 2.93E-01 | 0.5845 | -0.0273 | 5967 |
| Left nucleus accumbens | 0.559914 | 0.493753 | 1.13 | 2.57E-01 | 0.5844 | 0.0296 | 5856 |
| Right nucleus accumbens | 0.161914 | 0.451515 | 0.36 | 7.20E-01 | 0.8250 | 0.0094 | 5879 |
|  |  |  |  |  |  |  |  |
| **Cortical surface area** |  |  |  |  |  |  |  |
| Left banks of the superior temporal sulcus | 0.938038 | 0.814277 | 1.15 | 2.49E-01 | 0.5758 | 0.0309 | 5566 |
| Left caudal anterior cingulate cortex | 1.223647 | 0.714968 | 1.71 | 8.71E-02 | 0.5467 | 0.0446 | 5904 |
| Left caudal middle frontal gyrus | 2.819753 | 1.796882 | 1.57 | 1.17E-01 | 0.5547 | 0.0410 | 5904 |
| Left cuneus | -1.869113 | 1.071733 | -1.74 | 8.12E-02 | 0.5467 | -0.0456 | 5859 |
| Left entorhinal cortex | -0.318095 | 0.414033 | -0.77 | 4.42E-01 | 0.6876 | -0.0207 | 5555 |
| Left fusiform gyrus | 1.258059 | 1.815646 | 0.69 | 4.88E-01 | 0.7058 | 0.0182 | 5799 |
| Left inferior parietal cortex | 3.378796 | 2.893427 | 1.17 | 2.43E-01 | 0.5693 | 0.0307 | 5853 |
| Left inferior temporal gyrus | 4.083921 | 2.155419 | 1.89 | 5.82E-02 | 0.5415 | 0.0495 | 5876 |
| Left isthmus cingulate cortex | 0.183728 | 0.782643 | 0.23 | 8.14E-01 | 0.8758 | 0.0060 | 5962 |
| Left lateral occipital cortex | -2.981567 | 2.741828 | -1.09 | 2.77E-01 | 0.5845 | -0.0283 | 5946 |
| Left lateral orbitofrontal cortex | 0.681430 | 1.273379 | 0.54 | 5.93E-01 | 0.7443 | 0.0140 | 5979 |
| Left lingual gyrus | 1.315249 | 1.955360 | 0.67 | 5.01E-01 | 0.7086 | 0.0174 | 5944 |
| Left medial orbitofrontal cortex | 1.956346 | 1.101919 | 1.78 | 7.59E-02 | 0.5415 | 0.0467 | 5841 |
| Left middle temporal gyrus | 3.860671 | 1.836429 | 2.10 | 3.56E-02 | 0.4430 | 0.0559 | 5678 |
| Left parahippcampal gyrus | 0.826895 | 0.459285 | 1.80 | 7.19E-02 | 0.5415 | 0.0470 | 5897 |
| Left paracentral lobule | 1.099382 | 0.934897 | 1.18 | 2.40E-01 | 0.5693 | 0.0310 | 5838 |
| Left pars opercularis | 1.801772 | 1.271859 | 1.42 | 1.57E-01 | 0.5547 | 0.0370 | 5921 |
| Left pars orbitalis | 0.548604 | 0.397237 | 1.38 | 1.70E-01 | 0.5547 | 0.0358 | 5968 |
| Left pars triangularis | 1.278309 | 0.977075 | 1.31 | 1.91E-01 | 0.5547 | 0.0341 | 5943 |
| Left pericalcarine | -1.156588 | 1.185672 | -0.98 | 3.29E-01 | 0.5845 | -0.0256 | 5888 |
| Left precentral gyrus | 3.020906 | 2.032958 | 1.49 | 1.37E-01 | 0.5547 | 0.0391 | 5838 |
| Left posterior cingulate cortex | 1.181769 | 0.853530 | 1.38 | 1.66E-01 | 0.5547 | 0.0358 | 5965 |
| Left precentral gyrus | 2.138895 | 2.318934 | 0.92 | 3.56E-01 | 0.6143 | 0.0241 | 5881 |
| Left precuneus | 2.558203 | 2.012976 | 1.27 | 2.00E-01 | 0.5556 | 0.0330 | 5949 |
| Left rostral anterior cingulate cortex | 2.052501 | 0.731557 | 2.81 | 5.04E-03 | 0.2781 | 0.0739 | 5816 |
| Left rostral middle frontal gyrus | 3.989123 | 3.180806 | 1.25 | 2.10E-01 | 0.5556 | 0.0325 | 5952 |
| Left superior frontal gyrus | 5.158549 | 3.401558 | 1.52 | 1.29E-01 | 0.5547 | 0.0398 | 5859 |
| Left superior parietal cortex | 1.682170 | 2.945412 | 0.57 | 5.68E-01 | 0.7328 | 0.0149 | 5875 |
| Left superior temporal gyrus | 5.713334 | 1.952958 | 2.93 | 3.45E-03 | 0.2781 | 0.0787 | 5577 |
| Left supramarginal gyrus | 2.472724 | 2.545247 | 0.97 | 3.31E-01 | 0.5845 | 0.0258 | 5701 |
| Left frontal pole | 0.045592 | 0.176479 | 0.26 | 7.96E-01 | 0.8680 | 0.0067 | 5990 |
| Left temporal pole | -0.160657 | 0.304810 | -0.53 | 5.98E-01 | 0.7453 | -0.0138 | 5954 |
| Left transverse temporal gyrus | 0.373502 | 0.367948 | 1.02 | 3.10E-01 | 0.5845 | 0.0264 | 5986 |
| Left insula | 1.297881 | 1.107646 | 1.17 | 2.41E-01 | 0.5693 | 0.0306 | 5899 |
| Right banks of the superior temporal sulcus | 0.846278 | 0.666801 | 1.27 | 2.04E-01 | 0.5556 | 0.0334 | 5804 |
| Right caudal anterior cingulate cortex | 0.312889 | 0.774788 | 0.40 | 6.86E-01 | 0.8102 | 0.0104 | 5930 |
| Right caudal middle frontal gyrus | 1.910486 | 1.785621 | 1.07 | 2.85E-01 | 0.5845 | 0.0279 | 5922 |
| Right cuneus | 0.611717 | 1.009865 | 0.61 | 5.45E-01 | 0.7313 | 0.0160 | 5874 |
| Right entorhinal cortex | -0.046400 | 0.420115 | -0.11 | 9.12E-01 | 0.9389 | -0.0030 | 5468 |
| Right fusiform gyrus | 2.085677 | 1.784144 | 1.17 | 2.42E-01 | 0.5693 | 0.0308 | 5817 |
| Right inferior parietal cortex | 6.573521 | 3.145612 | 2.09 | 3.67E-02 | 0.4430 | 0.0548 | 5850 |
| Right inferior temporal gyrus | 0.200277 | 2.018946 | 0.10 | 9.21E-01 | 0.9389 | 0.0026 | 5911 |
| Right isthmus cingulate cortex | 0.317210 | 0.717531 | 0.44 | 6.58E-01 | 0.7891 | 0.0114 | 5976 |
| Right lateral occipital cortex | 0.292168 | 2.746313 | 0.11 | 9.15E-01 | 0.9389 | 0.0029 | 5962 |
| Right lateral orbitofrontal cortex | 1.834208 | 1.368591 | 1.34 | 1.80E-01 | 0.5547 | 0.0347 | 5984 |
| Right lingual gyrus | 0.530970 | 1.923896 | 0.28 | 7.83E-01 | 0.8592 | 0.0073 | 5942 |
| Right medial orbitofrontal cortex | 0.552190 | 0.950113 | 0.58 | 5.61E-01 | 0.7328 | 0.0151 | 5909 |
| Right middle temporal gyrus | 2.220537 | 1.791811 | 1.24 | 2.15E-01 | 0.5556 | 0.0325 | 5839 |
| Right parahippcampal gyrus | 0.817100 | 0.457199 | 1.79 | 7.40E-02 | 0.5415 | 0.0467 | 5909 |
| Right paracentral lobule | 1.316645 | 1.039379 | 1.27 | 2.05E-01 | 0.5556 | 0.0331 | 5904 |
| Right pars opercularis | 2.147428 | 1.143451 | 1.88 | 6.04E-02 | 0.5415 | 0.0491 | 5897 |
| Right pars orbitalis | 0.657240 | 0.464531 | 1.41 | 1.57E-01 | 0.5547 | 0.0366 | 5964 |
| Right pars triangularis | 1.419495 | 1.146834 | 1.24 | 2.16E-01 | 0.5556 | 0.0324 | 5909 |
| Right pericalcarine | -1.361261 | 1.230961 | -1.11 | 2.69E-01 | 0.5845 | -0.0290 | 5882 |
| Right precentral gyrus | 2.035812 | 2.020324 | 1.01 | 3.14E-01 | 0.5845 | 0.0264 | 5883 |
| Right posterior cingulate cortex | 0.822346 | 0.834564 | 0.99 | 3.24E-01 | 0.5845 | 0.0257 | 5972 |
| Right precentral gyrus | 3.782296 | 2.422642 | 1.56 | 1.19E-01 | 0.5547 | 0.0408 | 5894 |
| Right precuneus | 5.012337 | 2.109045 | 2.38 | 1.75E-02 | 0.3053 | 0.0618 | 5959 |
| Right rostral anterior cingulate cortex | 0.396023 | 0.667041 | 0.59 | 5.53E-01 | 0.7328 | 0.0154 | 5894 |
| Right rostral middle frontal gyrus | 5.575036 | 3.256380 | 1.71 | 8.69E-02 | 0.5467 | 0.0445 | 5947 |
| Right superior frontal gyrus | 2.954331 | 3.443979 | 0.86 | 3.91E-01 | 0.6395 | 0.0225 | 5897 |
| Right superior parietal cortex | 1.632039 | 2.803299 | 0.58 | 5.60E-01 | 0.7328 | 0.0151 | 5903 |
| Right superior temporal gyrus | 4.475112 | 1.727221 | 2.59 | 9.60E-03 | 0.2781 | 0.0688 | 5707 |
| Right supramarginal gyrus | -1.315656 | 2.369530 | -0.56 | 5.79E-01 | 0.7328 | -0.0148 | 5760 |
| Right frontal pole | 0.215303 | 0.221437 | 0.97 | 3.31E-01 | 0.5845 | 0.0251 | 5989 |
| Right temporal pole | -0.042273 | 0.334559 | -0.13 | 8.99E-01 | 0.9352 | -0.0034 | 5891 |
| Right transverse temporal gyrus | 0.458564 | 0.281831 | 1.63 | 1.04E-01 | 0.5547 | 0.0422 | 5991 |
| Right insula | 1.286499 | 1.249771 | 1.03 | 3.03E-01 | 0.5845 | 0.0270 | 5849 |

| **Label** | **Estimate** | **StdError** | **T** | **p** | **FDR adjusted p** | **Cohens D** | **N Obese** | **N NW** |
| --- | --- | --- | --- | --- | --- | --- | --- | --- |
|  |  |  |  |  |  |  |  |  |
| **Global measures** |  |  |  |  |  |  |  |  |
| Total Intracranial Volume | -22892.00000 | 7967.000000 | -2.87 | 4.10E-03 | 0.0257 | -0.1416 | 558 | 1566 |
| Total left hemispheral surface area | -918.500000 | 369.300000 | -2.49 | 1.30E-02 | 0.0690 | -0.1224 | 555 | 1636 |
| Total right hemispheral surface area | -914.700000 | 371.100000 | -2.46 | 1.38E-02 | 0.0690 | -0.1209 | 555 | 1636 |
| Left hemispheral average thickness | -0.015030 | 0.004980 | -3.02 | 2.60E-03 | 0.0212 | -0.1473 | 567 | 1629 |
| Right hemispheral average thickness | -0.014925 | 0.004924 | -3.03 | 2.50E-03 | 0.0212 | -0.1478 | 567 | 1629 |
|  |  |  |  |  |  |  |  |  |
| **Cortical thickness** |  |  |  |  |  |  |  |  |
| Left banks of the superior temporal sulcus | -0.019013 | 0.009217 | -2.06 | 3.93E-02 | 0.1469 | -0.1019 | 556 | 1548 |
| Left caudal anterior cingulate cortex | -0.019198 | 0.012986 | -1.48 | 1.39E-01 | 0.3475 | -0.0723 | 567 | 1618 |
| Left caudal middle frontal cortex | -0.018862 | 0.007972 | -2.37 | 1.81E-02 | 0.0788 | -0.1159 | 565 | 1611 |
| Left cuneus | -0.003823 | 0.007043 | -0.54 | 5.90E-01 | 0.7617 | -0.0266 | 559 | 1590 |
| Left entorhinal cortex | -0.035941 | 0.019390 | -1.85 | 6.39E-02 | 0.1985 | -0.0914 | 557 | 1551 |
| Left fusiform cortex | -0.043920 | 0.007582 | -5.79 | 7.94E-09 | <0.0001 | -0.2830 | 565 | 1619 |
| Left inferior parietal cortex | -0.007657 | 0.006983 | -1.10 | 2.73E-01 | 0.5179 | -0.0540 | 560 | 1612 |
| Left inferior temporal cortex | -0.038223 | 0.008699 | -4.39 | 1.17E-05 | 0.0004 | -0.2161 | 556 | 1608 |
| Left isthmus cingulate cortex | -0.019437 | 0.010400 | -1.87 | 6.18E-02 | 0.1979 | -0.0913 | 566 | 1623 |
| Left lateral occipital cortex | -0.001874 | 0.006497 | -0.29 | 7.70E-01 | 0.8697 | -0.0142 | 560 | 1622 |
| Left lateral orbitofrontal cortex | -0.021464 | 0.008631 | -2.49 | 1.30E-02 | 0.0690 | -0.1221 | 560 | 1618 |
| Left lingual cortex | -0.004354 | 0.006555 | -0.66 | 5.07E-01 | 0.7107 | -0.0323 | 562 | 1612 |
| Left medial orbitofrontal cortex | -0.011998 | 0.008611 | -1.39 | 1.64E-01 | 0.3835 | -0.0682 | 561 | 1601 |
| Left middle temporal cortex | -0.029444 | 0.008735 | -3.37 | 7.60E-04 | 0.0119 | -0.1666 | 554 | 1569 |
| Left parahippocampal cortex | -0.016565 | 0.016646 | -1.00 | 3.20E-01 | 0.5457 | -0.0488 | 566 | 1624 |
| Left paracentral cortex | -0.022696 | 0.008101 | -2.80 | 5.10E-03 | 0.0308 | -0.1370 | 563 | 1624 |
| Left pars opercularis | -0.015412 | 0.007868 | -1.96 | 5.00E-02 | 0.1744 | -0.0959 | 564 | 1620 |
| Left pars orbitalis | -0.003037 | 0.011793 | -0.26 | 7.97E-01 | 0.8810 | -0.0127 | 561 | 1625 |
| Left pars triangularis | -0.003222 | 0.008443 | -0.38 | 7.03E-01 | 0.8374 | -0.0186 | 561 | 1621 |
| Left pericalcarine | 0.002505 | 0.006887 | 0.36 | 7.16E-01 | 0.8374 | 0.0177 | 558 | 1595 |
| Left postcentral cortex | -0.002320 | 0.006170 | -0.38 | 7.07E-01 | 0.8374 | -0.0187 | 557 | 1606 |
| Left posterior cingulate cortex | -0.026904 | 0.008274 | -3.25 | 1.17E-03 | 0.0153 | -0.1588 | 565 | 1625 |
| Left precentral cortex | -0.025447 | 0.007496 | -3.39 | 7.00E-04 | 0.0119 | -0.1659 | 564 | 1611 |
| Left precuneus | -0.014273 | 0.006771 | -2.11 | 3.50E-02 | 0.1409 | -0.1035 | 560 | 1622 |
| Left rostral anterior cingulate cortex | -0.011352 | 0.012528 | -0.91 | 3.65E-01 | 0.6075 | -0.0445 | 565 | 1612 |
| Left rostral middle frontal cortex | -0.005900 | 0.007320 | -0.81 | 4.20E-01 | 0.6345 | -0.0395 | 567 | 1618 |
| Left superior frontal cortex | -0.015390 | 0.007600 | -2.02 | 4.31E-02 | 0.1574 | -0.0988 | 564 | 1621 |
| Left superior parietal cortex | -0.008710 | 0.006298 | -1.38 | 1.67E-01 | 0.3851 | -0.0677 | 560 | 1610 |
| Left superior temporal cortex | -0.025637 | 0.008337 | -3.08 | 2.10E-03 | 0.0196 | -0.1534 | 548 | 1534 |
| Left supramarginal cortex | -0.003867 | 0.007212 | -0.54 | 5.92E-01 | 0.7617 | -0.0267 | 555 | 1580 |
| Left frontal pole | -0.012768 | 0.016283 | -0.78 | 4.33E-01 | 0.6421 | -0.0381 | 566 | 1628 |
| Left temporal pole | -0.041205 | 0.019792 | -2.08 | 3.75E-02 | 0.1435 | -0.1023 | 556 | 1621 |
| Left transverse temporal cortex | -0.024645 | 0.011498 | -2.14 | 3.22E-02 | 0.1330 | -0.1048 | 562 | 1621 |
| Left insula | -0.021326 | 0.008652 | -2.46 | 1.38E-02 | 0.0690 | -0.1207 | 563 | 1592 |
| Right banks of the superior temporal sulcus | -0.016247 | 0.009219 | -1.76 | 7.82E-02 | 0.2183 | -0.0864 | 560 | 1601 |
| Right caudal anterior cingulate cortex | -0.007871 | 0.012073 | -0.65 | 5.14E-01 | 0.7148 | -0.0317 | 566 | 1621 |
| Right caudal middle frontal cortex | -0.013949 | 0.007824 | -1.78 | 7.47E-02 | 0.2132 | -0.0871 | 564 | 1617 |
| Right cuneus | -0.000124 | 0.007189 | -0.02 | 9.86E-01 | 0.9925 | -0.0010 | 557 | 1602 |
| Right entorhinal cortex | -0.014501 | 0.020460 | -0.71 | 4.79E-01 | 0.6888 | -0.0353 | 551 | 1525 |
| Right fusiform cortex | -0.044240 | 0.007539 | -5.87 | 5.09E-09 | <0.0001 | -0.2871 | 564 | 1622 |
| Right inferior parietal cortex | -0.008392 | 0.006826 | -1.23 | 2.19E-01 | 0.4710 | -0.0603 | 563 | 1609 |
| Right inferior temporal cortex | -0.030276 | 0.008963 | -3.38 | 7.40E-04 | 0.0119 | -0.1661 | 558 | 1610 |
| Right isthmus cingulate cortex | -0.016710 | 0.010040 | -1.66 | 9.60E-02 | 0.2512 | -0.0811 | 565 | 1622 |
| Right lateral occipital cortex | -0.007074 | 0.006621 | -1.07 | 2.90E-01 | 0.5294 | -0.0523 | 564 | 1618 |
| Right lateral orbitofrontal cortex | -0.015717 | 0.008537 | -1.84 | 6.58E-02 | 0.1985 | -0.0900 | 563 | 1624 |
| Right lingual cortex | -0.006535 | 0.006533 | -1.00 | 3.17E-01 | 0.5457 | -0.0491 | 558 | 1616 |
| Right medial orbitofrontal cortex | -0.034117 | 0.008965 | -3.81 | 1.50E-04 | 0.0039 | -0.1869 | 561 | 1610 |
| Right middle temporal cortex | -0.029775 | 0.008722 | -3.41 | 6.50E-04 | 0.0119 | -0.1674 | 561 | 1603 |
| Right parahippocampal cortex | -0.024775 | 0.014160 | -1.75 | 8.03E-02 | 0.2183 | -0.0856 | 563 | 1622 |
| Right paracentral cortex | -0.016096 | 0.008109 | -1.98 | 4.70E-02 | 0.1677 | -0.0967 | 566 | 1623 |
| Right pars opercularis | -0.024869 | 0.008081 | -3.08 | 2.12E-03 | 0.0196 | -0.1506 | 565 | 1615 |
| Right pars orbitalis | -0.004943 | 0.011633 | -0.42 | 6.71E-01 | 0.8230 | -0.0205 | 566 | 1624 |
| Right pars triangularis | -0.012042 | 0.008458 | -1.42 | 1.55E-01 | 0.3680 | -0.0695 | 563 | 1615 |
| Right pericalcarine | 0.000586 | 0.007131 | 0.08 | 9.30E-01 | 0.9606 | 0.0039 | 554 | 1598 |
| Right postcentral cortex | -0.004475 | 0.006372 | -0.70 | 4.83E-01 | 0.6888 | -0.0343 | 562 | 1610 |
| Right posterior cingulate cortex | -0.035165 | 0.007988 | -4.40 | 1.12E-05 | 0.0004 | -0.2149 | 566 | 1624 |
| Right precentral cortex | -0.025322 | 0.007693 | -3.29 | 1.00E-03 | 0.0143 | -0.1614 | 560 | 1614 |
| Right precuneus | -0.016142 | 0.006748 | -2.39 | 1.68E-02 | 0.0776 | -0.1170 | 563 | 1619 |
| Right rostral anterior cingulate cortex | 0.001868 | 0.012304 | 0.15 | 8.79E-01 | 0.9392 | 0.0073 | 565 | 1606 |
| Right rostral middle frontal cortex | -0.003592 | 0.007279 | -0.49 | 6.22E-01 | 0.7748 | -0.0240 | 563 | 1618 |
| Right superior frontal cortex | -0.022622 | 0.007539 | -3.00 | 2.70E-03 | 0.0212 | -0.1472 | 559 | 1624 |
| Right superior parietal cortex | -0.005474 | 0.006212 | -0.88 | 3.78E-01 | 0.6148 | -0.0430 | 565 | 1614 |
| Right superior temporal cortex | -0.024318 | 0.008270 | -2.94 | 3.30E-03 | 0.0236 | -0.1458 | 549 | 1570 |
| Right supramarginal cortex | -0.008713 | 0.007163 | -1.22 | 2.24E-01 | 0.4751 | -0.0601 | 558 | 1582 |
| Right frontal pole | -0.007646 | 0.015914 | -0.48 | 6.31E-01 | 0.7800 | -0.0234 | 567 | 1625 |
| Right temporal pole | -0.029693 | 0.020355 | -1.46 | 1.45E-01 | 0.3497 | -0.0716 | 560 | 1619 |
| Right transverse temporal cortex | -0.009753 | 0.012021 | -0.81 | 4.17E-01 | 0.6345 | -0.0398 | 559 | 1616 |
| Right insula | -0.011241 | 0.009091 | -1.24 | 2.16E-01 | 0.4710 | -0.0610 | 562 | 1564 |
|  |  |  |  |  |  |  |  |  |
| **Subcortical volumes** |  |  |  |  |  |  |  |  |
| Left lateral ventricle | 230.494832 | 223.579734 | 1.03 | 3.03E-01 | 0.5361 | 0.0516 | 539 | 1537 |
| Right lateral ventricle | 83.692021 | 215.701165 | 0.39 | 6.98E-01 | 0.8374 | 0.0196 | 534 | 1537 |
| Left thalamus | 82.233641 | 37.033117 | 2.22 | 2.65E-02 | 0.1124 | 0.1110 | 542 | 1528 |
| Right thalamus | 76.943415 | 32.107054 | 2.40 | 1.66E-02 | 0.0776 | 0.1205 | 538 | 1517 |
| Left caudate | 10.921128 | 21.592005 | 0.51 | 6.13E-01 | 0.7748 | 0.0254 | 549 | 1523 |
| Right caudate | 7.718116 | 21.970164 | 0.35 | 7.25E-01 | 0.8374 | 0.0176 | 533 | 1513 |
| Left putamen | 92.378151 | 29.375901 | 3.14 | 1.69E-03 | 0.0190 | 0.1616 | 507 | 1482 |
| Right putamen | 85.946348 | 27.123258 | 3.17 | 1.55E-03 | 0.0187 | 0.1608 | 525 | 1505 |
| Left pallidum | -10.415853 | 10.951092 | -0.95 | 3.42E-01 | 0.5768 | -0.0485 | 525 | 1441 |
| Right pallidum | -6.002348 | 9.736937 | -0.62 | 5.38E-01 | 0.7254 | -0.0310 | 542 | 1524 |
| Left hippocampus | 16.893198 | 19.439855 | 0.87 | 3.85E-01 | 0.6148 | 0.0437 | 537 | 1521 |
| Right hippocamups | 37.368798 | 19.673539 | 1.90 | 5.77E-02 | 0.1908 | 0.0950 | 542 | 1536 |
| Left amygdala | 25.353344 | 9.190837 | 2.76 | 5.86E-03 | 0.0341 | 0.1387 | 535 | 1529 |
| Right amygdala | 43.628727 | 9.896909 | 4.41 | 1.10E-05 | 0.0004 | 0.2216 | 535 | 1529 |
| Left nucleus accumbens | 14.869898 | 4.991413 | 2.98 | 2.90E-03 | 0.0217 | 0.1509 | 527 | 1504 |
| Right nucleus accumbens | 2.857379 | 4.638293 | 0.62 | 5.38E-01 | 0.7254 | 0.0315 | 525 | 1499 |
|  |  |  |  |  |  |  |  |  |
| **Cortical surface area** |  |  |  |  |  |  |  |  |
| Left banks of the superior temporal sulcus | -2.201914 | 8.167380 | -0.27 | 7.88E-01 | 0.8769 | -0.0137 | 524 | 1533 |
| Left caudal anterior cingulate cortex | -7.851823 | 7.377222 | -1.06 | 2.87E-01 | 0.5294 | -0.0528 | 539 | 1614 |
| Left caudal middle frontal cortex | -17.266227 | 19.160357 | -0.90 | 3.68E-01 | 0.6075 | -0.0447 | 542 | 1607 |
| Left cuneus | 7.929169 | 10.762602 | 0.74 | 4.61E-01 | 0.6707 | 0.0369 | 540 | 1591 |
| Left entorhinal cortex | 0.022972 | 4.126657 | 0.01 | 9.96E-01 | 0.9956 | 0.0005 | 496 | 1506 |
| Left fusiform cortex | -19.064578 | 18.760109 | -1.02 | 3.10E-01 | 0.5401 | -0.0516 | 520 | 1589 |
| Left inferior parietal cortex | -25.571702 | 29.596369 | -0.86 | 3.88E-01 | 0.6148 | -0.0427 | 543 | 1608 |
| Left inferior temporal cortex | -61.924606 | 21.566105 | -2.87 | 4.10E-03 | 0.0257 | -0.1429 | 538 | 1614 |
| Left isthmus cingulate cortex | 24.449382 | 7.881853 | 3.10 | 1.90E-03 | 0.0196 | 0.1530 | 550 | 1627 |
| Left lateral occipital cortex | 0.564421 | 28.169329 | 0.02 | 9.84E-01 | 0.9925 | 0.0010 | 549 | 1629 |
| Left lateral orbitofrontal cortex | 11.269930 | 12.931132 | 0.87 | 3.84E-01 | 0.6148 | 0.0428 | 554 | 1631 |
| Left lingual cortex | -36.707908 | 19.879585 | -1.85 | 6.50E-02 | 0.1985 | -0.0915 | 547 | 1622 |
| Left medial orbitofrontal cortex | -12.099556 | 11.562630 | -1.05 | 2.95E-01 | 0.5332 | -0.0526 | 533 | 1592 |
| Left middle temporal cortex | -32.000534 | 18.307686 | -1.75 | 8.06E-02 | 0.2183 | -0.0885 | 522 | 1563 |
| Left parahippocampal cortex | -0.850763 | 4.529120 | -0.19 | 8.51E-01 | 0.9214 | -0.0095 | 538 | 1618 |
| Left paracentral cortex | 11.257150 | 9.742860 | 1.16 | 2.48E-01 | 0.4910 | 0.0585 | 522 | 1595 |
| Left pars opercularis | -15.067812 | 13.099120 | -1.15 | 2.50E-01 | 0.4910 | -0.0571 | 541 | 1620 |
| Left pars orbitalis | -5.853929 | 3.993853 | -1.47 | 1.43E-01 | 0.3497 | -0.0724 | 552 | 1628 |
| Left pars triangularis | -5.776748 | 9.988929 | -0.58 | 5.63E-01 | 0.7429 | -0.0286 | 549 | 1623 |
| Left pericalcarine | 2.761370 | 12.081794 | 0.23 | 8.19E-01 | 0.8932 | 0.0114 | 547 | 1603 |
| Left postcentral cortex | 5.216692 | 21.230362 | 0.25 | 8.06E-01 | 0.8848 | 0.0126 | 521 | 1601 |
| Left posterior cingulate cortex | 5.347091 | 8.974157 | 0.60 | 5.51E-01 | 0.7336 | 0.0296 | 550 | 1630 |
| Left precentral cortex | 18.175815 | 23.201891 | 0.78 | 4.34E-01 | 0.6421 | 0.0388 | 540 | 1608 |
| Left precuneus | 11.626967 | 20.667241 | 0.56 | 5.74E-01 | 0.7507 | 0.0277 | 548 | 1626 |
| Left rostral anterior cingulate cortex | 0.470341 | 7.480604 | 0.06 | 9.50E-01 | 0.9684 | 0.0030 | 524 | 1593 |
| Left rostral middle frontal cortex | -63.129807 | 32.693465 | -1.93 | 5.36E-02 | 0.1830 | -0.0952 | 551 | 1621 |
| Left superior frontal cortex | 26.734800 | 34.979012 | 0.76 | 4.45E-01 | 0.6526 | 0.0381 | 530 | 1604 |
| Left superior parietal cortex | -11.345280 | 30.588958 | -0.37 | 7.11E-01 | 0.8374 | -0.0185 | 535 | 1607 |
| Left superior temporal cortex | -10.531486 | 19.905259 | -0.53 | 5.97E-01 | 0.7618 | -0.0268 | 527 | 1525 |
| Left supramarginal cortex | 21.400826 | 26.148945 | 0.82 | 4.13E-01 | 0.6345 | 0.0416 | 518 | 1552 |
| Left frontal pole | 1.985838 | 1.814162 | 1.09 | 2.74E-01 | 0.5179 | 0.0536 | 555 | 1635 |
| Left temporal pole | -0.341546 | 3.105960 | -0.11 | 9.12E-01 | 0.9550 | -0.0054 | 550 | 1626 |
| Left transverse temporal cortex | 8.942829 | 3.763793 | 2.38 | 1.76E-02 | 0.0788 | 0.1170 | 555 | 1632 |
| Left insula | 20.232301 | 11.080006 | 1.83 | 6.80E-02 | 0.2014 | 0.0906 | 549 | 1593 |
| Right banks of the superior temporal sulcus | -4.454447 | 6.932918 | -0.64 | 5.21E-01 | 0.7170 | -0.0319 | 539 | 1599 |
| Right caudal anterior cingulate cortex | 9.147814 | 7.918289 | 1.16 | 2.48E-01 | 0.4910 | 0.0577 | 540 | 1618 |
| Right caudal middle frontal cortex | -12.977852 | 18.798281 | -0.69 | 4.90E-01 | 0.6931 | -0.0343 | 541 | 1615 |
| Right cuneus | -11.439189 | 10.257735 | -1.12 | 2.65E-01 | 0.5134 | -0.0558 | 538 | 1606 |
| Right entorhinal cortex | 1.152961 | 4.183450 | 0.28 | 7.83E-01 | 0.8769 | 0.0146 | 491 | 1479 |
| Right fusiform cortex | -6.622796 | 18.371013 | -0.36 | 7.19E-01 | 0.8374 | -0.0181 | 524 | 1598 |
| Right inferior parietal cortex | -56.539894 | 31.585654 | -1.79 | 7.36E-02 | 0.2132 | -0.0891 | 541 | 1599 |
| Right inferior temporal cortex | -35.910444 | 20.660584 | -1.74 | 8.23E-02 | 0.2191 | -0.0863 | 545 | 1613 |
| Right isthmus cingulate cortex | 17.991497 | 7.320780 | 2.46 | 1.41E-02 | 0.0690 | 0.1214 | 550 | 1628 |
| Right lateral occipital cortex | 13.890642 | 27.789024 | 0.50 | 6.17E-01 | 0.7748 | 0.0247 | 550 | 1624 |
| Right lateral orbitofrontal cortex | 1.267581 | 14.132025 | 0.09 | 9.29E-01 | 0.9606 | 0.0044 | 554 | 1633 |
| Right lingual cortex | -21.790987 | 20.046950 | -1.09 | 2.77E-01 | 0.5180 | -0.0539 | 548 | 1624 |
| Right medial orbitofrontal cortex | 3.478958 | 9.843763 | 0.35 | 7.24E-01 | 0.8374 | 0.0175 | 536 | 1609 |
| Right middle temporal cortex | -15.153092 | 18.433724 | -0.82 | 4.11E-01 | 0.6345 | -0.0408 | 541 | 1595 |
| Right parahippocampal cortex | 0.647123 | 4.753242 | 0.14 | 8.92E-01 | 0.9459 | 0.0070 | 538 | 1620 |
| Right paracentral cortex | 20.870336 | 11.017070 | 1.89 | 5.83E-02 | 0.1908 | 0.0941 | 539 | 1614 |
| Right pars opercularis | -0.905509 | 11.729623 | -0.08 | 9.38E-01 | 0.9630 | -0.0040 | 541 | 1612 |
| Right pars orbitalis | -5.622879 | 4.787042 | -1.17 | 2.40E-01 | 0.4910 | -0.0576 | 554 | 1630 |
| Right pars triangularis | -13.938482 | 11.877705 | -1.17 | 2.41E-01 | 0.4910 | -0.0579 | 547 | 1616 |
| Right pericalcarine | 1.379911 | 12.305053 | 0.11 | 9.11E-01 | 0.9550 | 0.0054 | 548 | 1604 |
| Right postcentral cortex | 17.310186 | 20.411957 | 0.85 | 3.97E-01 | 0.6225 | 0.0423 | 541 | 1606 |
| Right posterior cingulate cortex | 8.845739 | 8.601423 | 1.03 | 3.04E-01 | 0.5361 | 0.0508 | 550 | 1630 |
| Right precentral cortex | -33.742716 | 24.903474 | -1.35 | 1.76E-01 | 0.3995 | -0.0676 | 531 | 1603 |
| Right precuneus | 3.872646 | 21.521945 | 0.18 | 8.57E-01 | 0.9218 | 0.0089 | 550 | 1624 |
| Right rostral anterior cingulate cortex | 10.491856 | 6.915848 | 1.52 | 1.29E-01 | 0.3282 | 0.0761 | 533 | 1596 |
| Right rostral middle frontal cortex | -96.932173 | 33.344340 | -2.91 | 3.69E-03 | 0.0252 | -0.1436 | 550 | 1627 |
| Right superior frontal cortex | 43.075364 | 34.800852 | 1.24 | 2.16E-01 | 0.4710 | 0.0619 | 536 | 1611 |
| Right superior parietal cortex | -10.202926 | 29.794273 | -0.34 | 7.32E-01 | 0.8389 | -0.0168 | 546 | 1613 |
| Right superior temporal cortex | 5.620932 | 17.656415 | 0.32 | 7.50E-01 | 0.8535 | 0.0161 | 530 | 1559 |
| Right supramarginal cortex | 50.064859 | 24.015263 | 2.08 | 3.72E-02 | 0.1435 | 0.1043 | 534 | 1568 |
| Right frontal pole | -3.017014 | 2.277325 | -1.32 | 1.85E-01 | 0.4158 | -0.0649 | 555 | 1630 |
| Right temporal pole | 3.915355 | 3.396853 | 1.15 | 2.49E-01 | 0.4910 | 0.0577 | 528 | 1615 |
| Right transverse temporal cortex | 1.807698 | 2.953598 | 0.61 | 5.41E-01 | 0.7254 | 0.0300 | 555 | 1635 |
| Right insula | 19.281044 | 12.716550 | 1.52 | 1.30E-01 | 0.3282 | 0.0755 | 547 | 1569 |

| **Label** | **Estimate** | **StdError** | **T** | **p** | **FDR adjusted p** | **Cohens D** | **N Obese** | **N NW** |
| --- | --- | --- | --- | --- | --- | --- | --- | --- |
|  |  |  |  |  |  |  |  |  |
| **Global measures** |  |  |  |  |  |  |  |  |
| Total Intracranial Volume | -18616.0000 | 7879.000000 | -2.36 | 1.82E-02 | 0.0502 | -0.1176 | 610 | 1189 |
| Total left hemispheral surface area | -326.700000 | 359.900000 | -0.91 | 3.64E-01 | 0.4987 | -0.0445 | 634 | 1236 |
| Total right hemispheral surface area | -427.600000 | 361.500000 | -1.18 | 2.37E-01 | 0.3758 | -0.0577 | 634 | 1236 |
| Left hemispheral average thickness | -0.026071 | 0.004635 | -5.63 | 2.14E-08 | <0.0001 | -0.2753 | 633 | 1236 |
| Right hemispheral average thickness | -0.024134 | 0.004714 | -5.12 | 3.37E-07 | <0.0001 | -0.2504 | 633 | 1236 |
|  |  |  |  |  |  |  |  |  |
| **Cortical thickness** |  |  |  |  |  |  |  |  |
| Left banks of the superior temporal sulcus | -0.043760 | 0.008923 | -4.90 | 1.03E-06 | <0.0001 | -0.2489 | 583 | 1160 |
| Left caudal anterior cingulate cortex | -0.011670 | 0.012290 | -0.95 | 3.43E-01 | 0.4892 | -0.0465 | 631 | 1234 |
| Left caudal middle frontal cortex | -0.032420 | 0.007350 | -4.41 | 1.08E-05 | 0.0001 | -0.2161 | 631 | 1229 |
| Left cuneus | -0.002703 | 0.006964 | -0.39 | 6.98E-01 | 0.7772 | -0.0192 | 624 | 1207 |
| Left entorhinal cortex | -0.079467 | 0.018909 | -4.20 | 2.77E-05 | 0.0002 | -0.2101 | 607 | 1174 |
| Left fusiform cortex | -0.056804 | 0.007526 | -7.55 | 6.96E-14 | <0.0001 | -0.3701 | 630 | 1230 |
| Left inferior parietal cortex | -0.024797 | 0.006455 | -3.84 | 1.30E-04 | 0.0007 | -0.1895 | 620 | 1219 |
| Left inferior temporal cortex | -0.039555 | 0.008339 | -4.74 | 2.27E-06 | <0.0001 | -0.2355 | 609 | 1215 |
| Left isthmus cingulate cortex | -0.031781 | 0.010036 | -3.17 | 1.60E-03 | 0.0057 | -0.1555 | 629 | 1229 |
| Left lateral occipital cortex | -0.011425 | 0.005971 | -1.91 | 5.60E-02 | 0.1204 | -0.0938 | 626 | 1231 |
| Left lateral orbitofrontal cortex | -0.018351 | 0.008171 | -2.25 | 2.50E-02 | 0.0643 | -0.1104 | 628 | 1233 |
| Left lingual cortex | -0.011957 | 0.006244 | -1.92 | 5.60E-02 | 0.1204 | -0.0942 | 629 | 1225 |
| Left medial orbitofrontal cortex | -0.017960 | 0.008182 | -2.20 | 2.83E-02 | 0.0716 | -0.1086 | 621 | 1217 |
| Left middle temporal cortex | -0.048164 | 0.008472 | -5.69 | 1.53E-08 | <0.0001 | -0.2863 | 595 | 1179 |
| Left parahippocampal cortex | -0.050969 | 0.016169 | -3.15 | 1.65E-03 | 0.0058 | -0.1550 | 624 | 1226 |
| Left paracentral cortex | -0.024312 | 0.007414 | -3.28 | 1.06E-03 | 0.0043 | -0.1605 | 632 | 1233 |
| Left pars opercularis | -0.026767 | 0.007271 | -3.68 | 2.40E-04 | 0.0012 | -0.1805 | 630 | 1225 |
| Left pars orbitalis | -0.027952 | 0.011141 | -2.51 | 1.22E-02 | 0.0342 | -0.1229 | 633 | 1229 |
| Left pars triangularis | -0.010441 | 0.008068 | -1.29 | 1.96E-01 | 0.3201 | -0.0632 | 632 | 1227 |
| Left pericalcarine | 0.004906 | 0.007050 | 0.70 | 4.87E-01 | 0.6125 | 0.0346 | 622 | 1208 |
| Left postcentral cortex | -0.007007 | 0.006164 | -1.14 | 2.56E-01 | 0.3884 | -0.0561 | 625 | 1223 |
| Left posterior cingulate cortex | -0.032728 | 0.008155 | -4.01 | 6.23E-05 | 0.0004 | -0.1964 | 631 | 1232 |
| Left precentral cortex | -0.033030 | 0.006890 | -4.80 | 1.74E-06 | <0.0001 | -0.2357 | 628 | 1226 |
| Left precuneus | -0.018522 | 0.006528 | -2.84 | 4.60E-03 | 0.0139 | -0.1393 | 629 | 1229 |
| Left rostral anterior cingulate cortex | -0.034544 | 0.011845 | -2.92 | 3.59E-03 | 0.0113 | -0.1437 | 624 | 1223 |
| Left rostral middle frontal cortex | -0.020012 | 0.006792 | -2.95 | 3.26E-03 | 0.0104 | -0.1446 | 630 | 1230 |
| Left superior frontal cortex | -0.030240 | 0.007144 | -4.23 | 2.42E-05 | 0.0002 | -0.2074 | 630 | 1230 |
| Left superior parietal cortex | -0.011045 | 0.005855 | -1.89 | 5.94E-02 | 0.1227 | -0.0929 | 627 | 1221 |
| Left superior temporal cortex | -0.053752 | 0.008404 | -6.40 | 2.05E-10 | <0.0001 | -0.3243 | 590 | 1150 |
| Left supramarginal cortex | -0.035240 | 0.006889 | -5.12 | 3.47E-07 | <0.0001 | -0.2541 | 618 | 1187 |
| Left frontal pole | -0.040825 | 0.015452 | -2.64 | 8.31E-03 | 0.0242 | -0.1291 | 633 | 1235 |
| Left temporal pole | -0.073153 | 0.018756 | -3.90 | 9.95E-05 | 0.0006 | -0.1911 | 631 | 1230 |
| Left transverse temporal cortex | -0.059959 | 0.011265 | -5.32 | 1.15E-07 | <0.0001 | -0.2603 | 633 | 1232 |
| Left insula | -0.039330 | 0.008160 | -4.82 | 1.56E-06 | <0.0001 | -0.2373 | 625 | 1219 |
| Right banks of the superior temporal sulcus | -0.042070 | 0.009030 | -4.66 | 3.40E-06 | <0.0001 | -0.2310 | 618 | 1195 |
| Right caudal anterior cingulate cortex | -0.024582 | 0.011567 | -2.13 | 3.37E-02 | 0.0802 | -0.1043 | 632 | 1231 |
| Right caudal middle frontal cortex | -0.025160 | 0.007120 | -3.53 | 4.20E-04 | 0.0019 | -0.1731 | 630 | 1228 |
| Right cuneus | -0.010167 | 0.007055 | -1.44 | 1.50E-01 | 0.2617 | -0.0711 | 622 | 1207 |
| Right entorhinal cortex | -0.046219 | 0.020478 | -2.26 | 2.41E-02 | 0.0643 | -0.1130 | 611 | 1161 |
| Right fusiform cortex | -0.053750 | 0.007610 | -7.06 | 2.34E-12 | <0.0001 | -0.3464 | 629 | 1227 |
| Right inferior parietal cortex | -0.011883 | 0.006495 | -1.83 | 6.75E-02 | 0.1358 | -0.0902 | 624 | 1217 |
| Right inferior temporal cortex | -0.040157 | 0.008597 | -4.67 | 3.21E-06 | <0.0001 | -0.2306 | 617 | 1228 |
| Right isthmus cingulate cortex | -0.024902 | 0.009800 | -2.54 | 1.10E-02 | 0.0314 | -0.1244 | 631 | 1232 |
| Right lateral occipital cortex | -0.002840 | 0.006360 | -0.45 | 6.55E-01 | 0.7451 | -0.0221 | 629 | 1230 |
| Right lateral orbitofrontal cortex | -0.026345 | 0.008235 | -3.20 | 1.40E-03 | 0.0051 | -0.1566 | 632 | 1234 |
| Right lingual cortex | -0.008546 | 0.006317 | -1.35 | 1.76E-01 | 0.2971 | -0.0665 | 624 | 1218 |
| Right medial orbitofrontal cortex | -0.027848 | 0.008400 | -3.32 | 9.30E-04 | 0.0038 | -0.1636 | 622 | 1221 |
| Right middle temporal cortex | -0.040200 | 0.008212 | -4.90 | 1.07E-06 | <0.0001 | -0.2417 | 623 | 1212 |
| Right parahippocampal cortex | -0.059251 | 0.013932 | -4.25 | 2.22E-05 | 0.0002 | -0.2085 | 629 | 1228 |
| Right paracentral cortex | -0.027483 | 0.007557 | -3.64 | 2.80E-04 | 0.0013 | -0.1783 | 630 | 1234 |
| Right pars opercularis | -0.039906 | 0.007691 | -5.19 | 2.36E-07 | <0.0001 | -0.2556 | 624 | 1220 |
| Right pars orbitalis | -0.032584 | 0.010833 | -3.01 | 2.67E-03 | 0.0089 | -0.1475 | 632 | 1224 |
| Right pars triangularis | -0.025892 | 0.008020 | -3.23 | 1.27E-03 | 0.0047 | -0.1590 | 624 | 1223 |
| Right pericalcarine | -0.005570 | 0.006968 | -0.80 | 4.20E-01 | 0.5588 | -0.0395 | 623 | 1205 |
| Right postcentral cortex | -0.005053 | 0.006169 | -0.82 | 4.13E-01 | 0.5540 | -0.0403 | 625 | 1232 |
| Right posterior cingulate cortex | -0.029891 | 0.007794 | -3.84 | 1.30E-04 | 0.0007 | -0.1881 | 630 | 1235 |
| Right precentral cortex | -0.034002 | 0.006993 | -4.86 | 1.26E-06 | <0.0001 | -0.2385 | 628 | 1230 |
| Right precuneus | -0.021397 | 0.006356 | -3.37 | 7.80E-04 | 0.0033 | -0.1650 | 632 | 1229 |
| Right rostral anterior cingulate cortex | -0.033411 | 0.011497 | -2.91 | 3.71E-03 | 0.0114 | -0.1427 | 629 | 1230 |
| Right rostral middle frontal cortex | -0.029142 | 0.006693 | -4.35 | 1.41E-05 | 0.0001 | -0.2133 | 629 | 1231 |
| Right superior frontal cortex | -0.035896 | 0.007086 | -5.07 | 4.48E-07 | <0.0001 | -0.2484 | 630 | 1235 |
| Right superior parietal cortex | -0.000033 | 0.005903 | -0.01 | 9.96E-01 | 0.9956 | -0.0005 | 630 | 1228 |
| Right superior temporal cortex | -0.056558 | 0.008147 | -6.94 | 5.42E-12 | <0.0001 | -0.3462 | 612 | 1175 |
| Right supramarginal cortex | -0.021469 | 0.006945 | -3.09 | 2.02E-03 | 0.0069 | -0.1530 | 620 | 1198 |
| Right frontal pole | -0.011130 | 0.015138 | -0.74 | 4.62E-01 | 0.5901 | -0.0362 | 633 | 1236 |
| Right temporal pole | -0.062730 | 0.019380 | -3.24 | 1.23E-03 | 0.0047 | -0.1587 | 631 | 1231 |
| Right transverse temporal cortex | -0.042906 | 0.011620 | -3.69 | 2.30E-04 | 0.0012 | -0.1807 | 631 | 1233 |
| Right insula | -0.028980 | 0.008590 | -3.37 | 7.60E-04 | 0.0033 | -0.1665 | 620 | 1213 |
|  |  |  |  |  |  |  |  |  |
| **Subcortical volumes** |  |  |  |  |  |  |  |  |
| Left lateral ventricle | 385.674063 | 239.176794 | 1.61 | 1.07E-01 | 0.1976 | 0.0807 | 604 | 1173 |
| Right lateral ventricle | 466.713986 | 217.682037 | 2.14 | 3.22E-02 | 0.0778 | 0.1075 | 601 | 1169 |
| Left thalamus | 136.055361 | 37.400913 | 3.64 | 2.80E-04 | 0.0013 | 0.1830 | 601 | 1163 |
| Right thalamus | 87.561337 | 31.250629 | 2.80 | 5.10E-03 | 0.0151 | 0.1407 | 601 | 1163 |
| Left caudate | 6.125204 | 21.947971 | 0.28 | 7.80E-01 | 0.8342 | 0.0140 | 607 | 1161 |
| Right caudate | 1.598154 | 21.829351 | 0.07 | 9.42E-01 | 0.9589 | 0.0035 | 600 | 1161 |
| Left putamen | 11.584444 | 30.710253 | 0.38 | 7.06E-01 | 0.7806 | 0.0194 | 586 | 1131 |
| Right putamen | -25.521129 | 28.150857 | -0.91 | 3.65E-01 | 0.4987 | -0.0463 | 586 | 1144 |
| Left pallidum | -13.126749 | 11.584643 | -1.13 | 2.57E-01 | 0.3884 | -0.0585 | 560 | 1122 |
| Right pallidum | -0.352357 | 9.861574 | -0.04 | 9.72E-01 | 0.9777 | -0.0020 | 592 | 1154 |
| Left hippocampus | 18.195989 | 19.982939 | 0.91 | 3.63E-01 | 0.4987 | 0.0459 | 594 | 1162 |
| Right hippocamups | 33.248576 | 19.735539 | 1.68 | 9.22E-02 | 0.1744 | 0.0845 | 598 | 1173 |
| Left amygdala | 17.894603 | 9.225695 | 1.94 | 5.26E-02 | 0.1180 | 0.0978 | 596 | 1165 |
| Right amygdala | 38.389191 | 10.033942 | 3.83 | 1.30E-04 | 0.0007 | 0.1924 | 599 | 1173 |
| Left nucleus accumbens | 9.320999 | 5.129854 | 1.82 | 6.94E-02 | 0.1369 | 0.0922 | 588 | 1156 |
| Right nucleus accumbens | -3.081495 | 4.543504 | -0.68 | 4.98E-01 | 0.6202 | -0.0343 | 595 | 1158 |
|  |  |  |  |  |  |  |  |  |
| **Cortical surface area** |  |  |  |  |  |  |  |  |
| Left banks of the superior temporal sulcus | -10.549288 | 7.932213 | -1.33 | 1.84E-01 | 0.3068 | -0.0680 | 575 | 1150 |
| Left caudal anterior cingulate cortex | 8.739443 | 7.187004 | 1.22 | 2.24E-01 | 0.3590 | 0.0599 | 627 | 1230 |
| Left caudal middle frontal cortex | 10.456001 | 17.130780 | 0.61 | 5.42E-01 | 0.6593 | 0.0299 | 628 | 1226 |
| Left cuneus | -14.835300 | 10.747985 | -1.38 | 1.68E-01 | 0.2893 | -0.0681 | 623 | 1206 |
| Left entorhinal cortex | -1.903877 | 4.133969 | -0.46 | 6.45E-01 | 0.7448 | -0.0232 | 595 | 1161 |
| Left fusiform cortex | -30.502038 | 17.870398 | -1.71 | 8.80E-02 | 0.1685 | -0.0843 | 622 | 1223 |
| Left inferior parietal cortex | -30.422156 | 28.262293 | -1.08 | 2.82E-01 | 0.4136 | -0.0534 | 620 | 1212 |
| Left inferior temporal cortex | -12.168190 | 21.671596 | -0.56 | 5.75E-01 | 0.6886 | -0.0278 | 609 | 1216 |
| Left isthmus cingulate cortex | 26.419069 | 7.761966 | 3.40 | 6.80E-04 | 0.0031 | 0.1667 | 630 | 1227 |
| Left lateral occipital cortex | -31.615655 | 27.173180 | -1.16 | 2.45E-01 | 0.3843 | -0.0570 | 626 | 1228 |
| Left lateral orbitofrontal cortex | 8.104674 | 13.032631 | 0.62 | 5.34E-01 | 0.6551 | 0.0304 | 631 | 1234 |
| Left lingual cortex | -30.492813 | 19.453245 | -1.57 | 1.17E-01 | 0.2115 | -0.0770 | 629 | 1226 |
| Left medial orbitofrontal cortex | 9.901536 | 10.811809 | 0.92 | 3.60E-01 | 0.4987 | 0.0455 | 620 | 1211 |
| Left middle temporal cortex | -7.703968 | 17.979254 | -0.43 | 6.68E-01 | 0.7495 | -0.0217 | 590 | 1172 |
| Left parahippocampal cortex | 5.353947 | 4.961463 | 1.08 | 2.81E-01 | 0.4136 | 0.0532 | 621 | 1223 |
| Left paracentral cortex | 19.626324 | 9.059177 | 2.17 | 3.04E-02 | 0.0746 | 0.1068 | 625 | 1222 |
| Left pars opercularis | 9.341699 | 12.225034 | 0.76 | 4.45E-01 | 0.5762 | 0.0374 | 627 | 1222 |
| Left pars orbitalis | 2.295703 | 3.912040 | 0.59 | 5.57E-01 | 0.6732 | 0.0289 | 634 | 1229 |
| Left pars triangularis | 4.187026 | 9.676173 | 0.43 | 6.65E-01 | 0.7495 | 0.0211 | 632 | 1225 |
| Left pericalcarine | -26.173239 | 11.652363 | -2.25 | 2.48E-02 | 0.0643 | -0.1109 | 625 | 1208 |
| Left postcentral cortex | 10.087252 | 19.723890 | 0.51 | 6.09E-01 | 0.7190 | 0.0252 | 620 | 1214 |
| Left posterior cingulate cortex | 9.482873 | 8.354516 | 1.14 | 2.57E-01 | 0.3884 | 0.0559 | 631 | 1230 |
| Left precentral cortex | 11.441090 | 23.587005 | 0.49 | 6.28E-01 | 0.7354 | 0.0241 | 625 | 1218 |
| Left precuneus | 31.199962 | 20.372846 | 1.53 | 1.26E-01 | 0.2219 | 0.0750 | 630 | 1229 |
| Left rostral anterior cingulate cortex | 13.603719 | 7.161512 | 1.90 | 5.77E-02 | 0.1223 | 0.0940 | 618 | 1212 |
| Left rostral middle frontal cortex | -21.037541 | 31.507753 | -0.67 | 5.04E-01 | 0.6236 | -0.0328 | 632 | 1229 |
| Left superior frontal cortex | 67.812809 | 34.044965 | 1.99 | 4.65E-02 | 0.1075 | 0.0980 | 623 | 1225 |
| Left superior parietal cortex | 49.568181 | 28.366189 | 1.75 | 8.07E-02 | 0.1565 | 0.0862 | 624 | 1218 |
| Left superior temporal cortex | 39.680473 | 20.027109 | 1.98 | 4.77E-02 | 0.1086 | 0.1007 | 585 | 1145 |
| Left supramarginal cortex | 40.459655 | 25.179069 | 1.61 | 1.08E-01 | 0.1977 | 0.0803 | 612 | 1176 |
| Left frontal pole | 1.721616 | 1.748525 | 0.98 | 3.25E-01 | 0.4680 | 0.0479 | 634 | 1235 |
| Left temporal pole | -1.596792 | 3.008509 | -0.53 | 5.96E-01 | 0.7085 | -0.0260 | 631 | 1230 |
| Left transverse temporal cortex | 10.917434 | 3.650982 | 2.99 | 2.83E-03 | 0.0093 | 0.1462 | 634 | 1235 |
| Left insula | 12.345810 | 11.152567 | 1.11 | 2.68E-01 | 0.4014 | 0.0546 | 626 | 1220 |
| Right banks of the superior temporal sulcus | 0.462886 | 6.684895 | 0.07 | 9.45E-01 | 0.9589 | 0.0035 | 614 | 1192 |
| Right caudal anterior cingulate cortex | 5.972686 | 7.684804 | 0.78 | 4.37E-01 | 0.5762 | 0.0383 | 627 | 1230 |
| Right caudal middle frontal cortex | -5.513040 | 17.495747 | -0.32 | 7.53E-01 | 0.8207 | -0.0157 | 629 | 1226 |
| Right cuneus | -2.665662 | 9.859730 | -0.27 | 7.87E-01 | 0.8348 | -0.0133 | 623 | 1205 |
| Right entorhinal cortex | -5.202209 | 4.272816 | -1.22 | 2.24E-01 | 0.3590 | -0.0619 | 589 | 1149 |
| Right fusiform cortex | -7.755149 | 17.178553 | -0.45 | 6.52E-01 | 0.7451 | -0.0223 | 613 | 1223 |
| Right inferior parietal cortex | -8.837258 | 31.786985 | -0.28 | 7.81E-01 | 0.8342 | -0.0138 | 625 | 1208 |
| Right inferior temporal cortex | -35.935349 | 19.548405 | -1.84 | 6.62E-02 | 0.1350 | -0.0908 | 618 | 1226 |
| Right isthmus cingulate cortex | 23.540311 | 7.235008 | 3.25 | 1.16E-03 | 0.0046 | 0.1590 | 633 | 1231 |
| Right lateral occipital cortex | -31.187068 | 27.470976 | -1.14 | 2.56E-01 | 0.3884 | -0.0559 | 628 | 1232 |
| Right lateral orbitofrontal cortex | 12.567023 | 13.877928 | 0.91 | 3.65E-01 | 0.4987 | 0.0445 | 633 | 1234 |
| Right lingual cortex | -34.329289 | 18.917736 | -1.81 | 6.98E-02 | 0.1369 | -0.0890 | 626 | 1221 |
| Right medial orbitofrontal cortex | 3.414728 | 9.593600 | 0.36 | 7.22E-01 | 0.7926 | 0.0178 | 621 | 1221 |
| Right middle temporal cortex | -3.975065 | 18.068492 | -0.22 | 8.26E-01 | 0.8702 | -0.0109 | 621 | 1203 |
| Right parahippocampal cortex | 0.612401 | 4.614320 | 0.13 | 8.94E-01 | 0.9239 | 0.0064 | 626 | 1224 |
| Right paracentral cortex | 22.660036 | 10.072825 | 2.25 | 2.46E-02 | 0.0643 | 0.1105 | 626 | 1231 |
| Right pars opercularis | 15.084320 | 11.538286 | 1.31 | 1.91E-01 | 0.3161 | 0.0645 | 624 | 1216 |
| Right pars orbitalis | -2.188403 | 4.609163 | -0.47 | 6.35E-01 | 0.7385 | -0.0230 | 633 | 1222 |
| Right pars triangularis | -3.238258 | 11.164822 | -0.29 | 7.72E-01 | 0.8342 | -0.0143 | 625 | 1219 |
| Right pericalcarine | -25.266816 | 12.146303 | -2.08 | 3.77E-02 | 0.0882 | -0.1026 | 625 | 1204 |
| Right postcentral cortex | 1.368534 | 20.472364 | 0.07 | 9.47E-01 | 0.9589 | 0.0035 | 620 | 1229 |
| Right posterior cingulate cortex | 15.481498 | 8.199051 | 1.89 | 5.92E-02 | 0.1227 | 0.0925 | 631 | 1234 |
| Right precentral cortex | 16.400423 | 23.624885 | 0.69 | 4.88E-01 | 0.6125 | 0.0339 | 626 | 1227 |
| Right precuneus | 35.528125 | 21.347457 | 1.66 | 9.62E-02 | 0.1799 | 0.0813 | 633 | 1229 |
| Right rostral anterior cingulate cortex | 6.639279 | 6.549302 | 1.01 | 3.11E-01 | 0.4518 | 0.0497 | 625 | 1227 |
| Right rostral middle frontal cortex | -43.234911 | 31.766420 | -1.36 | 1.74E-01 | 0.2964 | -0.0666 | 631 | 1228 |
| Right superior frontal cortex | 53.512591 | 34.546235 | 1.55 | 1.22E-01 | 0.2169 | 0.0763 | 622 | 1231 |
| Right superior parietal cortex | 20.682934 | 27.022799 | 0.77 | 4.44E-01 | 0.5762 | 0.0378 | 627 | 1222 |
| Right superior temporal cortex | 33.691016 | 17.565097 | 1.92 | 5.53E-02 | 0.1204 | 0.0960 | 609 | 1171 |
| Right supramarginal cortex | -4.572376 | 23.495547 | -0.19 | 8.46E-01 | 0.8852 | -0.0094 | 618 | 1193 |
| Right frontal pole | -0.323735 | 2.155210 | -0.15 | 8.81E-01 | 0.9156 | -0.0073 | 633 | 1236 |
| Right temporal pole | 2.821002 | 3.152096 | 0.89 | 3.71E-01 | 0.5020 | 0.0438 | 624 | 1228 |
| Right transverse temporal cortex | 5.983491 | 2.747596 | 2.18 | 2.96E-02 | 0.0737 | 0.1066 | 633 | 1236 |
| Right insula | 9.497205 | 12.507438 | 0.76 | 4.48E-01 | 0.5762 | 0.0375 | 621 | 1212 |

| **Label** | **Estimate** | **StdError** | **T** | **p** | **FDR adjusted p** | **Cohen´s d** | **N** |
| --- | --- | --- | --- | --- | --- | --- | --- |
|  |  |  |  |  |  |  |  |
| **Global measures** |  |  |  |  |  |  |  |
| Total Intracranial Volume | -2094.00000 | 820.000000 | -2.55 | 1.07E-02 | 0.0335 | -0.0654 | 6112 |
| Total left hemispheral surface area | -6.780000 | 37.970000 | -0.18 | 8.58E-01 | 0.8985 | -0.0046 | 6287 |
| Total right hemispheral surface area | -5.060000 | 38.060000 | -0.13 | 8.94E-01 | 0.9266 | -0.0033 | 6287 |
| Left hemispheral average thickness | 0.002327 | 0.000489 | 4.76 | 1.98E-06 | 0.0001 | 0.1202 | 6306 |
| Right hemispheral average thickness | 0.002526 | 0.000488 | 5.17 | 2.39E-07 | <0.0001 | 0.1306 | 6305 |
|  |  |  |  |  |  |  |  |
| **Cortical thickness** |  |  |  |  |  |  |  |
| Left banks of the superior temporal sulcus | 0.003666 | 0.000938 | 3.91 | 9.36E-05 | 0.0009 | 0.1014 | 5977 |
| Left caudal anterior cingulate cortex | 0.000870 | 0.001288 | 0.68 | 4.99E-01 | 0.6036 | 0.0172 | 6285 |
| Left caudal middle frontal gyrus | 0.002605 | 0.000776 | 3.35 | 8.00E-04 | 0.0045 | 0.0849 | 6263 |
| Left cuneus | 0.001628 | 0.000717 | 2.27 | 2.32E-02 | 0.0587 | 0.0578 | 6195 |
| Left entorhinal cortex | 0.000164 | 0.001944 | 0.08 | 9.33E-01 | 0.9448 | 0.0021 | 6066 |
| Left fusiform gyrus | 0.002637 | 0.000769 | 3.43 | 6.10E-04 | 0.0040 | 0.0868 | 6276 |
| Left inferior parietal cortex | 0.002992 | 0.000695 | 4.31 | 1.68E-05 | 0.0003 | 0.1096 | 6220 |
| Left inferior temporal gyrus | 0.000896 | 0.000877 | 1.02 | 3.07E-01 | 0.4155 | 0.0260 | 6193 |
| Left isthmus cingulate cortex | 0.002695 | 0.001044 | 2.58 | 9.90E-03 | 0.0331 | 0.0653 | 6284 |
| Left lateral occipital cortex | 0.001809 | 0.000637 | 2.84 | 4.56E-03 | 0.0181 | 0.0719 | 6267 |
| Left lateral orbitofrontal cortex | 0.003960 | 0.000860 | 4.60 | 4.25E-06 | 0.0001 | 0.1165 | 6267 |
| Left lingual gyrus | 0.001665 | 0.000649 | 2.57 | 1.03E-02 | 0.0335 | 0.0652 | 6254 |
| Left medial orbitofrontal cortex | 0.001614 | 0.000859 | 1.88 | 6.03E-02 | 0.1246 | 0.0478 | 6214 |
| Left middle temporal gyrus | 0.002289 | 0.000894 | 2.56 | 1.05E-02 | 0.0335 | 0.0659 | 6063 |
| Left parahippcampal gyrus | 0.000168 | 0.001669 | 0.10 | 9.20E-01 | 0.9376 | 0.0025 | 6275 |
| Left paracentral lobule | 0.000096 | 0.000797 | 0.12 | 9.04E-01 | 0.9276 | 0.0030 | 6282 |
| Left pars opercularis | 0.002974 | 0.000775 | 3.84 | 1.30E-04 | 0.0011 | 0.0973 | 6264 |
| Left pars orbitalis | 0.003731 | 0.001175 | 3.18 | 1.50E-03 | 0.0071 | 0.0805 | 6279 |
| Left pars triangularis | 0.001866 | 0.000843 | 2.21 | 2.70E-02 | 0.0661 | 0.0560 | 6268 |
| Left pericalcarine | 0.001256 | 0.000700 | 1.79 | 7.30E-02 | 0.1433 | 0.0456 | 6190 |
| Left precentral gyrus | 0.002136 | 0.000632 | 3.38 | 7.30E-04 | 0.0045 | 0.0859 | 6225 |
| Left posterior cingulate cortex | 0.001417 | 0.000839 | 1.69 | 9.10E-02 | 0.1709 | 0.0427 | 6287 |
| Left precentral gyrus | 0.002197 | 0.000737 | 2.98 | 2.88E-03 | 0.0126 | 0.0756 | 6246 |
| Left precuneus | 0.002109 | 0.000679 | 3.11 | 1.90E-03 | 0.0088 | 0.0787 | 6272 |
| Left rostral anterior cingulate cortex | 0.003380 | 0.001240 | 2.73 | 6.30E-03 | 0.0230 | 0.0692 | 6255 |
| Left rostral middle frontal gyrus | 0.003347 | 0.000720 | 4.65 | 3.45E-06 | 0.0001 | 0.1177 | 6276 |
| Left superior frontal gyrus | 0.003032 | 0.000753 | 4.03 | 5.72E-05 | 0.0006 | 0.1020 | 6272 |
| Left superior parietal cortex | 0.001733 | 0.000621 | 2.79 | 5.30E-03 | 0.0198 | 0.0708 | 6239 |
| Left superior temporal gyrus | 0.002891 | 0.000862 | 3.35 | 8.10E-04 | 0.0045 | 0.0871 | 5949 |
| Left supramarginal gyrus | 0.003614 | 0.000723 | 5.00 | 5.88E-07 | <0.0001 | 0.1281 | 6128 |
| Left frontal pole | 0.002646 | 0.001606 | 1.65 | 9.95E-02 | 0.1796 | 0.0417 | 6300 |
| Left temporal pole | 0.001969 | 0.001972 | 1.00 | 3.18E-01 | 0.4233 | 0.0253 | 6262 |
| Left transverse temporal gyrus | 0.001178 | 0.001160 | 1.02 | 3.10E-01 | 0.4160 | 0.0258 | 6276 |
| Left insula | 0.001215 | 0.000860 | 1.41 | 1.58E-01 | 0.2476 | 0.0359 | 6217 |
| Right banks of the superior temporal sulcus | 0.003074 | 0.000926 | 3.32 | 9.10E-04 | 0.0047 | 0.0848 | 6165 |
| Right caudal anterior cingulate cortex | 0.001362 | 0.001197 | 1.14 | 2.55E-01 | 0.3577 | 0.0288 | 6288 |
| Right caudal middle frontal gyrus | 0.003186 | 0.000773 | 4.12 | 3.83E-05 | 0.0004 | 0.1043 | 6271 |
| Right cuneus | 0.003147 | 0.000721 | 4.37 | 1.29E-05 | 0.0003 | 0.1113 | 6199 |
| Right entorhinal cortex | 0.002169 | 0.002088 | 1.04 | 2.99E-01 | 0.4080 | 0.0269 | 6010 |
| Right fusiform gyrus | 0.002809 | 0.000780 | 3.60 | 3.20E-04 | 0.0023 | 0.0911 | 6281 |
| Right inferior parietal cortex | 0.002257 | 0.000682 | 3.31 | 9.30E-04 | 0.0047 | 0.0841 | 6224 |
| Right inferior temporal gyrus | 0.002954 | 0.000891 | 3.31 | 9.20E-04 | 0.0047 | 0.0841 | 6227 |
| Right isthmus cingulate cortex | 0.002338 | 0.001016 | 2.30 | 2.15E-02 | 0.0553 | 0.0582 | 6284 |
| Right lateral occipital cortex | 0.001275 | 0.000660 | 1.93 | 5.30E-02 | 0.1124 | 0.0489 | 6271 |
| Right lateral orbitofrontal cortex | 0.002805 | 0.000858 | 3.27 | 1.10E-03 | 0.0054 | 0.0827 | 6283 |
| Right lingual gyrus | 0.002242 | 0.000651 | 3.45 | 5.70E-04 | 0.0039 | 0.0876 | 6239 |
| Right medial orbitofrontal cortex | 0.003458 | 0.000887 | 3.90 | 9.77E-05 | 0.0009 | 0.0990 | 6237 |
| Right middle temporal gyrus | 0.002155 | 0.000862 | 2.50 | 1.24E-02 | 0.0376 | 0.0636 | 6212 |
| Right parahippcampal gyrus | 0.001920 | 0.001430 | 1.34 | 1.80E-01 | 0.2786 | 0.0339 | 6280 |
| Right paracentral lobule | 0.001174 | 0.000798 | 1.47 | 1.41E-01 | 0.2362 | 0.0372 | 6286 |
| Right pars opercularis | 0.002443 | 0.000812 | 3.01 | 2.64E-03 | 0.0118 | 0.0764 | 6242 |
| Right pars orbitalis | 0.002689 | 0.001150 | 2.34 | 1.94E-02 | 0.0516 | 0.0592 | 6277 |
| Right pars triangularis | 0.002317 | 0.000826 | 2.80 | 5.10E-03 | 0.0195 | 0.0711 | 6241 |
| Right pericalcarine | 0.002380 | 0.000708 | 3.36 | 7.90E-04 | 0.0045 | 0.0858 | 6172 |
| Right precentral gyrus | 0.002675 | 0.000636 | 4.21 | 2.62E-05 | 0.0004 | 0.1068 | 6245 |
| Right posterior cingulate cortex | 0.001585 | 0.000814 | 1.95 | 5.20E-02 | 0.1118 | 0.0493 | 6290 |
| Right precentral gyrus | 0.001297 | 0.000743 | 1.74 | 8.11E-02 | 0.1571 | 0.0441 | 6254 |
| Right precuneus | 0.002796 | 0.000671 | 4.17 | 3.11E-05 | 0.0004 | 0.1056 | 6274 |
| Right rostral anterior cingulate cortex | 0.003253 | 0.001215 | 2.68 | 7.43E-03 | 0.0265 | 0.0679 | 6258 |
| Right rostral middle frontal gyrus | 0.003018 | 0.000716 | 4.21 | 2.55E-05 | 0.0004 | 0.1066 | 6269 |
| Right superior frontal gyrus | 0.003181 | 0.000753 | 4.23 | 2.40E-05 | 0.0004 | 0.1071 | 6277 |
| Right superior parietal cortex | 0.001272 | 0.000620 | 2.05 | 4.00E-02 | 0.0897 | 0.0520 | 6256 |
| Right superior temporal gyrus | 0.003136 | 0.000842 | 3.72 | 2.00E-04 | 0.0015 | 0.0957 | 6081 |
| Right supramarginal gyrus | 0.003339 | 0.000726 | 4.60 | 4.29E-06 | 0.0001 | 0.1177 | 6145 |
| Right frontal pole | 0.003327 | 0.001585 | 2.10 | 3.59E-02 | 0.0828 | 0.0531 | 6296 |
| Right temporal pole | 0.002568 | 0.002038 | 1.26 | 2.08E-01 | 0.3077 | 0.0319 | 6267 |
| Right transverse temporal gyrus | 0.000937 | 0.001222 | 0.77 | 4.43E-01 | 0.5525 | 0.0195 | 6267 |
| Right insula | 0.001556 | 0.000913 | 1.70 | 8.84E-02 | 0.1693 | 0.0434 | 6165 |
|  |  |  |  |  |  |  |  |
| **Subcortical volumes** |  |  |  |  |  |  |  |
| Left lateral ventricle | -56.263118 | 24.858388 | -2.26 | 2.37E-02 | 0.0589 | -0.0586 | 5991 |
| Right lateral ventricle | -60.049175 | 23.117157 | -2.60 | 9.41E-03 | 0.0321 | -0.0675 | 5976 |
| Left thalamus | 5.467060 | 3.777183 | 1.45 | 1.48E-01 | 0.2393 | 0.0376 | 5971 |
| Right thalamus | 3.754810 | 3.259570 | 1.15 | 2.49E-01 | 0.3527 | 0.0299 | 5957 |
| Left caudate | 4.196276 | 2.213210 | 1.90 | 5.80E-02 | 0.1214 | 0.0493 | 5968 |
| Right caudate | 3.579537 | 2.234748 | 1.60 | 1.09E-01 | 0.1949 | 0.0417 | 5914 |
| Left putamen | 2.914209 | 3.023627 | 0.96 | 3.35E-01 | 0.4349 | 0.0253 | 5773 |
| Right putamen | 6.664575 | 2.811943 | 2.37 | 1.78E-02 | 0.0483 | 0.0622 | 5842 |
| Left pallidum | 3.359704 | 1.143224 | 2.94 | 3.31E-03 | 0.0140 | 0.0781 | 5702 |
| Right pallidum | 2.418579 | 1.020688 | 2.37 | 1.78E-02 | 0.0483 | 0.0618 | 5925 |
| Left hippocampus | 2.722418 | 2.033271 | 1.34 | 1.81E-01 | 0.2786 | 0.0349 | 5943 |
| Right hippocamups | 2.258446 | 2.020834 | 1.12 | 2.64E-01 | 0.3665 | 0.0290 | 5994 |
| Left amygdala | 1.606488 | 0.956948 | 1.68 | 9.33E-02 | 0.1710 | 0.0437 | 5956 |
| Right amygdala | 0.997927 | 1.017983 | 0.98 | 3.27E-01 | 0.4314 | 0.0254 | 5967 |
| Left nucleus accumbens | 1.035266 | 0.510389 | 2.03 | 4.26E-02 | 0.0941 | 0.0532 | 5856 |
| Right nucleus accumbens | 1.323421 | 0.466808 | 2.84 | 4.60E-03 | 0.0181 | 0.0743 | 5879 |
|  |  |  |  |  |  |  |  |
| **Cortical surface area** |  |  |  |  |  |  |  |
| Left banks of the superior temporal sulcus | -0.582927 | 0.857439 | -0.68 | 4.97E-01 | 0.6036 | -0.0183 | 5566 |
| Left caudal anterior cingulate cortex | 1.890406 | 0.742232 | 2.55 | 1.09E-02 | 0.0335 | 0.0666 | 5904 |
| Left caudal middle frontal gyrus | 3.485747 | 1.867634 | 1.87 | 6.20E-02 | 0.1264 | 0.0488 | 5904 |
| Left cuneus | 1.395612 | 1.113730 | 1.25 | 2.10E-01 | 0.3085 | 0.0328 | 5859 |
| Left entorhinal cortex | 0.414944 | 0.429676 | 0.97 | 3.34E-01 | 0.4349 | 0.0261 | 5555 |
| Left fusiform gyrus | 1.455878 | 1.880362 | 0.77 | 4.39E-01 | 0.5511 | 0.0203 | 5799 |
| Left inferior parietal cortex | 5.082967 | 3.011105 | 1.69 | 9.15E-02 | 0.1709 | 0.0443 | 5853 |
| Left inferior temporal gyrus | -1.409486 | 2.237550 | -0.63 | 5.29E-01 | 0.6289 | -0.0165 | 5876 |
| Left isthmus cingulate cortex | 0.397457 | 0.812644 | 0.49 | 6.25E-01 | 0.7108 | 0.0127 | 5962 |
| Left lateral occipital cortex | 4.162874 | 2.853560 | 1.46 | 1.45E-01 | 0.2371 | 0.0380 | 5946 |
| Left lateral orbitofrontal cortex | 1.899493 | 1.321889 | 1.44 | 1.51E-01 | 0.2406 | 0.0373 | 5979 |
| Left lingual gyrus | 2.196599 | 2.028359 | 1.08 | 2.79E-01 | 0.3841 | 0.0281 | 5944 |
| Left medial orbitofrontal cortex | -0.351330 | 1.145507 | -0.31 | 7.59E-01 | 0.8219 | -0.0081 | 5841 |
| Left middle temporal gyrus | 0.248745 | 1.922597 | 0.13 | 8.97E-01 | 0.9266 | 0.0035 | 5678 |
| Left parahippcampal gyrus | 1.177986 | 0.477183 | 2.47 | 1.36E-02 | 0.0398 | 0.0645 | 5897 |
| Left paracentral lobule | 1.486412 | 0.967201 | 1.54 | 1.24E-01 | 0.2146 | 0.0404 | 5838 |
| Left pars opercularis | 0.738634 | 1.323418 | 0.56 | 5.77E-01 | 0.6658 | 0.0146 | 5921 |
| Left pars orbitalis | 0.528549 | 0.412661 | 1.28 | 2.00E-01 | 0.3001 | 0.0332 | 5968 |
| Left pars triangularis | 0.935189 | 1.015210 | 0.92 | 3.57E-01 | 0.4594 | 0.0239 | 5943 |
| Left pericalcarine | -0.712014 | 1.232509 | -0.58 | 5.63E-01 | 0.6553 | -0.0152 | 5888 |
| Left precentral gyrus | 3.882163 | 2.112839 | 1.84 | 6.62E-02 | 0.1332 | 0.0483 | 5838 |
| Left posterior cingulate cortex | 0.587961 | 0.886558 | 0.66 | 5.07E-01 | 0.6079 | 0.0171 | 5965 |
| Left precentral gyrus | 5.961154 | 2.416596 | 2.47 | 1.37E-02 | 0.0398 | 0.0646 | 5881 |
| Left precuneus | 4.163436 | 2.093622 | 1.99 | 4.70E-02 | 0.1025 | 0.0517 | 5949 |
| Left rostral anterior cingulate cortex | 2.021191 | 0.759045 | 2.66 | 7.77E-03 | 0.0271 | 0.0700 | 5816 |
| Left rostral middle frontal gyrus | 9.412579 | 3.303018 | 2.85 | 4.39E-03 | 0.0181 | 0.0741 | 5952 |
| Left superior frontal gyrus | 6.384922 | 3.526891 | 1.81 | 7.03E-02 | 0.1397 | 0.0474 | 5859 |
| Left superior parietal cortex | 4.657582 | 3.061197 | 1.52 | 1.28E-01 | 0.2164 | 0.0398 | 5875 |
| Left superior temporal gyrus | 0.488151 | 2.046686 | 0.24 | 8.12E-01 | 0.8608 | 0.0064 | 5577 |
| Left supramarginal gyrus | -1.586499 | 2.654032 | -0.60 | 5.50E-01 | 0.6493 | -0.0159 | 5701 |
| Left frontal pole | 0.239935 | 0.183253 | 1.31 | 1.90E-01 | 0.2903 | 0.0339 | 5990 |
| Left temporal pole | 0.150491 | 0.316574 | 0.48 | 6.35E-01 | 0.7116 | 0.0125 | 5954 |
| Left transverse temporal gyrus | -0.092589 | 0.382154 | -0.24 | 8.09E-01 | 0.8608 | -0.0062 | 5986 |
| Left insula | 4.804266 | 1.151545 | 4.17 | 3.06E-05 | 0.0004 | 0.1089 | 5899 |
| Right banks of the superior temporal sulcus | 0.837256 | 0.695364 | 1.20 | 2.29E-01 | 0.3263 | 0.0316 | 5804 |
| Right caudal anterior cingulate cortex | 0.598207 | 0.803191 | 0.74 | 4.56E-01 | 0.5642 | 0.0193 | 5930 |
| Right caudal middle frontal gyrus | 3.939023 | 1.852173 | 2.13 | 3.35E-02 | 0.0785 | 0.0555 | 5922 |
| Right cuneus | 1.636059 | 1.048273 | 1.56 | 1.19E-01 | 0.2093 | 0.0408 | 5874 |
| Right entorhinal cortex | 0.557350 | 0.435512 | 1.28 | 2.01E-01 | 0.3001 | 0.0347 | 5468 |
| Right fusiform gyrus | 4.026650 | 1.851563 | 2.17 | 2.97E-02 | 0.0717 | 0.0571 | 5817 |
| Right inferior parietal cortex | 1.579498 | 3.277868 | 0.48 | 6.30E-01 | 0.7115 | 0.0126 | 5850 |
| Right inferior temporal gyrus | 1.839515 | 2.092255 | 0.88 | 3.79E-01 | 0.4837 | 0.0230 | 5911 |
| Right isthmus cingulate cortex | 0.028988 | 0.745861 | 0.04 | 9.69E-01 | 0.9690 | 0.0010 | 5976 |
| Right lateral occipital cortex | 1.064106 | 2.849206 | 0.37 | 7.09E-01 | 0.7809 | 0.0096 | 5962 |
| Right lateral orbitofrontal cortex | 5.381874 | 1.419622 | 3.79 | 1.50E-04 | 0.0012 | 0.0983 | 5984 |
| Right lingual gyrus | -1.060776 | 1.999819 | -0.53 | 5.96E-01 | 0.6828 | -0.0138 | 5942 |
| Right medial orbitofrontal cortex | 0.068274 | 0.988008 | 0.07 | 9.45E-01 | 0.9510 | 0.0018 | 5909 |
| Right middle temporal gyrus | 1.101075 | 1.864333 | 0.59 | 5.55E-01 | 0.6500 | 0.0155 | 5839 |
| Right parahippcampal gyrus | 1.828247 | 0.474918 | 3.85 | 1.20E-04 | 0.0010 | 0.1004 | 5909 |
| Right paracentral lobule | 0.354857 | 1.078302 | 0.33 | 7.42E-01 | 0.8091 | 0.0086 | 5904 |
| Right pars opercularis | 0.800018 | 1.185481 | 0.67 | 5.00E-01 | 0.6036 | 0.0175 | 5897 |
| Right pars orbitalis | 1.003046 | 0.482686 | 2.08 | 3.78E-02 | 0.0859 | 0.0540 | 5964 |
| Right pars triangularis | 1.471076 | 1.188385 | 1.24 | 2.16E-01 | 0.3108 | 0.0324 | 5909 |
| Right pericalcarine | -0.518802 | 1.280182 | -0.41 | 6.85E-01 | 0.7631 | -0.0107 | 5882 |
| Right precentral gyrus | 3.522018 | 2.100585 | 1.68 | 9.37E-02 | 0.1710 | 0.0439 | 5883 |
| Right posterior cingulate cortex | 1.242219 | 0.866416 | 1.43 | 1.52E-01 | 0.2406 | 0.0371 | 5972 |
| Right precentral gyrus | 6.135732 | 2.508355 | 2.45 | 1.45E-02 | 0.0413 | 0.0640 | 5894 |
| Right precuneus | 1.917531 | 2.193453 | 0.87 | 3.82E-01 | 0.4837 | 0.0226 | 5959 |
| Right rostral anterior cingulate cortex | 1.481351 | 0.692398 | 2.14 | 3.24E-02 | 0.0772 | 0.0559 | 5894 |
| Right rostral middle frontal gyrus | 8.130382 | 3.378427 | 2.41 | 1.61E-02 | 0.0452 | 0.0627 | 5947 |
| Right superior frontal gyrus | 5.449815 | 3.571644 | 1.53 | 1.27E-01 | 0.2164 | 0.0400 | 5897 |
| Right superior parietal cortex | -1.076755 | 2.908915 | -0.37 | 7.11E-01 | 0.7809 | -0.0097 | 5903 |
| Right superior temporal gyrus | 4.201291 | 1.801330 | 2.33 | 1.97E-02 | 0.0516 | 0.0619 | 5707 |
| Right supramarginal gyrus | 3.608133 | 2.471489 | 1.46 | 1.44E-01 | 0.2371 | 0.0386 | 5760 |
| Right frontal pole | -0.286380 | 0.229991 | -1.25 | 2.13E-01 | 0.3098 | -0.0324 | 5989 |
| Right temporal pole | -0.099487 | 0.346954 | -0.29 | 7.74E-01 | 0.8327 | -0.0076 | 5891 |
| Right transverse temporal gyrus | 0.066923 | 0.292730 | 0.23 | 8.19E-01 | 0.8632 | 0.0060 | 5991 |
| Right insula | 1.999775 | 1.301203 | 1.54 | 1.24E-01 | 0.2146 | 0.0404 | 5849 |

| **Label** | **Estimate** | **StdError** | **T** | **p** | **FDR adjusted p** | **Cohen´s d** | **N Obese** | **N NW** |
| --- | --- | --- | --- | --- | --- | --- | --- | --- |
|  |  |  |  |  |  |  |  |  |
| **Global measures** |  |  |  |  |  |  |  |  |
| Total Intracranial Volume | 1347.000000 | 371.000000 | 3.63 | 2.90E-04 | 0.0152 | 0.1164 | 1168 | 2755 |
| Total left hemispheral surface area | 31.200000 | 17.100000 | 1.83 | 6.72E-02 | 0.3019 | 0.0577 | 1189 | 2872 |
| Total right hemispheral surface area | 27.100000 | 17.100000 | 1.58 | 1.14E-01 | 0.3980 | 0.0498 | 1189 | 2872 |
| Left hemispheral average thickness | -0.000436 | 0.000224 | -1.94 | 5.20E-02 | 0.3019 | -0.0611 | 1200 | 2865 |
| Right hemispheral average thickness | -0.000476 | 0.000225 | -2.12 | 3.40E-02 | 0.2485 | -0.0668 | 1200 | 2865 |
|  |  |  |  |  |  |  |  |  |
| **Cortical thickness** |  |  |  |  |  |  |  |  |
| Left banks of the superior temporal sulcus | -0.000076 | 0.000423 | -0.18 | 8.57E-01 | 0.9395 | -0.0058 | 1139 | 2708 |
| Left caudal anterior cingulate cortex | 0.000029 | 0.000592 | 0.05 | 9.61E-01 | 0.9707 | 0.0016 | 1198 | 2852 |
| Left caudal middle frontal gyrus | -0.000547 | 0.000358 | -1.53 | 1.27E-01 | 0.4051 | -0.0484 | 1196 | 2840 |
| Left cuneus | 0.000344 | 0.000326 | 1.06 | 2.90E-01 | 0.5839 | 0.0337 | 1183 | 2797 |
| Left entorhinal cortex | 0.000219 | 0.000893 | 0.25 | 8.06E-01 | 0.9309 | 0.0081 | 1164 | 2725 |
| Left fusiform gyrus | 0.000123 | 0.000352 | 0.35 | 7.26E-01 | 0.8981 | 0.0111 | 1195 | 2849 |
| Left inferior parietal cortex | 0.000037 | 0.000314 | 0.12 | 9.06E-01 | 0.9646 | 0.0038 | 1180 | 2831 |
| Left inferior temporal gyrus | -0.000490 | 0.000396 | -1.24 | 2.16E-01 | 0.5553 | -0.0394 | 1165 | 2823 |
| Left isthmus cingulate cortex | -0.000719 | 0.000477 | -1.51 | 1.32E-01 | 0.4051 | -0.0477 | 1195 | 2852 |
| Left lateral occipital cortex | -0.000144 | 0.000291 | -0.50 | 6.20E-01 | 0.8504 | -0.0158 | 1186 | 2853 |
| Left lateral orbitofrontal cortex | -0.001205 | 0.000393 | -3.07 | 2.18E-03 | 0.0428 | -0.0970 | 1188 | 2851 |
| Left lingual gyrus | -0.000320 | 0.000298 | -1.07 | 2.83E-01 | 0.5839 | -0.0339 | 1191 | 2837 |
| Left medial orbitofrontal cortex | -0.000068 | 0.000392 | -0.17 | 8.62E-01 | 0.9395 | -0.0054 | 1182 | 2818 |
| Left middle temporal gyrus | -0.000241 | 0.000401 | -0.60 | 5.48E-01 | 0.8270 | -0.0193 | 1149 | 2748 |
| Left parahippcampal gyrus | 0.000076 | 0.000766 | 0.10 | 9.21E-01 | 0.9646 | 0.0032 | 1190 | 2850 |
| Left paracentral lobule | -0.000210 | 0.000362 | -0.58 | 5.61E-01 | 0.8393 | -0.0183 | 1195 | 2857 |
| Left pars opercularis | -0.000141 | 0.000353 | -0.40 | 6.89E-01 | 0.8896 | -0.0126 | 1194 | 2845 |
| Left pars orbitalis | -0.001741 | 0.000538 | -3.24 | 1.22E-03 | 0.0274 | -0.1023 | 1194 | 2854 |
| Left pars triangularis | -0.001252 | 0.000386 | -3.24 | 1.19E-03 | 0.0274 | -0.1024 | 1193 | 2848 |
| Left pericalcarine | -0.000577 | 0.000324 | -1.78 | 7.50E-02 | 0.3019 | -0.0566 | 1180 | 2803 |
| Left precentral gyrus | -0.000823 | 0.000288 | -2.86 | 4.25E-03 | 0.0667 | -0.0907 | 1182 | 2829 |
| Left posterior cingulate cortex | -0.000183 | 0.000383 | -0.48 | 6.33E-01 | 0.8572 | -0.0151 | 1196 | 2857 |
| Left precentral gyrus | -0.000622 | 0.000336 | -1.85 | 6.40E-02 | 0.3019 | -0.0585 | 1192 | 2837 |
| Left precuneus | -0.000537 | 0.000310 | -1.73 | 8.34E-02 | 0.3270 | -0.0547 | 1189 | 2851 |
| Left rostral anterior cingulate cortex | 0.000487 | 0.000569 | 0.86 | 3.93E-01 | 0.6909 | 0.0272 | 1189 | 2835 |
| Left rostral middle frontal gyrus | -0.001460 | 0.000330 | -4.43 | 9.60E-06 | 0.0015 | -0.1399 | 1197 | 2848 |
| Left superior frontal gyrus | -0.000614 | 0.000344 | -1.79 | 7.43E-02 | 0.3019 | -0.0565 | 1194 | 2851 |
| Left superior parietal cortex | -0.000286 | 0.000283 | -1.01 | 3.13E-01 | 0.6143 | -0.0320 | 1187 | 2831 |
| Left superior temporal gyrus | -0.000121 | 0.000389 | -0.31 | 7.56E-01 | 0.8992 | -0.0101 | 1138 | 2684 |
| Left supramarginal gyrus | -0.000029 | 0.000328 | -0.09 | 9.29E-01 | 0.9646 | -0.0029 | 1173 | 2767 |
| Left frontal pole | -0.001097 | 0.000741 | -1.48 | 1.39E-01 | 0.4124 | -0.0466 | 1199 | 2863 |
| Left temporal pole | -0.000597 | 0.000905 | -0.66 | 5.10E-01 | 0.8000 | -0.0209 | 1187 | 2851 |
| Left transverse temporal gyrus | 0.000413 | 0.000534 | 0.77 | 4.39E-01 | 0.7382 | 0.0243 | 1195 | 2853 |
| Left insula | 0.000211 | 0.000392 | 0.54 | 5.90E-01 | 0.8502 | 0.0171 | 1188 | 2811 |
| Right banks of the superior temporal sulcus | -0.000084 | 0.000426 | -0.20 | 8.44E-01 | 0.9395 | -0.0064 | 1178 | 2796 |
| Right caudal anterior cingulate cortex | -0.000337 | 0.000551 | -0.61 | 5.41E-01 | 0.8248 | -0.0192 | 1198 | 2852 |
| Right caudal middle frontal gyrus | -0.000471 | 0.000349 | -1.35 | 1.78E-01 | 0.4816 | -0.0427 | 1194 | 2845 |
| Right cuneus | 0.000338 | 0.000332 | 1.02 | 3.10E-01 | 0.6143 | 0.0324 | 1179 | 2809 |
| Right entorhinal cortex | 0.000327 | 0.000957 | 0.34 | 7.33E-01 | 0.8989 | 0.0110 | 1162 | 2686 |
| Right fusiform gyrus | -0.000265 | 0.000354 | -0.75 | 4.54E-01 | 0.7467 | -0.0237 | 1193 | 2849 |
| Right inferior parietal cortex | -0.000477 | 0.000311 | -1.53 | 1.25E-01 | 0.4051 | -0.0485 | 1187 | 2826 |
| Right inferior temporal gyrus | -0.000797 | 0.000410 | -1.94 | 5.19E-02 | 0.3019 | -0.0615 | 1175 | 2838 |
| Right isthmus cingulate cortex | -0.000883 | 0.000463 | -1.91 | 5.70E-02 | 0.3019 | -0.0603 | 1196 | 2854 |
| Right lateral occipital cortex | -0.000503 | 0.000303 | -1.66 | 9.80E-02 | 0.3497 | -0.0524 | 1193 | 2848 |
| Right lateral orbitofrontal cortex | -0.000579 | 0.000391 | -1.48 | 1.39E-01 | 0.4124 | -0.0467 | 1195 | 2858 |
| Right lingual gyrus | -0.000231 | 0.000300 | -0.77 | 4.42E-01 | 0.7382 | -0.0244 | 1182 | 2834 |
| Right medial orbitofrontal cortex | -0.000633 | 0.000407 | -1.56 | 1.20E-01 | 0.4051 | -0.0494 | 1183 | 2831 |
| Right middle temporal gyrus | -0.000347 | 0.000396 | -0.88 | 3.80E-01 | 0.6909 | -0.0279 | 1184 | 2815 |
| Right parahippcampal gyrus | 0.000266 | 0.000655 | 0.41 | 6.85E-01 | 0.8896 | 0.0130 | 1192 | 2850 |
| Right paracentral lobule | -0.000246 | 0.000366 | -0.67 | 5.02E-01 | 0.7963 | -0.0211 | 1196 | 2857 |
| Right pars opercularis | -0.000122 | 0.000368 | -0.33 | 7.39E-01 | 0.8992 | -0.0104 | 1189 | 2835 |
| Right pars orbitalis | -0.000292 | 0.000527 | -0.55 | 5.80E-01 | 0.8502 | -0.0174 | 1198 | 2848 |
| Right pars triangularis | -0.000420 | 0.000384 | -1.09 | 2.75E-01 | 0.5839 | -0.0345 | 1187 | 2838 |
| Right pericalcarine | -0.000353 | 0.000330 | -1.07 | 2.80E-01 | 0.5839 | -0.0341 | 1177 | 2803 |
| Right precentral gyrus | -0.000855 | 0.000294 | -2.91 | 3.70E-03 | 0.0645 | -0.0921 | 1187 | 2842 |
| Right posterior cingulate cortex | 0.000074 | 0.000368 | 0.20 | 8.41E-01 | 0.9395 | 0.0063 | 1196 | 2859 |
| Right precentral gyrus | -0.000654 | 0.000343 | -1.90 | 5.70E-02 | 0.3019 | -0.0601 | 1188 | 2844 |
| Right precuneus | -0.000290 | 0.000306 | -0.95 | 3.44E-01 | 0.6668 | -0.0300 | 1195 | 2848 |
| Right rostral anterior cingulate cortex | 0.000407 | 0.000557 | 0.73 | 4.66E-01 | 0.7534 | 0.0231 | 1194 | 2836 |
| Right rostral middle frontal gyrus | -0.000849 | 0.000327 | -2.59 | 9.52E-03 | 0.1150 | -0.0818 | 1192 | 2849 |
| Right superior frontal gyrus | -0.000919 | 0.000343 | -2.68 | 7.36E-03 | 0.1050 | -0.0846 | 1189 | 2859 |
| Right superior parietal cortex | -0.000485 | 0.000282 | -1.72 | 8.61E-02 | 0.3270 | -0.0544 | 1195 | 2842 |
| Right superior temporal gyrus | -0.000682 | 0.000383 | -1.78 | 7.50E-02 | 0.3019 | -0.0572 | 1161 | 2745 |
| Right supramarginal gyrus | -0.000602 | 0.000330 | -1.83 | 6.80E-02 | 0.3019 | -0.0584 | 1178 | 2780 |
| Right frontal pole | -0.000908 | 0.000726 | -1.25 | 2.11E-01 | 0.5522 | -0.0394 | 1200 | 2861 |
| Right temporal pole | 0.000063 | 0.000931 | 0.07 | 9.46E-01 | 0.9646 | 0.0022 | 1191 | 2850 |
| Right transverse temporal gyrus | 0.000698 | 0.000553 | 1.26 | 2.07E-01 | 0.5497 | 0.0398 | 1190 | 2849 |
| Right insula | 0.000162 | 0.000413 | 0.39 | 6.95E-01 | 0.8896 | 0.0124 | 1182 | 2777 |
|  |  |  |  |  |  |  |  |  |
| **Subcortical volumes** |  |  |  |  |  |  |  |  |
| Left lateral ventricle | 26.582994 | 10.777970 | 2.47 | 1.37E-02 | 0.1303 | 0.0800 | 1138 | 2710 |
| Right lateral ventricle | 13.808605 | 10.081675 | 1.37 | 1.71E-01 | 0.4706 | 0.0444 | 1130 | 2706 |
| Left thalamus | -6.417322 | 1.723616 | -3.72 | 2.00E-04 | 0.0152 | -0.1208 | 1138 | 2691 |
| Right thalamus | -1.665835 | 1.473281 | -1.13 | 2.58E-01 | 0.5801 | -0.0368 | 1134 | 2680 |
| Left caudate | 0.757748 | 1.017775 | 0.74 | 4.57E-01 | 0.7467 | 0.0240 | 1151 | 2684 |
| Right caudate | 0.928952 | 1.022735 | 0.91 | 3.64E-01 | 0.6881 | 0.0296 | 1128 | 2674 |
| Left putamen | 0.719696 | 1.401686 | 0.51 | 6.08E-01 | 0.8504 | 0.0168 | 1089 | 2613 |
| Right putamen | -0.561811 | 1.283516 | -0.44 | 6.62E-01 | 0.8878 | -0.0144 | 1107 | 2649 |
| Left pallidum | 0.634710 | 0.524685 | 1.21 | 2.26E-01 | 0.5644 | 0.0403 | 1080 | 2563 |
| Right pallidum | 0.069107 | 0.455144 | 0.15 | 8.79E-01 | 0.9521 | 0.0049 | 1129 | 2678 |
| Left hippocampus | -1.990372 | 0.922302 | -2.16 | 3.10E-02 | 0.2485 | -0.0703 | 1127 | 2683 |
| Right hippocamups | -3.011013 | 0.920460 | -3.27 | 1.08E-03 | 0.0274 | -0.1059 | 1136 | 2709 |
| Left amygdala | -0.247893 | 0.432600 | -0.57 | 5.67E-01 | 0.8393 | -0.0185 | 1127 | 2694 |
| Right amygdala | -0.544836 | 0.466889 | -1.17 | 2.43E-01 | 0.5801 | -0.0380 | 1129 | 2702 |
| Left nucleus accumbens | 0.271945 | 0.236178 | 1.15 | 2.50E-01 | 0.5801 | 0.0376 | 1110 | 2660 |
| Right nucleus accumbens | -0.065393 | 0.214229 | -0.31 | 7.60E-01 | 0.8992 | -0.0101 | 1116 | 2657 |
|  |  |  |  |  |  |  |  |  |
| **Cortical surface area** |  |  |  |  |  |  |  |  |
| Left banks of the superior temporal sulcus | 0.086690 | 0.377183 | 0.23 | 8.18E-01 | 0.9309 | 0.0077 | 1052 | 2527 |
| Left caudal anterior cingulate cortex | -0.477913 | 0.340597 | -1.40 | 1.61E-01 | 0.4671 | -0.0456 | 1120 | 2687 |
| Left caudal middle frontal gyrus | 0.060518 | 0.851257 | 0.07 | 9.43E-01 | 0.9646 | 0.0023 | 1123 | 2678 |
| Left cuneus | -0.315860 | 0.500080 | -0.63 | 5.28E-01 | 0.8202 | -0.0206 | 1115 | 2644 |
| Left entorhinal cortex | -0.098505 | 0.194651 | -0.51 | 6.13E-01 | 0.8504 | -0.0172 | 1046 | 2513 |
| Left fusiform gyrus | -1.794763 | 0.857339 | -2.09 | 3.64E-02 | 0.2485 | -0.0686 | 1097 | 2653 |
| Left inferior parietal cortex | -2.432435 | 1.352600 | -1.80 | 7.22E-02 | 0.3019 | -0.0588 | 1115 | 2663 |
| Left inferior temporal gyrus | -2.111720 | 1.005172 | -2.10 | 3.57E-02 | 0.2485 | -0.0687 | 1099 | 2673 |
| Left isthmus cingulate cortex | 0.110493 | 0.364971 | 0.30 | 7.62E-01 | 0.8992 | 0.0097 | 1134 | 2700 |
| Left lateral occipital cortex | -0.490866 | 1.291552 | -0.38 | 7.04E-01 | 0.8913 | -0.0123 | 1127 | 2701 |
| Left lateral orbitofrontal cortex | 0.163256 | 0.605430 | 0.27 | 7.90E-01 | 0.9187 | 0.0087 | 1139 | 2708 |
| Left lingual gyrus | 0.040701 | 0.915419 | 0.04 | 9.65E-01 | 0.9707 | 0.0013 | 1128 | 2692 |
| Left medial orbitofrontal cortex | -0.807752 | 0.521368 | -1.55 | 1.21E-01 | 0.4051 | -0.0508 | 1107 | 2650 |
| Left middle temporal gyrus | -0.304498 | 0.850228 | -0.36 | 7.20E-01 | 0.8981 | -0.0120 | 1064 | 2578 |
| Left parahippcampal gyrus | -0.568363 | 0.221683 | -2.56 | 1.04E-02 | 0.1166 | -0.0835 | 1114 | 2682 |
| Left paracentral lobule | -0.494275 | 0.436965 | -1.13 | 2.58E-01 | 0.5801 | -0.0370 | 1099 | 2658 |
| Left pars opercularis | -1.452298 | 0.591449 | -2.46 | 1.41E-02 | 0.1303 | -0.0801 | 1120 | 2687 |
| Left pars orbitalis | -0.308067 | 0.184222 | -1.67 | 9.46E-02 | 0.3453 | -0.0541 | 1139 | 2703 |
| Left pars triangularis | -0.517221 | 0.458528 | -1.13 | 2.59E-01 | 0.5801 | -0.0367 | 1134 | 2692 |
| Left pericalcarine | 0.584253 | 0.552202 | 1.06 | 2.90E-01 | 0.5839 | 0.0346 | 1124 | 2654 |
| Left precentral gyrus | -0.511267 | 0.963485 | -0.53 | 5.96E-01 | 0.8502 | -0.0174 | 1094 | 2658 |
| Left posterior cingulate cortex | -0.870709 | 0.406173 | -2.14 | 3.21E-02 | 0.2485 | -0.0694 | 1135 | 2702 |
| Left precentral gyrus | -0.324739 | 1.098245 | -0.30 | 7.67E-01 | 0.8992 | -0.0098 | 1117 | 2674 |
| Left precuneus | -1.713425 | 0.955313 | -1.79 | 7.30E-02 | 0.3019 | -0.0581 | 1130 | 2697 |
| Left rostral anterior cingulate cortex | -0.291316 | 0.342666 | -0.85 | 3.95E-01 | 0.6909 | -0.0279 | 1097 | 2655 |
| Left rostral middle frontal gyrus | 1.298706 | 1.498549 | 0.87 | 3.86E-01 | 0.6909 | 0.0282 | 1135 | 2694 |
| Left superior frontal gyrus | -0.570178 | 1.615819 | -0.35 | 7.24E-01 | 0.8981 | -0.0114 | 1106 | 2672 |
| Left superior parietal cortex | -0.557024 | 1.375243 | -0.41 | 6.85E-01 | 0.8896 | -0.0134 | 1111 | 2668 |
| Left superior temporal gyrus | -0.483315 | 0.928306 | -0.52 | 6.03E-01 | 0.8504 | -0.0175 | 1064 | 2514 |
| Left supramarginal gyrus | -1.304253 | 1.199018 | -1.09 | 2.77E-01 | 0.5839 | -0.0362 | 1082 | 2572 |
| Left frontal pole | -0.074493 | 0.083231 | -0.90 | 3.71E-01 | 0.6909 | -0.0291 | 1141 | 2711 |
| Left temporal pole | -0.014543 | 0.143113 | -0.10 | 9.19E-01 | 0.9646 | -0.0032 | 1134 | 2698 |
| Left transverse temporal gyrus | -0.261376 | 0.173177 | -1.51 | 1.31E-01 | 0.4051 | -0.0489 | 1141 | 2708 |
| Left insula | -0.429634 | 0.518465 | -0.83 | 4.07E-01 | 0.6952 | -0.0271 | 1127 | 2660 |
| Right banks of the superior temporal sulcus | -0.277702 | 0.318031 | -0.87 | 3.83E-01 | 0.6909 | -0.0286 | 1106 | 2632 |
| Right caudal anterior cingulate cortex | -0.142429 | 0.365735 | -0.39 | 6.97E-01 | 0.8896 | -0.0127 | 1119 | 2693 |
| Right caudal middle frontal gyrus | 0.981786 | 0.847315 | 1.16 | 2.47E-01 | 0.5801 | 0.0378 | 1123 | 2685 |
| Right cuneus | -0.519563 | 0.470468 | -1.10 | 2.70E-01 | 0.5839 | -0.0360 | 1115 | 2654 |
| Right entorhinal cortex | -0.221413 | 0.197496 | -1.12 | 2.62E-01 | 0.5801 | -0.0380 | 1036 | 2477 |
| Right fusiform gyrus | -2.188601 | 0.831348 | -2.63 | 8.51E-03 | 0.1113 | -0.0862 | 1089 | 2665 |
| Right inferior parietal cortex | -2.705505 | 1.474336 | -1.84 | 6.66E-02 | 0.3019 | -0.0602 | 1118 | 2652 |
| Right inferior temporal gyrus | -1.298971 | 0.938510 | -1.38 | 1.66E-01 | 0.4706 | -0.0450 | 1115 | 2684 |
| Right isthmus cingulate cortex | -0.167426 | 0.340441 | -0.49 | 6.23E-01 | 0.8504 | -0.0159 | 1137 | 2706 |
| Right lateral occipital cortex | -1.577131 | 1.286665 | -1.23 | 2.20E-01 | 0.5581 | -0.0399 | 1131 | 2701 |
| Right lateral orbitofrontal cortex | -0.348140 | 0.653862 | -0.53 | 5.94E-01 | 0.8502 | -0.0172 | 1140 | 2709 |
| Right lingual gyrus | -0.770388 | 0.909361 | -0.85 | 3.97E-01 | 0.6909 | -0.0276 | 1127 | 2688 |
| Right medial orbitofrontal cortex | -1.133968 | 0.454760 | -2.49 | 1.27E-02 | 0.1303 | -0.0812 | 1116 | 2679 |
| Right middle temporal gyrus | -0.979477 | 0.853439 | -1.15 | 2.51E-01 | 0.5801 | -0.0377 | 1114 | 2644 |
| Right parahippcampal gyrus | -0.481029 | 0.219634 | -2.19 | 2.86E-02 | 0.2485 | -0.0714 | 1116 | 2685 |
| Right paracentral lobule | 0.043083 | 0.491954 | 0.09 | 9.30E-01 | 0.9646 | 0.0029 | 1117 | 2688 |
| Right pars opercularis | 0.168423 | 0.543576 | 0.31 | 7.57E-01 | 0.8992 | 0.0101 | 1117 | 2671 |
| Right pars orbitalis | -0.015468 | 0.219381 | -0.07 | 9.44E-01 | 0.9646 | -0.0023 | 1139 | 2696 |
| Right pars triangularis | -0.097218 | 0.537673 | -0.18 | 8.57E-01 | 0.9395 | -0.0059 | 1124 | 2677 |
| Right pericalcarine | -0.354559 | 0.570142 | -0.62 | 5.34E-01 | 0.8220 | -0.0203 | 1128 | 2653 |
| Right precentral gyrus | -1.972611 | 0.955619 | -2.06 | 3.91E-02 | 0.2556 | -0.0672 | 1115 | 2679 |
| Right posterior cingulate cortex | -0.166935 | 0.393177 | -0.42 | 6.71E-01 | 0.8896 | -0.0136 | 1134 | 2707 |
| Right precentral gyrus | -0.784691 | 1.136465 | -0.69 | 4.90E-01 | 0.7849 | -0.0225 | 1111 | 2678 |
| Right precuneus | -3.238882 | 0.999338 | -3.24 | 1.20E-03 | 0.0274 | -0.1051 | 1135 | 2698 |
| Right rostral anterior cingulate cortex | -0.434264 | 0.316512 | -1.37 | 1.70E-01 | 0.4706 | -0.0447 | 1115 | 2679 |
| Right rostral middle frontal gyrus | 0.360131 | 1.522415 | 0.24 | 8.13E-01 | 0.9309 | 0.0078 | 1135 | 2698 |
| Right superior frontal gyrus | 0.012355 | 1.625343 | 0.01 | 9.94E-01 | 0.9939 | 0.0003 | 1110 | 2686 |
| Right superior parietal cortex | -0.113124 | 1.331266 | -0.08 | 9.32E-01 | 0.9646 | -0.0026 | 1125 | 2679 |
| Right superior temporal gyrus | -1.406035 | 0.822604 | -1.71 | 8.75E-02 | 0.3270 | -0.0568 | 1091 | 2572 |
| Right supramarginal gyrus | -0.936538 | 1.113662 | -0.84 | 4.00E-01 | 0.6909 | -0.0277 | 1104 | 2604 |
| Right frontal pole | -0.196833 | 0.103688 | -1.90 | 5.77E-02 | 0.3019 | -0.0615 | 1140 | 2710 |
| Right temporal pole | -0.141932 | 0.153357 | -0.93 | 3.55E-01 | 0.6792 | -0.0303 | 1106 | 2686 |
| Right transverse temporal gyrus | -0.269491 | 0.133248 | -2.02 | 4.32E-02 | 0.2713 | -0.0654 | 1141 | 2712 |
| Right insula | -0.124389 | 0.589183 | -0.21 | 8.33E-01 | 0.9395 | -0.0069 | 1121 | 2626 |

| **Label** | **Estimate** | **StdError** | **T** | **p** | **FDR adjusted p** | **Cohen´s d** | **N** |
| --- | --- | --- | --- | --- | --- | --- | --- |
|  |  |  |  |  |  |  |  |
| **Global measures** |  |  |  |  |  |  |  |
| Total Intracranial Volume | -1898.000000 | 1227.000000 | -1.55 | 1.23E-01 | 0.3651 | -0.1405 | 512 |
| Total left hemispheral surface area | -19.100000 | 66.300000 | -0.29 | 7.73E-01 | 0.8547 | -0.0257 | 535 |
| Total right hemispheral surface area | 9.770000 | 66.760000 | 0.15 | 8.84E-01 | 0.9249 | 0.0133 | 535 |
| Left hemispheral average thickness | -0.002170 | 0.000837 | -2.59 | 9.80E-03 | 0.1427 | -0.2296 | 534 |
| Right hemispheral average thickness | -0.001564 | 0.000847 | -1.85 | 6.56E-02 | 0.2784 | -0.1642 | 533 |
|  |  |  |  |  |  |  |  |
| **Cortical thickness** |  |  |  |  |  |  |  |
| Left banks of the superior temporal sulcus | -0.001960 | 0.001630 | -1.20 | 2.29E-01 | 0.4992 | -0.1092 | 508 |
| Left caudal anterior cingulate cortex | -0.004870 | 0.002190 | -2.22 | 2.66E-02 | 0.1547 | -0.1970 | 533 |
| Left caudal middle frontal gyrus | -0.001320 | 0.001340 | -0.98 | 3.27E-01 | 0.5908 | -0.0870 | 533 |
| Left cuneus | -0.001380 | 0.001320 | -1.04 | 2.98E-01 | 0.5547 | -0.0924 | 532 |
| Left entorhinal cortex | 0.001088 | 0.003387 | 0.32 | 7.48E-01 | 0.8391 | 0.0289 | 516 |
| Left fusiform gyrus | -0.002829 | 0.001233 | -2.29 | 2.22E-02 | 0.1457 | -0.2036 | 531 |
| Left inferior parietal cortex | -0.002970 | 0.001200 | -2.48 | 1.35E-02 | 0.1427 | -0.2205 | 531 |
| Left inferior temporal gyrus | -0.001290 | 0.001530 | -0.84 | 4.00E-01 | 0.6403 | -0.0749 | 528 |
| Left isthmus cingulate cortex | -0.000170 | 0.001870 | -0.09 | 9.27E-01 | 0.9578 | -0.0080 | 529 |
| Left lateral occipital cortex | -0.002580 | 0.001050 | -2.47 | 1.38E-02 | 0.1427 | -0.2192 | 533 |
| Left lateral orbitofrontal cortex | -0.002700 | 0.001490 | -1.81 | 7.13E-02 | 0.2870 | -0.1608 | 532 |
| Left lingual gyrus | -0.000658 | 0.001193 | -0.55 | 5.82E-01 | 0.7571 | -0.0490 | 529 |
| Left medial orbitofrontal cortex | -0.003440 | 0.001470 | -2.34 | 1.98E-02 | 0.1427 | -0.2089 | 527 |
| Left middle temporal gyrus | -0.003800 | 0.001510 | -2.51 | 1.24E-02 | 0.1427 | -0.2270 | 514 |
| Left parahippcampal gyrus | 0.000524 | 0.002612 | 0.20 | 8.41E-01 | 0.9045 | 0.0178 | 531 |
| Left paracentral lobule | -0.001200 | 0.001320 | -0.90 | 3.67E-01 | 0.6158 | -0.0799 | 532 |
| Left pars opercularis | -0.000873 | 0.001230 | -0.71 | 4.78E-01 | 0.7046 | -0.0632 | 530 |
| Left pars orbitalis | -0.002990 | 0.001960 | -1.53 | 1.26E-01 | 0.3663 | -0.1362 | 530 |
| Left pars triangularis | -0.001150 | 0.001320 | -0.87 | 3.82E-01 | 0.6315 | -0.0774 | 530 |
| Left pericalcarine | 0.001370 | 0.001250 | 1.10 | 2.74E-01 | 0.5495 | 0.0980 | 529 |
| Left precentral gyrus | -0.001010 | 0.001100 | -0.91 | 3.61E-01 | 0.6158 | -0.0811 | 528 |
| Left posterior cingulate cortex | -0.000014 | 0.001463 | -0.01 | 9.93E-01 | 0.9927 | -0.0009 | 533 |
| Left precentral gyrus | -0.002920 | 0.001150 | -2.53 | 1.16E-02 | 0.1427 | -0.2258 | 527 |
| Left precuneus | -0.001500 | 0.001160 | -1.29 | 1.97E-01 | 0.4760 | -0.1145 | 533 |
| Left rostral anterior cingulate cortex | -0.004440 | 0.002080 | -2.14 | 3.32E-02 | 0.1782 | -0.1906 | 529 |
| Left rostral middle frontal gyrus | -0.003112 | 0.001220 | -2.55 | 1.10E-02 | 0.1427 | -0.2265 | 532 |
| Left superior frontal gyrus | -0.003050 | 0.001340 | -2.28 | 2.29E-02 | 0.1457 | -0.2027 | 531 |
| Left superior parietal cortex | -0.001850 | 0.001070 | -1.73 | 8.40E-02 | 0.3051 | -0.1541 | 529 |
| Left superior temporal gyrus | -0.003410 | 0.001400 | -2.43 | 1.56E-02 | 0.1427 | -0.2207 | 510 |
| Left supramarginal gyrus | -0.003519 | 0.001197 | -2.94 | 3.44E-03 | 0.1039 | -0.2627 | 526 |
| Left frontal pole | -0.004230 | 0.003040 | -1.39 | 1.65E-01 | 0.4466 | -0.1232 | 534 |
| Left temporal pole | -0.003830 | 0.003520 | -1.09 | 2.76E-01 | 0.5495 | -0.0968 | 532 |
| Left transverse temporal gyrus | -0.002160 | 0.001830 | -1.18 | 2.38E-01 | 0.4992 | -0.1049 | 531 |
| Left insula | -0.001940 | 0.001250 | -1.54 | 1.23E-01 | 0.3651 | -0.1373 | 528 |
| Right banks of the superior temporal sulcus | 0.001520 | 0.001680 | 0.91 | 3.66E-01 | 0.6158 | 0.0821 | 517 |
| Right caudal anterior cingulate cortex | -0.000452 | 0.002250 | -0.20 | 8.41E-01 | 0.9045 | -0.0177 | 534 |
| Right caudal middle frontal gyrus | -0.000708 | 0.001341 | -0.53 | 5.98E-01 | 0.7571 | -0.0471 | 532 |
| Right cuneus | -0.001830 | 0.001280 | -1.43 | 1.53E-01 | 0.4285 | -0.1271 | 531 |
| Right entorhinal cortex | -0.003140 | 0.003620 | -0.87 | 3.86E-01 | 0.6315 | -0.0793 | 506 |
| Right fusiform gyrus | -0.000636 | 0.001292 | -0.49 | 6.23E-01 | 0.7740 | -0.0437 | 529 |
| Right inferior parietal cortex | -0.001360 | 0.001150 | -1.18 | 2.37E-01 | 0.4992 | -0.1054 | 526 |
| Right inferior temporal gyrus | 0.000022 | 0.001583 | 0.01 | 9.89E-01 | 0.9927 | 0.0009 | 531 |
| Right isthmus cingulate cortex | -0.000792 | 0.001733 | -0.46 | 6.48E-01 | 0.7785 | -0.0409 | 531 |
| Right lateral occipital cortex | -0.000877 | 0.001108 | -0.79 | 4.29E-01 | 0.6737 | -0.0703 | 530 |
| Right lateral orbitofrontal cortex | -0.003150 | 0.001480 | -2.13 | 3.41E-02 | 0.1782 | -0.1894 | 531 |
| Right lingual gyrus | 0.000816 | 0.001113 | 0.73 | 4.64E-01 | 0.7003 | 0.0650 | 529 |
| Right medial orbitofrontal cortex | -0.003660 | 0.001550 | -2.36 | 1.88E-02 | 0.1427 | -0.2102 | 529 |
| Right middle temporal gyrus | -0.002130 | 0.001560 | -1.37 | 1.72E-01 | 0.4488 | -0.1227 | 524 |
| Right parahippcampal gyrus | -0.002160 | 0.002310 | -0.94 | 3.50E-01 | 0.6158 | -0.0833 | 534 |
| Right paracentral lobule | -0.002370 | 0.001370 | -1.73 | 8.48E-02 | 0.3051 | -0.1537 | 532 |
| Right pars opercularis | 0.000728 | 0.001377 | 0.53 | 5.97E-01 | 0.7571 | 0.0472 | 530 |
| Right pars orbitalis | -0.003526 | 0.002009 | -1.76 | 7.99E-02 | 0.3051 | -0.1569 | 528 |
| Right pars triangularis | -0.002250 | 0.001370 | -1.65 | 9.97E-02 | 0.3330 | -0.1466 | 532 |
| Right pericalcarine | 0.000766 | 0.001321 | 0.58 | 5.62E-01 | 0.7544 | 0.0518 | 527 |
| Right precentral gyrus | -0.000596 | 0.001119 | -0.53 | 5.94E-01 | 0.7571 | -0.0472 | 529 |
| Right posterior cingulate cortex | -0.001760 | 0.001390 | -1.27 | 2.05E-01 | 0.4811 | -0.1127 | 533 |
| Right precentral gyrus | -0.002310 | 0.001240 | -1.87 | 6.26E-02 | 0.2784 | -0.1666 | 529 |
| Right precuneus | -0.000673 | 0.001144 | -0.59 | 5.56E-01 | 0.7544 | -0.0525 | 531 |
| Right rostral anterior cingulate cortex | 0.001030 | 0.002100 | 0.49 | 6.26E-01 | 0.7740 | 0.0436 | 530 |
| Right rostral middle frontal gyrus | -0.003600 | 0.001190 | -3.02 | 2.65E-03 | 0.1039 | -0.2682 | 532 |
| Right superior frontal gyrus | -0.003170 | 0.001310 | -2.42 | 1.59E-02 | 0.1427 | -0.2152 | 531 |
| Right superior parietal cortex | -0.000827 | 0.001084 | -0.76 | 4.46E-01 | 0.6922 | -0.0676 | 531 |
| Right superior temporal gyrus | -0.002545 | 0.001388 | -1.83 | 6.74E-02 | 0.2784 | -0.1652 | 516 |
| Right supramarginal gyrus | -0.000385 | 0.001288 | -0.30 | 7.65E-01 | 0.8519 | -0.0269 | 522 |
| Right frontal pole | -0.006510 | 0.002790 | -2.34 | 2.00E-02 | 0.1427 | -0.2078 | 532 |
| Right temporal pole | -0.004700 | 0.003810 | -1.23 | 2.18E-01 | 0.4960 | -0.1093 | 532 |
| Right transverse temporal gyrus | -0.002441 | 0.001896 | -1.29 | 1.99E-01 | 0.4760 | -0.1145 | 533 |
| Right insula | -0.000367 | 0.001389 | -0.26 | 7.92E-01 | 0.8693 | -0.0232 | 528 |
|  |  |  |  |  |  |  |  |
| **Subcortical volumes** |  |  |  |  |  |  |  |
| Left lateral ventricle | 32.477217 | 27.026343 | 1.20 | 2.30E-01 | 0.4992 | 0.1091 | 509 |
| Right lateral ventricle | 21.798390 | 26.609830 | 0.82 | 4.13E-01 | 0.6550 | 0.0744 | 511 |
| Left thalamus | 9.824526 | 6.035082 | 1.63 | 1.04E-01 | 0.3339 | 0.1486 | 506 |
| Right thalamus | -0.650169 | 5.365774 | -0.12 | 9.04E-01 | 0.9399 | -0.0109 | 508 |
| Left caudate | 3.099923 | 4.097102 | 0.76 | 4.50E-01 | 0.6922 | 0.0693 | 506 |
| Right caudate | 2.154988 | 4.183742 | 0.52 | 6.07E-01 | 0.7620 | 0.0473 | 508 |
| Left putamen | -0.223755 | 6.186160 | -0.04 | 9.71E-01 | 0.9927 | -0.0037 | 490 |
| Right putamen | 6.959352 | 5.423655 | 1.28 | 2.00E-01 | 0.4760 | 0.1172 | 502 |
| Left pallidum | -5.024890 | 1.993630 | -2.52 | 1.21E-02 | 0.1427 | -0.2345 | 487 |
| Right pallidum | -1.062324 | 1.775800 | -0.60 | 5.50E-01 | 0.7544 | -0.0547 | 507 |
| Left hippocampus | 1.560542 | 3.432422 | 0.45 | 6.50E-01 | 0.7785 | 0.0412 | 503 |
| Right hippocamups | 4.502391 | 3.366997 | 1.34 | 1.82E-01 | 0.4531 | 0.1218 | 509 |
| Left amygdala | 4.503279 | 1.649172 | 2.73 | 6.55E-03 | 0.1315 | 0.2487 | 507 |
| Right amygdala | 7.337543 | 1.722407 | 4.26 | 2.46E-05 | 0.0039 | 0.3897 | 503 |
| Left nucleus accumbens | -0.364708 | 0.985368 | -0.37 | 7.11E-01 | 0.8274 | -0.0339 | 501 |
| Right nucleus accumbens | 0.307618 | 0.855603 | 0.36 | 7.19E-01 | 0.8301 | 0.0328 | 506 |
|  |  |  |  |  |  |  |  |
| **Cortical surface area** |  |  |  |  |  |  |  |
| Left banks of the superior temporal sulcus | 0.352037 | 1.560555 | 0.23 | 8.22E-01 | 0.8958 | 0.0215 | 482 |
| Left caudal anterior cingulate cortex | -1.298519 | 1.245186 | -1.04 | 2.98E-01 | 0.5547 | -0.0950 | 504 |
| Left caudal middle frontal gyrus | 1.027549 | 3.067353 | 0.33 | 7.38E-01 | 0.8375 | 0.0301 | 505 |
| Left cuneus | 0.322813 | 1.805661 | 0.18 | 8.58E-01 | 0.9104 | 0.0164 | 506 |
| Left entorhinal cortex | -0.262153 | 0.794257 | -0.33 | 7.42E-01 | 0.8375 | -0.0308 | 485 |
| Left fusiform gyrus | -5.238736 | 3.092096 | -1.69 | 9.09E-02 | 0.3170 | -0.1548 | 502 |
| Left inferior parietal cortex | -1.721840 | 4.611080 | -0.37 | 7.09E-01 | 0.8274 | -0.0337 | 506 |
| Left inferior temporal gyrus | 2.981320 | 4.001792 | 0.74 | 4.57E-01 | 0.6960 | 0.0679 | 500 |
| Left isthmus cingulate cortex | 3.693521 | 1.356403 | 2.72 | 6.70E-03 | 0.1315 | 0.2483 | 505 |
| Left lateral occipital cortex | -1.763698 | 4.520024 | -0.39 | 6.97E-01 | 0.8223 | -0.0355 | 507 |
| Left lateral orbitofrontal cortex | 0.373432 | 2.482029 | 0.15 | 8.80E-01 | 0.9249 | 0.0137 | 507 |
| Left lingual gyrus | -1.495285 | 3.622529 | -0.41 | 6.80E-01 | 0.8087 | -0.0374 | 505 |
| Left medial orbitofrontal cortex | -0.386864 | 2.064166 | -0.19 | 8.51E-01 | 0.9093 | -0.0174 | 503 |
| Left middle temporal gyrus | 2.193948 | 3.319691 | 0.66 | 5.09E-01 | 0.7331 | 0.0615 | 486 |
| Left parahippcampal gyrus | 1.281300 | 0.884763 | 1.45 | 1.48E-01 | 0.4231 | 0.1325 | 504 |
| Left paracentral lobule | 3.888153 | 1.652868 | 2.35 | 1.91E-02 | 0.1427 | 0.2145 | 505 |
| Left pars opercularis | 4.404032 | 2.268641 | 1.94 | 5.28E-02 | 0.2438 | 0.1771 | 505 |
| Left pars orbitalis | 0.739349 | 0.709897 | 1.04 | 2.98E-01 | 0.5547 | 0.0949 | 505 |
| Left pars triangularis | -0.052062 | 1.782780 | -0.03 | 9.77E-01 | 0.9927 | -0.0027 | 505 |
| Left pericalcarine | -1.121153 | 1.961211 | -0.57 | 5.68E-01 | 0.7555 | -0.0523 | 500 |
| Left precentral gyrus | 4.344190 | 3.508330 | 1.24 | 2.16E-01 | 0.4960 | 0.1136 | 502 |
| Left posterior cingulate cortex | 1.442803 | 1.468223 | 0.98 | 3.26E-01 | 0.5908 | 0.0895 | 505 |
| Left precentral gyrus | -0.090382 | 3.836055 | -0.02 | 9.81E-01 | 0.9927 | -0.0018 | 502 |
| Left precuneus | 6.175617 | 3.353201 | 1.84 | 6.61E-02 | 0.2784 | 0.1676 | 507 |
| Left rostral anterior cingulate cortex | 1.325555 | 1.254285 | 1.06 | 2.91E-01 | 0.5547 | 0.0968 | 505 |
| Left rostral middle frontal gyrus | 1.828485 | 5.181522 | 0.35 | 7.24E-01 | 0.8301 | 0.0319 | 506 |
| Left superior frontal gyrus | 8.898500 | 5.383700 | 1.65 | 9.90E-02 | 0.3330 | 0.1508 | 504 |
| Left superior parietal cortex | 10.254149 | 4.776851 | 2.15 | 3.23E-02 | 0.1782 | 0.1965 | 504 |
| Left superior temporal gyrus | 2.423829 | 3.362529 | 0.72 | 4.71E-01 | 0.7046 | 0.0674 | 481 |
| Left supramarginal gyrus | 5.861810 | 4.352553 | 1.35 | 1.79E-01 | 0.4531 | 0.1240 | 499 |
| Left frontal pole | 0.363319 | 0.309925 | 1.17 | 2.42E-01 | 0.4992 | 0.1066 | 507 |
| Left temporal pole | -0.255116 | 0.556673 | -0.46 | 6.47E-01 | 0.7785 | -0.0420 | 505 |
| Left transverse temporal gyrus | 1.947322 | 0.672812 | 2.89 | 3.97E-03 | 0.1039 | 0.2638 | 505 |
| Left insula | 3.398575 | 1.957055 | 1.74 | 8.31E-02 | 0.3051 | 0.1593 | 502 |
| Right banks of the superior temporal sulcus | 0.717739 | 1.323339 | 0.54 | 5.88E-01 | 0.7571 | 0.0501 | 489 |
| Right caudal anterior cingulate cortex | 2.757839 | 1.359001 | 2.03 | 4.30E-02 | 0.2177 | 0.1851 | 506 |
| Right caudal middle frontal gyrus | 4.789748 | 3.092560 | 1.55 | 1.22E-01 | 0.3651 | 0.1415 | 505 |
| Right cuneus | 1.607728 | 1.701055 | 0.95 | 3.45E-01 | 0.6156 | 0.0868 | 504 |
| Right entorhinal cortex | 1.090855 | 0.792293 | 1.38 | 1.69E-01 | 0.4488 | 0.1294 | 480 |
| Right fusiform gyrus | 1.896736 | 3.196680 | 0.59 | 5.53E-01 | 0.7544 | 0.0539 | 504 |
| Right inferior parietal cortex | 4.659344 | 5.178638 | 0.90 | 3.69E-01 | 0.6158 | 0.0825 | 501 |
| Right inferior temporal gyrus | 2.478260 | 3.544002 | 0.70 | 4.85E-01 | 0.7046 | 0.0639 | 505 |
| Right isthmus cingulate cortex | 3.739950 | 1.285942 | 2.91 | 3.80E-03 | 0.1039 | 0.2659 | 504 |
| Right lateral occipital cortex | 5.029590 | 4.616620 | 1.09 | 2.77E-01 | 0.5495 | 0.0994 | 506 |
| Right lateral orbitofrontal cortex | 4.609446 | 2.825360 | 1.63 | 1.03E-01 | 0.3339 | 0.1488 | 505 |
| Right lingual gyrus | -2.200023 | 3.456360 | -0.64 | 5.25E-01 | 0.7379 | -0.0584 | 505 |
| Right medial orbitofrontal cortex | 1.814138 | 1.749658 | 1.04 | 3.00E-01 | 0.5547 | 0.0947 | 507 |
| Right middle temporal gyrus | 5.057820 | 3.163600 | 1.60 | 1.11E-01 | 0.3471 | 0.1476 | 495 |
| Right parahippcampal gyrus | 0.843167 | 0.924267 | 0.91 | 3.62E-01 | 0.6158 | 0.0829 | 507 |
| Right paracentral lobule | 3.431783 | 1.720843 | 1.99 | 4.67E-02 | 0.2222 | 0.1815 | 506 |
| Right pars opercularis | 0.947563 | 2.023254 | 0.47 | 6.40E-01 | 0.7785 | 0.0429 | 504 |
| Right pars orbitalis | -0.513094 | 0.809263 | -0.63 | 5.26E-01 | 0.7379 | -0.0576 | 503 |
| Right pars triangularis | 1.887364 | 2.229871 | 0.85 | 3.98E-01 | 0.6403 | 0.0776 | 505 |
| Right pericalcarine | 1.115392 | 2.012968 | 0.55 | 5.80E-01 | 0.7571 | 0.0504 | 501 |
| Right precentral gyrus | 6.825419 | 3.960492 | 1.72 | 8.55E-02 | 0.3051 | 0.1572 | 504 |
| Right posterior cingulate cortex | 3.322555 | 1.480863 | 2.24 | 2.53E-02 | 0.1528 | 0.2041 | 507 |
| Right precentral gyrus | 5.953008 | 4.423001 | 1.35 | 1.79E-01 | 0.4531 | 0.1234 | 504 |
| Right precuneus | 4.590986 | 3.756874 | 1.22 | 2.22E-01 | 0.4986 | 0.1111 | 507 |
| Right rostral anterior cingulate cortex | 2.555289 | 1.121855 | 2.28 | 2.32E-02 | 0.1457 | 0.2081 | 505 |
| Right rostral middle frontal gyrus | -3.804302 | 5.438492 | -0.70 | 4.85E-01 | 0.7046 | -0.0638 | 507 |
| Right superior frontal gyrus | 3.208081 | 5.523759 | 0.58 | 5.62E-01 | 0.7544 | 0.0529 | 506 |
| Right superior parietal cortex | 5.160365 | 4.887719 | 1.06 | 2.92E-01 | 0.5547 | 0.0966 | 507 |
| Right superior temporal gyrus | 8.548639 | 2.924718 | 2.92 | 3.60E-03 | 0.1039 | 0.2726 | 484 |
| Right supramarginal gyrus | 6.277160 | 4.440353 | 1.41 | 1.58E-01 | 0.4356 | 0.1304 | 493 |
| Right frontal pole | 1.055703 | 0.445838 | 2.37 | 1.83E-02 | 0.1427 | 0.2159 | 507 |
| Right temporal pole | 0.674599 | 0.565702 | 1.19 | 2.34E-01 | 0.4992 | 0.1085 | 506 |
| Right transverse temporal gyrus | 1.035327 | 0.519075 | 1.99 | 4.67E-02 | 0.2222 | 0.1813 | 507 |
| Right insula | -1.335824 | 2.050473 | -0.65 | 5.15E-01 | 0.7351 | -0.0596 | 501 |

|  | **Estimate** | **StdError** | **T** | **p** | **R2** |
| --- | --- | --- | --- | --- | --- |
| **PRS (p= 0.1)** | 0.5488 | 0.0785 | 6.99 | 3.10E-12 | 0.0121 |
| **PRS (p= 0.2)** | 0.6622 | 0.0782 | 8.46 | <2.00E-16 | 0.0178 |
| **PRS (p= 0.3)** | 0.6025 | 0.0784 | 7.69 | 1.90E-14 | 0.0147 |
| **PRS (p= 0.4)** | 0.6076 | 0.0784 | 7.75 | 1.10E-14 | 0.0149 |
| **PRS (p= 0.5)** | 0.6173 | 0.0783 | 7.88 | 4.20E-15 | 0.0154 |
| **PRS (p= 0.6)** | 0.6036 | 0.0784 | 7.7 | 1.70E-14 | 0.0147 |
| **PRS (p= 0.7)** | 0.6075 | 0.0784 | 7.75 | 1.10E-14 | 0.0149 |
| **PRS (p= 0.8)** | 0.5977 | 0.0784 | 7.63 | 3.00E-14 | 0.0144 |
| **PRS (p= 0.9)** | 0.5971 | 0.0784 | 7.62 | 3.20E-14 | 0.0144 |
| **PRS (p= 1.0)** | 0.5963 | 0.0784 | 7.61 | 3.50E-14 | 0.0144 |

|  | **Estimate** | **StdError** | **T** | **p** | **R2** |
| --- | --- | --- | --- | --- | --- |
| **PRS (p= 0.1)** | 0.554 | 0.0744 | 7.44 | 1.20E-13 | 0.0121 |
| **PRS (p= 0.2)** | 0.67139 | 0.07404 | 9.07 | <2E-16 | 0.0178 |
| **PRS (p= 0.3)** | 0.61787 | 0.07421 | 8.33 | <2E-16 | 0.0147 |
| **PRS (p= 0.4)** | 0.61712 | 0.07422 | 8.31 | <2E-16 | 0.0149 |
| **PRS (p= 0.5)** | 0.62648 | 0.07418 | 8.44 | <2E-16 | 0.0154 |
| **PRS (p= 0.6)** | 0.61587 | 0.07418 | 8.3 | <2E-16 | 0.0147 |
| **PRS (p= 0.7)** | 0.62476 | 0.07412 | 8.43 | <2E-16 | 0.0149 |
| **PRS (p= 0.8)** | 0.6163 | 0.07414 | 8.31 | <2E-16 | 0.0144 |
| **PRS (p= 0.9)** | 0.61655 | 0.07414 | 8.32 | <2E-16 | 0.0144 |
| **PRS (p= 1.0)** | 0.61634 | 0.07414 | 8.31 | <2E-16 | 0.0144 |

| **Label** | **Estimate** | **StdError** | **T** | **p** | **FDR adjusted p** | **Cohen´s d** | **N** |
| --- | --- | --- | --- | --- | --- | --- | --- |
|  |  |  |  |  |  |  |  |
| **Global measures** |  |  |  |  |  |  |  |
| Total Intracranial Volume | -3022.00000 | 3471.000000 | -0.87 | 3.84E-01 | 0.7264 | -0.0290 | 3615 |
| Total left hemispheral surface area | -165.920000 | 160.540000 | -1.03 | 3.01E-01 | 0.7264 | -0.0334 | 3828 |
| Total right hemispheral surface area | -195.550000 | 161.580000 | -1.21 | 2.26E-01 | 0.7264 | -0.0392 | 3828 |
| Left hemispheral average thickness | -0.002298 | 0.002048 | -1.12 | 2.60E-01 | 0.7264 | -0.0362 | 3834 |
| Right hemispheral average thickness | -0.002174 | 0.002054 | -1.06 | 2.90E-01 | 0.7264 | -0.0343 | 3833 |
|  |  |  |  |  |  |  |  |
| **Cortical thickness** |  |  |  |  |  |  |  |
| Left banks of the superior temporal sulcus | -0.009163 | 0.003949 | -2.32 | 2.00E-02 | 0.3925 | -0.0771 | 3632 |
| Left caudal anterior cingulate cortex | 0.000638 | 0.005341 | 0.12 | 9.00E-01 | 0.9813 | 0.0039 | 3822 |
| Left caudal middle frontal gyrus | 0.001925 | 0.003250 | 0.59 | 5.50E-01 | 0.8525 | 0.0191 | 3820 |
| Left cuneus | -0.003607 | 0.002997 | -1.20 | 2.30E-01 | 0.7264 | -0.0390 | 3795 |
| Left entorhinal cortex | -0.015933 | 0.008402 | -1.90 | 5.80E-02 | 0.4351 | -0.0626 | 3697 |
| Left fusiform gyrus | -0.007320 | 0.003311 | -2.21 | 2.71E-02 | 0.4305 | -0.0716 | 3823 |
| Left inferior parietal cortex | 0.000039 | 0.002879 | 0.01 | 9.89E-01 | 0.9893 | 0.0003 | 3806 |
| Left inferior temporal gyrus | -0.005330 | 0.003700 | -1.44 | 1.50E-01 | 0.6708 | -0.0470 | 3766 |
| Left isthmus cingulate cortex | -0.005790 | 0.004493 | -1.29 | 1.98E-01 | 0.7136 | -0.0418 | 3824 |
| Left lateral occipital cortex | 0.001096 | 0.002676 | 0.41 | 6.82E-01 | 0.8990 | 0.0133 | 3820 |
| Left lateral orbitofrontal cortex | -0.009839 | 0.003562 | -2.76 | 5.80E-03 | 0.2277 | -0.0896 | 3812 |
| Left lingual gyrus | -0.001790 | 0.002690 | -0.66 | 5.07E-01 | 0.8525 | -0.0214 | 3807 |
| Left medial orbitofrontal cortex | -0.003798 | 0.003493 | -1.09 | 2.80E-01 | 0.7264 | -0.0354 | 3801 |
| Left middle temporal gyrus | -0.005288 | 0.003800 | -1.39 | 1.64E-01 | 0.6708 | -0.0458 | 3695 |
| Left parahippcampal gyrus | -0.004166 | 0.007154 | -0.58 | 5.60E-01 | 0.8525 | -0.0188 | 3824 |
| Left paracentral lobule | -0.005005 | 0.003412 | -1.47 | 1.42E-01 | 0.6578 | -0.0476 | 3825 |
| Left pars opercularis | -0.003296 | 0.003273 | -1.01 | 3.14E-01 | 0.7264 | -0.0327 | 3820 |
| Left pars orbitalis | -0.005749 | 0.004989 | -1.15 | 2.50E-01 | 0.7264 | -0.0373 | 3824 |
| Left pars triangularis | -0.002310 | 0.003637 | -0.64 | 5.30E-01 | 0.8525 | -0.0207 | 3821 |
| Left pericalcarine | 0.002119 | 0.002887 | 0.73 | 4.63E-01 | 0.8012 | 0.0238 | 3787 |
| Left precentral gyrus | 0.000242 | 0.002644 | 0.09 | 9.27E-01 | 0.9866 | 0.0029 | 3793 |
| Left posterior cingulate cortex | -0.004571 | 0.003564 | -1.28 | 2.00E-01 | 0.7136 | -0.0415 | 3826 |
| Left precentral gyrus | -0.004683 | 0.003108 | -1.51 | 1.32E-01 | 0.6280 | -0.0490 | 3806 |
| Left precuneus | -0.001440 | 0.002860 | -0.50 | 6.14E-01 | 0.8607 | -0.0162 | 3826 |
| Left rostral anterior cingulate cortex | -0.007300 | 0.005190 | -1.41 | 1.60E-01 | 0.6708 | -0.0458 | 3812 |
| Left rostral middle frontal gyrus | -0.001950 | 0.002996 | -0.65 | 5.20E-01 | 0.8525 | -0.0211 | 3826 |
| Left superior frontal gyrus | -0.000846 | 0.003115 | -0.27 | 7.86E-01 | 0.9278 | -0.0088 | 3821 |
| Left superior parietal cortex | -0.000112 | 0.002560 | -0.04 | 9.70E-01 | 0.9893 | -0.0013 | 3822 |
| Left superior temporal gyrus | -0.003641 | 0.003695 | -0.99 | 3.25E-01 | 0.7264 | -0.0329 | 3635 |
| Left supramarginal gyrus | -0.002791 | 0.003074 | -0.91 | 3.60E-01 | 0.7264 | -0.0298 | 3754 |
| Left frontal pole | -0.002089 | 0.006833 | -0.31 | 7.60E-01 | 0.9277 | -0.0100 | 3830 |
| Left temporal pole | 0.003280 | 0.008758 | 0.37 | 7.08E-01 | 0.8990 | 0.0120 | 3809 |
| Left transverse temporal gyrus | -0.012323 | 0.004997 | -2.47 | 1.40E-02 | 0.3663 | -0.0801 | 3813 |
| Left insula | -0.006626 | 0.003747 | -1.77 | 7.71E-02 | 0.5044 | -0.0576 | 3787 |
| Right banks of the superior temporal sulcus | -0.002878 | 0.003933 | -0.73 | 4.64E-01 | 0.8012 | -0.0238 | 3765 |
| Right caudal anterior cingulate cortex | -0.005070 | 0.005050 | -1.00 | 3.16E-01 | 0.7264 | -0.0324 | 3824 |
| Right caudal middle frontal gyrus | 0.001214 | 0.003281 | 0.37 | 7.10E-01 | 0.8990 | 0.0120 | 3823 |
| Right cuneus | -0.003548 | 0.003026 | -1.17 | 2.41E-01 | 0.7264 | -0.0381 | 3779 |
| Right entorhinal cortex | -0.007775 | 0.009111 | -0.85 | 3.93E-01 | 0.7294 | -0.0281 | 3661 |
| Right fusiform gyrus | -0.007251 | 0.003344 | -2.17 | 3.02E-02 | 0.4305 | -0.0703 | 3823 |
| Right inferior parietal cortex | 0.000199 | 0.002855 | 0.07 | 9.40E-01 | 0.9893 | 0.0023 | 3799 |
| Right inferior temporal gyrus | -0.007312 | 0.003801 | -1.92 | 5.45E-02 | 0.4351 | -0.0625 | 3790 |
| Right isthmus cingulate cortex | -0.002452 | 0.004410 | -0.56 | 5.78E-01 | 0.8565 | -0.0181 | 3821 |
| Right lateral occipital cortex | 0.002765 | 0.002774 | 1.00 | 3.19E-01 | 0.7264 | 0.0324 | 3821 |
| Right lateral orbitofrontal cortex | -0.006742 | 0.003595 | -1.88 | 6.10E-02 | 0.4353 | -0.0609 | 3824 |
| Right lingual gyrus | -0.000803 | 0.002685 | -0.30 | 7.65E-01 | 0.9277 | -0.0097 | 3808 |
| Right medial orbitofrontal cortex | -0.007293 | 0.003593 | -2.03 | 4.24E-02 | 0.4351 | -0.0659 | 3807 |
| Right middle temporal gyrus | -0.006713 | 0.003732 | -1.80 | 7.21E-02 | 0.4923 | -0.0585 | 3799 |
| Right parahippcampal gyrus | -0.004930 | 0.006115 | -0.81 | 4.20E-01 | 0.7669 | -0.0262 | 3831 |
| Right paracentral lobule | -0.005237 | 0.003375 | -1.55 | 1.21E-01 | 0.6280 | -0.0502 | 3825 |
| Right pars opercularis | -0.005170 | 0.003414 | -1.51 | 1.30E-01 | 0.6280 | -0.0489 | 3826 |
| Right pars orbitalis | -0.003560 | 0.004870 | -0.73 | 4.60E-01 | 0.8012 | -0.0236 | 3830 |
| Right pars triangularis | -0.001009 | 0.003470 | -0.29 | 7.70E-01 | 0.9277 | -0.0094 | 3819 |
| Right pericalcarine | 0.001587 | 0.002933 | 0.54 | 5.90E-01 | 0.8577 | 0.0176 | 3766 |
| Right precentral gyrus | 0.004343 | 0.002693 | 1.61 | 1.07E-01 | 0.6280 | 0.0523 | 3799 |
| Right posterior cingulate cortex | -0.003069 | 0.003468 | -0.88 | 3.76E-01 | 0.7264 | -0.0285 | 3827 |
| Right precentral gyrus | -0.003004 | 0.003177 | -0.95 | 3.45E-01 | 0.7264 | -0.0309 | 3804 |
| Right precuneus | -0.003654 | 0.002798 | -1.31 | 1.90E-01 | 0.7102 | -0.0425 | 3822 |
| Right rostral anterior cingulate cortex | -0.005545 | 0.005135 | -1.08 | 2.80E-01 | 0.7264 | -0.0351 | 3803 |
| Right rostral middle frontal gyrus | -0.001730 | 0.002954 | -0.59 | 5.58E-01 | 0.8525 | -0.0191 | 3817 |
| Right superior frontal gyrus | -0.001630 | 0.003152 | -0.52 | 6.10E-01 | 0.8607 | -0.0169 | 3821 |
| Right superior parietal cortex | -0.000554 | 0.002585 | -0.21 | 8.30E-01 | 0.9444 | -0.0068 | 3819 |
| Right superior temporal gyrus | 0.000198 | 0.003630 | 0.05 | 9.57E-01 | 0.9893 | 0.0016 | 3726 |
| Right supramarginal gyrus | -0.001474 | 0.003046 | -0.48 | 6.29E-01 | 0.8739 | -0.0157 | 3765 |
| Right frontal pole | 0.000371 | 0.006658 | 0.06 | 9.56E-01 | 0.9893 | 0.0019 | 3825 |
| Right temporal pole | -0.005565 | 0.009135 | -0.61 | 5.42E-01 | 0.8525 | -0.0198 | 3812 |
| Right transverse temporal gyrus | -0.005495 | 0.005291 | -1.04 | 2.99E-01 | 0.7264 | -0.0338 | 3811 |
| Right insula | -0.004556 | 0.003942 | -1.16 | 2.48E-01 | 0.7264 | -0.0379 | 3756 |
|  |  |  |  |  |  |  |  |
| **Subcortical volumes** |  |  |  |  |  |  |  |
| Left lateral ventricle | 137.264701 | 111.736931 | 1.23 | 2.19E-01 | 0.7264 | 0.0412 | 3571 |
| Right lateral ventricle | 106.840126 | 102.996802 | 1.04 | 3.00E-01 | 0.7264 | 0.0349 | 3562 |
| Left thalamus | 22.597937 | 16.498755 | 1.37 | 1.71E-01 | 0.6708 | 0.0460 | 3568 |
| Right thalamus | 20.778390 | 13.725382 | 1.51 | 1.30E-01 | 0.6280 | 0.0507 | 3562 |
| Left caudate | 5.800975 | 9.298253 | 0.62 | 5.33E-01 | 0.8525 | 0.0208 | 3566 |
| Right caudate | 6.384681 | 9.494831 | 0.67 | 5.01E-01 | 0.8525 | 0.0226 | 3531 |
| Left putamen | 19.560563 | 12.290794 | 1.59 | 1.12E-01 | 0.6280 | 0.0543 | 3449 |
| Right putamen | 28.048779 | 11.397902 | 2.46 | 1.40E-02 | 0.3663 | 0.0832 | 3512 |
| Left pallidum | -4.272508 | 4.791377 | -0.89 | 3.73E-01 | 0.7264 | -0.0308 | 3361 |
| Right pallidum | -0.177585 | 4.312423 | -0.04 | 9.67E-01 | 0.9893 | -0.0013 | 3526 |
| Left hippocampus | 8.516739 | 8.820086 | 0.97 | 3.34E-01 | 0.7264 | 0.0326 | 3549 |
| Right hippocamups | 8.371903 | 8.747367 | 0.96 | 3.39E-01 | 0.7264 | 0.0322 | 3568 |
| Left amygdala | -0.881309 | 4.106187 | -0.21 | 8.30E-01 | 0.9444 | -0.0071 | 3533 |
| Right amygdala | 4.035177 | 4.417208 | 0.91 | 3.61E-01 | 0.7264 | 0.0307 | 3537 |
| Left nucleus accumbens | 1.350768 | 2.094065 | 0.65 | 5.19E-01 | 0.8525 | 0.0220 | 3493 |
| Right nucleus accumbens | -1.979483 | 1.942080 | -1.02 | 3.08E-01 | 0.7264 | -0.0346 | 3499 |
|  |  |  |  |  |  |  |  |
| **Cortical surface area** |  |  |  |  |  |  |  |
| Left banks of the superior temporal sulcus | 8.479138 | 3.628057 | 2.34 | 1.90E-02 | 0.3925 | 0.0817 | 3298 |
| Left caudal anterior cingulate cortex | -0.751532 | 3.207634 | -0.23 | 8.15E-01 | 0.9406 | -0.0078 | 3504 |
| Left caudal middle frontal gyrus | 3.395978 | 8.059321 | 0.42 | 6.74E-01 | 0.8990 | 0.0142 | 3503 |
| Left cuneus | -4.229202 | 4.970837 | -0.85 | 3.95E-01 | 0.7294 | -0.0288 | 3496 |
| Left entorhinal cortex | 2.584034 | 1.813832 | 1.42 | 1.54E-01 | 0.6708 | 0.0493 | 3332 |
| Left fusiform gyrus | -4.793406 | 7.983491 | -0.60 | 5.48E-01 | 0.8525 | -0.0204 | 3461 |
| Left inferior parietal cortex | -7.697724 | 12.976098 | -0.59 | 5.53E-01 | 0.8525 | -0.0200 | 3484 |
| Left inferior temporal gyrus | -5.003294 | 9.271762 | -0.54 | 5.89E-01 | 0.8577 | -0.0184 | 3473 |
| Left isthmus cingulate cortex | 2.063116 | 3.582271 | 0.58 | 5.65E-01 | 0.8525 | 0.0196 | 3527 |
| Left lateral occipital cortex | -45.924566 | 12.557722 | -3.66 | 2.60E-04 | 0.0408 | -0.1235 | 3526 |
| Left lateral orbitofrontal cortex | 0.689686 | 5.466988 | 0.13 | 9.00E-01 | 0.9813 | 0.0044 | 3544 |
| Left lingual gyrus | -11.838384 | 8.899076 | -1.33 | 1.84E-01 | 0.7046 | -0.0449 | 3521 |
| Left medial orbitofrontal cortex | -0.106874 | 4.938208 | -0.02 | 9.83E-01 | 0.9893 | -0.0007 | 3492 |
| Left middle temporal gyrus | -3.205616 | 8.092592 | -0.40 | 6.92E-01 | 0.8990 | -0.0138 | 3365 |
| Left parahippcampal gyrus | 4.149313 | 2.034305 | 2.04 | 4.15E-02 | 0.4351 | 0.0689 | 3515 |
| Left paracentral lobule | 5.040742 | 4.108017 | 1.23 | 2.20E-01 | 0.7264 | 0.0418 | 3480 |
| Left pars opercularis | -0.108560 | 5.787904 | -0.02 | 9.85E-01 | 0.9893 | -0.0007 | 3517 |
| Left pars orbitalis | -0.660644 | 1.735405 | -0.38 | 7.00E-01 | 0.8990 | -0.0128 | 3538 |
| Left pars triangularis | -8.544960 | 4.267278 | -2.00 | 4.53E-02 | 0.4351 | -0.0675 | 3527 |
| Left pericalcarine | -11.238165 | 5.432625 | -2.07 | 3.87E-02 | 0.4351 | -0.0700 | 3506 |
| Left precentral gyrus | -16.734865 | 8.830564 | -1.90 | 5.82E-02 | 0.4351 | -0.0647 | 3467 |
| Left posterior cingulate cortex | 7.656927 | 3.964806 | 1.93 | 5.35E-02 | 0.4351 | 0.0651 | 3534 |
| Left precentral gyrus | 3.700729 | 10.465350 | 0.35 | 7.24E-01 | 0.9017 | 0.0119 | 3495 |
| Left precuneus | -8.206296 | 9.032632 | -0.91 | 3.64E-01 | 0.7264 | -0.0307 | 3524 |
| Left rostral anterior cingulate cortex | -1.162556 | 3.214723 | -0.36 | 7.18E-01 | 0.9014 | -0.0122 | 3474 |
| Left rostral middle frontal gyrus | -24.028814 | 14.164509 | -1.70 | 8.99E-02 | 0.5646 | -0.0573 | 3530 |
| Left superior frontal gyrus | -23.403584 | 15.471816 | -1.51 | 1.30E-01 | 0.6280 | -0.0513 | 3483 |
| Left superior parietal cortex | -6.153872 | 13.164309 | -0.47 | 6.40E-01 | 0.8814 | -0.0159 | 3503 |
| Left superior temporal gyrus | 1.334456 | 8.620893 | 0.15 | 8.77E-01 | 0.9813 | 0.0052 | 3323 |
| Left supramarginal gyrus | 11.453331 | 11.405303 | 1.00 | 3.15E-01 | 0.7264 | 0.0344 | 3397 |
| Left frontal pole | 0.278937 | 0.738513 | 0.38 | 7.06E-01 | 0.8990 | 0.0128 | 3543 |
| Left temporal pole | -0.127866 | 1.368164 | -0.09 | 9.30E-01 | 0.9866 | -0.0030 | 3529 |
| Left transverse temporal gyrus | 1.450908 | 1.629562 | 0.89 | 3.73E-01 | 0.7264 | 0.0300 | 3543 |
| Left insula | 1.609925 | 4.979266 | 0.32 | 7.50E-01 | 0.9272 | 0.0108 | 3501 |
| Right banks of the superior temporal sulcus | 4.071139 | 2.949754 | 1.38 | 1.68E-01 | 0.6708 | 0.0470 | 3459 |
| Right caudal anterior cingulate cortex | 0.891471 | 3.560649 | 0.25 | 8.02E-01 | 0.9400 | 0.0085 | 3514 |
| Right caudal middle frontal gyrus | -10.142311 | 8.072613 | -1.26 | 2.09E-01 | 0.7264 | -0.0426 | 3512 |
| Right cuneus | -5.132058 | 4.541350 | -1.13 | 2.59E-01 | 0.7264 | -0.0383 | 3486 |
| Right entorhinal cortex | -0.529317 | 1.847456 | -0.29 | 7.75E-01 | 0.9277 | -0.0102 | 3271 |
| Right fusiform gyrus | 1.132783 | 7.807451 | 0.15 | 8.85E-01 | 0.9813 | 0.0051 | 3460 |
| Right inferior parietal cortex | -8.207963 | 14.631230 | -0.56 | 5.75E-01 | 0.8565 | -0.0190 | 3478 |
| Right inferior temporal gyrus | -8.372715 | 8.893634 | -0.94 | 3.50E-01 | 0.7264 | -0.0318 | 3503 |
| Right isthmus cingulate cortex | -0.889462 | 3.244593 | -0.27 | 7.80E-01 | 0.9277 | -0.0091 | 3539 |
| Right lateral occipital cortex | -27.398546 | 12.452940 | -2.20 | 2.80E-02 | 0.4305 | -0.0742 | 3532 |
| Right lateral orbitofrontal cortex | -5.355716 | 5.982030 | -0.90 | 3.70E-01 | 0.7264 | -0.0303 | 3546 |
| Right lingual gyrus | -18.671389 | 8.762917 | -2.13 | 3.30E-02 | 0.4318 | -0.0719 | 3523 |
| Right medial orbitofrontal cortex | -4.441744 | 4.000005 | -1.11 | 2.67E-01 | 0.7264 | -0.0375 | 3513 |
| Right middle temporal gyrus | -4.258817 | 8.039266 | -0.53 | 5.96E-01 | 0.8589 | -0.0180 | 3478 |
| Right parahippcampal gyrus | 0.464717 | 1.968782 | 0.24 | 8.13E-01 | 0.9406 | 0.0081 | 3517 |
| Right paracentral lobule | 4.042958 | 4.614477 | 0.88 | 3.81E-01 | 0.7264 | 0.0298 | 3507 |
| Right pars opercularis | -4.583373 | 4.970237 | -0.92 | 3.57E-01 | 0.7264 | -0.0311 | 3511 |
| Right pars orbitalis | -6.578213 | 2.107827 | -3.12 | 1.80E-03 | 0.1413 | -0.1050 | 3543 |
| Right pars triangularis | -8.125762 | 5.146801 | -1.58 | 1.15E-01 | 0.6280 | -0.0534 | 3521 |
| Right pericalcarine | -16.151451 | 5.721404 | -2.82 | 4.78E-03 | 0.2277 | -0.0956 | 3492 |
| Right precentral gyrus | 4.570246 | 8.966412 | 0.51 | 6.10E-01 | 0.8607 | 0.0173 | 3479 |
| Right posterior cingulate cortex | 0.380131 | 3.771124 | 0.10 | 9.20E-01 | 0.9866 | 0.0034 | 3537 |
| Right precentral gyrus | -1.444359 | 11.019974 | -0.13 | 8.96E-01 | 0.9813 | -0.0044 | 3490 |
| Right precuneus | -7.418150 | 9.551063 | -0.78 | 4.37E-01 | 0.7893 | -0.0263 | 3531 |
| Right rostral anterior cingulate cortex | 0.063095 | 2.968576 | 0.02 | 9.83E-01 | 0.9893 | 0.0007 | 3496 |
| Right rostral middle frontal gyrus | -28.592967 | 14.636177 | -1.95 | 5.08E-02 | 0.4351 | -0.0658 | 3525 |
| Right superior frontal gyrus | 6.481753 | 15.558503 | 0.42 | 6.77E-01 | 0.8990 | 0.0142 | 3498 |
| Right superior parietal cortex | -5.492576 | 12.253001 | -0.45 | 6.54E-01 | 0.8929 | -0.0152 | 3505 |
| Right superior temporal gyrus | -0.819473 | 7.807429 | -0.10 | 9.16E-01 | 0.9866 | -0.0034 | 3420 |
| Right supramarginal gyrus | -11.570728 | 10.584494 | -1.09 | 2.74E-01 | 0.7264 | -0.0372 | 3447 |
| Right frontal pole | -0.165206 | 0.969924 | -0.17 | 8.60E-01 | 0.9714 | -0.0057 | 3541 |
| Right temporal pole | -1.007556 | 1.375508 | -0.73 | 4.64E-01 | 0.8012 | -0.0247 | 3498 |
| Right transverse temporal gyrus | -1.529340 | 1.278951 | -1.20 | 2.32E-01 | 0.7264 | -0.0404 | 3544 |
| Right insula | 2.390216 | 5.722353 | 0.42 | 6.80E-01 | 0.8990 | 0.0143 | 3470 |

| **Label** | **Estimate** | **StdError** | **T** | **p** | **FDR adjusted p** | **Cohen´s d** | **N** |
| --- | --- | --- | --- | --- | --- | --- | --- |
|  |  |  |  |  |  |  |  |
| **Global measures** |  |  |  |  |  |  |  |
| Total Intracranial Volume | -3223.00000 | 3181.000000 | -1.01 | 3.11E-01 | 0.7469 | -0.0337 | 3615 |
| Total left hemispheral surface area | -158.530000 | 146.710000 | -1.08 | 2.80E-01 | 0.7469 | -0.0350 | 3828 |
| Total right hemispheral surface area | -169.990000 | 147.660000 | -1.15 | 2.50E-01 | 0.7469 | -0.0372 | 3828 |
| Left hemispheral average thickness | -0.001261 | 0.001871 | -0.67 | 5.01E-01 | 0.8109 | -0.0217 | 3834 |
| Right hemispheral average thickness | -0.001449 | 0.001877 | -0.77 | 4.40E-01 | 0.7762 | -0.0249 | 3833 |
|  |  |  |  |  |  |  |  |
| **Cortical thickness** |  |  |  |  |  |  |  |
| Left banks of the superior temporal sulcus | -0.006012 | 0.003607 | -1.67 | 9.60E-02 | 0.5797 | -0.0555 | 3632 |
| Left caudal anterior cingulate cortex | -0.001842 | 0.004879 | -0.38 | 7.10E-01 | 0.9137 | -0.0123 | 3822 |
| Left caudal middle frontal gyrus | 0.003485 | 0.002968 | 1.17 | 2.40E-01 | 0.7469 | 0.0379 | 3820 |
| Left cuneus | -0.004134 | 0.002739 | -1.51 | 1.30E-01 | 0.5971 | -0.0491 | 3795 |
| Left entorhinal cortex | -0.008228 | 0.007679 | -1.07 | 2.84E-01 | 0.7469 | -0.0353 | 3697 |
| Left fusiform gyrus | -0.007378 | 0.003025 | -2.44 | 1.48E-02 | 0.2498 | -0.0791 | 3823 |
| Left inferior parietal cortex | 0.001486 | 0.002630 | 0.57 | 5.72E-01 | 0.8411 | 0.0185 | 3806 |
| Left inferior temporal gyrus | -0.008110 | 0.003380 | -2.40 | 1.65E-02 | 0.2498 | -0.0784 | 3766 |
| Left isthmus cingulate cortex | -0.001751 | 0.004104 | -0.43 | 6.70E-01 | 0.8991 | -0.0139 | 3824 |
| Left lateral occipital cortex | -0.000006 | 0.002445 | 0.00 | 9.98E-01 | 0.9980 | <0.0001 | 3820 |
| Left lateral orbitofrontal cortex | -0.006766 | 0.003255 | -2.08 | 3.80E-02 | 0.3827 | -0.0675 | 3812 |
| Left lingual gyrus | -0.001480 | 0.002460 | -0.60 | 5.47E-01 | 0.8318 | -0.0195 | 3807 |
| Left medial orbitofrontal cortex | -0.002328 | 0.003191 | -0.73 | 4.66E-01 | 0.7867 | -0.0237 | 3801 |
| Left middle temporal gyrus | -0.003861 | 0.003470 | -1.11 | 2.66E-01 | 0.7469 | -0.0366 | 3695 |
| Left parahippcampal gyrus | -0.000499 | 0.006537 | -0.08 | 9.39E-01 | 0.9829 | -0.0026 | 3824 |
| Left paracentral lobule | -0.002297 | 0.003116 | -0.74 | 4.61E-01 | 0.7867 | -0.0240 | 3825 |
| Left pars opercularis | -0.001407 | 0.002988 | -0.47 | 6.38E-01 | 0.8785 | -0.0152 | 3820 |
| Left pars orbitalis | -0.005093 | 0.004557 | -1.12 | 2.60E-01 | 0.7469 | -0.0363 | 3824 |
| Left pars triangularis | -0.000665 | 0.003323 | -0.20 | 8.40E-01 | 0.9626 | -0.0065 | 3821 |
| Left pericalcarine | 0.001646 | 0.002640 | 0.62 | 5.33E-01 | 0.8318 | 0.0202 | 3787 |
| Left precentral gyrus | 0.001302 | 0.002415 | 0.54 | 5.90E-01 | 0.8411 | 0.0176 | 3793 |
| Left posterior cingulate cortex | -0.000844 | 0.003258 | -0.26 | 7.96E-01 | 0.9600 | -0.0084 | 3826 |
| Left precentral gyrus | -0.002493 | 0.002837 | -0.88 | 3.80E-01 | 0.7559 | -0.0286 | 3806 |
| Left precuneus | 0.000496 | 0.002614 | 0.19 | 8.50E-01 | 0.9670 | 0.0062 | 3826 |
| Left rostral anterior cingulate cortex | -0.007574 | 0.004739 | -1.60 | 1.10E-01 | 0.5971 | -0.0519 | 3812 |
| Left rostral middle frontal gyrus | -0.001486 | 0.002737 | -0.54 | 5.90E-01 | 0.8411 | -0.0175 | 3826 |
| Left superior frontal gyrus | -0.000622 | 0.002846 | -0.22 | 8.27E-01 | 0.9626 | -0.0071 | 3821 |
| Left superior parietal cortex | -0.000236 | 0.002337 | -0.10 | 9.20E-01 | 0.9778 | -0.0032 | 3822 |
| Left superior temporal gyrus | -0.003058 | 0.003385 | -0.90 | 3.66E-01 | 0.7559 | -0.0299 | 3635 |
| Left supramarginal gyrus | 0.000151 | 0.002809 | 0.05 | 9.60E-01 | 0.9926 | 0.0016 | 3754 |
| Left frontal pole | -0.004216 | 0.006242 | -0.68 | 4.99E-01 | 0.8109 | -0.0220 | 3830 |
| Left temporal pole | 0.004309 | 0.008002 | 0.54 | 5.90E-01 | 0.8411 | 0.0175 | 3809 |
| Left transverse temporal gyrus | -0.009238 | 0.004567 | -2.02 | 4.30E-02 | 0.3971 | -0.0655 | 3813 |
| Left insula | -0.005106 | 0.003424 | -1.49 | 1.36E-01 | 0.5971 | -0.0485 | 3787 |
| Right banks of the superior temporal sulcus | -0.003923 | 0.003594 | -1.09 | 2.75E-01 | 0.7469 | -0.0356 | 3765 |
| Right caudal anterior cingulate cortex | -0.006320 | 0.004620 | -1.37 | 1.71E-01 | 0.6392 | -0.0444 | 3824 |
| Right caudal middle frontal gyrus | 0.003409 | 0.002998 | 1.14 | 2.60E-01 | 0.7469 | 0.0369 | 3823 |
| Right cuneus | -0.004120 | 0.002765 | -1.49 | 1.36E-01 | 0.5971 | -0.0486 | 3779 |
| Right entorhinal cortex | -0.007228 | 0.008319 | -0.87 | 3.85E-01 | 0.7559 | -0.0288 | 3661 |
| Right fusiform gyrus | -0.008476 | 0.003052 | -2.78 | 5.51E-03 | 0.2498 | -0.0901 | 3823 |
| Right inferior parietal cortex | 0.002100 | 0.002610 | 0.80 | 4.20E-01 | 0.7758 | 0.0260 | 3799 |
| Right inferior temporal gyrus | -0.009248 | 0.003473 | -2.66 | 7.80E-03 | 0.2498 | -0.0866 | 3790 |
| Right isthmus cingulate cortex | 0.002334 | 0.004028 | 0.58 | 5.62E-01 | 0.8408 | 0.0188 | 3821 |
| Right lateral occipital cortex | 0.001577 | 0.002535 | 0.62 | 5.34E-01 | 0.8318 | 0.0201 | 3821 |
| Right lateral orbitofrontal cortex | -0.003016 | 0.003285 | -0.92 | 3.59E-01 | 0.7559 | -0.0298 | 3824 |
| Right lingual gyrus | -0.001900 | 0.002450 | -0.78 | 4.38E-01 | 0.7762 | -0.0253 | 3808 |
| Right medial orbitofrontal cortex | -0.004791 | 0.003283 | -1.46 | 1.45E-01 | 0.6007 | -0.0474 | 3807 |
| Right middle temporal gyrus | -0.007498 | 0.003410 | -2.20 | 2.80E-02 | 0.3376 | -0.0715 | 3799 |
| Right parahippcampal gyrus | -0.004853 | 0.005586 | -0.87 | 3.85E-01 | 0.7559 | -0.0282 | 3831 |
| Right paracentral lobule | -0.003281 | 0.003084 | -1.06 | 2.87E-01 | 0.7469 | -0.0343 | 3825 |
| Right pars opercularis | -0.002289 | 0.003119 | -0.73 | 4.63E-01 | 0.7867 | -0.0236 | 3826 |
| Right pars orbitalis | -0.001620 | 0.004450 | -0.37 | 7.20E-01 | 0.9190 | -0.0120 | 3830 |
| Right pars triangularis | 0.000512 | 0.003172 | 0.16 | 8.72E-01 | 0.9778 | 0.0052 | 3819 |
| Right pericalcarine | 0.000547 | 0.002677 | 0.20 | 8.40E-01 | 0.9626 | 0.0065 | 3766 |
| Right precentral gyrus | 0.004100 | 0.002460 | 1.67 | 9.56E-02 | 0.5797 | 0.0543 | 3799 |
| Right posterior cingulate cortex | -0.002016 | 0.003169 | -0.64 | 5.25E-01 | 0.8318 | -0.0207 | 3827 |
| Right precentral gyrus | -0.001527 | 0.002899 | -0.53 | 5.98E-01 | 0.8411 | -0.0172 | 3804 |
| Right precuneus | -0.001974 | 0.002556 | -0.77 | 4.40E-01 | 0.7762 | -0.0250 | 3822 |
| Right rostral anterior cingulate cortex | -0.002282 | 0.004692 | -0.49 | 6.27E-01 | 0.8711 | -0.0159 | 3803 |
| Right rostral middle frontal gyrus | -0.000065 | 0.002699 | -0.02 | 9.80E-01 | 0.9926 | -0.0006 | 3817 |
| Right superior frontal gyrus | 0.000105 | 0.002880 | 0.04 | 9.70E-01 | 0.9926 | 0.0013 | 3821 |
| Right superior parietal cortex | 0.000529 | 0.002361 | 0.22 | 8.20E-01 | 0.9626 | 0.0071 | 3819 |
| Right superior temporal gyrus | -0.000423 | 0.003318 | -0.13 | 8.99E-01 | 0.9778 | -0.0043 | 3726 |
| Right supramarginal gyrus | -0.000419 | 0.002786 | -0.15 | 8.80E-01 | 0.9778 | -0.0049 | 3765 |
| Right frontal pole | -0.005501 | 0.006082 | -0.90 | 3.66E-01 | 0.7559 | -0.0292 | 3825 |
| Right temporal pole | -0.007309 | 0.008347 | -0.88 | 3.81E-01 | 0.7559 | -0.0286 | 3812 |
| Right transverse temporal gyrus | -0.005617 | 0.004834 | -1.16 | 2.45E-01 | 0.7469 | -0.0376 | 3811 |
| Right insula | -0.003926 | 0.003602 | -1.09 | 2.76E-01 | 0.7469 | -0.0356 | 3756 |
|  |  |  |  |  |  |  |  |
| **Subcortical volumes** |  |  |  |  |  |  |  |
| Left lateral ventricle | 178.357502 | 102.258501 | 1.74 | 8.12E-02 | 0.5539 | 0.0583 | 3571 |
| Right lateral ventricle | 182.098155 | 94.295272 | 1.93 | 5.35E-02 | 0.4000 | 0.0648 | 3562 |
| Left thalamus | 35.496376 | 15.124856 | 2.35 | 1.90E-02 | 0.2498 | 0.0788 | 3568 |
| Right thalamus | 30.711597 | 12.575890 | 2.44 | 1.50E-02 | 0.2498 | 0.0819 | 3562 |
| Left caudate | 12.777842 | 8.526175 | 1.50 | 1.34E-01 | 0.5971 | 0.0503 | 3566 |
| Right caudate | 11.275844 | 8.714720 | 1.29 | 1.96E-01 | 0.7149 | 0.0435 | 3531 |
| Left putamen | 27.082560 | 11.279642 | 2.40 | 1.64E-02 | 0.2498 | 0.0819 | 3449 |
| Right putamen | 27.008424 | 10.475305 | 2.58 | 1.00E-02 | 0.2498 | 0.0872 | 3512 |
| Left pallidum | -4.438683 | 4.394772 | -1.01 | 3.13E-01 | 0.7469 | -0.0349 | 3361 |
| Right pallidum | 1.042479 | 3.952258 | 0.26 | 7.92E-01 | 0.9600 | 0.0088 | 3526 |
| Left hippocampus | 6.990158 | 8.084666 | 0.86 | 3.87E-01 | 0.7559 | 0.0289 | 3549 |
| Right hippocamups | 1.624632 | 8.021469 | 0.20 | 8.40E-01 | 0.9626 | 0.0067 | 3568 |
| Left amygdala | -1.111604 | 3.766882 | -0.30 | 7.68E-01 | 0.9568 | -0.0101 | 3533 |
| Right amygdala | 3.305014 | 4.044373 | 0.82 | 4.14E-01 | 0.7758 | 0.0276 | 3537 |
| Left nucleus accumbens | 2.796133 | 1.920041 | 1.46 | 1.45E-01 | 0.6007 | 0.0495 | 3493 |
| Right nucleus accumbens | 0.046081 | 1.780077 | 0.03 | 9.79E-01 | 0.9926 | 0.0010 | 3499 |
|  |  |  |  |  |  |  |  |
| **Cortical surface area** |  |  |  |  |  |  |  |
| Left banks of the superior temporal sulcus | 8.197853 | 3.323462 | 2.47 | 1.40E-02 | 0.2498 | 0.0862 | 3298 |
| Left caudal anterior cingulate cortex | -0.325288 | 2.939197 | -0.11 | 9.12E-01 | 0.9778 | -0.0037 | 3504 |
| Left caudal middle frontal gyrus | -0.664597 | 7.383534 | -0.09 | 9.28E-01 | 0.9778 | -0.0030 | 3503 |
| Left cuneus | -4.192598 | 4.556884 | -0.92 | 3.58E-01 | 0.7559 | -0.0312 | 3496 |
| Left entorhinal cortex | 3.261515 | 1.665792 | 1.96 | 5.00E-02 | 0.4000 | 0.0680 | 3332 |
| Left fusiform gyrus | -2.886424 | 7.340840 | -0.39 | 6.94E-01 | 0.9082 | -0.0133 | 3461 |
| Left inferior parietal cortex | -3.596809 | 11.906083 | -0.30 | 7.63E-01 | 0.9568 | -0.0102 | 3484 |
| Left inferior temporal gyrus | -6.720091 | 8.511823 | -0.79 | 4.30E-01 | 0.7762 | -0.0269 | 3473 |
| Left isthmus cingulate cortex | -0.326415 | 3.283657 | -0.10 | 9.21E-01 | 0.9778 | -0.0034 | 3527 |
| Left lateral occipital cortex | -40.838930 | 11.520052 | -3.55 | 4.00E-04 | 0.0628 | -0.1198 | 3526 |
| Left lateral orbitofrontal cortex | -1.261714 | 5.011762 | -0.25 | 8.00E-01 | 0.9600 | -0.0084 | 3544 |
| Left lingual gyrus | -12.522662 | 8.163568 | -1.53 | 1.25E-01 | 0.5971 | -0.0517 | 3521 |
| Left medial orbitofrontal cortex | -0.162041 | 4.528289 | -0.04 | 9.71E-01 | 0.9926 | -0.0014 | 3492 |
| Left middle temporal gyrus | -0.080274 | 7.418951 | -0.01 | 9.91E-01 | 0.9974 | -0.0003 | 3365 |
| Left parahippcampal gyrus | 3.295408 | 1.865499 | 1.77 | 7.70E-02 | 0.5495 | 0.0598 | 3515 |
| Left paracentral lobule | 3.453370 | 3.767213 | 0.92 | 3.59E-01 | 0.7559 | 0.0312 | 3480 |
| Left pars opercularis | -1.363379 | 5.305910 | -0.26 | 7.97E-01 | 0.9600 | -0.0088 | 3517 |
| Left pars orbitalis | 0.183716 | 1.590910 | 0.12 | 9.10E-01 | 0.9778 | 0.0040 | 3538 |
| Left pars triangularis | -4.772332 | 3.913184 | -1.22 | 2.23E-01 | 0.7469 | -0.0412 | 3527 |
| Left pericalcarine | -11.680759 | 4.981465 | -2.34 | 1.91E-02 | 0.2498 | -0.0792 | 3506 |
| Left precentral gyrus | -17.153161 | 8.102027 | -2.12 | 3.43E-02 | 0.3827 | -0.0721 | 3467 |
| Left posterior cingulate cortex | 7.079524 | 3.635433 | 1.95 | 5.16E-02 | 0.4000 | 0.0657 | 3534 |
| Left precentral gyrus | 7.743671 | 9.588993 | 0.81 | 4.19E-01 | 0.7758 | 0.0275 | 3495 |
| Left precuneus | -8.564401 | 8.283104 | -1.03 | 3.01E-01 | 0.7469 | -0.0348 | 3524 |
| Left rostral anterior cingulate cortex | -3.240077 | 2.946419 | -1.10 | 2.72E-01 | 0.7469 | -0.0374 | 3474 |
| Left rostral middle frontal gyrus | -16.247876 | 12.987965 | -1.25 | 2.11E-01 | 0.7469 | -0.0422 | 3530 |
| Left superior frontal gyrus | -15.900495 | 14.181738 | -1.12 | 2.62E-01 | 0.7469 | -0.0380 | 3483 |
| Left superior parietal cortex | -4.722512 | 12.072852 | -0.39 | 7.00E-01 | 0.9083 | -0.0132 | 3503 |
| Left superior temporal gyrus | 6.923890 | 7.926056 | 0.87 | 3.82E-01 | 0.7559 | 0.0302 | 3323 |
| Left supramarginal gyrus | 14.968720 | 10.469133 | 1.43 | 1.50E-01 | 0.6038 | 0.0492 | 3397 |
| Left frontal pole | 0.458746 | 0.676881 | 0.68 | 4.98E-01 | 0.8109 | 0.0229 | 3543 |
| Left temporal pole | 0.559280 | 1.254384 | 0.45 | 6.60E-01 | 0.8991 | 0.0152 | 3529 |
| Left transverse temporal gyrus | 1.532705 | 1.493872 | 1.03 | 3.05E-01 | 0.7469 | 0.0347 | 3543 |
| Left insula | 1.963434 | 4.566546 | 0.43 | 6.70E-01 | 0.8991 | 0.0146 | 3501 |
| Right banks of the superior temporal sulcus | 4.067965 | 2.701261 | 1.51 | 1.32E-01 | 0.5971 | 0.0514 | 3459 |
| Right caudal anterior cingulate cortex | 1.333832 | 3.264037 | 0.41 | 6.83E-01 | 0.9082 | 0.0139 | 3514 |
| Right caudal middle frontal gyrus | -10.443886 | 7.401339 | -1.41 | 1.58E-01 | 0.6062 | -0.0477 | 3512 |
| Right cuneus | -4.172495 | 4.166820 | -1.00 | 3.20E-01 | 0.7499 | -0.0339 | 3486 |
| Right entorhinal cortex | -0.384640 | 1.693191 | -0.23 | 8.20E-01 | 0.9626 | -0.0081 | 3271 |
| Right fusiform gyrus | 4.374378 | 7.160477 | 0.61 | 5.41E-01 | 0.8318 | 0.0208 | 3460 |
| Right inferior parietal cortex | 7.999175 | 13.409001 | 0.60 | 5.51E-01 | 0.8318 | 0.0204 | 3478 |
| Right inferior temporal gyrus | -13.439785 | 8.156807 | -1.65 | 1.00E-01 | 0.5815 | -0.0559 | 3503 |
| Right isthmus cingulate cortex | -2.469189 | 2.974651 | -0.83 | 4.10E-01 | 0.7758 | -0.0280 | 3539 |
| Right lateral occipital cortex | -17.836313 | 11.425211 | -1.56 | 1.20E-01 | 0.5971 | -0.0526 | 3532 |
| Right lateral orbitofrontal cortex | -5.934231 | 5.483533 | -1.08 | 2.80E-01 | 0.7469 | -0.0363 | 3546 |
| Right lingual gyrus | -16.562296 | 8.033429 | -2.06 | 3.90E-02 | 0.3827 | -0.0695 | 3523 |
| Right medial orbitofrontal cortex | -3.728974 | 3.661882 | -1.02 | 3.09E-01 | 0.7469 | -0.0345 | 3513 |
| Right middle temporal gyrus | -1.143913 | 7.375370 | -0.16 | 8.77E-01 | 0.9778 | -0.0054 | 3478 |
| Right parahippcampal gyrus | 1.611155 | 1.805689 | 0.89 | 3.72E-01 | 0.7559 | 0.0301 | 3517 |
| Right paracentral lobule | 4.262160 | 4.231916 | 1.01 | 3.14E-01 | 0.7469 | 0.0342 | 3507 |
| Right pars opercularis | -4.302173 | 4.556949 | -0.94 | 3.45E-01 | 0.7559 | -0.0318 | 3511 |
| Right pars orbitalis | -3.763266 | 1.934304 | -1.95 | 5.18E-02 | 0.4000 | -0.0656 | 3543 |
| Right pars triangularis | -5.678089 | 4.720186 | -1.20 | 2.29E-01 | 0.7469 | -0.0405 | 3521 |
| Right pericalcarine | -14.339893 | 5.244613 | -2.73 | 6.30E-03 | 0.2498 | -0.0926 | 3492 |
| Right precentral gyrus | 4.436554 | 8.239765 | 0.54 | 5.90E-01 | 0.8411 | 0.0183 | 3479 |
| Right posterior cingulate cortex | -0.872567 | 3.459013 | -0.25 | 8.01E-01 | 0.9600 | -0.0084 | 3537 |
| Right precentral gyrus | -0.951802 | 10.091935 | -0.09 | 9.25E-01 | 0.9778 | -0.0031 | 3490 |
| Right precuneus | -7.489661 | 8.758376 | -0.86 | 3.90E-01 | 0.7559 | -0.0290 | 3531 |
| Right rostral anterior cingulate cortex | 0.316070 | 2.719851 | 0.12 | 9.08E-01 | 0.9778 | 0.0041 | 3496 |
| Right rostral middle frontal gyrus | -23.154411 | 13.425600 | -1.72 | 8.47E-02 | 0.5539 | -0.0580 | 3525 |
| Right superior frontal gyrus | 10.488017 | 14.269606 | 0.73 | 4.62E-01 | 0.7867 | 0.0247 | 3498 |
| Right superior parietal cortex | -6.782977 | 11.249212 | -0.60 | 5.47E-01 | 0.8318 | -0.0203 | 3505 |
| Right superior temporal gyrus | 2.826210 | 7.154377 | 0.40 | 6.93E-01 | 0.9082 | 0.0137 | 3420 |
| Right supramarginal gyrus | -14.472862 | 9.728214 | -1.49 | 1.37E-01 | 0.5971 | -0.0509 | 3447 |
| Right frontal pole | 0.305563 | 0.889140 | 0.34 | 7.31E-01 | 0.9255 | 0.0114 | 3541 |
| Right temporal pole | -0.902938 | 1.261084 | -0.72 | 4.74E-01 | 0.7917 | -0.0244 | 3498 |
| Right transverse temporal gyrus | -1.654654 | 1.172474 | -1.41 | 1.58E-01 | 0.6062 | -0.0475 | 3544 |
| Right insula | 2.766983 | 5.246195 | 0.53 | 6.00E-01 | 0.8411 | 0.0180 | 3470 |
